# Novel Regioselective Synthesis of 1,3,4,5-Tetrasubstituted Pyrazoles and Biochemical Valuation on F_1_F_O_-ATPase and Mitochondrial Permeability Transition Pore Formation

**DOI:** 10.3390/pharmaceutics15020498

**Published:** 2023-02-02

**Authors:** Vincenzo Algieri, Cristina Algieri, Paola Costanzo, Giulia Fiorani, Antonio Jiritano, Fabrizio Olivito, Matteo Antonio Tallarida, Fabiana Trombetti, Loredana Maiuolo, Antonio De Nino, Salvatore Nesci

**Affiliations:** 1 Department of Chemistry and Chemical Technologies, University of Calabria, Via P. Bucci, Cubo 12C, 87036 Rende, CS, Italy; 2 Department of Veterinary Medical Sciences, Mitochondrial Biochemistry Lab, Via Tolara di Sopra, 50, 40064 Ozzano Emilia, BO, Italy; 3 Department Molecular Sciences and Nanosystems, University Ca’ Foscari Venezia, Via Torino 155, 30172 Venezia Mestre, VE, Italy

**Keywords:** pyrazoles, 1,3-dipolar cycloaddition, hydrazonyl chlorides, mitochondrial permeability transition pore, F_1_F_O_-ATPase

## Abstract

An efficient, eco-compatible, and very cheap method for the construction of fully substituted pyrazoles (Pzs) via eliminative nitrilimine-alkene 1,3-dipolar cycloaddition (ENAC) reaction was developed in excellent yield and high regioselectivity. Enaminones and nitrilimines generated in situ were selected as dipolarophiles and dipoles, respectively. A deep screening of the employed base, solvent, and temperature was carried out to optimize reaction conditions. Recycling tests of ionic liquid were performed, furnishing efficient performance until six cycles. Finally, a plausible mechanism of cycloaddition was proposed. Then, the effect of three different structures of Pzs was evaluated on the F_1_F_O_-ATPase activity and mitochondrial permeability transition pore (mPTP) opening. The Pz derivatives’ titration curves of **6a**, **6h**, and **6o** on the F_1_F_O_-ATPase showed a reduced activity of 86%, 35%, and 31%, respectively. Enzyme inhibition analysis depicted an uncompetitive mechanism with the typical formation of the tertiary complex enzyme-substrate-inhibitor (*ESI*). The dissociation constant of the *ESI* complex (*K*_i_’) in the presence of the **6a** had a lower order of magnitude than other Pzs. The pyrazole core might set the specific mechanism of inhibition with the F_1_F_O_-ATPase, whereas specific functional groups of Pzs might modulate the binding affinity. The mPTP opening decreased in Pz-treated mitochondria and the Pzs’ inhibitory effect on the mPTP was concentration-dependent with **6a** and **6o**. Indeed, the mPTP was more efficiently blocked with 0.1 mM **6a** than with 1 mM **6a**. On the contrary, 1 mM **6o** had stronger desensitization of mPTP formation than 0.1 mM **6o**. The F_1_F_O_-ATPase is a target of Pzs blocking mPTP formation.

## 1. Introduction

**Figure 1:**
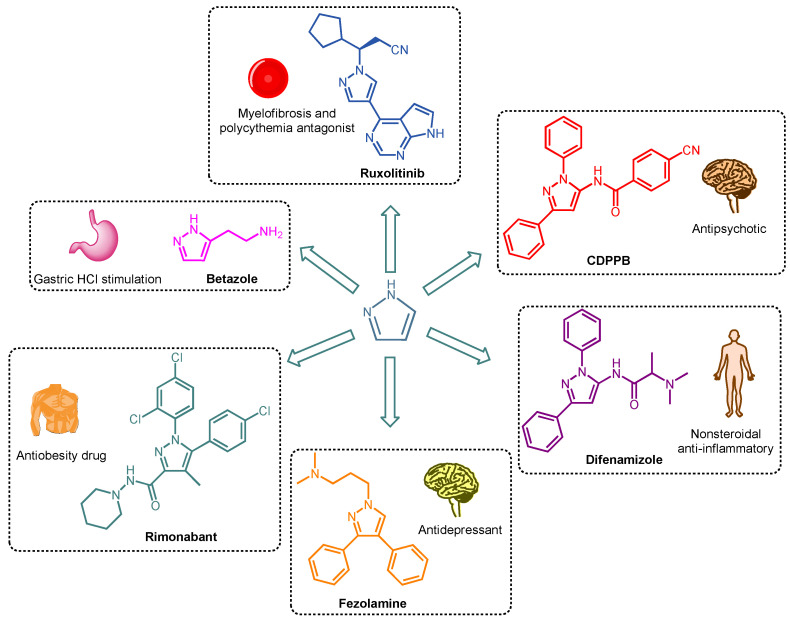
Clinical uses of drugs containing a pyrazole *core*.

Pyrazoles (Pzs) are aromatic five-membered heterocyclic compounds characterized by two adjacent nitrogen atoms and three carbons in the ring. They can exist in three tautomeric forms due to the π-electrons shifting into the heterocycle ring [1]. Pzs show a large number of applications due to the aromatic character of the heterocycle *core* that makes them resistant to oxidation, reduction, and acid or basic hydrolysis [2]. For this reason, Pz derivatives are useful as agrochemicals, such as insecticides, fungicides, and herbicides [3,4]; or as dyes [5], sunscreen materials [6], analytical reagents [7], and powerful ligands in coordination chemistry [8]. In addition, polyaromatic pyrazoles have important photophysical, optical, and electronic properties [5,9]. Pzs and their derivatives surely play a significant role in medicinal chemistry towards the development of novel and more efficient drugs, considering their active participation in hydrogen bond formation, π-stacking, and dipole–dipole interactions with different biological targets [10]. Among others, Pzs present significant antibacterial [11], antimicrobic, anticancer, anti-inflammatory, anti-diabetic, and anti-degenerative activity [12]. In Figure 1, Betazole [13], CDPPB [14], Difenamizole [15], Fezolamine [16], Rimonabant [17], and Ruxolitinib [18,19] are examples of Pz-containing drugs already available on the market (Figure 1).

**Scheme 1:**
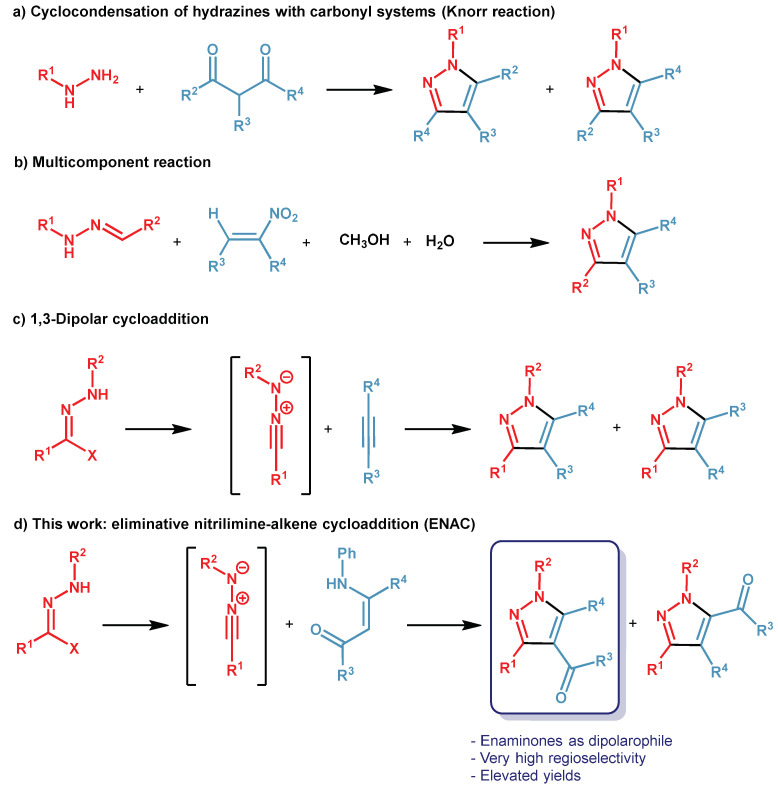
Conventional approaches and our proposal for synthesis of tetrasubstituted pyrazoles.

Especially, their potential bioactive properties have made this class of heterocycles attractive targets for organic synthesis and in particular, in the past decade, many methods to synthesize tetrasubstituted Pzs have been developed [20]. Among them, one of the first realized procedures was the cyclocondensation of substituted hydrazines with 1,3-diketones, known as the Knorr reaction that, however, turned out to be a low-performant reaction for the obtaining of a regioisomer mixture (Scheme 1a) [21]. An approach that surely solved the problem of regioselectivity was the multicomponent synthesis illustrated in Scheme 1b [22], but the method has limited applications due to the difficult preparation of reagents or use of closed reactors [23]. The 1,3-dipolar cycloaddition reaction between alkynes and nitrilimines generated in situ from hydrazonyl halides (Scheme 1c) provided tetrasubstituted Pzs in good yields without, however, the overcoming of very low regioselectivity [24,25].

With the aim to develop a novel and higher-performing synthesis of 1,3,4,5-tetrasubstituted Pzs, and in continuity with our experience in the construction of biologically active heterocycle compounds [26,27,28,29], especially by eliminative 1,3-dipolar cycloadditions [30,31], herein, we present our highly regioselective method founded on a base-promoted eliminative nitrilimine-alkene cycloaddition (ENAC). The reactions, performed between various enaminones and several nitrilimines generated in situ by hydrazonyl chlorides in ionic liquid as an eco-friendly solvent (Scheme 1d) are, to our knowledge, the first attempt of ENAC with enaminones as a dipolarophile.

In general, in 1,3-dipolar cycloadditions, olefins are a valid alternative to alkynes because of their easy availability and low-cost preparation [32]. In detail, the ENAC reaction proceeds through the formation of an unstable intermediate pyrazoline, which is easily transformed into the corresponding stable Pz by an eliminative step, as reported in several works of literature [33,34,35,36,37,38,39]. Moreover, enaminones are excellent and versatile dipolarophiles to promote the quick formation of *N*-containing heterocycles by eliminative cycloaddition [40] because they can easily lose a primary amine [41].

In this work, after an in-depth examination of the best reaction conditions by varying base, solvent, and temperature, we extended the procedure to several enaminones and nitrilimines, obtaining a series of 1,3,4,5-tetrasubstituted Pzs in very high yield and regioselective manner. We selected the [mPy]OTf as a non-conventional solvent by considering the ionic liquid typical properties (i.e., low vapor pressure, solvent recover/recycle, and so on) [42], the acclaimed strong stabilization of reaction intermediates [43], and its easy one-step preparation through a halide-free direct procedure [44]. Hence, recovery procedures to reuse the [mPy]OTf were conducted, observing an elevated performance until six cycles.

Finally, we proposed a probable mechanism of base-promoted eliminative nitrilimine-alkene cycloaddition, starting from enaminones and nitrilimines. Furthermore, we evaluated the effect of new Pzs on mitochondrial F_1_F_O_-ATPase and the mitochondrial permeability transition pore (mPTP) event, considering analogous previous studies conducted on triazole substrates. The F_1_F_O_-ATPase is evolutionarily specialized in ATP synthesis by using the protonmotive force across the inner mitochondrial membrane, and it is also the main candidate responsible for the mPTP formation [45,46,47]. The F_1_F_O_-ATPase as a bifunctional membrane-bound molecular machine transduces the energy of the transmembrane proton motive force (Δ*p*) to the chemical energy in the form of ATP via torque rotation. Vice versa, working as an H^+^ pump reenergizes the inner mitochondrial membranes (IMM) building the Δ*p* [48]. The drop of Δ*p* increases mitochondrial ROS production and Ca^2+^ accumulation. These are molecular events that open the mPTP [49,50]. In several diseases, the mPTP is involved in triggering different forms of regulated cell death [51,52], and the F_1_F_O_-ATPase is considered the outstanding biological target in drug discovery to block this phenomenon [53,54,55,56]. 

## 2. Materials and Methods

Commercial starting materials were purchased from Merck (Milano, Italy) or Alfa Aesar (Karlsruhe, Germany) and were used without further purification. Reactions were monitored by TLC using silica plates 60-F264, commercially available from Merck (Milano, Italy). Mono and bidimensional ^1^H and ^13^C NMR experiments were recorded at 300, 500, and 125.7 MHz, respectively, in CDCl_3_ and DMSO-d_6_ as solvent using tetramethylsilane (TMS) as an internal standard (Bruker ACP 300 MHz and Bruker Avance 500 MHz with a 5 mm TBO probe, Rheinstetten, Germany). Chemical shifts are given in parts per million and coupling constants in Hertz. Regiochemistry was established by the NMR technique. High-resolution mass spectra (HRMS) were recorded with a Bruker Compact QTOF instrument (Bruker, Billerica, MA, USA). HRMS spectra were acquired in positive ion mode, with a mass resolution of 30,000. Mass calibration was performed with a solution of sodium formate clusters and processed in HPC mode. Spectra acquisition was performed in flow injection, with a full scan mode in the range of 50 to 500 m/z. N_2_ was the source of dry gas (V = 4 L/min, T = 180 °C). The ion formula of each compound was calculated with the Smart Formula tool of the Bruker software platform, analyzing the isotopic pattern ratio with 4 mDa mass confidence. All samples were dissolved in MeOH. The final substrates were further purified before biological assays by recrystallization. Synthesis and characterization of variously substituted benzoyl phenylhydrazines **3a**–**3g** and hydrazonyl chlorides **4a**–**4g** are realized by modified literature procedures [57,58], respectively] and reported in the Supplementary Materials. Enaminones **5a**–**5c** were prepared according to procedures in the literature [59,60]. [mPy]OTf was prepared according to procedures in the literature [44] (see the Supplementary Materials for the synthetic procedure). 

### 2.1. General Procedure for Synthesis of 1,3,4,5-Tetrasubstituted Pyrazoles ***6a***–***6g***

In a 50 mL two-necked round-bottom flask, equipped with a bubble condenser and magnetic stir bar, enaminone **5a**–**5c** (0.5 g, 1 eq), anhydrous K_2_CO_3_ (2.5 eq), and hydrazonyl chloride **4a**–**4g** (1.3 eq) were mixed with 12 mL of [*m*Py](OTf)/H_2_O (9:1 v:v). The reaction was heated at 50 °C for the appropriate time. The mixture was extracted with dichloromethane (3 × 10 mL) and the combined organic layer was washed with a saturated aqueous NH_4_Cl solution (3 × 10 mL), dried with anhydrous Na_2_SO_4_, filtered, and evaporated under vacuum. The crude was purified by flash chromatography (hexane/ethyl acetate 8:2 v:v), isolating product **6a**–**6g**.

The regiochemistry was attributed by comparison with data in the literature for compound **6a** [61]. Furthermore, the regioisomeric ratio was calculated by ^1^H NMR of the crude of reaction, by comparison of the CH_3_ singlet on the pyrazole ring for both regioisomers (see the Supplementary Materials).

### 2.2. Recovery and Recycling Procedure of Ionic Liquid [mPy](OTf)

The [mPy](OTf)/H_2_O mixture, recovered as residue insoluble in the extraction phase, was washed with diethyl ether (2 × 5 mL) and dried at 70 °C under vacuum conditions. Successive runs were performed in the recycled ionic liquid after the addition of water and fresh reagents. 

**1-(5-Methyl-1,3-diphenyl-1*H*-pyrazol-4-yl)ethanone (6a).** White solid. ^1^H NMR (CDCl_3_, 300 MHz): δ (ppm) 2.13 (s, 3H, CH_3_), 2.56 (s, 3H, CH_3_), 7.41–7.48 (m, 4H, Ar), 7.48–7.50 (m, 3H, Ar), 7.50–7.53 (m, 1H, Ar), 7.53–7.59 (m, 2H, Ar). ^13^C NMR (CDCl_3_, 125 MHz): δ (ppm) 12.82, 30.83, 120.56, 125.76, 128.41, 128.70, 128.73, 129.25, 129.44, 133.71, 138.56, 143.70, 153.32, 195.94. ESI(+)-MS: *m*/*z* [M + H]^+^ calcd for [C_18_H_17_N_2_O]^+^, 277.1335, found 277.1331. 

**1-(3-(4-Methoxyphenyl)-5-methyl-1-phenyl-1*H*-pyrazol-4-yl)ethanone (6b).** White solid. ^1^H NMR (CDCl_3_, 300 MHz): δ (ppm) 2.15 (s, 3H, CH_3_), 2.55 (s, 3H, CH_3_), 3.85 (s, 3H, CH_3_),6.95–7.01 (m, 2H, Ar), 7.40–7.55 (m, 7H, Ar). ^13^C NMR (CDCl_3_, 125 MHz): δ (ppm) 12.78, 30.73, 55.28, 113.83, 120.49, 125.72, 125.95, 128.61, 129.19, 130.61, 138.59, 143.57, 153.04, 160.02, 196.01. ESI(+)-MS: *m*/*z* [M + H]^+^ calcd for [C_19_H_19_N_2_O_2_]^+^, 307.1441, found 307.1436. 

**1-(5-Methyl-1-phenyl-3-(*p*-tolyl)-1*H*-pyrazol-4-yl)ethanone (6c).** Pale-yellow solid. ^1^H NMR (CDCl_3_, 300 MHz): δ (ppm) 2.14 (s, 3H, CH_3_), 2.41 (s, 3H, CH_3_), 2.55 (s, 3H, CH_3_),7.22–7.29 (m, 2H, Ar), 7.39–7.54 (m, 7H, Ar). ^13^C NMR (CDCl_3_, 125 MHz): δ (ppm) 12.75, 21.29, 30.78, 120.53, 125.72, 128.61, 129.05, 129.18, 129.25, 130.68, 138.50, 138.59, 143.55, 153.32, 196.05. ESI(+)-MS: *m*/*z* [M + H]^+^ calcd for [C_19_H_19_N_2_O]^+^, 291.1492, found 291.1492.

**1-(5-Methyl-3-(4-nitrophenyl)-1-phenyl-1*H*-pyrazol-4-yl)ethanone (6d).** Yellow solid. ^1^H NMR (CDCl_3_, 300 MHz): δ (ppm) 2.24 (s, 3H, CH_3_), 2.57 (s, 3H, CH_3_), 7.44–7.60 (m, 5H, Ar), 7.74–7.82 (m, 2H, Ar), 8.28–8.35 (m, 2H, Ar). ^13^C NMR (CDCl_3_, 125 MHz): δ (ppm) 12.89, 31.03, 120.82, 123.58, 125.78, 129.20, 129.46, 130.30, 138.27, 140.21, 143.97, 147.96, 150.78, 194.76. ESI(+)-MS: *m*/*z* [M + H]^+^ calcd for [C_18_H_16_N_3_O_3_]^+^, 322.1186, found 322.1186. 

**1-(3-(2-Chlorophenyl)-5-methyl-1-phenyl-1*H*-pyrazol-4-yl)ethanone (6e).** Yellow solid. ^1^H NMR (CDCl_3_, 300 MHz): δ (ppm) 2.06 (s, 3H, CH_3_), 2.62 (s, 3H, CH_3_), 7.31–7.41 (m, 2H, Ar), 7.42–7.54 (m, 7H, Ar). ^13^C NMR (CDCl_3_, 125 MHz): δ (ppm) 13.01, 29.61, 120.83, 125.70, 126.87, 128.73, 129.20, 129.65, 130.23, 131.64, 133.42, 134.30, 138.40, 143.79, 150.55, 194.70. ESI(+)-MS: *m*/*z* [M + H]^+^ calcd for [C_18_H_16_ClN_2_O]^+^, 311.0946, found 311.0941.

**1-(3-(3-Chlorophenyl)-5-methyl-1-phenyl-1*H*-pyrazol-4-yl)ethanone (6f).** Yellow solid. ^1^H NMR (CDCl_3_, 300 MHz): δ (ppm) 2.17 (s, 3H, CH_3_), 2.55 (s, 3H, CH_3_), 7.34–7.43 (m, 3H, Ar), 7.44–7.56 (m, 5H, Ar), 7.57–7.61 (m, 1H, Ar). ^13^C NMR (CDCl_3_, 125 MHz): δ (ppm) 12.78, 30.87, 120.52, 125.72, 127.70, 128.80, 128.87, 129.30, 129.42, 129.59, 134.36, 135.45, 138.40, 143.83, 151.75, 195.34. ESI(+)-MS: *m*/*z* [M + H]^+^ calcd for [C_18_H_16_ClN_2_O]^+^, 311.0946, found 311.0952.

**1-(3-(4-Chlorophenyl)-5-methyl-1-phenyl-1*H*-pyrazol-4-yl)ethanone (6g).** Yellow solid. ^1^H-NMR (CDCl_3_, 300 MHz): δ (ppm) 2.16 (s, 3H, CH_3_), 2.55 (s, 3H, CH_3_), 7.37–7.43 (m, 1H, Ar), 7.43–7.46 (m, 2H, Ar), 7.47–7.51 (m, 4H, Ar), 7.51–7.57 (m, 2H, Ar). ^13^C-NMR (CDCl_3_, 125 MHz): δ (ppm) 12.79, 30.86, 120.54, 125.72, 128.62, 128.84, 129.29, 130.71, 132.15, 134.82, 138.43, 143.79, 151.98, 195.41. ESI(+)-MS: *m*/*z* [M + H]^+^ calcd for [C_18_H_16_ClN_2_O]^+^, 311.0946, found 311.0948.

**(5-Methyl-1,3-diphenyl-1*H*-pyrazol-4-yl)(phenyl)methanone (6h).** White solid. ^1^H-NMR (CDCl_3_, 300 MHz): δ (ppm) 2.45 (s, 3H, CH_3_), 7.11–7.19 (m, 3H, Ar), 7.19–7.32 (m, 3H, Ar), 7.36–7.46 (m, 3H, Ar), 7.50–7.61 (m, 4H, Ar), 7.69–7.77 (m, 2H, Ar). ^13^C-NMR (CDCl_3_, 125 MHz): δ (ppm) 12.26, 118.86, 125.61, 127.92, 128.00, 128.04, 128.61, 128.70, 129.31, 129.77, 132.45, 132.53, 138.43, 138.92, 143.33, 152.52, 192.79. ESI(+)-MS: *m*/*z* [M + H]^+^ calcd for [C_23_H_19_N_2_O]^+^, 339.1492, found 339.1492.

**(3-(4-Methoxyphenyl)-5-methyl-1-phenyl-1*H*-pyrazol-4-yl)(phenyl)methanone (6i).** White solid. ^1^H-NMR (CDCl_3_, 300 MHz): δ (ppm) 2.42 (s, 3H, CH_3_), 3.72 (s, 3H, CH_3_), 6.64–6.74 (m, 2H, Ar), 7.22–7.30 (m, 3H, Ar), 7.33–7.42 (m, 3H, Ar), 7.51–7.59 (m, 4H, Ar), 7.71–7.79 (m, 2H, Ar). ^13^C-NMR (CDCl_3_, 125 MHz): δ (ppm) 12.24, 55.14, 113.44, 118.58, 124.98, 125.54, 128.05, 128.47, 129.23, 129.73, 129.85, 132.50, 138.40, 138.91, 143.10, 152.15, 159.34, 192.86. ESI(+)-MS: *m*/*z* [M + H]^+^ calcd for [C_24_H_21_N_2_O_2_]^+^, 369.1598, found 369.1597.

**(5-Methyl-1-phenyl-3-(*p*-tolyl)-1*H*-pyrazol-4-yl)(phenyl)methanone (6j).** White solid. ^1^H NMR (CDCl_3_, 300 MHz): δ (ppm) 2.07 (s, 3H, CH_3_), 2.41 (s, 3H, CH_3_), 7.22–7.29 (m, 6H, Ar), 7.29–7.34 (m, 3H, Ar), 7.34–7.41 (m, 3H, Ar), 7.56–7.63 (m, 2H, Ar). ^13^C NMR (CDCl_3_, 125 MHz): δ (ppm) 21.37, 31.30, 121.88, 125.44, 127.82, 128.49, 128.81, 128.96, 129.07, 129.32, 129.56, 129.90, 130.42, 138.50, 139.15, 144.64, 152.46, 196.07. ESI(+)-MS: *m*/*z* [M + H]^+^ calcd for [C_24_H_21_N_2_O]^+^, 353.1648, found 353.1645.

**(5-Methyl-3-(4-nitrophenyl)-1-phenyl-1*H*-pyrazol-4-yl)(phenyl)methanone (6k).** Yellow solid. ^1^H NMR (CDCl_3_, 300 MHz): δ (ppm) 2.04 (s, 3H, CH_3_), 7.23–7.29 (m, 2H, Ar), 7.29–7.36 (m, 5H, Ar), 7.38–7.47 (m, 3H, Ar), 7.90–7.99 (m, 2H, Ar), 8.26–8.32 (m, 2H, Ar). ^13^C NMR (CDCl_3_, 125 MHz): δ (ppm) 31.16, 122.03, 123.36, 125.42, 128.38, 128.90, 129.01, 129.21, 129.91, 130.05, 130.32, 138.77, 139.39, 145.72, 147.77, 150.18, 195.30. ESI(+)-MS: *m*/*z* [M + H]^+^ calcd for [C_23_H_18_N_3_O_3_]^+^, 384.1343, found 384.1352.

**(3-(2-Chlorophenyl)-5-methyl-1-phenyl-1*H*-pyrazol-4-yl)(phenyl)methanone (6l).** Pale-yellow solid. ^1^H NMR (CDCl_3_, 300 MHz): δ (ppm) 2.53 (s, 3H, CH_3_), 7.6–7.15 (m, 2H, Ar), 7.16–7.22 (m, 2H, Ar), 7.27–7.42 (m, 2H, Ar), 7.43–7.50 (m, 2H, Ar), 7.50–7.61 (m, 4H, Ar), 7.61–7.68 (m, 2H, Ar). ^13^C NMR (CDCl_3_, 125 MHz): δ (ppm) 12.50, 120.10, 125.58, 126.46, 127.57, 128.68, 129.29, 129.35, 129.43, 129.50, 131.79, 131.98, 132.33, 133.44, 138.51, 138.74, 143.37, 150.60, 192.16. ESI(+)-MS: *m*/*z* [M + H]^+^ calcd for [C_23_H_18_ClN_2_O]^+^, 373.1102, found 373.1110.

**(3-(3-Chlorophenyl)-5-methyl-1-phenyl-1*H*-pyrazol-4-yl)(phenyl)methanone (6m).** Pale-yellow solid. ^1^H NMR (CDCl_3_, 300 MHz): δ (ppm) 2.44 (s, 3H, CH_3_), 7.01–7.10 (m, 1H, Ar), 7.10–7.16 (m, 1H, Ar), 7.20–7.31 (m, 4H, Ar), 7.36–7.43 (m, 1H, Ar), 7.48–7.52 (m, 1H, Ar), 7.52–7.58 (m, 4H, Ar), 7.68–7.75 (m, 2H, Ar). ^13^C NMR (CDCl_3_, 125 MHz): δ (ppm) 12.28, 118.90, 125.58, 126.95, 127.98, 128.19, 128.57, 128.80, 129.17, 129.38, 129.62, 132.72, 133.96, 134.22, 138.41, 138.72, 143.56, 151.09, 192.49. ESI(+)-MS: *m*/*z* [M + H]^+^ calcd for [C_23_H_18_ClN_2_O]^+^, 373.1102, found 373.1117.

**(3-(4-Chlorophenyl)-5-methyl-1-phenyl-1*H*-pyrazol-4-yl)(phenyl)methanone (6n).** Pale-yellow solid. ^1^H NMR (CDCl_3_, 300 MHz): δ (ppm) 2.41 (s, 3H, CH_3_), 7.10–7.17 (m, 2H, Ar), 7.27–7.32 (m, 2H, Ar), 7.35–7.40 (m, 2H, Ar), 7.42–7.48 (m, 2H, Ar), 7.49–7.58 (m, 4H, Ar), 7.68–7.76 (m, 2H, Ar). ^13^C NMR (CDCl_3_, 125 MHz): δ (ppm) 12.29, 125.58, 128.23, 128.48, 128.76, 129.37, 129.72, 129.86, 130.36, 131.00, 132.81, 133.94, 138.33, 138.78, 143.41, 151.27, 192.57. ESI(+)-MS: *m*/*z* [M + H]^+^ calcd for [C_23_H_18_ClN_2_O]^+^, 373.1102, found 373.1114.

**Phenyl(1,3,5-triphenyl-1*H*-pyrazol-4-yl)methanone (6o).** White solid. ^1^H NMR (CDCl_3_, 300 MHz): δ (ppm) 7.08–7.21 (m, 2H, Ar), 7.30–7.40 (m, 4H, Ar), 7.42–7.58 (m, 6H, Ar), 7.61–7.69 (m, 3H, Ar), 7.82–7.90 (m, 3H, Ar), 7.91–8.03 (m, 2H, Ar). ^13^C NMR (CDCl_3_, 125 MHz): δ (ppm) 120.24, 123.21, 124.57, 127.03, 128.30, 128.37, 128.57, 128.71, 128.77, 129.09, 129.45, 129.86, 131.82, 135.00, 137.94, 139.41, 139.87, 161.54, 165.80, 189.71.ESI(+)-MS: *m*/*z* [M + H]^+^ calcd for [C_28_H_21_N_2_O]^+^, 401.1648, found 401.1647.

### 2.3. Preparation of Mitochondrial Fraction

Swine hearts were collected in a local abattoir and transported to the laboratory within 2 h on ice at 0–4 °C. After removing as much fat and blood clots as possible, approximately 30–40 g of heart tissue was rinsed in ice-cold Tris-HCl wash buffer (medium A) consisting of 0.25 M sucrose, 10 mM Tris (hydroxymethyl)-aminomethane (Tris), pH 7.4 and finely chopped into fine pieces with scissors. Subsequently, the tissues were gently dried on absorbent paper, weighed, and homogenized with Ultraturrax T25 in medium B (0.25 M of sucrose, 10 mM of Tris, 1 mM of EDTA (free acid), 0.5 mg/mL of BSA without fatty acid, pH 7.4 with HCl) in a ratio of 10 mL of medium B to 1 g of fresh tissue. The tissue was then carefully homogenized by a motorized Teflon pestle homogenizer (Braun Melsungen type 853202) at 650 rpm with 3 strokes up and down. The mitochondrial fraction was then obtained by gradual centrifugation (Sorvall RC2-B, SS34 rotor). The homogenate was centrifuged at 1000× *g* for 5 min, thus obtaining a supernatant and a pellet. The pellet was re-homogenized again under the same conditions as the first homogenization, and recentrifuged at 1000× *g* for 5 min. The supernatants collected from these two centrifugations, filtered through four cotton gauze layers, were centrifuged at 10,500× *g* for 10 min to produce the raw mitochondrial pellet. The raw pellet was resuspended in medium A and further centrifuged at 10,500× *g* for 10 min to obtain the final mitochondrial pellet. The latter was resuspended by gentle agitation using a Potter Elvejehm Teflon homogenizer in a small volume of medium A, thus obtaining a protein concentration of 30 mg/mL [62]. All steps were performed at 0–4 °C. Protein concentration was determined according to the Bradford colorimetric method using the Bio-Rad Protein Assay kit II with BSA as standard [63]. The mitochondrial preparations were then stored in liquid nitrogen.

### 2.4. Mitochondrial F-ATPase Activity Assay

After the thawing of the mitochondria from the liquid nitrogen, different mitochondrial preparations were used to evaluate F-ATPase activity. To measure the hydrolysis capacity of ATP by Mg^2+^-activated F_1_F_O_-ATPase, 1 mL of a reaction medium, consisting of 0.15 mg mitochondrial protein and 75 mM ethanolamine-HCl buffer at pH 9.0, was used in the presence of 6.0 mM Na_2_ATP and 2.0 mM MgCl_2_; while the same buffer was used to determine the activity of F_1_F_O_-ATPase activated with Ca^2+^ but at pH 8.8 in the presence of 3.0 mM Na_2_ATP and 2.0 mM CaCl_2_. The test involves a 5 min pre-incubation at 37 °C with the subsequent addition of the Na_2_ATP substrate to start the reaction. After 5 min, the reaction was stopped using 1 mL of an ice-cold aqueous solution of 15% (*w*/*w*) trichloroacetic acid (TCA). At this point, the samples were centrifuged for 15 min at 3500 rpm (Eppendorf Centrifuge 5202). The indirect determination of the F-ATPase activity was defined spectrophotometrically [64] by calculating the concentration of inorganic phosphate (P_i_) hydrolyzed by known quantities of mitochondrial protein present in the supernatant. Therefore, before the start of the reaction, 1.0 µL of 3.0 mg/mL of oligomycin was added to the mixture, used in F-ATPase tests as it represents a specific inhibitor of F-ATPase capable of selectively blocking the F_O_ subunit, solubilized in dimethyl sulfoxide (DMSO). For each series of experiments, at the same time as the conditions being tested, the total ATPase activity was calculated by evaluating the P_i_ in control tubes containing 1.0 μL of DMSO per mL of the reaction system. In the experiments we conducted, a 3.0 µg/mL dose of oligomycin gave the greatest inhibition of F-ATPase [65]. In each experiment, F_1_F_O_-ATPase activity was obtained as the difference between hydrolyzed P_i_ in the presence of oligomycin and hydrolyzed P_i_ by total ATPase activity and expressed as µmol P_i_ · mg protein^−1^ · min^−1^. The concentration of P_i_ hydrolyzed by known amounts of mitochondrial protein, which is an indirect measure of ATPase activity was evaluated spectrophotometrically according to Fiske and Subbarow [66].

### 2.5. Kinetic Analysis

To calculate the values of IC_50_, i.e., the concentration of the inhibitor that causes half of the maximal inhibition of enzyme activity, the data on the enzymatic activity obtained in the absence of pyrazoles and the presence of increasing concentrations of pyrazoles were used to calculate the inhibition of the enzyme which, after background correction, have been adapted to a 3 parameter Equation (1), where the lower data limit (no enzyme inhibition) is 0. In Equation (1), the enzyme activity (*y*) is a function of the inhibitor concentration (*x*), “*Range*” is the uninhibited enzyme activity (in the absence of the inhibitor) and s is a slope factor. Since *x* is in the denominator, *y* falls at increasing *x* values.

(1) $$y=\frac{Range}{1+{\left(\frac{x}{\mathrm{IC}50}\right)}^{s}}$$

The study of pyrazole inhibition mechanisms on Ca^2+^- or Mg^2+^-activated F_1_F_O_-ATPases was performed using Dixon and Cornish-Bowden diagrams [67]. Several experimental sets were designed in which the activity of F-ATPase was evaluated in the presence of increasing concentrations of pyrazoles at two concentrations of ATP, maintaining a constant concentration of the cofactor Mg^2+^ or Ca^2+^. The reciprocal of enzymatic activity, 1/v in the Dixon diagram or the s/v ratio in the Cornish-Bowden diagram, was plotted as a function of pyrazole concentration. In all plots, the specific activity of the enzyme was taken as the expression of v. The *K*’_i_ values, which represent the dissociation constant of the ternary enzyme-substrate-inhibitor complex (*ESI*), were calculated as the abscissa (changed to positive) of the intercept of the lines obtained in the Cornish-Bowden graphs.

### 2.6. mPTP Assay

On freshly isolated mitochondrial fractions from swine heart, fresh mitochondrial suspensions (1 mg/mL) were energized in the assay buffer (130 mM KCl, 1 mM KH_2_PO_4_, 20 mM HEPES, pH 7.2 with TRIS), incubated at 37 °C with 1 μg/mL of rotenone and 5 mM of succinate. Selected doses of pyrazoles were added to the mitochondrial suspensions after the evaluation of the mPTP. The opening of mPTP was induced by the addition of Ca^2+^ 10 μM as a CaCl_2_ solution at fixed time intervals (1 min). The calcium retention capacity (CRC), the lowering of which indicates the opening of mPTP, was evaluated spectro-fluorophotometrically in the presence of 0.8 μM of Fura-FF. The probe has different spectral properties in the absence and presence of Ca^2+^; that is, it shows an excitation/emission spectrum of 365/514 nm in the absence of Ca^2+^ (Fura-FF low Ca^2+^) and shifts to 339/507 nm in the presence of high concentrations of Ca^2+^ (Fura-FF high Ca^2+^). An increase in the fluorescence intensity ratio (Fura-FF high Ca^2+^)/(Fura-FF low Ca^2+^), i.e., a decrease in CRC [68], indicates the opening of mPTP. All measurements were processed by LabSolutions RF software.

### 2.7. Statistical Analysis

The data represent the mean ± SD (shown as vertical bars in the figures) of the number of experiments reported in the figure captions. In each set of biochemical experiments, the analyses were carried out on at least three distinct mitochondrial preparations. The differences between the enzyme activity data in differently treated mitochondria were evaluated by one-way ANOVA followed by Dunnett’s test when the *F* values indicated a significance (*P* ≤ 0.05).

## 3. Results and Discussion

### 3.1. Synthesis

**Scheme 2:**
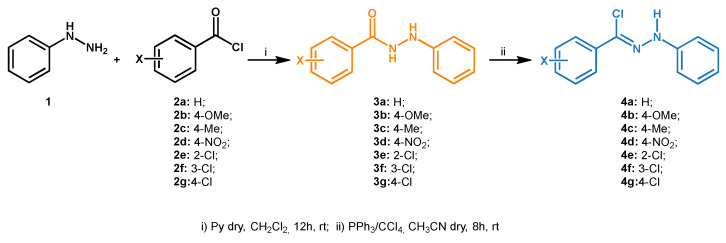
Synthesis of hydrazonyl chlorides **4a**–**4g**.

With the purpose of developing a highly regioselective synthesis of 1,3,4,5-tetrasubstituted pyrazoles by 1,3-dipolar cycloaddition reaction, a preventive preparation of dipoles and dipolarophiles was necessary. Firstly, hydrazonyl chlorides **4a**–**4g,** a source of nitrilimines as 1,3-dipole, were synthesized from phenylhydrazine **1** and commercially available acyl chlorides **2a**–**2g**, as described in Scheme 2.

In detail, intermediate aroyl phenylhydrazines **3a**–**3g** were obtained in very good yields and immediately transformed in the correspondent hydrazonyl chlorides **4a**–**4g**, isolated in excellent yields (see the Supplementary Materials). In addition, three enaminones **5a**–**5c** were prepared as dipolarophiles through procedures in the literature [59,60].

With the precursors in our hand, initial attempts to optimize the ENAC reaction were performed by choosing hydrazonyl chloride **4a** and enaminone **5a** as starting materials. The changes in solvent, base, and temperature are summarized in Table 1. 

Initially, the reaction was tested in [mPy]OTf/H_2_O 9:1 *v*/*v* at room temperature with Et_3_N as the base, observing a low yield, also after the extension of the reaction time to 24 h (Table 1, entries 1 and 2). A temperature rise to 50 °C allowed an increase in yield in just 2 h (Table 1, entry 3). Unfortunately, the prolonging of the reaction times favored the degradation of reactants. A further increase in temperature until 85 °C drastically reduced reaction yields due to the decomposition of reagents (Table 1, entry 4). Subsequently, a screening of different bases was evaluated in the same reaction conditions. In more detail, DBU and DMAP were used as organic bases with results less satisfactory than triethylamine (Table 1, entries 5 and 6). Then, we decided to employ inorganic bases and we observed only traces of the final product in the presence of NaOH (Table 1, entry 7), while K_2_CO_3_ furnished 6a in high yield both at room temperature and at 50 °C (Table 1, entries 8 and 9). The slight increase of hydrazonyl chloride (1.3 eq) favored an elevated yield (90%, Table 1, entry 10). Finally, the change of ionic liquid as solvent did not show any yield improvement (Table 1, entries 11–12). In this context, a particular consideration concerns the presence of water that supports the solubility both of inorganic base and [mPy]OTf, which tends to solidify at a temperature between 25 and 60 °C. In addition, the reaction was conducted in common organic solvents (i.e., CH_2_Cl_2_, THF, AcOEt, and ACN) without observing any product (data not reported). On the contrary, the employment of DMF or DMSO furnished the final product in moderate yields and good regioisomeric ratios (Table 1, entries 13–14).

It is remarkable that the best reaction conditions (Table 1, entry 10) also led to the highest regioisomeric ratio (**6a/7a**: 97/3). 

With the optimized reaction conditions in our hand, we extended the investigation on different enaminones **5a**–**5c** and various hydrazonyl chlorides **4a**–**4g** to synthesize a series of 1,3,4,5-tetrasubstituted pyrazoles **6a**–**6o** (Table 2). 

As you can see from the data collected in Table 2, the reactivity of the enaminones changed in function in the presence of aryl or alkyl groups as substituents. Especially, enaminone **5a** was more reactive than **5b** and **5c,** probably due to steric hindrance effects that determine a longer reaction time and lower yields (Table 2, entries 8–15). On the contrary, the different nature of functional groups on the aromatic moiety of hydrazonyl chlorides **4a**–**4g** did not seem to affect the reaction trend (Table 2, entries 2–7 and 9–14).

Finally, to our delight, a very high regioselectivity was confirmed for all reactions, as reported in Table 2.

### 3.2. Reaction Mechanism

**Scheme 3:**
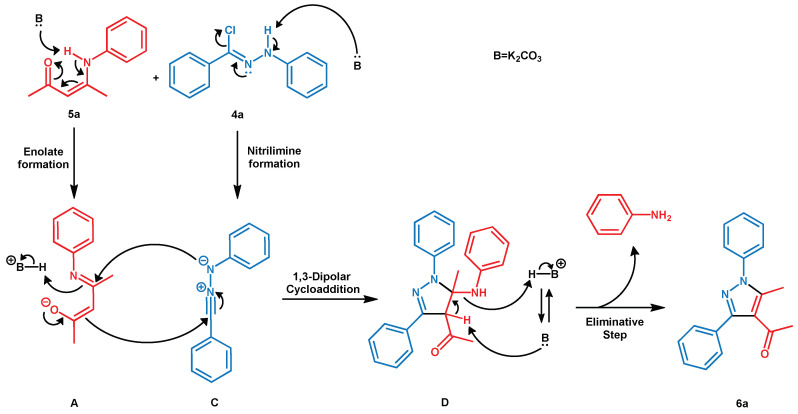
Proposed mechanism for regioselective synthesis of tetrasubstituted pyrazoles by eliminative nitrilimine-alkene 1,3-dipolar cycloaddition reaction.

At this point, we proposed a possible reaction mechanism, as illustrated in Scheme 3.

Initially, the base produces both the enolate form (**A**) of the enaminone **5a** and deprotonates the hydrazonyl chloride **4a,** generating the nitrilimine **C** in situ [69]. The latter acts as a dipole with **A** through a regioselective 1,3-dipolar cycloaddition reaction. Generally, the accepted mechanism of 1,3-dipolar cycloaddition is of a concerted nature, providing for the alignment of the dipole and the dipolarophile on two parallel planes with a highly ordered system and a low degree of entropy. Regioselectivity can be explained through the frontier molecular orbital theory (FMO) and considering the inverse reactivity of cycloaddition [70,71]. Probably, the best orbital interaction is between the HOMO of the dipolarophile and the LUMO of the nitrilimine [72]. Furthermore, when the base deprotonates the enaminone, the HOMO energy tends to increase activating the dipolarophile and improving its reactivity. Moreover, the so-called ionic self-assembly (ISA) structure of ionic liquids due to noncovalent electrostatic interactions can give clusters that act as a support of dipole and dipolarophile, favoring their alignment and improving the orbital interaction [73,74]. Then, the trapped reagents lead to the formation of a pyrazoline **D** that is immediately transformed into the corresponding pyrazole **6a** by elimination reaction with consequent loss of aniline. The heterocycle aromatization process corresponds to the fast stadium of the reaction because it was not possible to observe the pyrazoline intermedium **D**.

### 3.3. Recycling Ionic Liquid

**Figure 2:**
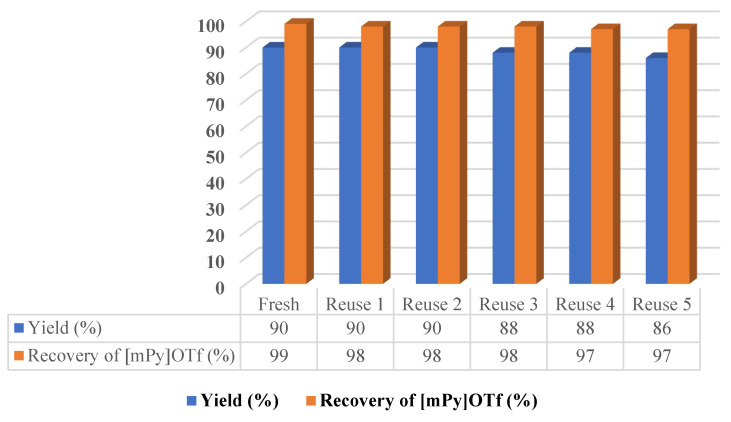
Recovery and reuse of [mPy]OTf until six cycles.

The [mPy]OTf ionic liquid has been analyzed also with respect to recovery and reuse in the reaction of enaminone **5a** with hydrazonyl chloride **4a** and the results are shown in Figure 2. 

As shown in Figure 2, similar reaction yields were obtained, showing that the ionic liquid remains active until six cycles and that it can be recovered efficiently. 

### 3.4. Pzs’ Effect on F_1_F_O_-ATPase

In previous studies, we investigated the F_1_F_O_-ATPase inhibition activity of triazole derivatives with interesting results. [75,76] Considering the nitrogen-based heterocyclic structure both of triazoles and pyrazoles, we decided to conduct a study at the mitochondrial level to evaluate whether the synthesized pyrazole compounds (Pzs) contribute to blocking the molecular events related to different forms of cell death. 

**Figure 3:**
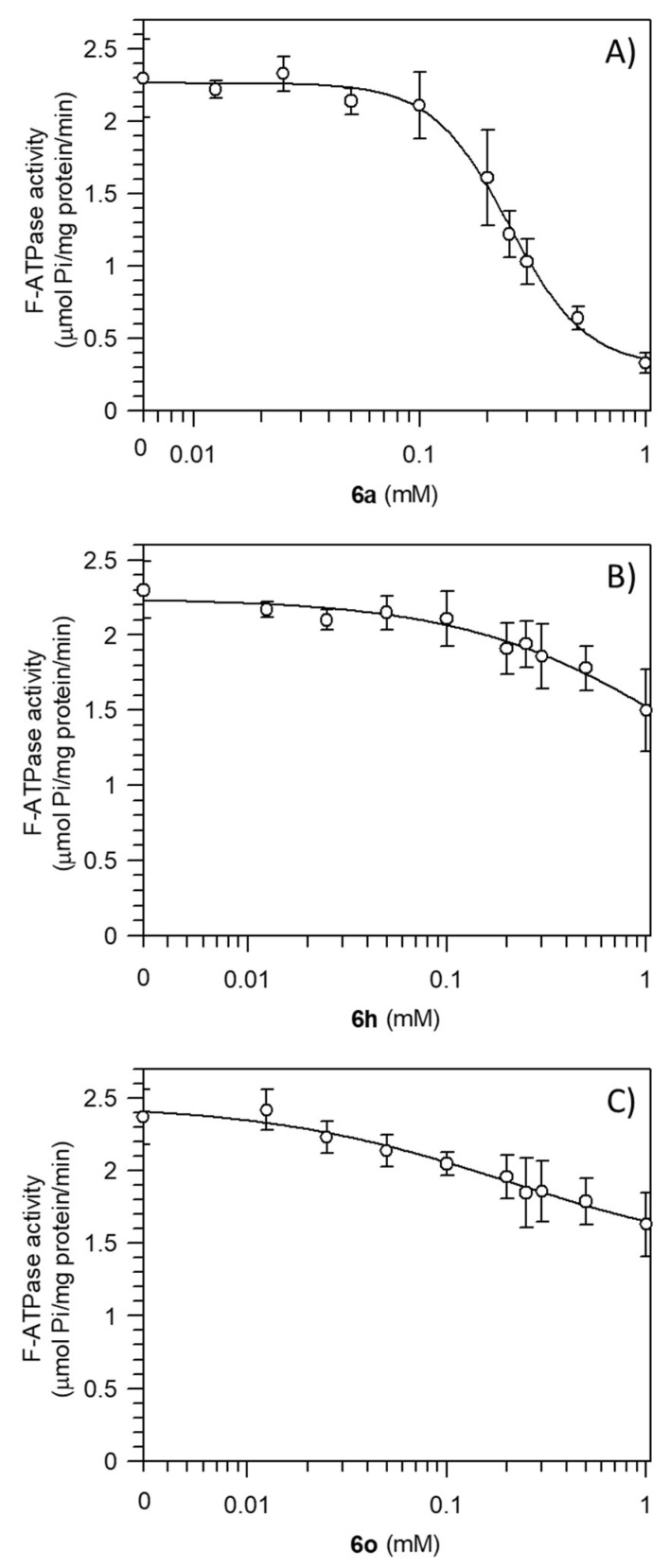
Effect of pyrazole derivatives **6a** (**A**), **6h** (**B**), and **6o** (**C**) on the activity of mitochondrial F_1_F_O_-ATPase. F-ATPase activity is plotted against concentrations of Pzs (logarithmic scale). Data represent the mean ± SD from three independent experiments carried out on different mitochondrial preparations.

To perform structure–activity relationship studies of fully-substituted pyrazoles on the molecular mechanism of F_1_F_O_-ATPase activity, uncoupled mitochondria obtained by freeze-thawing were used for kinetic analysis. We selected three pyrazole substrates (**6a**, **6h,** and **6o**) for the main difference related to the number of aryl groups linked to the azole *core*. Specifically, **6a** has a two-aryl group, **6h** has a three-aryl group, and **6o** has a four-aryl group. The Pzs’ effect, in the range of 0.01–1.0 mM, was evaluated on the F_1_F_O_-ATPase activity. The substrate-depending inhibition potency of Pzs, estimated as IC_50_ values, was calculated as 0.25 ± 0.01 mM **6a**, 1.62 ± 0.85 mM **6h**, and 0.21 ± 0.15 mM **6o** (Table 3). However, the maximal inhibition of **6a**, **6h**, and **6o** on the ATP hydrolysis by F_1_F_O_-ATPase was 87%, 79%, and 43%, respectively (Figure 3). Thus, the **6a** has the highest inhibiting power on F_1_F_O_-ATPase.

**Figure 4:**
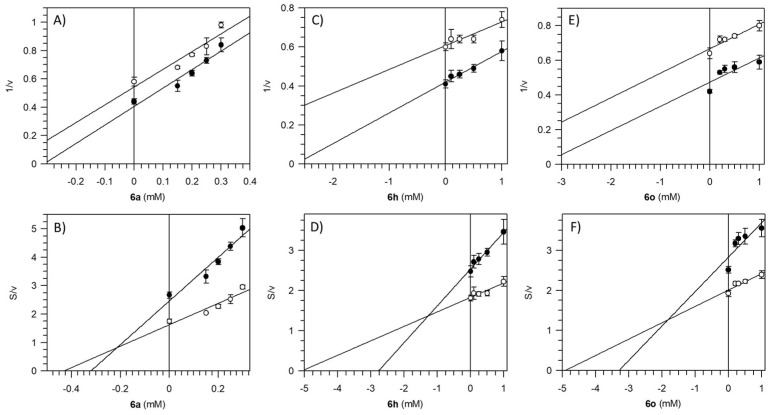
Mitochondrial F_1_F_O_-ATPase inhibition by Pzs. Dixon (**A**,**C**,**E**) and Cornish-Bowden (**B**,**D**,**E**) plots were obtained at two fixed ATP concentrations and designing the experiments as detailed in the “Kinetic analysis” section of Materials and Methods. All points represent the mean ± SD (vertical bars) of three independent experiments carried out on different mitochondrial preparations.

To understand the mechanism of inhibition by the pyrazole derivatives **6a**, **6h**, and **6o** of the F_1_F_O_-ATPase, a kinetic inhibition analysis has been set up to define the features of the enzyme-inhibitor complex formation in relation to the ATP substrate present or absence. The building of Dixon and Cornish-Bowden plots, which complement one another [67], have been performed to identify the type of inhibition of Pzs. We always obtained parallel straight lines in Dixon plots (Figure 4A,C,E), whereas in Cornish-Bowden plots **(**Figure 4B,D,F) we depicted two straight lines intersecting above the *x*-axis.

The uncompetitive mechanism of inhibition with respect to the ATP substrate was irrespective of the nature of Pzs. Therefore, Pzs can bind the F_1_F_O_-ATPase only when the enzyme-substrate (*ES*) complex was already formed, to yield the tertiary enzyme-substrate-inhibitor (*ESI*) complex. In addition, the Cornish-Bowden plots showed that the dissociation constant of the *ESI* complex (*K*’_i_) of the F_1_F_O_-ATPase inhibited with **6a** was approximately six and nine times lower than **6h** and **6o**, respectively (Table 3). The results highlighted that in the presence of **6a** the formation of the ternary complex was easier and stronger than in the presence of other Pzs.

The kinetic parameters corroborate that the **6a** has a higher inhibition efficiency on F_1_F_O_-ATPase than other Pz derivatives tested, as shown by the lower IC_50_ value for **6a** (Table 3, Entry 1). However, we can assert that the Pz core sets the specific mechanism of inhibition with the enzyme, whereas specific functional groups, in particular aryl groups in Pz derivatives, modulate the binding affinity with the F_1_F_O_-ATPase.

### 3.5. Pzs Effect on mPTP

**Figure 5:**
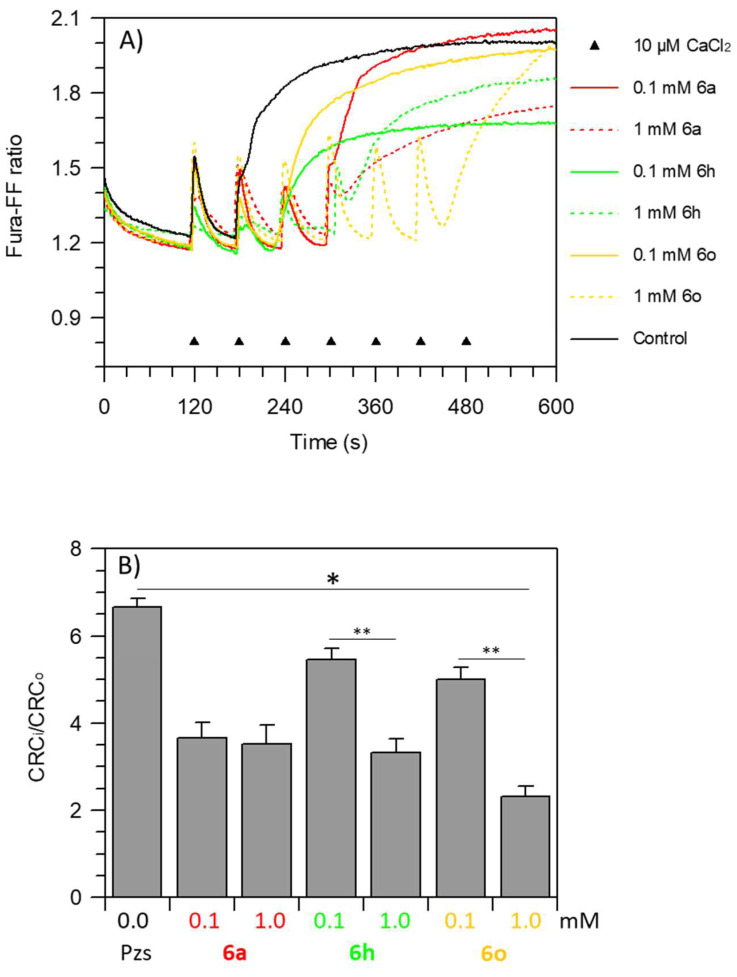
Evaluation of mPTP opening. Representative curves (**A**) of the calcium retention capacity (CRC) expressed as Fura-FF ratio, monitored in response to subsequent 10 μM CaCl_2_ pulses (shown by the triangles), as detailed in “mPTP assay” section of Materials and Methods, in untreated mitochondria (control) or in the presence of 0.1 or 1.0 mM Pzs. (**B**) Quantification of the mPTP is expressed as the ratio of the number of calcium pulses required to induce the mPTP in inhibited (CRC_i_) and treated (CRC_o_) mitochondria with different concentrations of Pzs. Data represent the mean ± SD from three independent experiments carried out on different mitochondrial preparations. * indicates significant differences with respect to the control (*p* ≤ 0.05). ** indicates significant differences (*p* ≤ 0.05) among treatments within the same Pzs.

Mitochondrial calcium retention is linked to IMM integrity. The IMM remains intact until a high channel conductance is not formed. Increasing Ca^2+^ concentration in mitochondria stimulates mPTP formation. The capability of intact mitochondria to accumulate Ca^2+^ is identified as CRC (Ca^2+^ retention capacity) and mPTP opening was measured when the Ca^2+^ pulses accumulated in the mitochondrial matrix were released. The Pzs were tested by adding 10  μM Ca^2+^ at subsequent steps of 1 min to succinate-energized freshly prepared mitochondrial suspensions. The CRC decreased in Pzs-treated mitochondria, which was revealed by an increase in fluorescence intensity, pointing out the Pzs ability to desensitize the mPTP opening (Figure 5A). In control mitochondria, the CRC decrease was revealed after 180 sec upon a two-train Ca^2+^ pulse, as shown by the rise in the Fura-FF ratio ((Fura-FF high Ca^2+^)/(Fura-FF low Ca^2+^)). Accordingly, the increase in CRC upon subsequent 10 μM Ca^2+^ additions at 1 min intervals indicated that the mPTP formation was triggered at a higher threshold value of Ca^2+^ concentration in the matrix with Pzs. In detail, the two concentrations of the Pzs tested (0.1 mM and 1 mM) had different desensitizing powers on the mPTP. In detail, **6a** had no concentration-dependent difference in mPTP inhibition. The same Ca^2+^ pulses were required to form the pore. At 0.1 mM, **6h** and **6o** had a smaller inhibitor effect on mPTP than 1 mM **6a**. Conversely, 1 mM **6h** blocked the mPTP opening as well as **6a**. Moreover, **6h** attained a higher CRC value (low Fura-FF ratio) than other Pzs on the mPTP opening with both concentrations tested. On these bases, a higher CRC value would mirror a smaller mPTP size. F_1_F_O_-ATPase and the adenine nucleotide translocator, which support the high and low ion conductance, respectively, depict the different nature of the mPTP phenomenon related to the pore size and this physiological event could be affected by **6h [77]**. Otherwise, in terms of mPTP desensitization to Ca^2+^, the effect on CRC was inversely proportional to the concentration of the **6o**. This compound showed a typical profile with a delayed rise in the Fura-FF ratio increase that indicated a CRC decreased more marked at 1 mM than at 0.1 mM (Figure 5A).

Consistently, the PTP formation extent expressed as the ratio between inhibited and uninhibited CRC (CRC_i_/CRC_o_) [78] was significantly reduced by all Pzs. Moreover, in the presence of both the **6a** concentrations tested, a half CRC_i_/CRC_o_ value was produced. On the contrary, **6h** inhibited the mPTP by 20% and 50% with 0.1 mM and 1.0 mM, respectively. A high overload of mitochondrial Ca^2+^ was required to open the mPTP with 1 mM **6o**, highlighting a more pronounced protective effect on mPTP formation compared with the other different conditions tested in the presence of other Pzs. Indeed, the CRC_i_/CRC_o_ values were decreased by 25% and 65% with 0.1 mM and 1.0 mM **6o**, respectively (Figure 5B).

## 4. Conclusions

In conclusion, we have developed a new synthetic method to obtain 1,3,4,5-tetrasubstituted pyrazoles with complete regioselectivity, starting from hydrazonyl chlorides and enaminones in a very simple reaction medium involving K_2_CO_3_ and [mPy]OTf/H_2_O 9:1 *v*/*v* at 50 °C. The reaction consists of a typical Huisgen’s 1,3-dipolar cycloaddition to form a specific pyrazoline intermedium that is rapidly transformed in correspondent pyrazole by the elimination of aniline. The experimental observations revealed the special effects exerted by the ionic liquid in the reaction, due to the particular capacity to stabilize the reaction intermediates, allowed the formation of a pyrazole *core*. The proposed process provides an easy procedure, low reaction times, complete regioselectivity with no formation of by-products, and very good versatility of final fully substituted Pzs. In addition, it is evident that the ionic liquid can be easily recovered and reused for a few cycles without losing its efficiency.

The main difference between pyrazole structures is related to the number of aryl groups linked to the azole *core*. Specifically, **6a** has a two-aryl group, **6h** has a three-aryl group, and **6o** has a four-aryl group. Pzs at the concentration of 0.1 mM were non-toxic on the F_1_F_O_-ATPase activity. Otherwise, at the high concentrations tested of 1 mM, only the **6h** and **6o** did not cause a strong percentage of inhibition of the F_1_F_O_-ATPase activity. Importantly, we have identified the F_1_F_O_-ATPase as the target of Pzs. The molecular mechanism of compounds on the enzyme arises from the core structure of Pzs and the aryl groups can lessen the inhibitory power of compounds. It is noteworthy that the data of structure–activity relationship analysis performed on the F_1_F_O_-ATPase conceive the link with the inhibition of mPTP opening. Thus, as the aryl groups increase, the inhibitory effect on F_1_F_O_-ATPase decreases, while the desensitization effect of the pore increases and the effect increases in a concentration-dependent manner with **6h** or **6o**. Moreover, the results corroborate the arcane nature of mPTP that relies on the structure and catalysis of F_1_F_O_-ATPase [79,80,81,82]. 

To sum up, future studies will aim to evaluate the time of half-life in the biological systems and stability of these molecules [83] to understand if the non-toxic action on F_1_F_O_-ATPase at low concentrations could maintain a potent mPTP inhibition.

## Figures and Tables

**Table 1:**
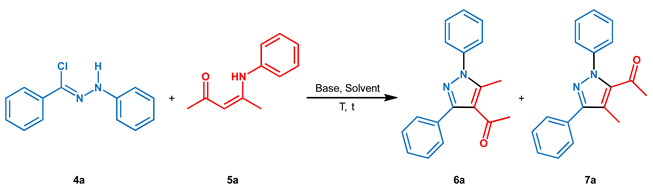
Optimization of reaction conditions.

Entry ^a^	Solvent ^b^	Base	T (°C)	t (h)	Yield (%) ^c^	6a:7a ^e^
**1**	[mPy](OTf)/H_2_O	Et_3_N	rt	2	50	90:10
**2**	[mPy](OTf)/H_2_O	Et_3_N	rt	24	51	80:20
**3**	[mPy](OTf)/H_2_O	Et_3_N	50	2	67	96:4
**4**	[mPy](OTf)/H_2_O	Et_3_N	85	2	32	95:5
**5**	[mPy](OTf)/H_2_O	DBU	50	2	42	96:4
**6**	[mPy](OTf)/H_2_O	DMAP	50	2	38	96:4
**7**	[mPy](OTf)/H_2_O	NaOH	50	2	traces	-
**8**	[mPy](OTf)/H_2_O	K_2_CO_3_	rt	2	72	96:4
**9**	[mPy](OTf)/H_2_O	K_2_CO_3_	50	2	85	96:4
**10 ^d^**	[mPy](OTf)/H_2_O	K_2_CO_3_	50	2	90	97:3
**11 ^d^**	[Bmim]Cl/H_2_O	K_2_CO_3_	50	2	75	95:5
**12 ^d^**	[Bmim][BF_4_]/H_2_O	K_2_CO_3_	50	2	78	96:4
**13 ^d^**	DMF	K_2_CO_3_	50	2	52	76:24
**14 ^d^**	DMSO	K_2_CO_3_	50	2	64	82:18

^a^ Reaction Conditions: enaminone **5a** (1 eq), base (2.5 eq), hydrazonyl chloride **4a** (1 eq) in 12 mL of solvent mixture. ^b^ IL/H_2_O 9:1 v:v; ^c^ isolated yield. ^d^ reaction conditions: enaminone **5a** (1 eq), base (2.5 eq), hydrazonyl chloride **4a** (1.3 eq) in 12 mL of solvent mixture. ^e^ regioisomeric ratio was determined by ^1^H NMR analysis of the crude reaction mixture (see Supplementary Materials).

**Table 2:**
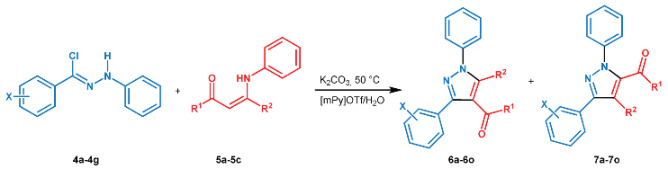
Synthesis of 1,3,4,5-tetrasubstituted pyrazoles by eliminative 1,3-dipolar cycloaddition.

Entry ^a^	Hydrazonyl Chloride	X	Enaminone	R^1^	R^2^	Product	t (h)	Yield 6a–6o (%) ^b^	6:7 ^c^
**1**	**4a**	H	**5a**	CH_3_	CH_3_	**6a**	2	90	97:3
**2**	**4b**	4-OCH_3_	**5a**	CH_3_	CH_3_	**6b**	2	95	97:3
**3**	**4c**	4-CH_3_	**5a**	CH_3_	CH_3_	**6c**	2	94	96:4
**4**	**4d**	4-NO_2_	**5a**	CH_3_	CH_3_	**6d**	2	90	95:5
**5**	**4e**	2-Cl	**5a**	CH_3_	CH_3_	**6e**	2	92	96:4
**6**	**4f**	3-Cl	**5a**	CH_3_	CH_3_	**6f**	2	90	94:6
**7**	**4g**	4-Cl	**5a**	CH_3_	CH_3_	**6g**	2	92	98:2
**8**	**4a**	H	**5b**	Ph	CH_3_	**6h**	4	81	93:7
**9**	**4b**	4-OCH_3_	**5b**	Ph	CH_3_	**6i**	4	83	96:4
**10**	**4c**	4-CH_3_	**5b**	Ph	CH_3_	**6j**	4	82	96:4
**11**	**4d**	4-NO_2_	**5b**	Ph	CH_3_	**6k**	4	80	95:5
**12**	**4e**	2-Cl	**5b**	Ph	CH_3_	**6l**	4	82	95:5
**13**	**4f**	3-Cl	**5b**	Ph	CH_3_	**6m**	4	84	96:4
**14**	**4g**	4-Cl	**5b**	Ph	CH_3_	**6n**	4	81	96:4
**15**	**4a**	H	**5c**	Ph	Ph	**6o**	4	76	94:6

^a^ Reaction conditions: enaminone **5a**–**5c** (1 eq), K_2_CO_3_ (2.5 eq), hydrazonyl chloride **4a**–**4g** (1.3 eq) in 12 mL of solvent mixture. ^b^ isolated yield. ^c^ regioisomeric ratio values were determined by ^1^H NMR analysis of the crude reaction mixture (see Supplementary Materials).

**Table 3:** Kinetic parameters for the F_1_F_O_-ATPase inhibition by Pzs. IC_50_ and *K*’_i_ values were graphically obtained from the Pzs’ titration curve and Cornish-Bowden plots of Figure 3 and Figure 4, respectively.

Entry ^a^	Compound	IC_50_ (mM)	*K*’_i_ (mM)
**1**	**6a**	0.25 ± 0.01	0.21 ± 0.03
**2**	**6h**	1.62 ± 0.85	1.27 ± 0.25
**3**	**6o**	0.21 ± 0.15	1.86 ± 0.33

^a^ Data are the mean ± SD of three independent experiments carried out on different mitochondrial preparations.

## Data Availability

Not applicable.

## Supplementary Materials

The following supporting information can be downloaded at: https://www.mdpi.com/article/10.3390/pharmaceutics15020498/s1, Scheme S1: synthesis of variously substituted benzoyl phenylhydrazines **3a**–**3g**. Scheme S2: synthesis of variously substituted hydrazonyl chlorides **4a**–**4g**. Characterization spectra of precursors and final products.

## Abbreviations

mPTP, mitochondrial permeability transition pore; Δp, proton motive force; IMM, inner mitochondrial membranes; Pzs, pyrazoles.
