# Supplementary material for: Novel Regioselective Synthesis of 1,3,4,5-Tetrasubstituted Pyrazoles and Biochemical Valuation on F1FO-ATPase and Mitochondrial Permeability Transition Pore Formation
Source: Pharmaceutics. 2023 Feb 2;15(2):498. doi: 10.3390/pharmaceutics15020498 (PMC9967880; doi:10.3390/pharmaceutics15020498)
Supplement: Supplementary file 1 [file pharmaceutics-15-00498-s001.zip › pharmaceutics-2147354-supplementary.pdf]

ELECTRONING SUPPORTING INFORMATION

Novel regioselective synthesis of 1,3,4,5-tetrasubstituted pyrazoles and biochemical valuation on F_1F_O-ATPase and mitochondrial permeability transition pore formation

Vincenzo Algieri [1,*], Cristina Algieri[2], Paola Costanzo[1], Giulia Fiorani[3], Antonio Jiritano[1], Fabrizio Olivito[1], Matteo Antonio Tallarida[1], Fabiana Trombetti[2], Loredana Maiuolo[1], Antonio De Nino[1,*] and Salvatore Nesci[2]

[1] Department of Chemistry and Chemical Technologies, University of Calabria, Via P. Bucci, Cubo 12C, 87036 Rende, CS, Italy
[2] Department of Veterinary Medical Sciences, Mitochondrial Biochemistry Lab, Via Tolara di Sopra, 50, 40064 Ozzano Emilia, BO, Italy
[3] Department Molecular Sciences and Nanosystems, University Ca' Foscari Venezia, Via Torino 155, 30172 Venezia Mestre, VE, Italy
* Correspondence: Correspondence: vincenzo.algieri@unical.it (V.A.); denino@unical.it (A.D.N.)

General procedure for synthesis of benzoyl phenylhydrazines.......................................S1

General procedure for synthesis of hydrazonyl chlorides………………………………..S3

General procedure for synthesis of [mPy]OTf……………………………………………S5

Characterization spectra of benzoyl phenylhydrazines…………………………………..S6

Characterization spectra of hydrazonyl chlorides………………………………………S20

Characterization spectra of 1,3,4,5-tetrasubstituted pyrazoles…………………………S34

^{1}H NMR spectra of crude reaction mixtures illustrated in Table 1…………………...S93

^{1}H NMR spectra of crude reaction mixtures illustrated in Table 2…………………..S104

General procedure for synthesis of benzoyl phenylhydrazines

In according to the reaction described in **Scheme S1,** benzoyl phenylhydrazines were synthesized through a modified procedure reported in literature [1].

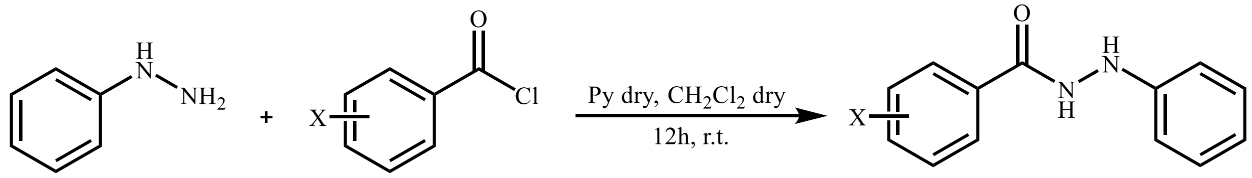

1 **2a-2g** **3a-3g**

Entry	X	Reagent	Product
1	H	**2a**	**3a**
2	4-OCH_3	**2b**	**3b**
3	4-CH_3	**2c**	**3c**
4	4-NO_2	**2d**	**3d**
5	2-Cl	**2e**	**3e**
6	3-Cl	**2f**	**3f**
7	4-Cl	**2g**	**3g**

Scheme S1. *Synthesis of variously substituted benzoyl phenylhydrazines* ***3a-3g****.*

In a 500 ml three-necked round-bottomed flask, equipped with dropping funnel and magnetic stir bar, freshly distilled phenylhydrazine **1** (16.485 g, 0.1524 mol, 1 eq) was dissolved under nitrogen in dry CH_2Cl_2 (100 ml) and fresh distilled dry pyridine (1.1 eq) was added dropwise at 0 °C for 30 min under vigorously stirring. Subsequently, temperature is kept at 0 °C and a solution of benzoyl chloride **2a-2g** (1.1 eq) in dry CH_2Cl_2 (60 ml) was added dropwise for 3h. The reaction was gently warmed to room temperature and stirred for 16h. The mixture was extracted with CH_2Cl_2 (3 x 100 ml) after addition of water (250 ml) and the combined organic layers were further washed with brine (3 x 30 ml), dried over anhydrous Na_2SO_4 and the solvent was removed under reduced pressure. The crude solid was then purified by recrystallization with EtOH.

***N*'-phenylbenzohydrazide (3a).** White solid. Yield 88%.

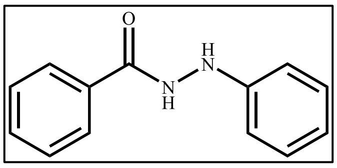

^{1}H NMR (DMSO-d_6, 300 MHz): δ (ppm) 6.67-6.76 (m, 1H, Ar), 6.76-6.88 (m, 2H, Ar), 7.10-7.22 (m, 2H, Ar), 7.43-7.64 (m, 3H, Ar), 7.84-8.06 (m, 3H, Ar and NH), 10.39 (s, 1H, NH).

^{13}C NMR (DMSO-d_6, 125 MHz): δ (ppm) 113.12, 119.56, 127.97, 129.22, 129.47, 132.41, 133.59, 150.06, 167.16.

4-methoxy-*N*'-phenylbenzohydrazide (3b). White solid. Yield 80%.

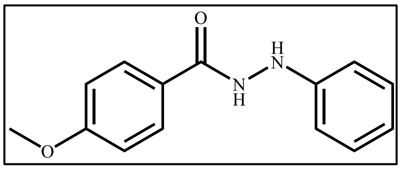

^{1}H NMR (DMSO-d6, 300 MHz): δ (ppm) 3.82 (s, 3H, CH_3), 6.66-6.74 (m, 1H, Ar), 6.74-6.82 (m, 2H, Ar), 6.97-7.08 (m, 2H, Ar), 7.08-7.21 (m, 2H, Ar), 7.86 (d, J= 2.74Hz, 1H, NH), 7.88-7.97 (m, 2H, Ar), 10.24 (d, J= 2.74Hz, 1H, NH).

^{13}C NMR (DMSO-d6, 125 MHz): δ (ppm) 55.88, 112.82, 114.20, 119.06, 125.65, 129.21, 129.65, 150.20, 162.39, 166.34.

4-methyl-*N*'-phenylbenzohydrazide (3c). White solid. Yield 85%.

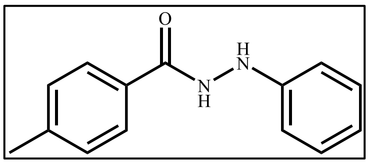

^{1}H NMR (DMSO-d6, 300 MHz): δ (ppm) 2.37 (s, 3H, CH_3), 6.67-6.75 (m, 1H, Ar), 6.75-6.84 (m, 2H, Ar), 7.10-7.20 (m, 2H, Ar), 7.24-7.37 (m, 2H, Ar), 7.77-7.86 (m, 2H, Ar), 7.88 (d, J= 2.30Hz, 1H, NH), 10.30 (d, J= 2.30Hz, 1H, NH).

^{13}C NMR (DMSO-d6, 125 MHz): δ (ppm) 21.78, 113.17, 119.61, 128.09, 129.58, 129.86, 130.87, 142.58, 150.25, 167.35.

4-nitro-*N*'-phenylbenzohydrazide (3d). Orange solid. Yield 85%.

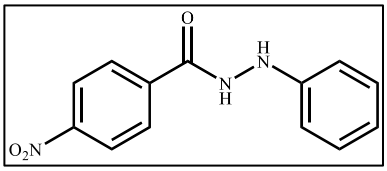

^{1}H NMR (DMSO-d6, 300 MHz): δ (ppm) 6.67-6.78 (m, 1H, Ar), 6.78-6.87 (m, 2H, Ar), 7.10-7.24 (m, 2H, Ar), 8.05 (bs, 1H, NH), 8.11-8.21 (m, 2H, Ar), 8.28-8.43 (m, 2H, Ar), 10.70 (s, 1H, NH).

^{13}C NMR (DMSO-d6, 125 MHz): δ (ppm) 112.88, 119.37, 124.16, 129.29, 129.35, 139.20, 149.57, 149.77, 165.30.

2-chloro-*N*'-phenylbenzohydrazide (3e). White solid. Yield 86%.

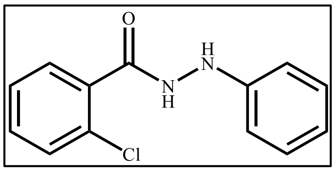

^{1}H NMR (DMSO-d6, 300 MHz): δ (ppm) 3.65 (bs, 1H, NH), 6.67-6.79 (m, 1H, Ar), 6.81-6.91 (m, 2H, Ar), 7.13-7.24 (m, 2H, Ar), 7.39-7.61 (m, 4H, Ar), 10.22 (s, 1H, NH).

^{13}C NMR (DMSO-d6, 125 MHz): δ (ppm) 112.82, 119.17, 127.75, 129.24, 129.68, 130.26, 130.75, 131.75, 135.76, 149.58, 166.77.

3-chloro-*N*'-phenylbenzohydrazide (3f). White solid. Yield 88%.

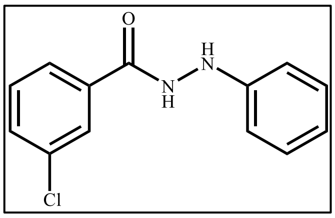

^{1}H NMR (DMSO-d6, 300 MHz): δ (ppm) 6.65-6.77 (m, 1H, Ar), 6.77-6.88 (m, 2H, Ar), 7.09-7.24 (m, 2H, Ar), 7.48-7.61 (m, 1H, Ar), 7.61-7.73 (m, 1H, Ar), 7.83-7.95 (m, 1H, Ar), 7.95-8.06 (m, 1H, Ar), 10.53 (s, 1H, NH).

^{13}C NMR (DMSO-d6, 125 MHz): δ (ppm) 112.86, 119.26, 126.54, 127.61, 129.25, 131.02, 131.99, 133.84, 135.48, 149.75, 165.47.

4-chloro-*N*'-phenylbenzohydrazide (3g). White solid. Yield 90%.

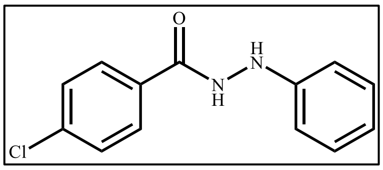

^{1}H NMR (DMSO-d6, 300 MHz): δ (ppm) 6.66-6.76 (m, 1H, Ar), 6.76-6.85 (m, 2H, Ar), 7.10-7.21 (m, 2H, Ar), 7.53-7.65 (m, 2H, Ar), 7.89-8.03 (m, 3H, Ar and NH), 10.47 (s, 1H, NH).

^{13}C NMR (DMSO-d6, 125 MHz): δ (ppm) 112.83, 119.22, 129.08, 129.25, 129.73, 132.25, 136.97, 149.85, 165.84.

General procedure for synthesis of hydrazonyl chlorides

In according to the reaction depicted in **Scheme S2,** hydrazonyl chlorides were synthesized through a modified procedure reported in literature [2].

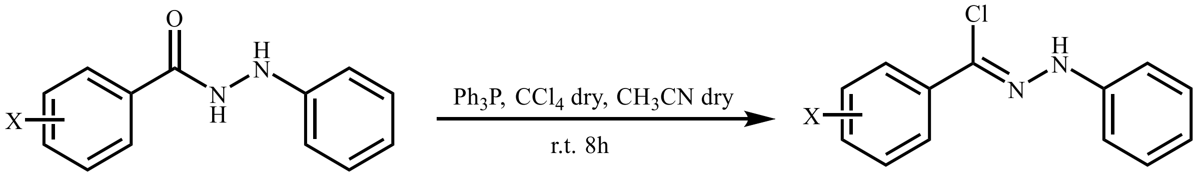

3a-3g **4a-4g**

Entry	R^1	Reagent	Product
1	H	**3a**	**4a**
2	4-OCH_3	**3b**	**4b**
3	4-CH_3	**3c**	**4c**
4	4-NO_2	**3d**	**4d**
5	2-Cl	**3e**	**4e**
6	3-Cl	**3f**	**4f**
7	4-Cl	**3g**	**4g**

***Scheme S2**. Synthesis of variously substituted hydrazonyl chlorides **4a-4g**.*

In 500 ml two-necked round-bottomed flask, equipped with magnetic stir bar, opportune benzoyl phenylhydrazine **3a-3g** (59.7 mmol, 1 eq) was suspended under nitrogen in dry acetonitrile (100 ml). Subsequently, triphenylphosphine (74.6 mmol, 1.25 eq) and dry carbon tetrachloride (89.6 mmol, 1.5 eq) were respectively added. The reaction mixture was stirred at room temperature for 4h. During the reaction course, suspended benzoyl phenylhydrazine completely dissolved and then, the formation of a solid was observed. At the end of the reaction, the solution was cooled in an ice-bath and the solid was recuperate by filtration and washed with small quantity of acetonitrile. The obtained solid was recrystallized with acetonitrile.

***N*-phenylbenzohydrazonyl chloride (4a).** White solid. Yield 80%.

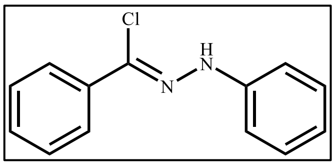

^{1}H NMR (DMSO-d_6, 300 MHz): δ (ppm) 6.82-6.96 (m, 1H, Ar), 7.23-7.33 (m, 2H, Ar), 7.33-7.41 (m, 2H, Ar), 7.42-7.60 (m, 3H, Ar), 7.83-8.00 (m, 2H, Ar), 9.92 (s, 1H, NH).

^{13}C NMR (DMSO-d_6, 125 MHz): δ (ppm) 114.06, 120.96, 122.73, 126.44, 129.22, 129.59, 129.65, 134.82, 144.61.

4-methoxy-*N*-phenylbenzohydrazonyl chloride (4b). White solid. Yield 88%.

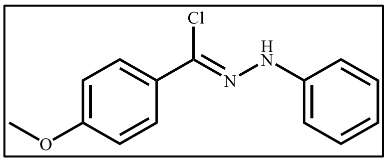

^{1}H NMR ($CDCl_3$, 300 MHz): δ (ppm) 3.84 (s, 3H, CH_3), 6.86-6.98 (m, 3H, Ar), 7.11-7.20 (m, 2H, Ar), 7.25-7.35 (m, 2H, Ar), 7.80-7.89 (m, 2H, Ar), 7.93 (bs, 1H, NH).

^{13}C NMR ($CDCl_3$, 125 MHz): δ (ppm) 55.36, 113.27, 113.77, 120.83, 124.71, 127.15, 127.88, 129.31, 143.57, 160.54.

4-methyl-*N*-phenylbenzohydrazonyl chloride (4c). White solid. Yield 84%.

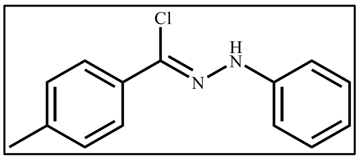

^{1}H NMR ($CDCl_3$, 300 MHz): δ (ppm) 2.38 (s, 3H, CH_3), 6.87-6.98 (m, 1H, Ar), 7.11-7.25 (m, 4H, Ar), 7.25-7.36 (m, 2H, Ar), 7.75-7.87 (m, 2H, Ar), 7.98 (bs, 1H, NH).

^{13}C NMR ($CDCl_3$, 125 MHz): δ (ppm) 21.25, 113.35, 120.98, 124.93, 126.36, 129.09, 129.34, 131.76, 139.38, 143.46.

4-nitro-*N*-phenylbenzohydrazonyl chloride (4d). Red solid. Yield 80%.

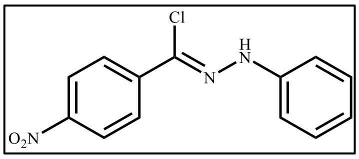

^{1}H NMR ($CDCl_3$, 300 MHz): δ (ppm) 6.95-7.08 (m, 1H, Ar), 7.16-7.24 (m, 2H, Ar), 7.30-7.41 (m, 2H, Ar), 8.00-8.10 (m, 2H, Ar), 8.19-8.33 (m, 3H, Ar and NH).

^{13}C NMR ($CDCl_3$, 125 MHz): δ (ppm) 113.79, 122.10, 122.22, 123.72, 126.58, 129.51, 140.14, 142.39, 147.60.

2-chloro-*N*-phenylbenzohydrazonyl chloride (4e). White solid. Yield 78%.

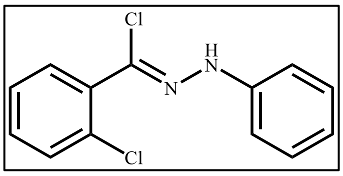

^{1}H NMR ($CDCl_3$, 300 MHz): δ (ppm) 6.88-6.99 (m, 1H, Ar), 7.09-7.19 (m, 2H, Ar), 7.21-7.37 (m, 4H, Ar), 7.40-7.50 (m, 1H, Ar), 7.55-7.64 (m, 1H, Ar), 8.07 (bs, 1H, NH).

^{13}C NMR ($CDCl_3$, 125 MHz): δ (ppm) 113.48, 120.41, 121.35, 126.68, 129.35, 130.24, 130.57, 131.24, 132.91, 134.19, 143.12.

3-chloro-*N*-phenylbenzohydrazonyl chloride (4f). White solid. Yield 83%.

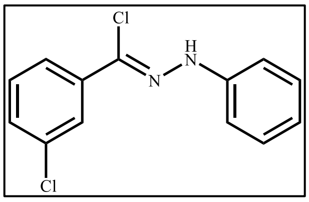

^{1}H NMR ($CDCl_3$, 300 MHz): δ (ppm) 6.90-7.01 (m, 1H, Ar), 7.12-7.21 (m, 2H, Ar), 7.26-7.38 (m, 4H, Ar), 7.74-7.83 (m, 1H, Ar), 7.85-7.94 (m, 1H, Ar), 8.06 (bs, 1H, NH).

^{13}C NMR ($CDCl_3$, 125 MHz): δ (ppm) 113.53, 121.51, 122.97, 124.41, 126.22, 129.03, 129.41, 129.56, 134.52, 136.15, 142.96.

4-chloro-*N*-phenylbenzohydrazonyl chloride (4g). White solid. Yield 84%.

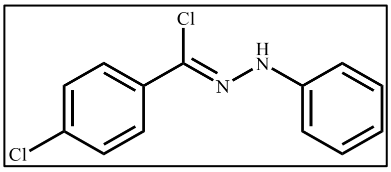

^{1}H NMR ($CDCl_3$, 300 MHz): δ (ppm) 6.90-7.00 (m, 1H, Ar), 7.12-7.21 (m, 2H, Ar), 7.26-7.41 (m, 4H, Ar), 7.79-7.89 (m, 2H, Ar), 8.03 (bs, 1H, NH).

^{13}C-NMR ($CDCl_3$, 125 MHz): δ (ppm) 113.46, 121.38, 123.47, 127.49, 128.59, 129.39, 132.94, 135.10, 143.09.

General procedure for synthesis of [mPy]OTf

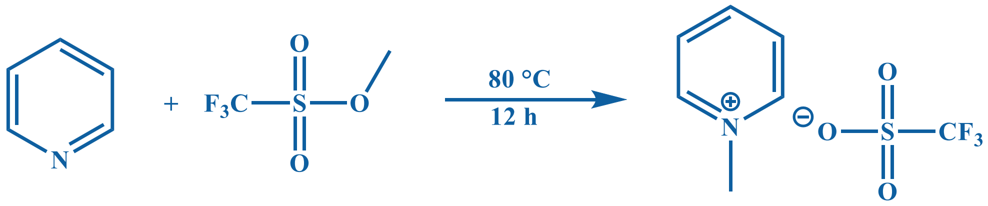

The ionic liquid [mPy][OTf] was prepared in total halide free direct synthesis in according to literature procedure [3]. Specifically, 0.29 mols of dry pyridine was placed in a two-necked flask and 0.30 mols of methyltrifluoromethanesulfonate dropwise was added. The mixture was reacted at 80 °C for 12 hours. The crude product was washed with diethyl ether and dried under vacuum, providing 68.04 gr (yield 96%) of pure ionic liquid.

Characterization spectra of benzoyl phenylhydrazines

N'-phenylbenzohydrazide (3a)

^{1}H NMR

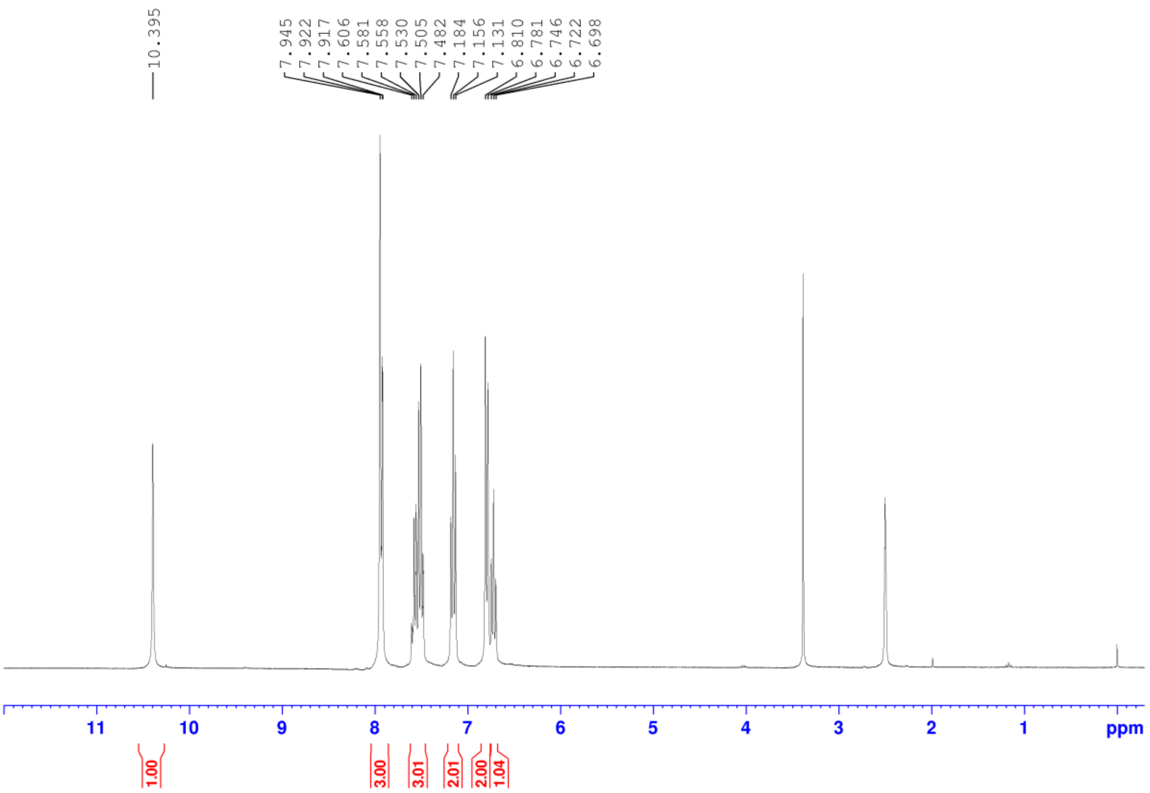

^{13}C NMR

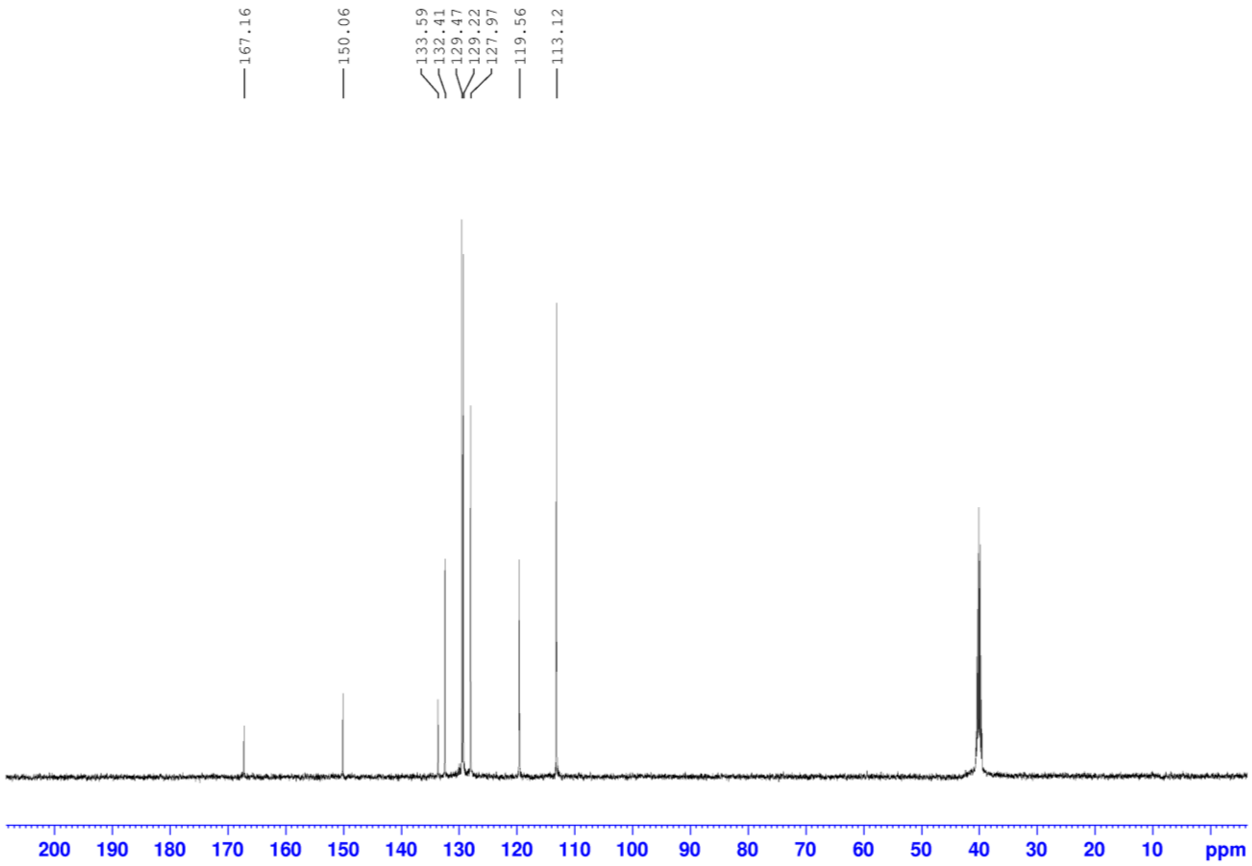

167.16
150.06
133.59
132.41
129.47
129.22
127.97
119.56
113.12
200
190
180
170
160
150
140
130
120
110
100
90
80
70
60
50
40
30
20
10
ppm

4-methoxy-N'-phenylbenzohydrazide (3b)

^{1}H NMR

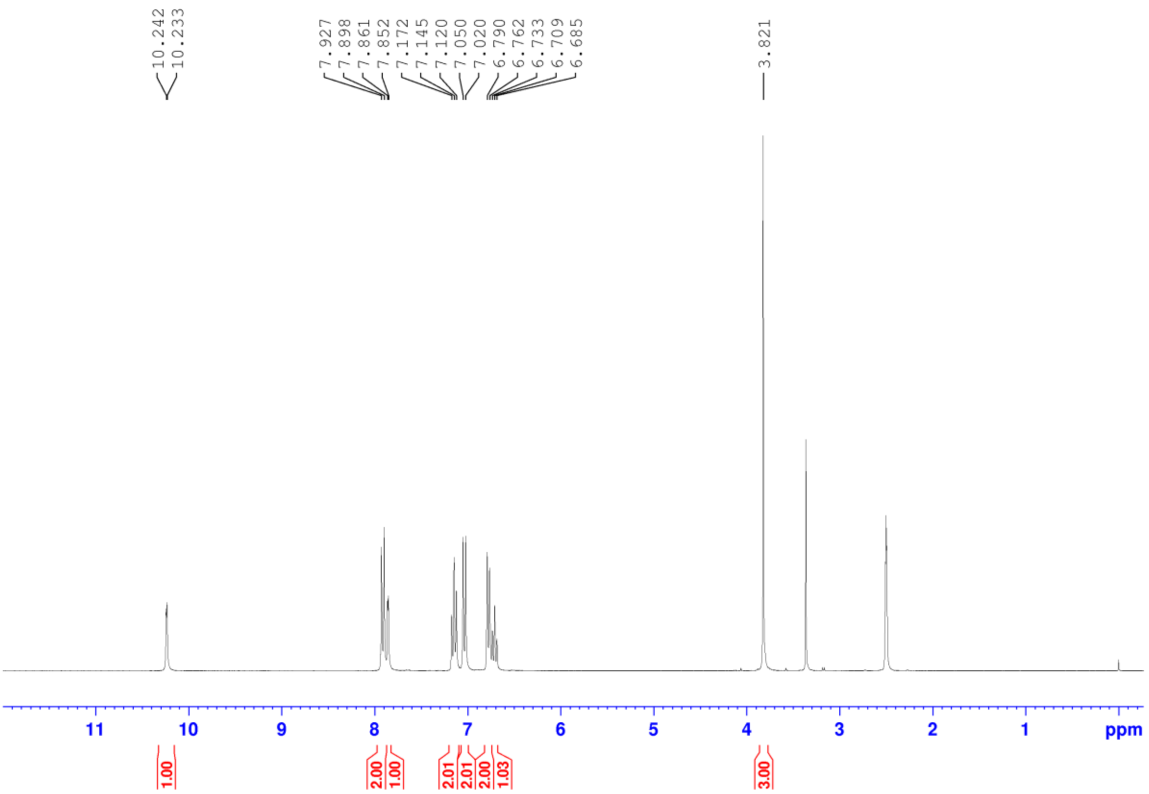

^{13}C NMR

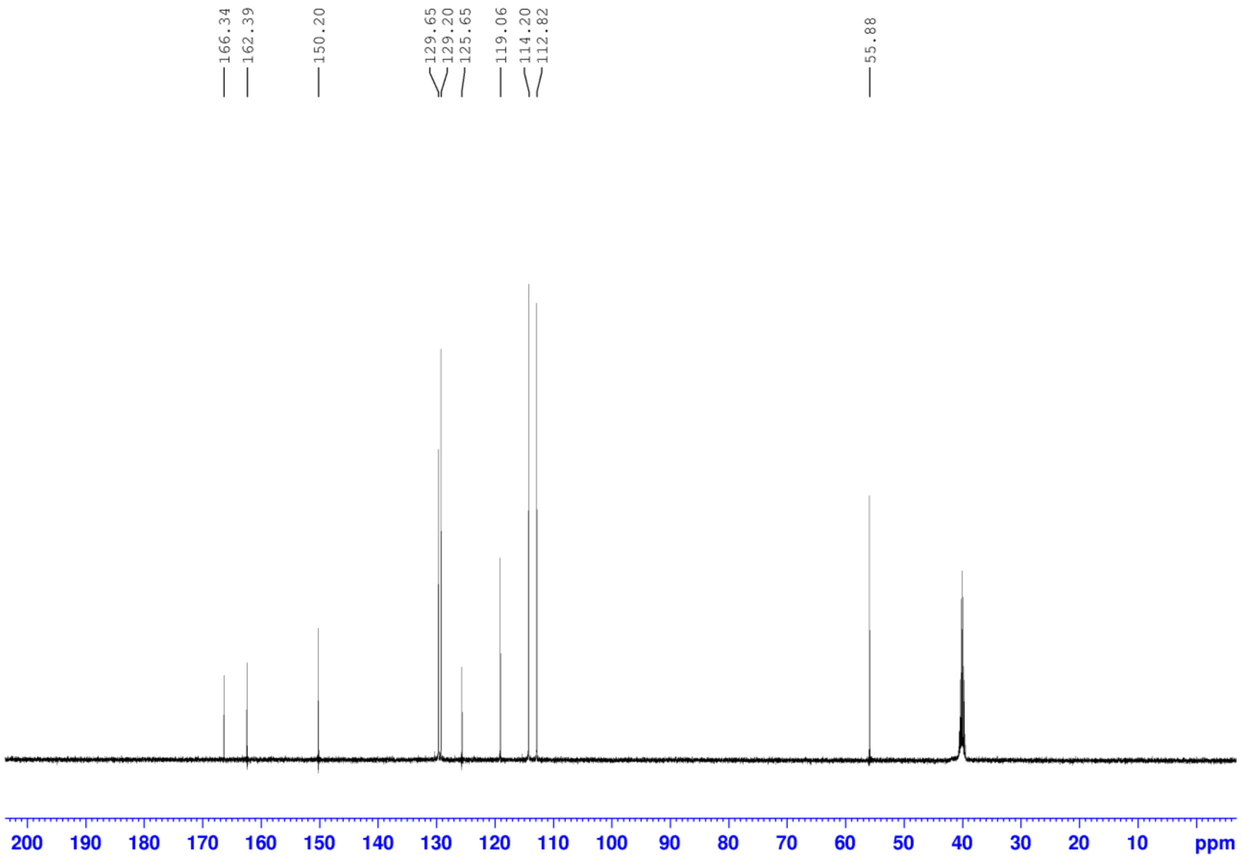
166.34
162.39
150.20
129.65
129.20
125.65
119.06
114.20
112.82
55.88
200
190
180
170
160
150
140
130
120
110
100
90
80
70
60
50
40
30
20
10
ppm

4-methyl-N'-phenylbenzohydrazide (3c)

¹H NMR

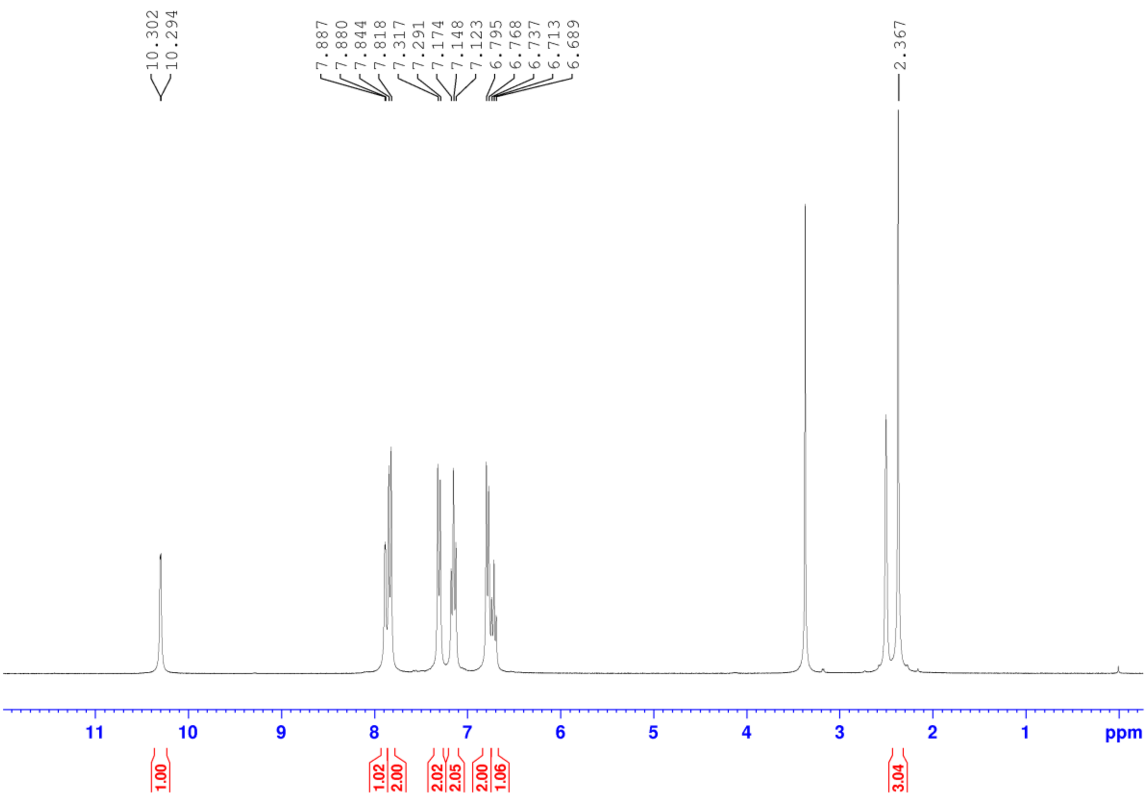

^{13}C NMR

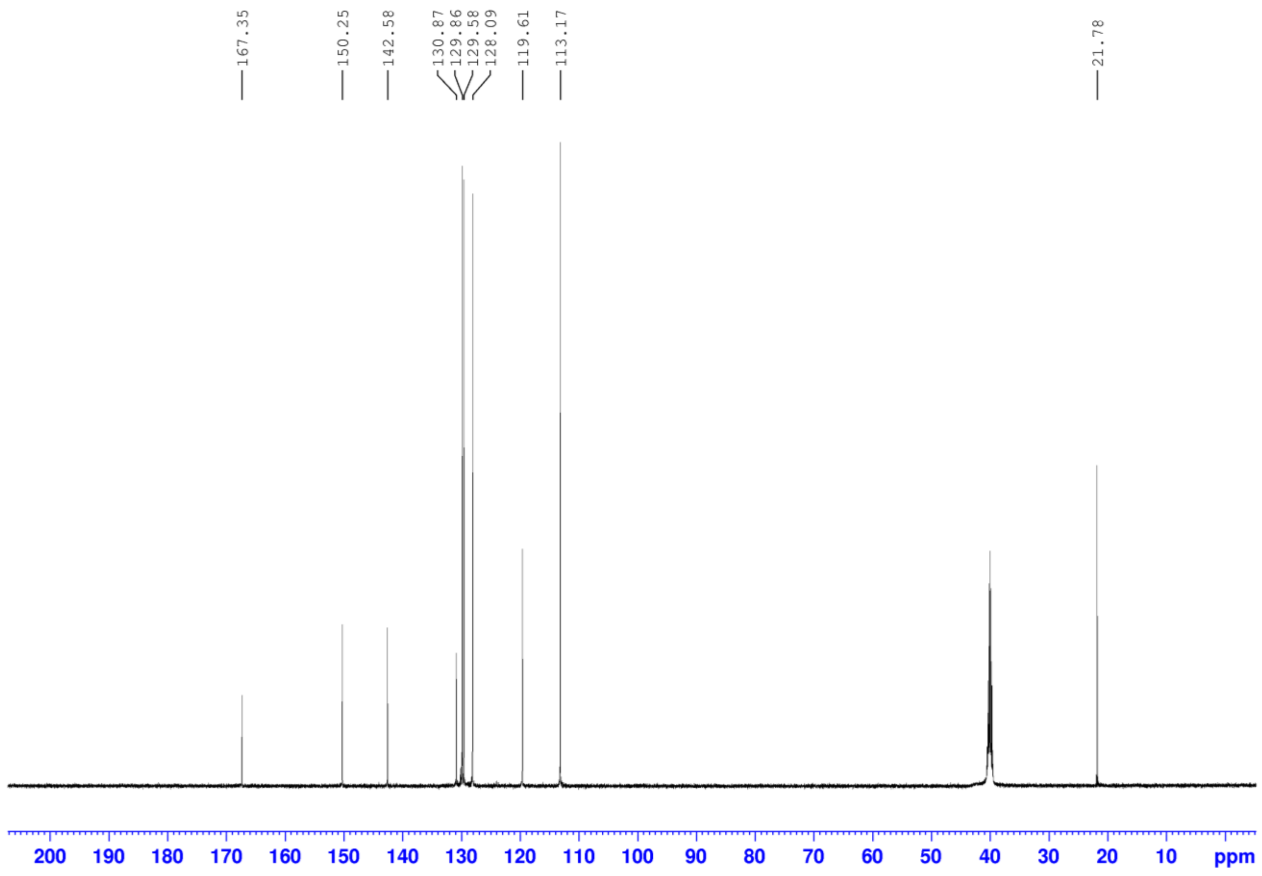
167.35
150.25
142.58
130.87
129.86
129.58
128.09
119.61
113.17
21.78
200 190 180 170 160 150 140 130 120 110 100 90 80 70 60 50 40 30 20 10 ppm

4-nitro-N'-phenylbenzohydrazide (3d)

1H NMR

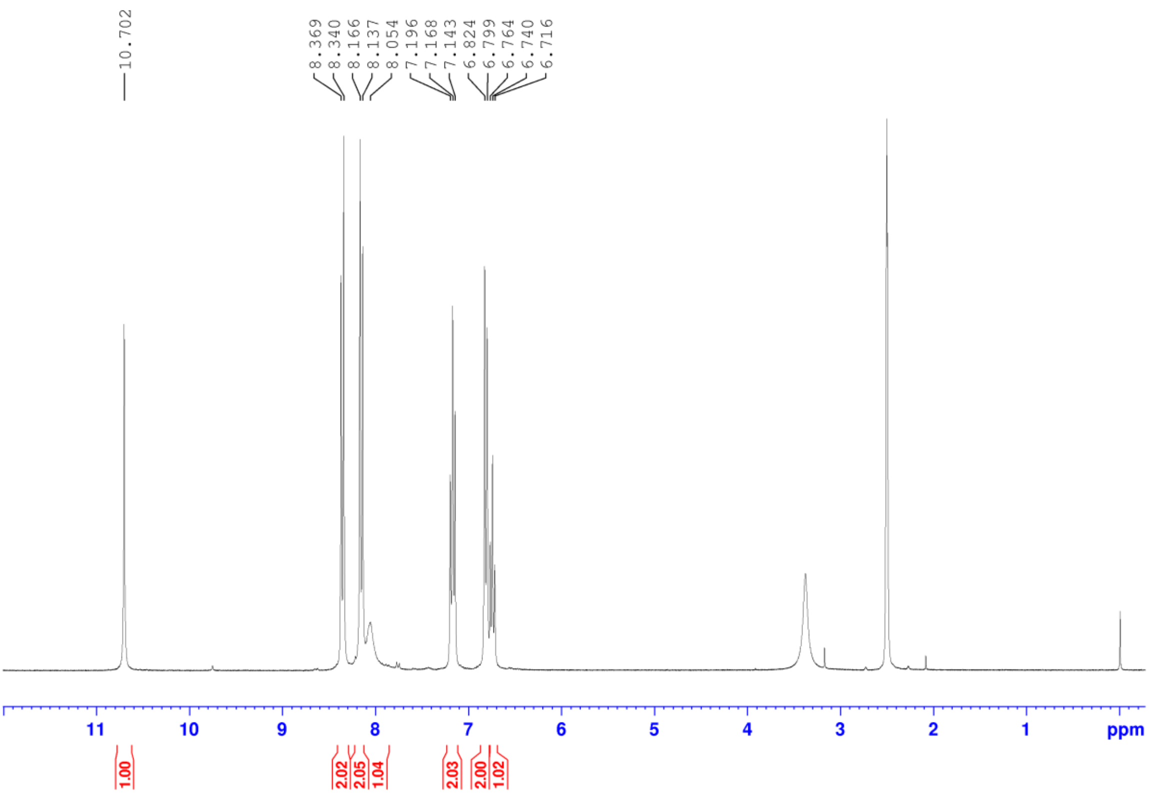

^{13}C NMR

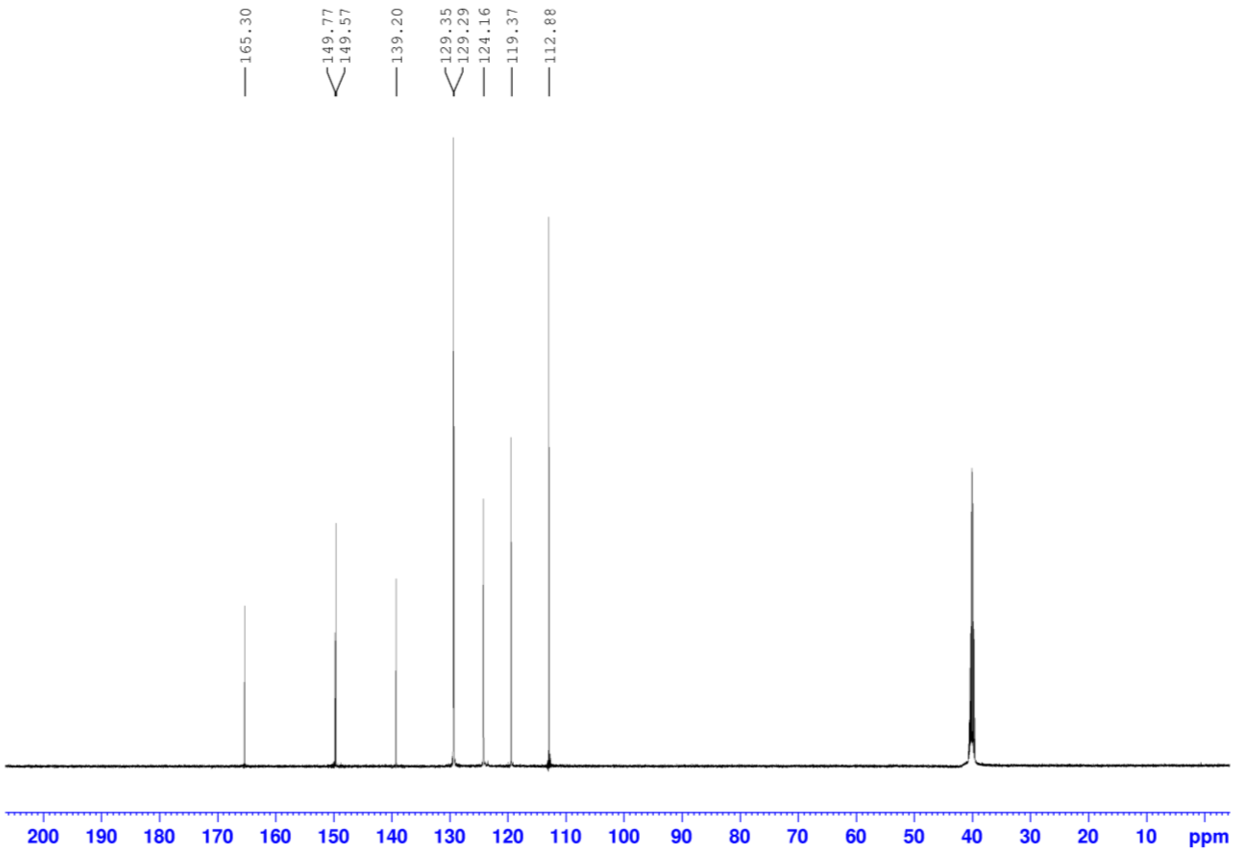
165.30
149.77
149.57
139.20
129.35
129.29
124.16
119.37
112.88
200 190 180 170 160 150 140 130 120 110 100 90 80 70 60 50 40 30 20 10 ppm

2-chloro-N'-phenylbenzohydrazide (3e)

^{1}H NMR

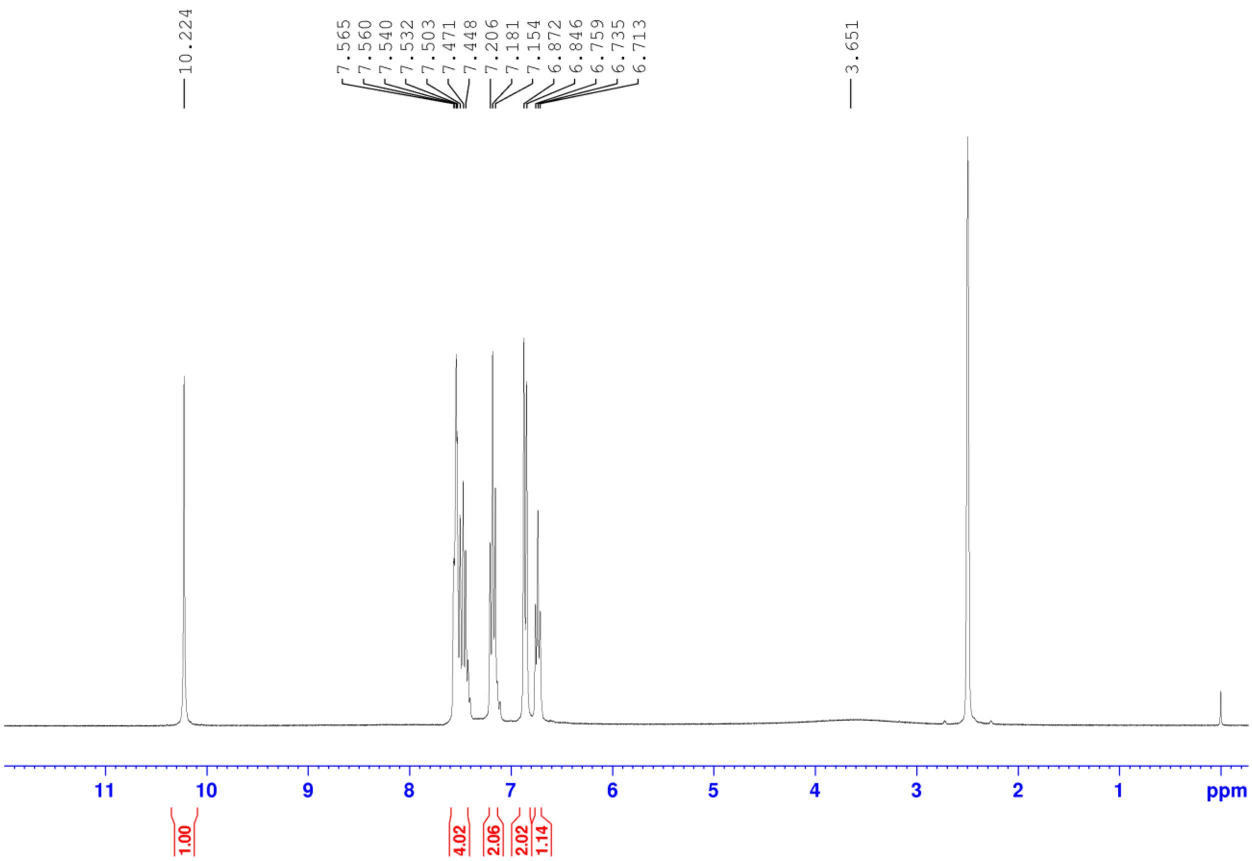

^{13}C NMR

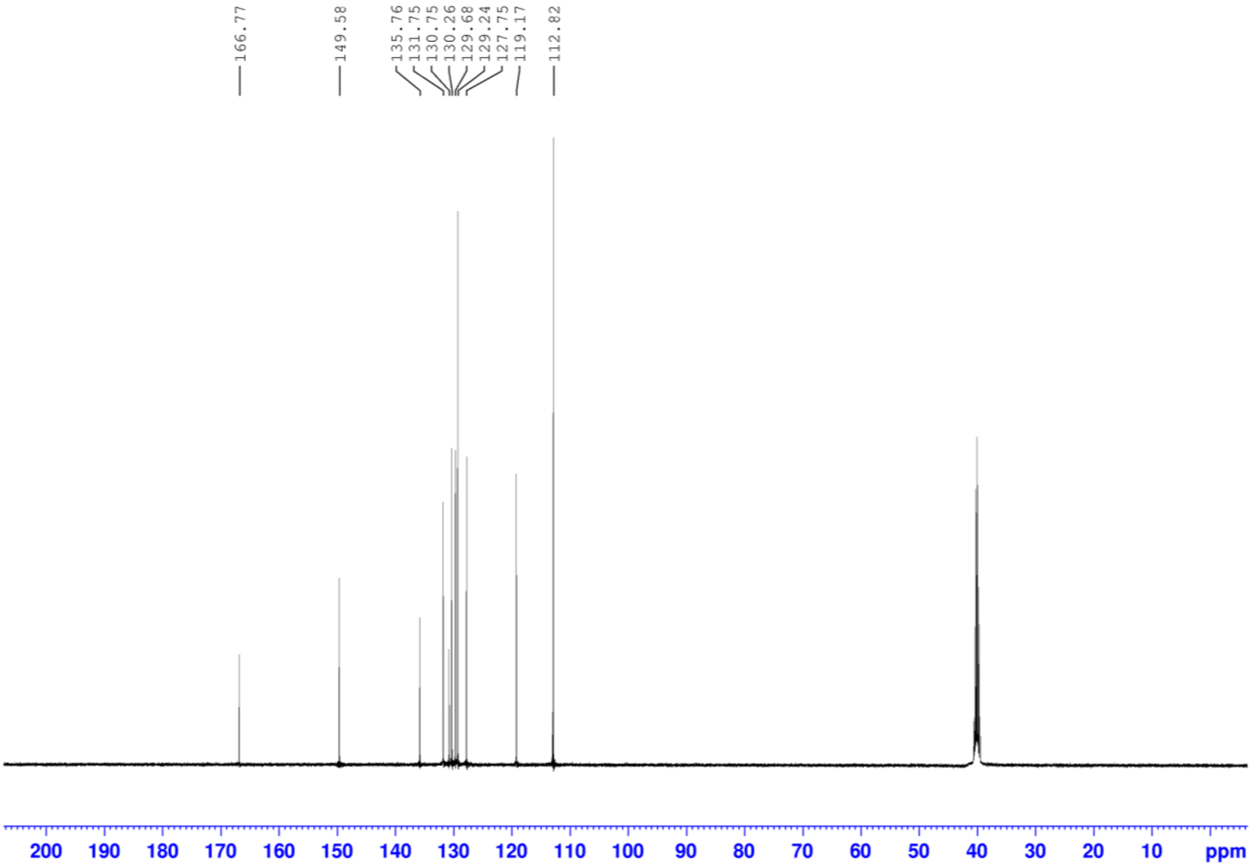
166.77
149.58
135.76
131.75
130.75
130.26
129.68
129.24
127.75
119.17
112.82
200 190 180 170 160 150 140 130 120 110 100 90 80 70 60 50 40 30 20 10 ppm

3-chloro-N'-phenylbenzohydrazide (3f)

^{1}H NMR

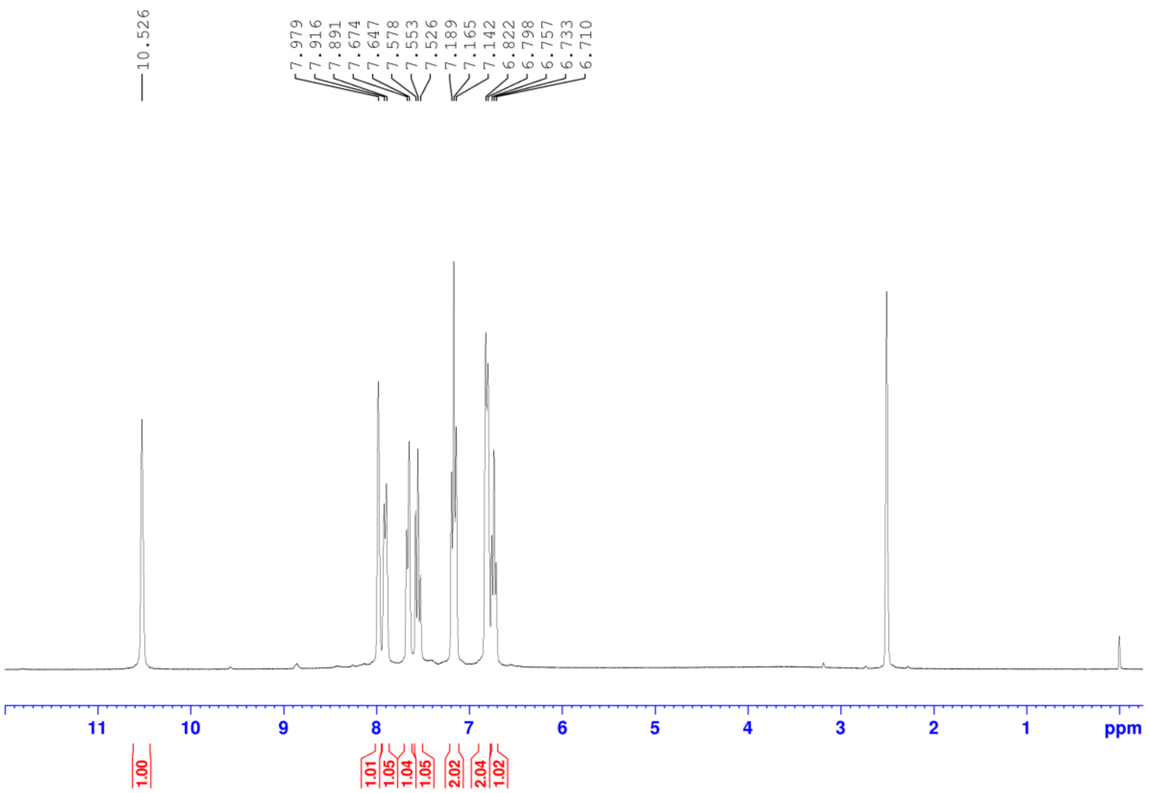

^{13}C NMR

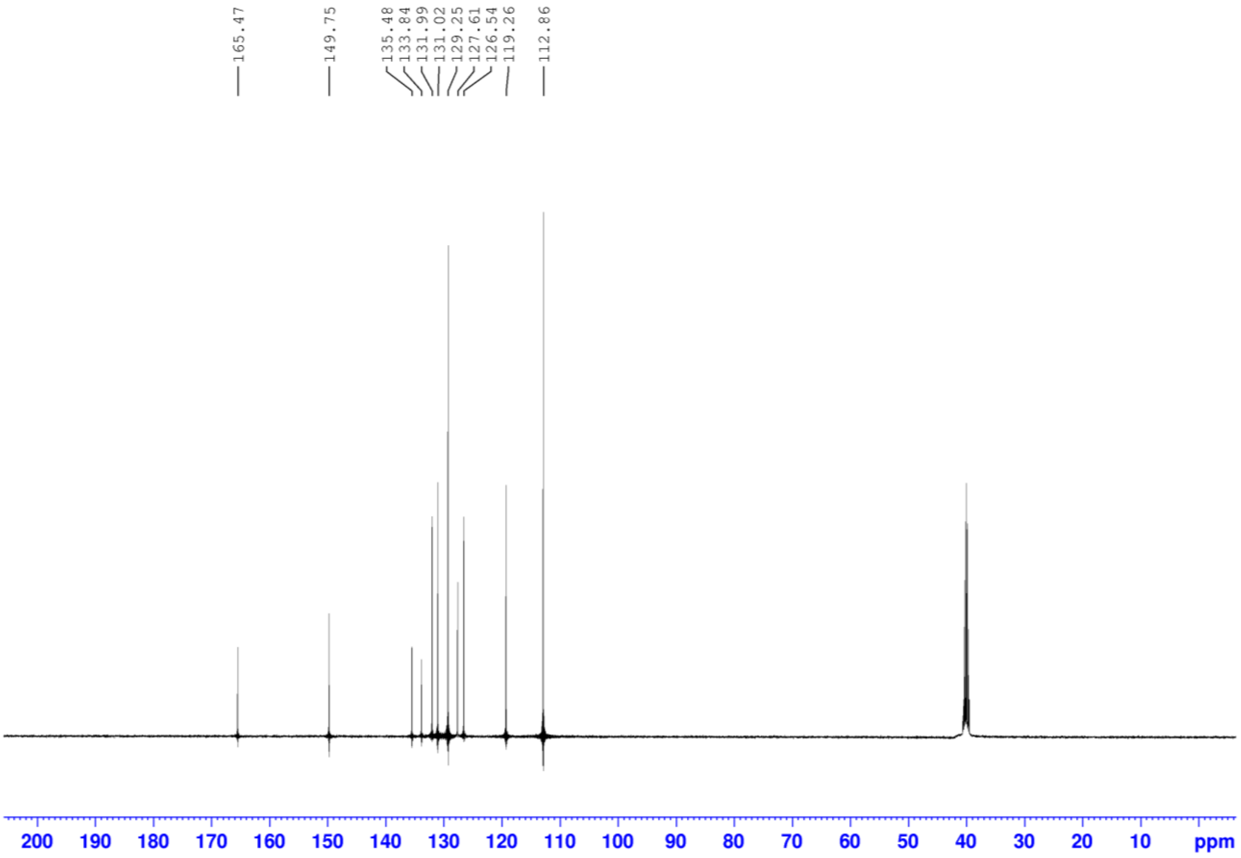
165.47
149.75
135.48
133.84
131.99
131.02
129.25
127.61
126.54
119.26
112.86
200 190 180 170 160 150 140 130 120 110 100 90 80 70 60 50 40 30 20 10 ppm

4-chloro-N'-phenylbenzohydrazide (3g)

^{1}H NMR

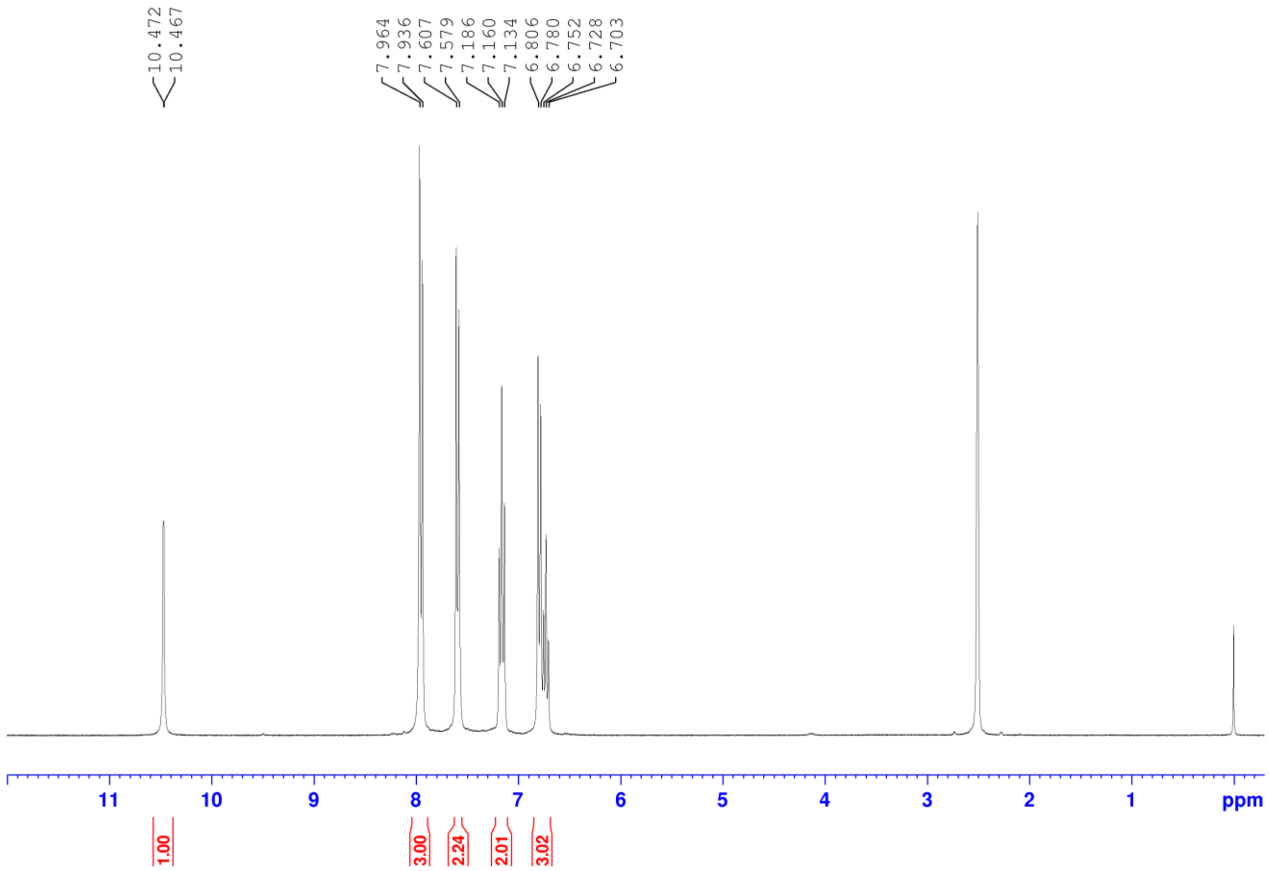

^{13}C NMR

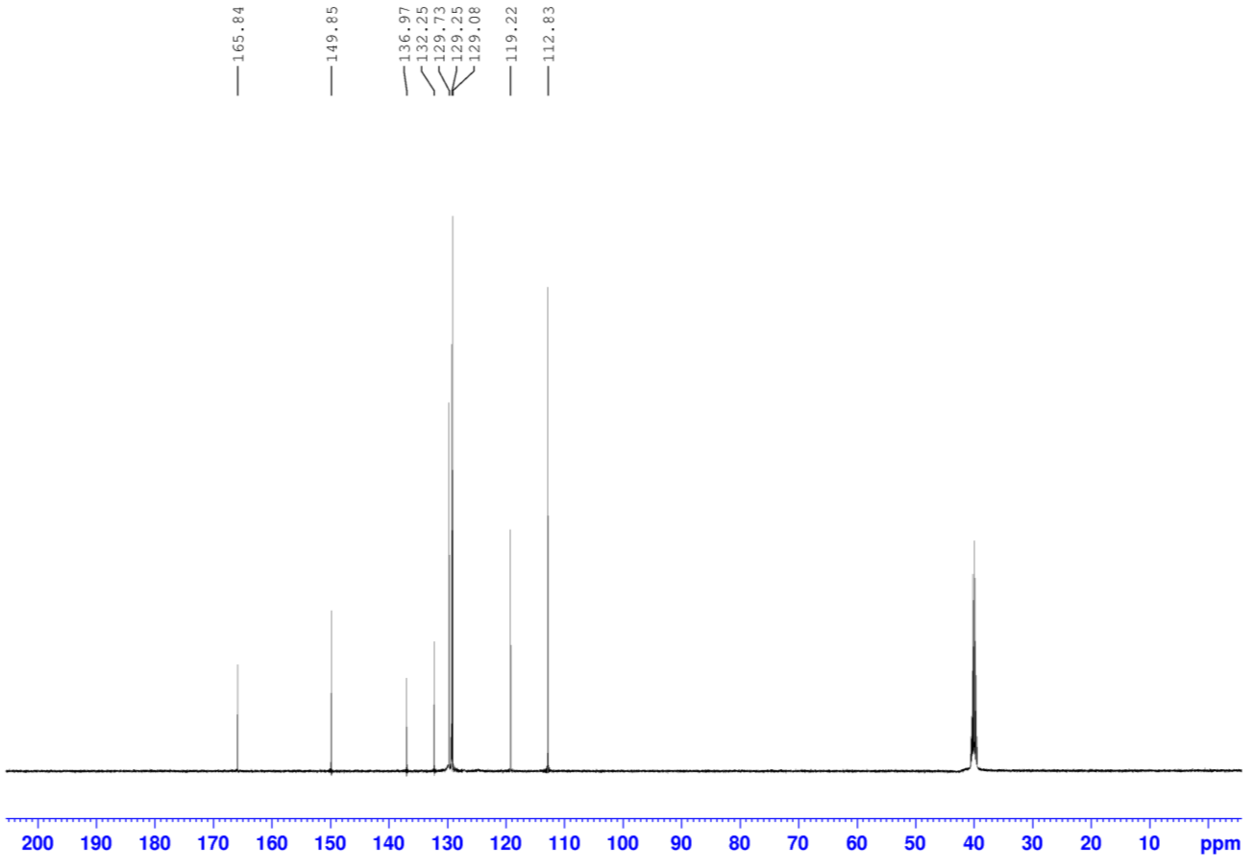

165.84
149.85
136.97
132.25
129.73
129.25
129.08
119.22
112.83
200 190 180 170 160 150 140 130 120 110 100 90 80 70 60 50 40 30 20 10 ppm

Characterization spectra of hydrazonyl chlorides

N-phenylbenzohydrazonyl chloride (4a)

^{1}H NMR

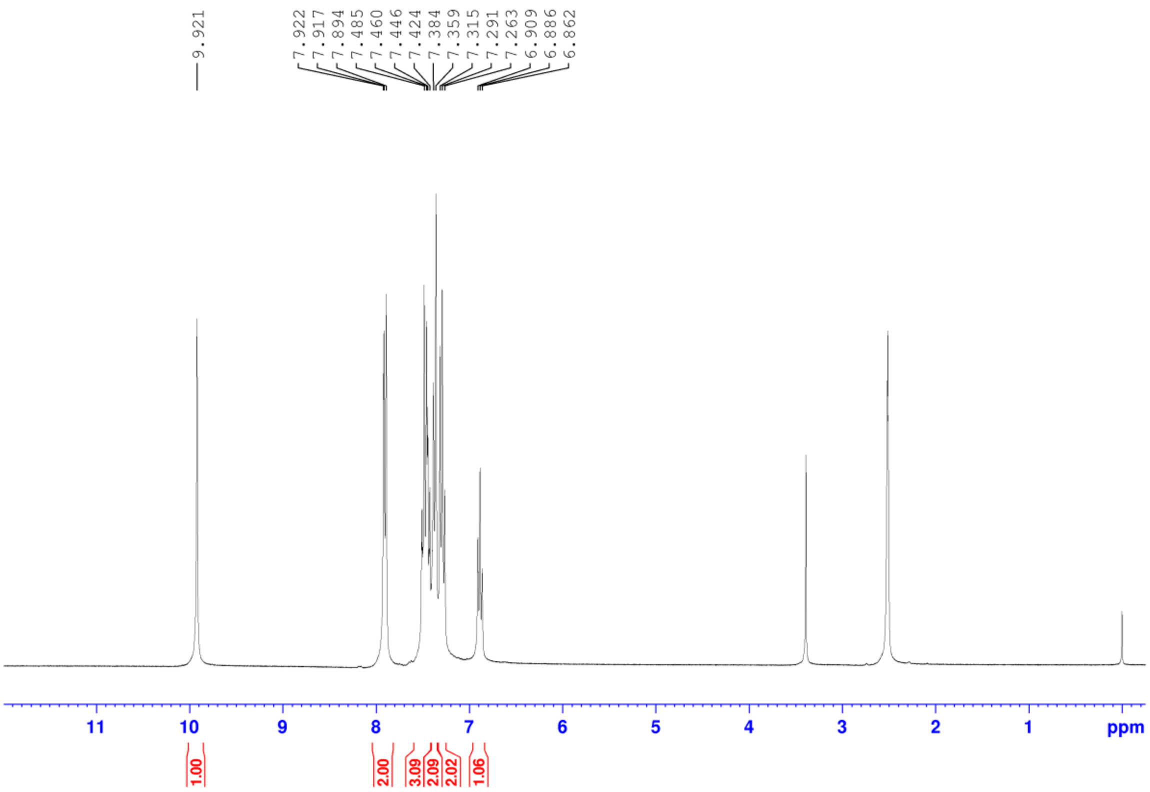

^{13}C NMR

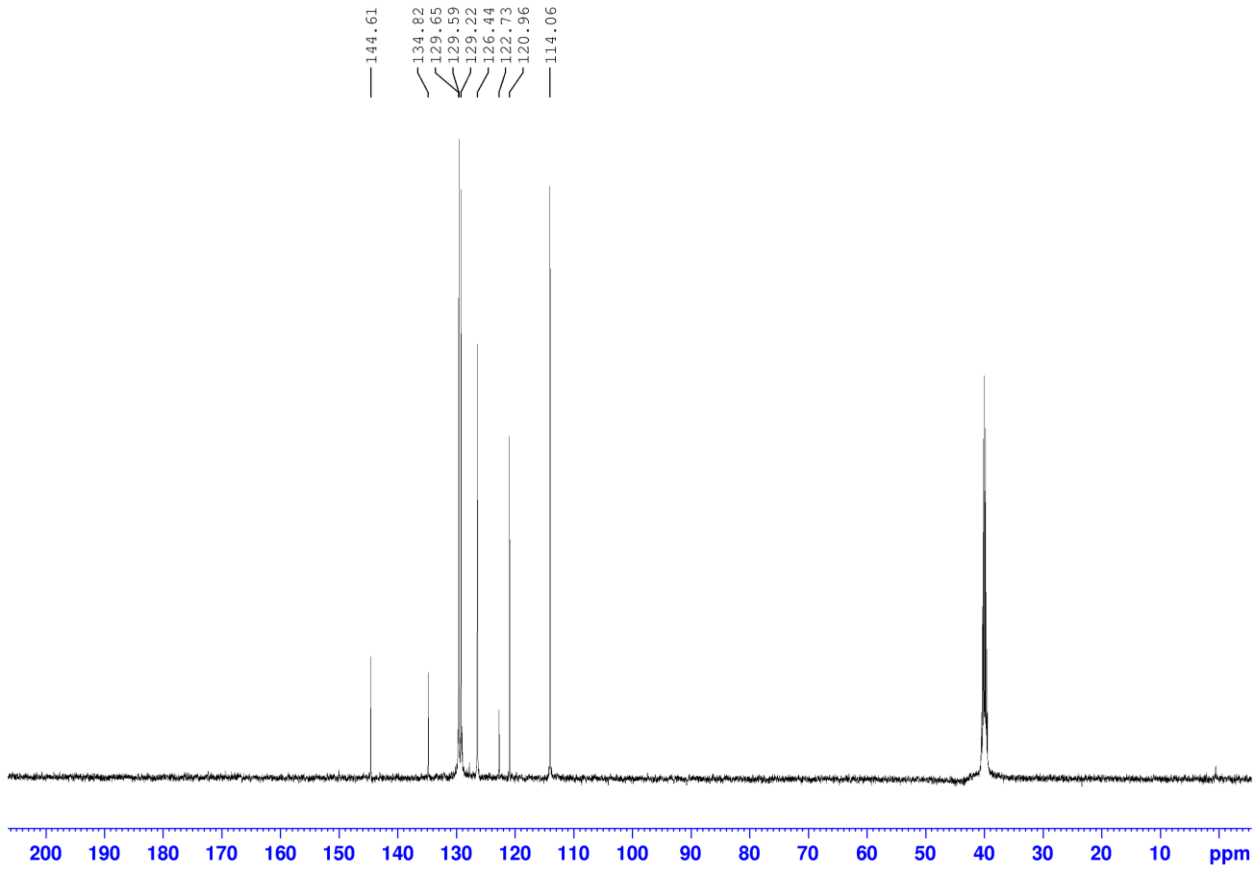
144.61
134.82
129.65
129.59
129.22
126.44
122.73
120.96
114.06
200
190
180
170
160
150
140
130
120
110
100
90
80
70
60
50
40
30
20
10
ppm

4-methoxy-N-phenylbenzohydrazonyl chloride (4b)

^{1}H NMR

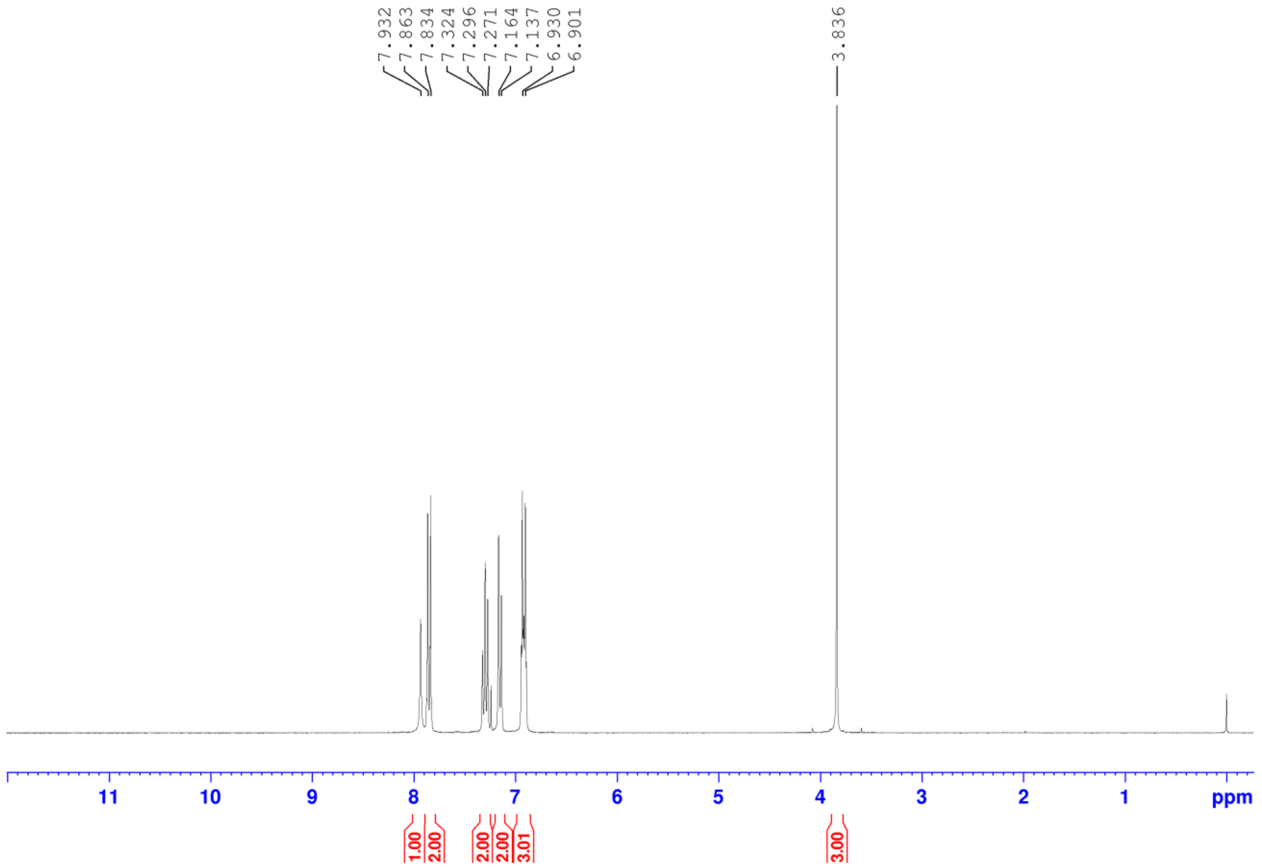

^{13}C NMR

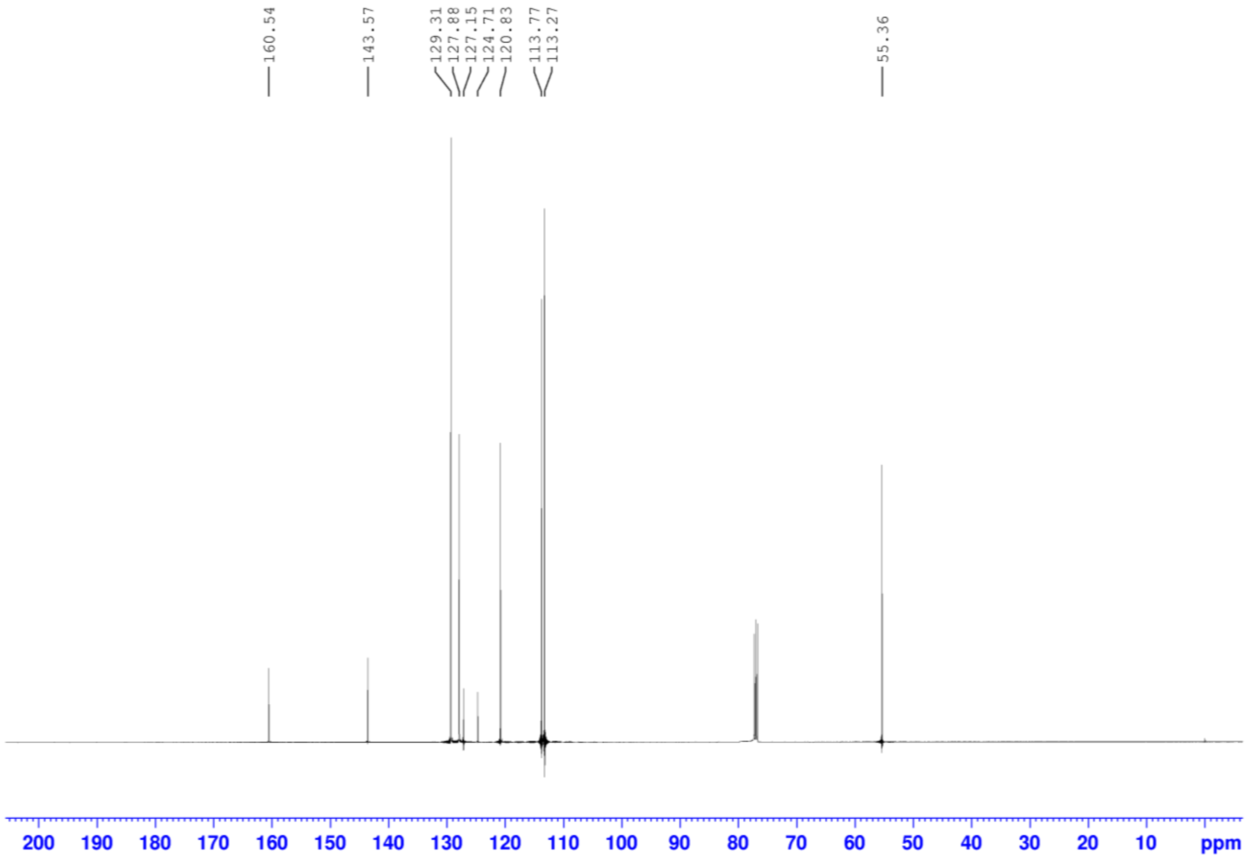
160.54
143.57
129.31
127.88
127.15
124.71
120.83
113.77
113.27
55.36
200 190 180 170 160 150 140 130 120 110 100 90 80 70 60 50 40 30 20 10 ppm

4-methyl-N-phenylbenzohydrazonyl chloride (4c)

^{1}H NMR

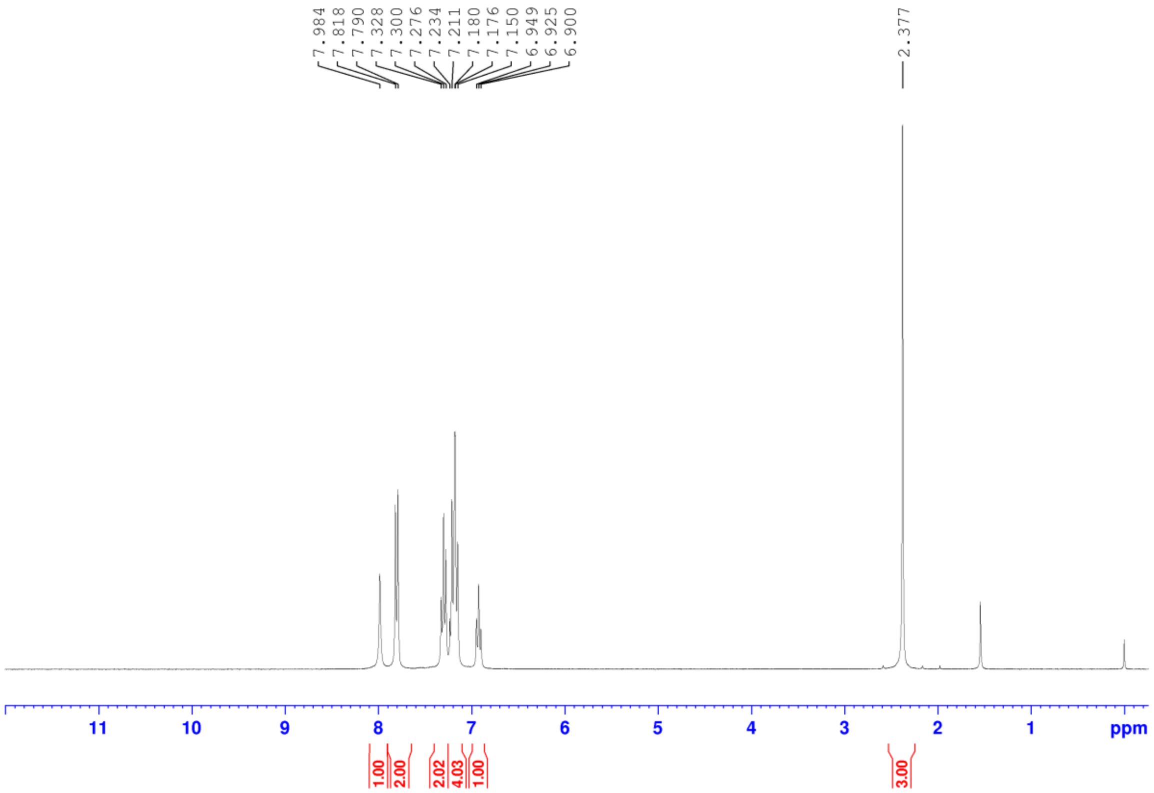

^{13}C NMR

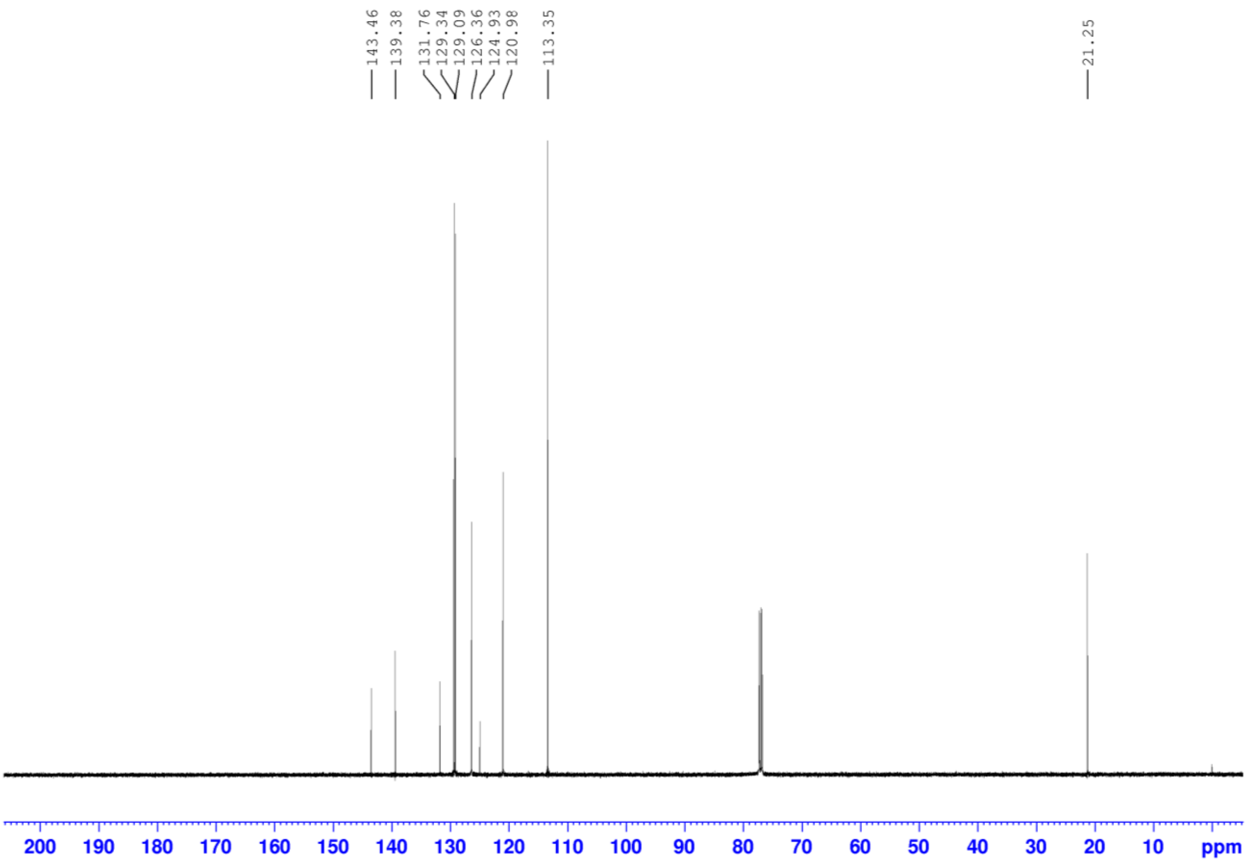

143.46
139.38
131.76
129.34
129.09
126.36
124.93
120.98
113.35
21.25
200 190 180 170 160 150 140 130 120 110 100 90 80 70 60 50 40 30 20 10 ppm

4-nitro-N-phenylbenzohydrazonyl chloride (4d)

^{1}H NMR

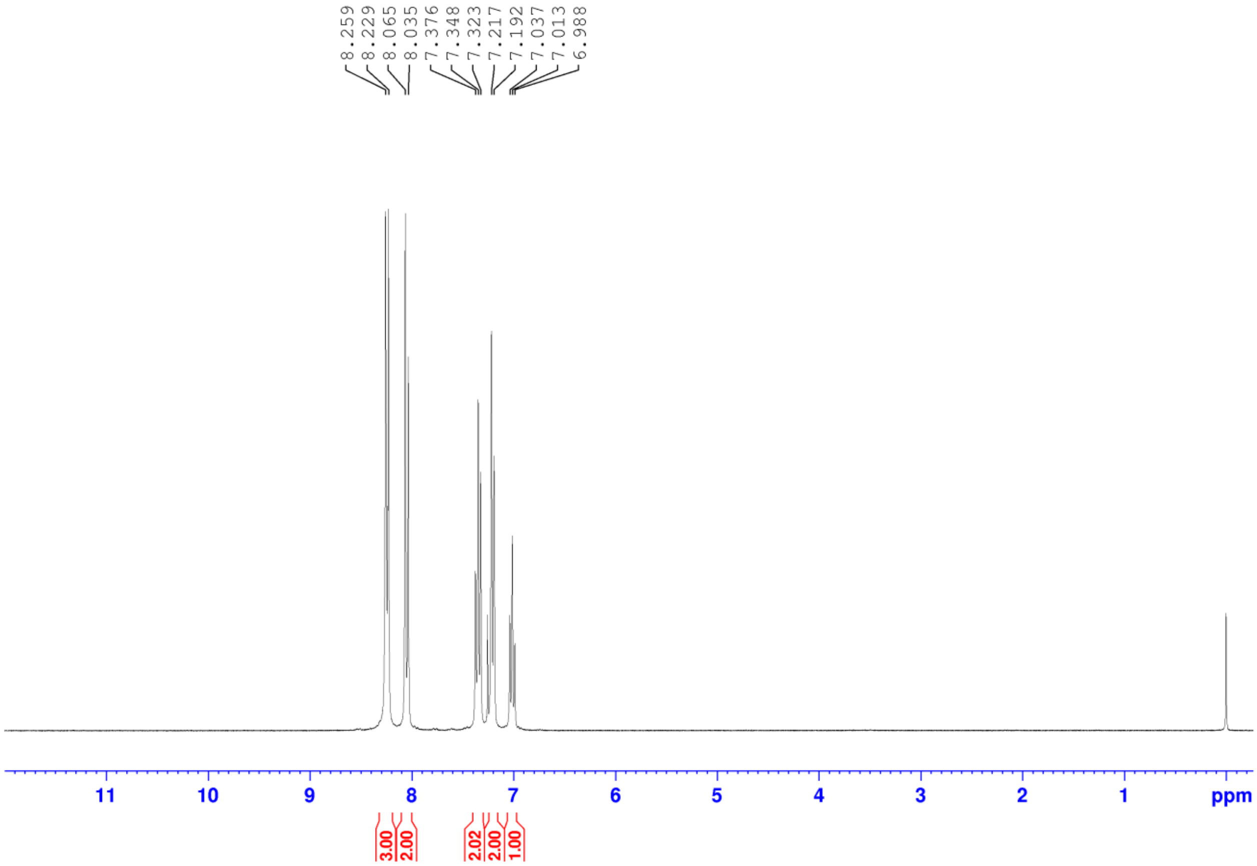

^{13}C NMR

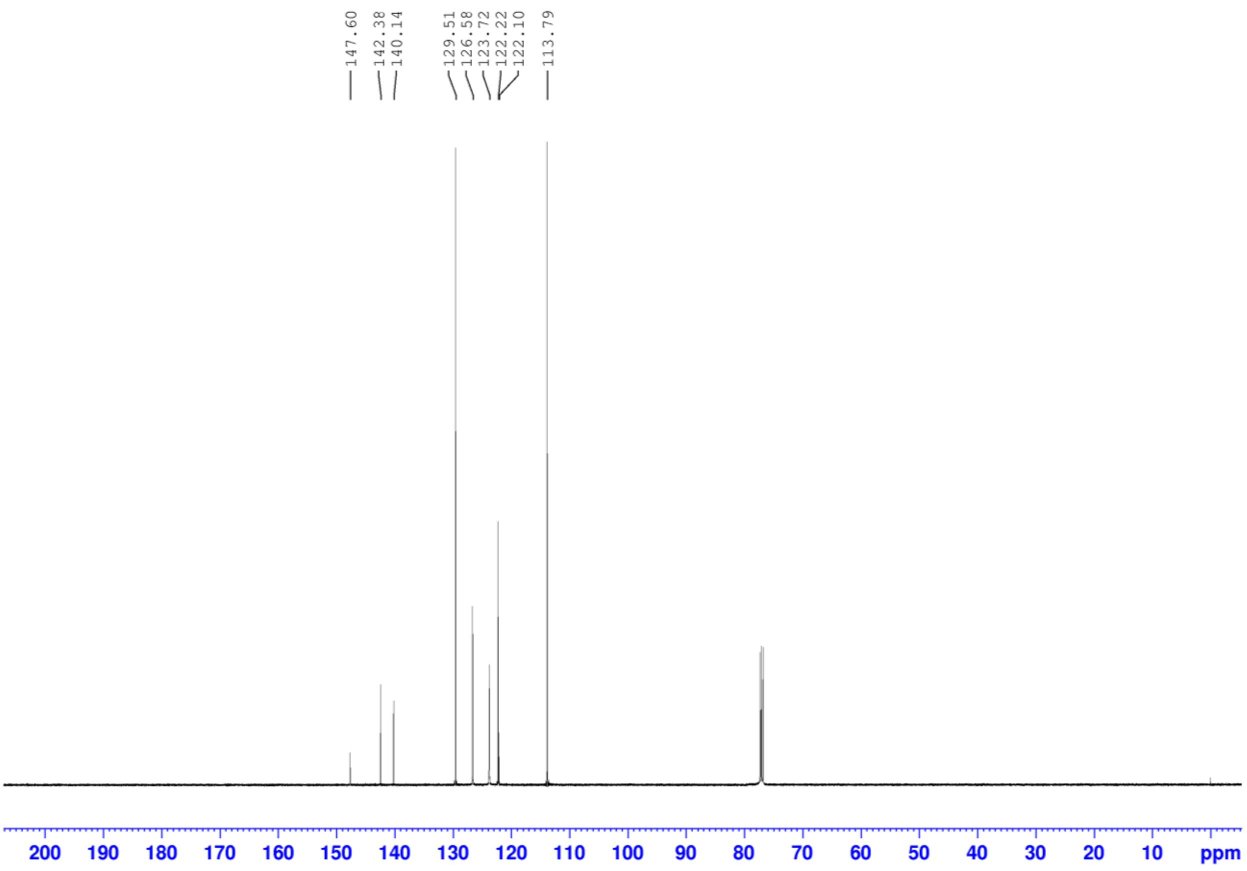

147.60
142.38
140.14
129.51
126.58
123.72
122.22
122.10
113.79
200
190
180
170
160
150
140
130
120
110
100
90
80
70
60
50
40
30
20
10
ppm

2-chloro-N-phenylbenzohydrazonyl chloride (4e)

^{1}H NMR

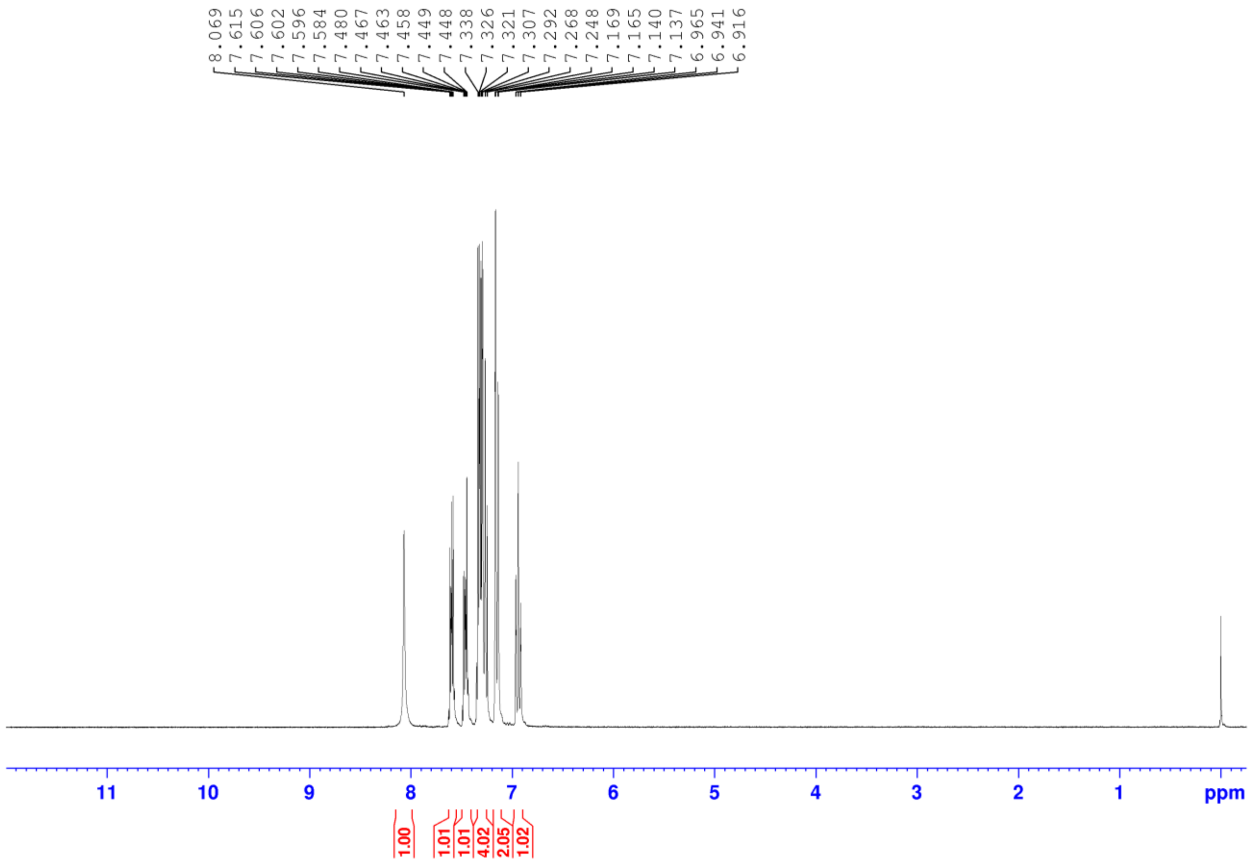

^{13}C NMR

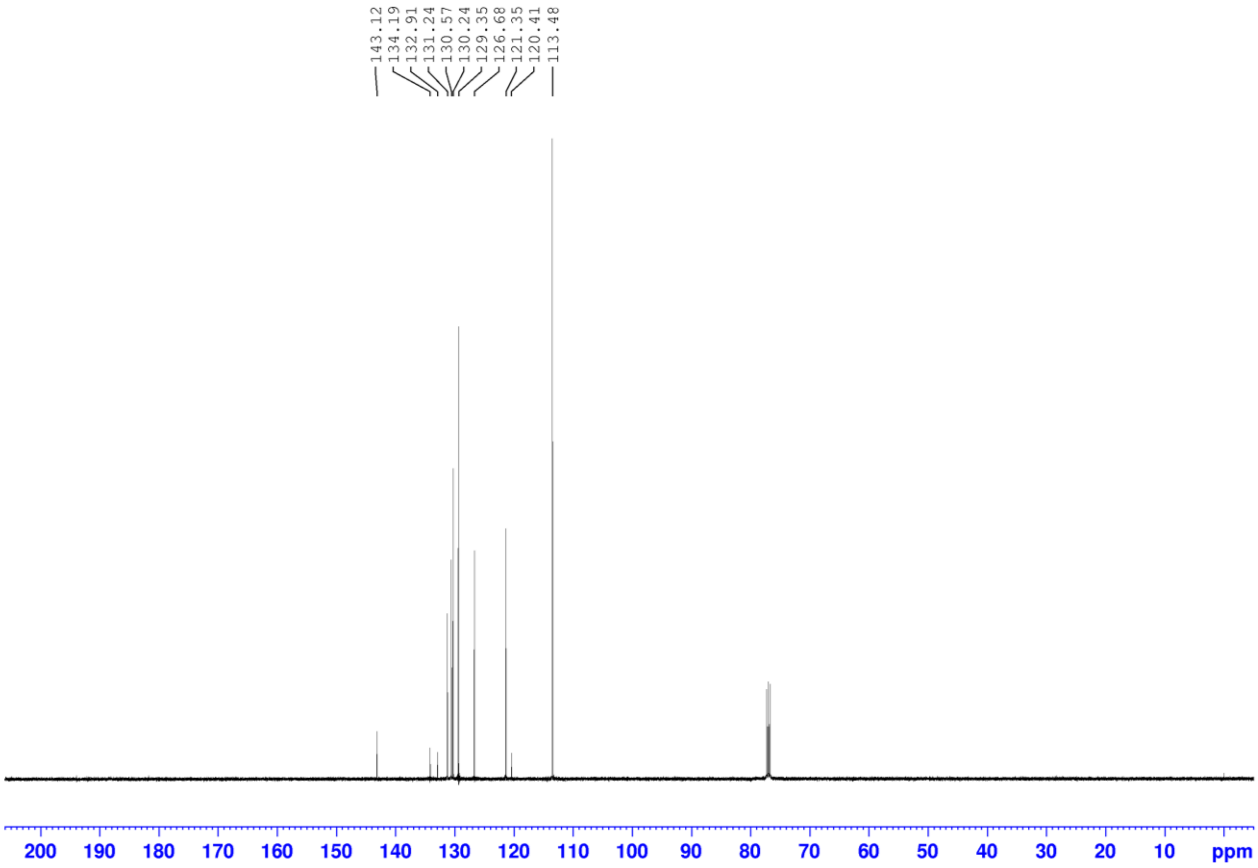
143.12
134.19
132.91
131.24
130.57
130.24
129.35
126.68
121.35
120.41
113.48
200 190 180 170 160 150 140 130 120 110 100 90 80 70 60 50 40 30 20 10 ppm

3-chloro-N-phenylbenzohydrazonyl chloride (4f)

^{1}H NMR

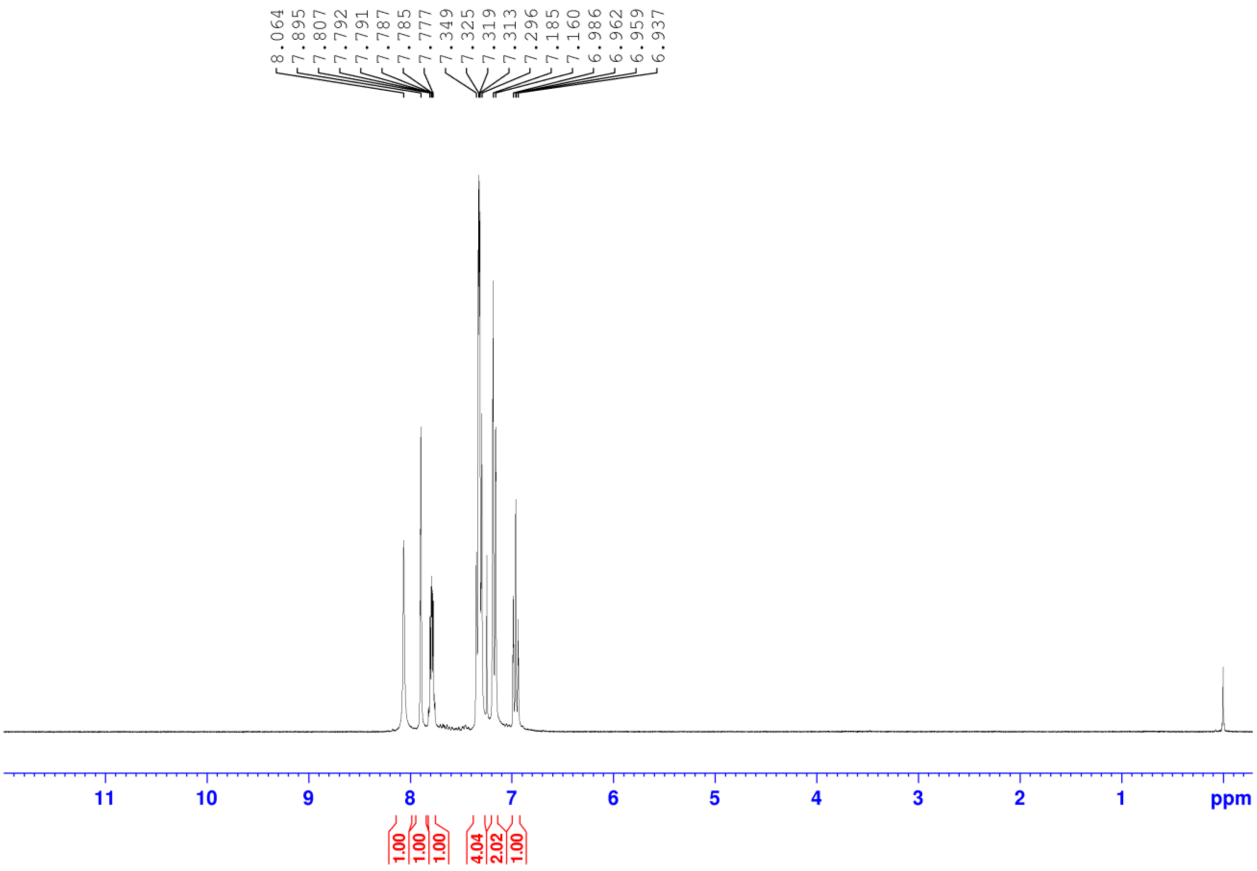

^{13}C NMR

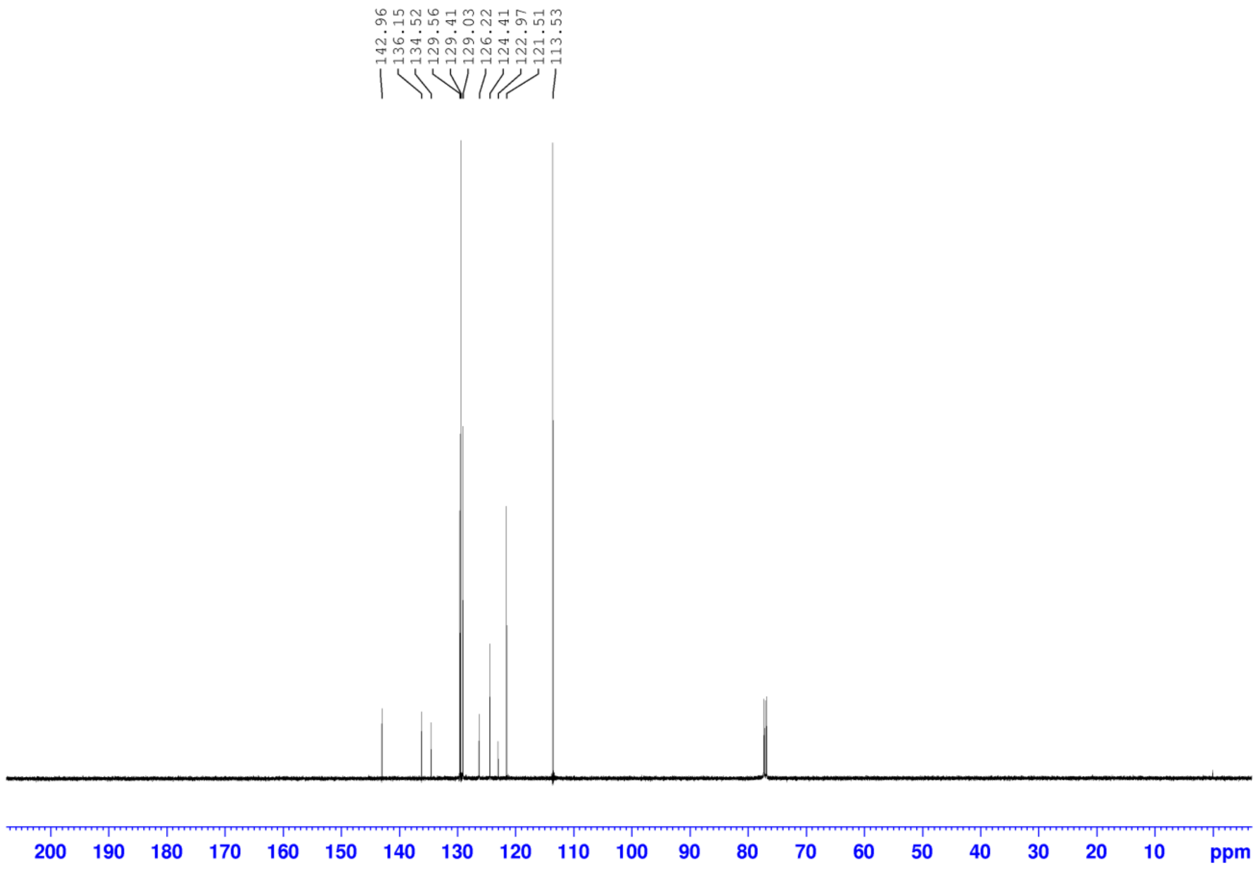
142.96
136.15
134.52
129.56
129.41
129.03
126.22
124.41
122.97
121.51
113.53
200 190 180 170 160 150 140 130 120 110 100 90 80 70 60 50 40 30 20 10 ppm

4-chloro-N-phenylbenzohydrazonyl chloride (4g)

^{1}H NMR

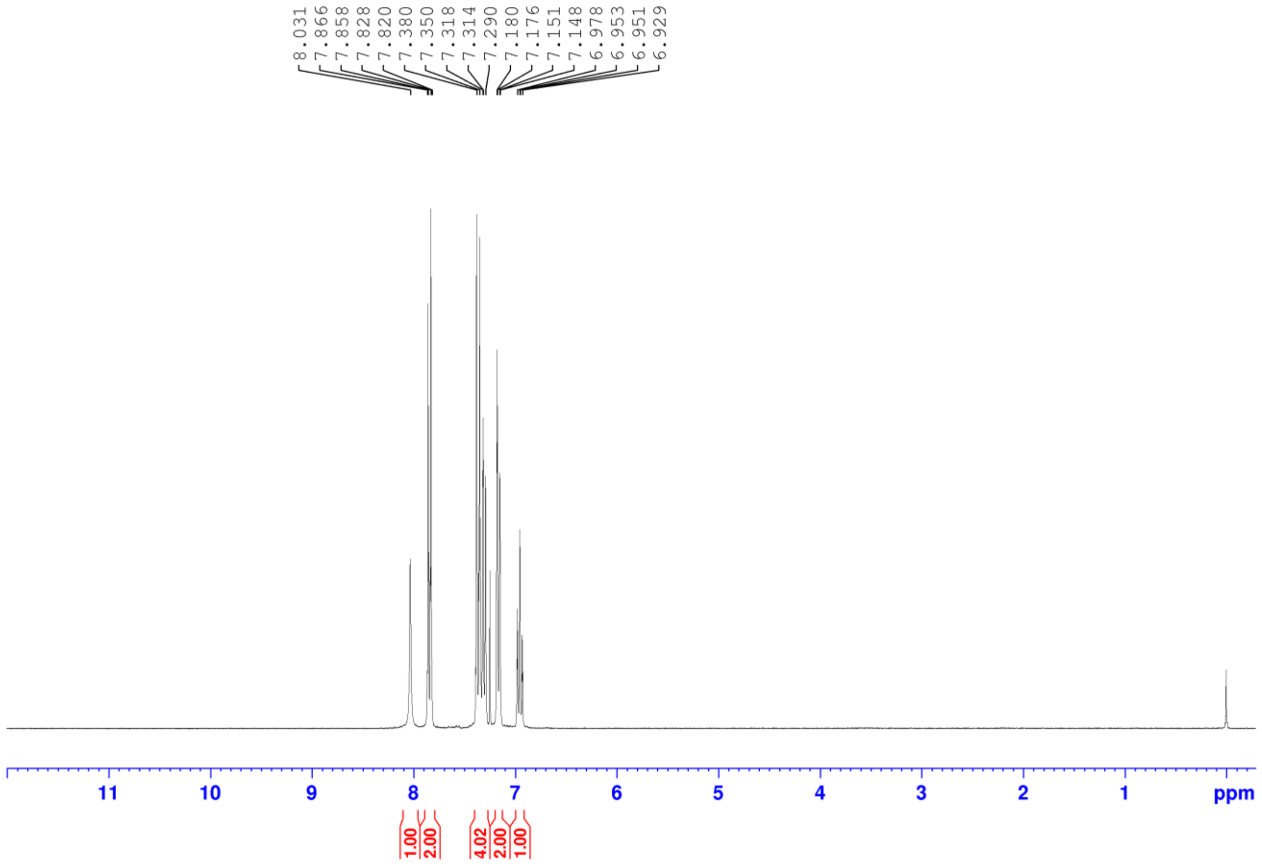

^{13}C NMR

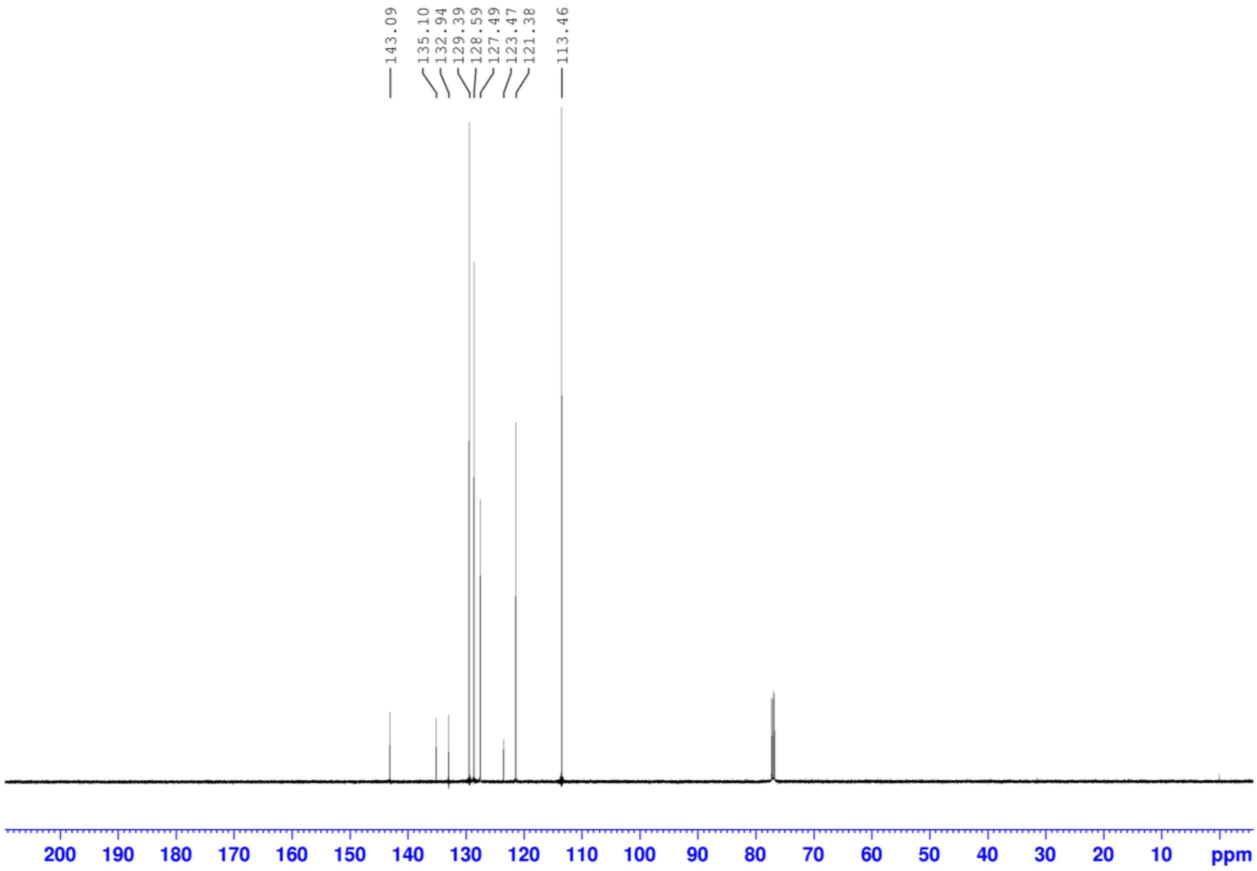
143.09
135.10
132.94
129.39
128.59
127.49
123.47
121.38
113.46
200 190 180 170 160 150 140 130 120 110 100 90 80 70 60 50 40 30 20 10 ppm

Characterization spectra of 1,3,4,5-tetrasubstituted pyrazoles

1-(5-Methyl-1,3-diphenyl-1*H*-pyrazol-4-yl)ethanone (6a)

^{1}H NMR

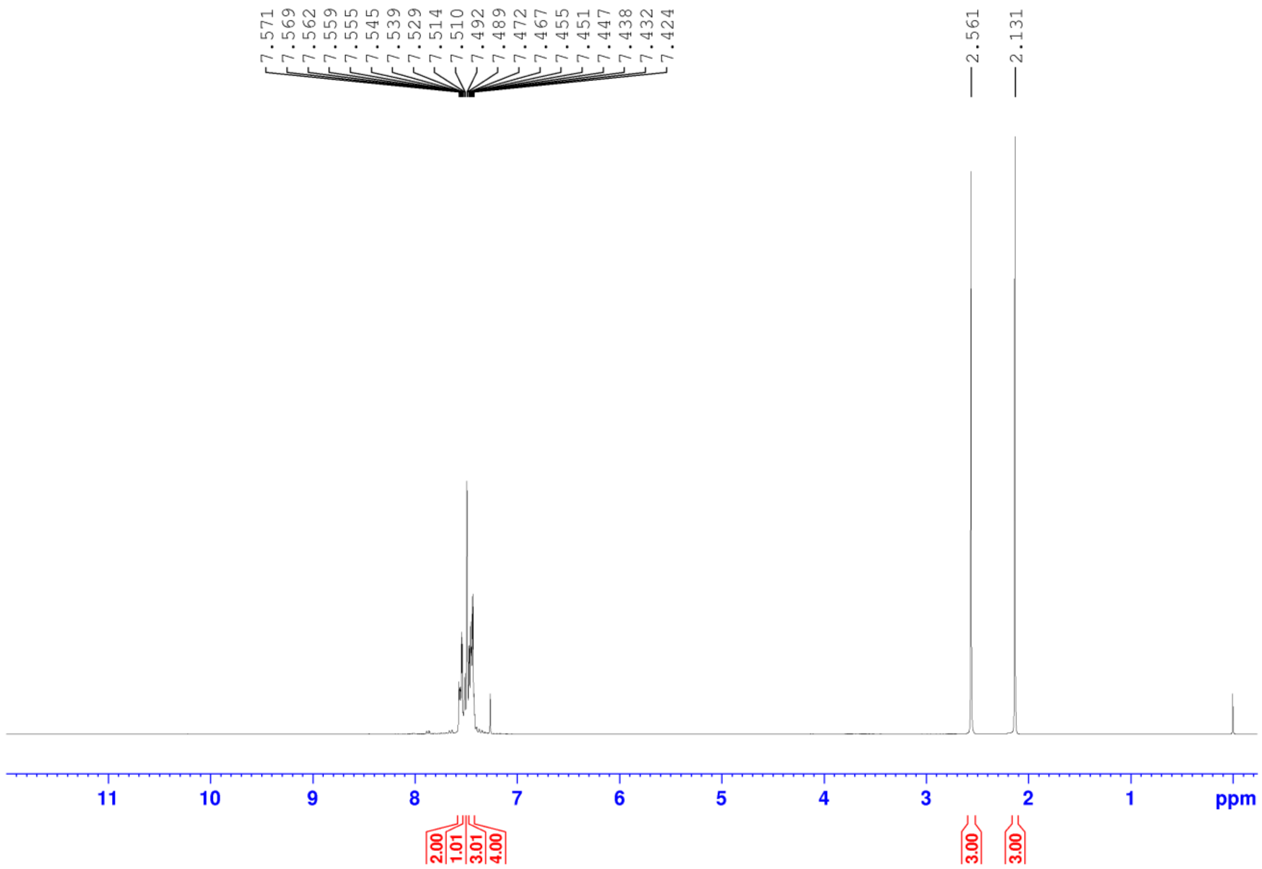

^{13}C NMR

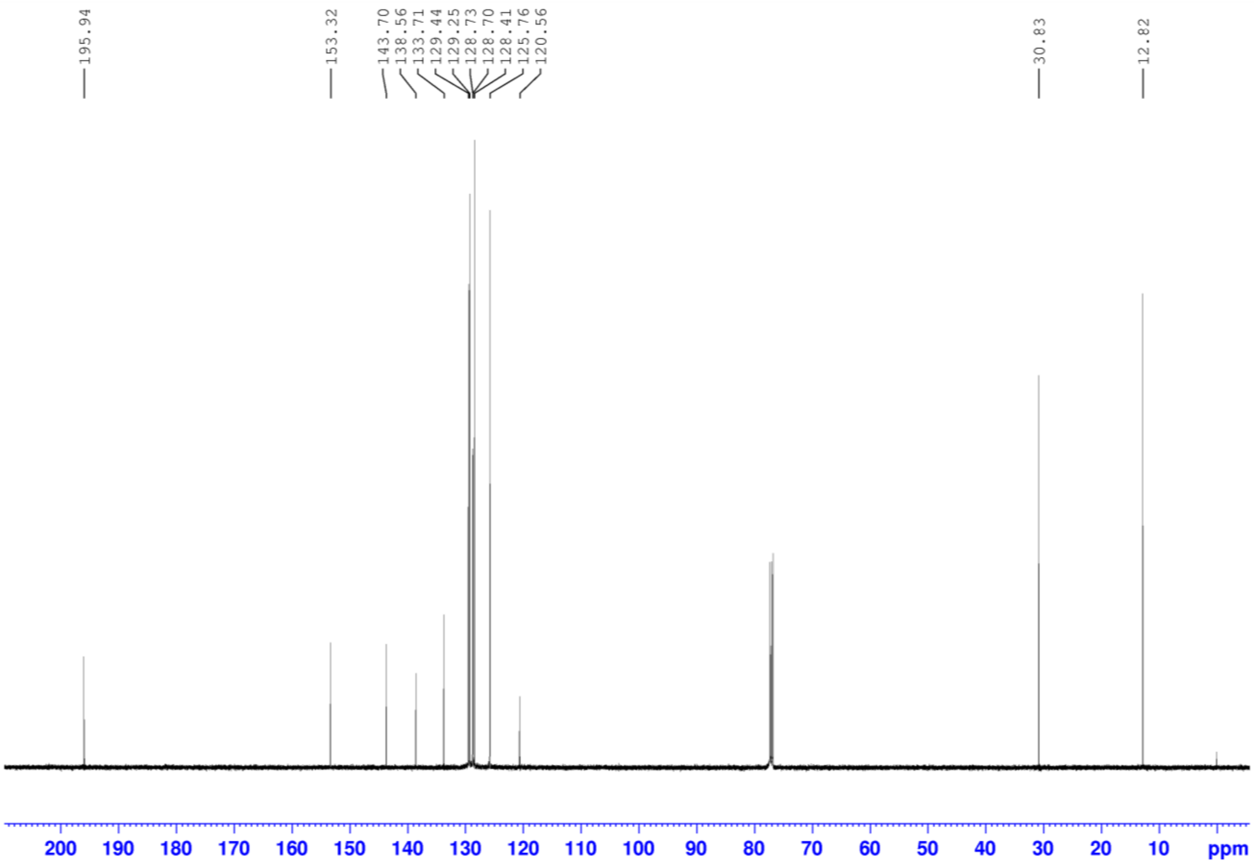

195.94
153.32
143.70
138.56
133.71
129.44
129.25
128.73
128.70
128.41
125.76
120.56
30.83
12.82
200
190
180
170
160
150
140
130
120
110
100
90
80
70
60
50
40
30
20
10
ppm

COSY NMR

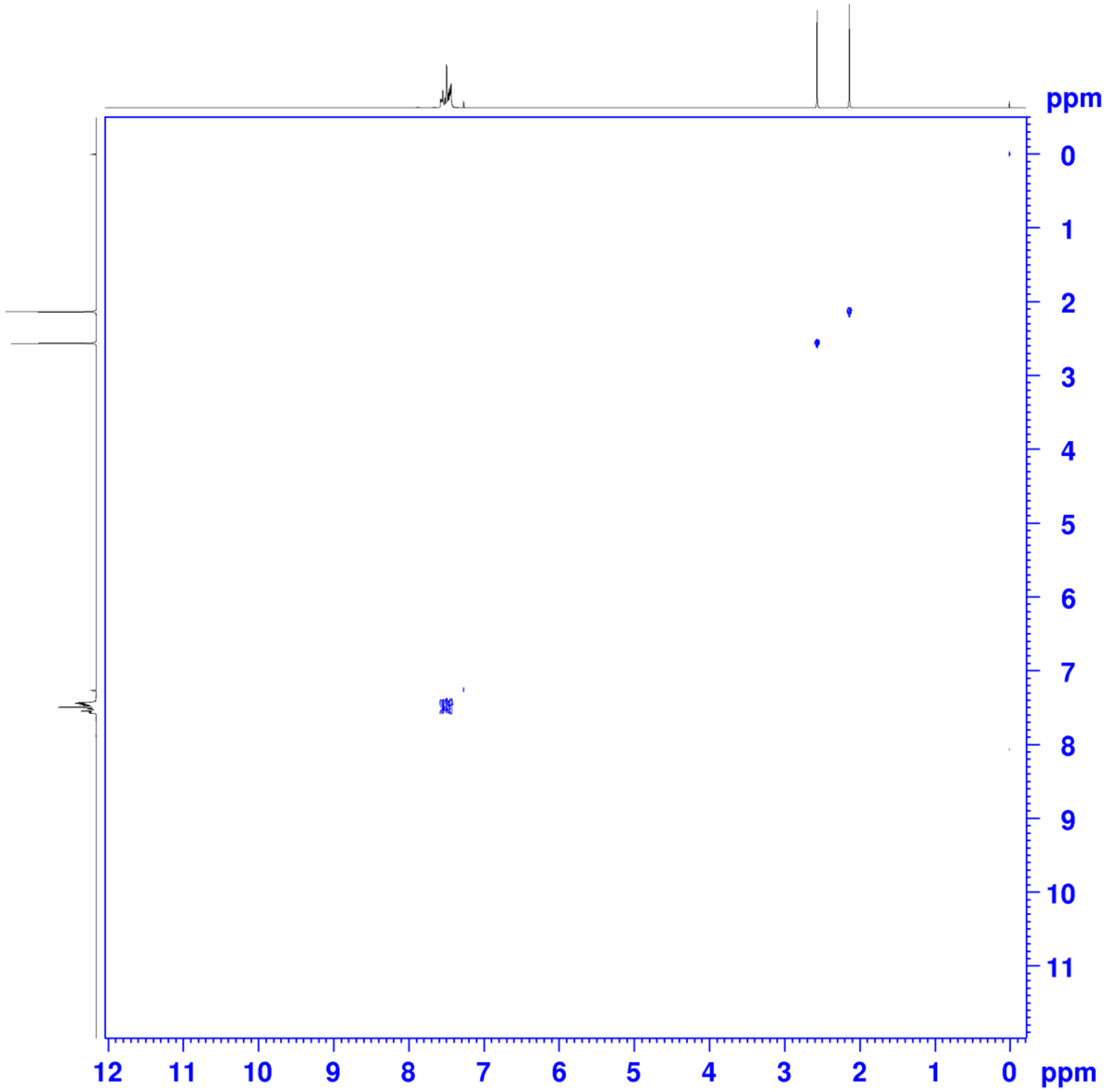

HRMS

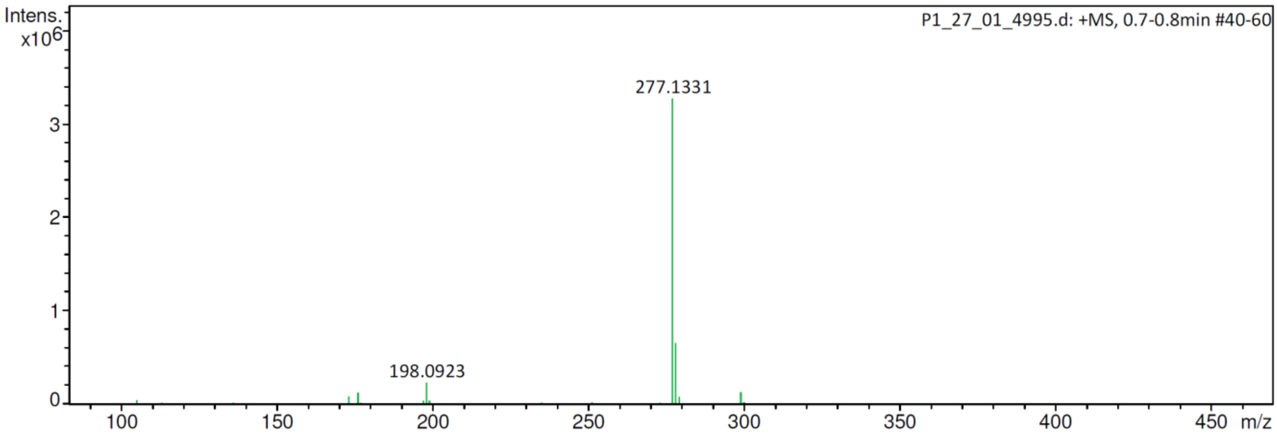

Intens.
x10^6
P1_27_01_4995.d: +MS, 0.7-0.8min #40-60
277.1331
198.0923
3
2
1
0
100
150
200
250
300
350
400
450
m/z

1-(3-(4-Methoxyphenyl)-5-methyl-1-phenyl-1*H*-pyrazol-4-yl)ethanone (6b)

^{1}H NMR

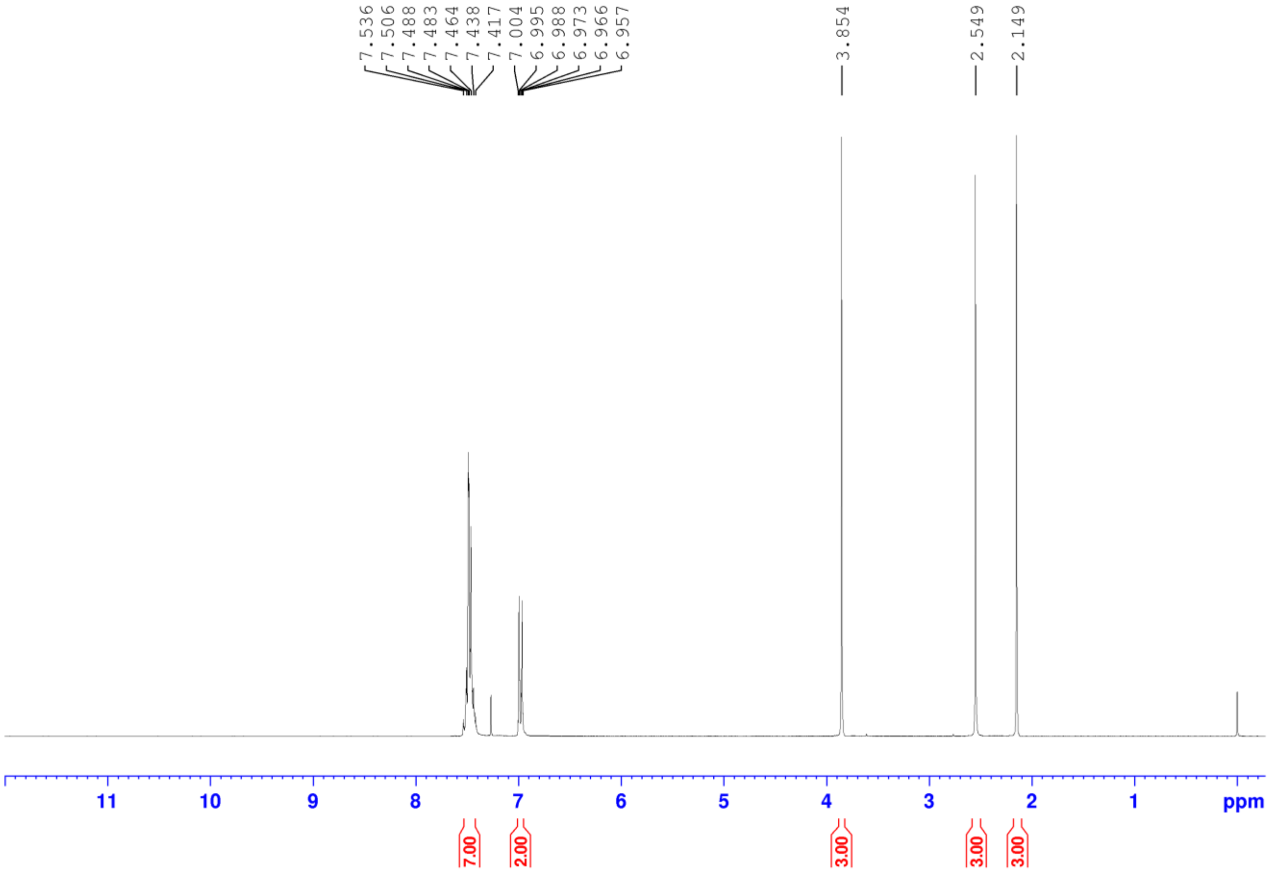

^{13}C NMR

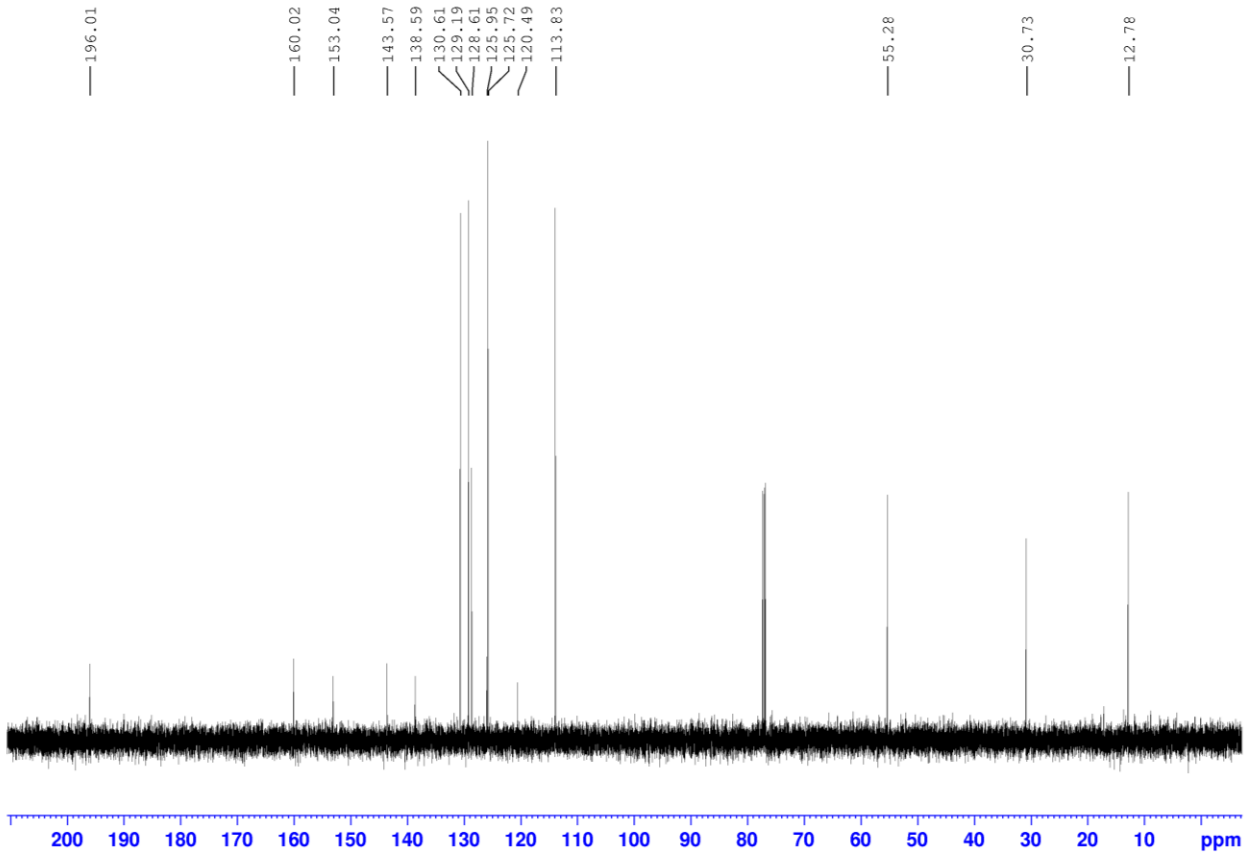
196.01
160.02
153.04
143.57
138.59
130.61
129.19
128.61
125.95
125.72
120.49
113.83
55.28
30.73
12.78
200
190
180
170
160
150
140
130
120
110
100
90
80
70
60
50
40
30
20
10
ppm

COSY NMR

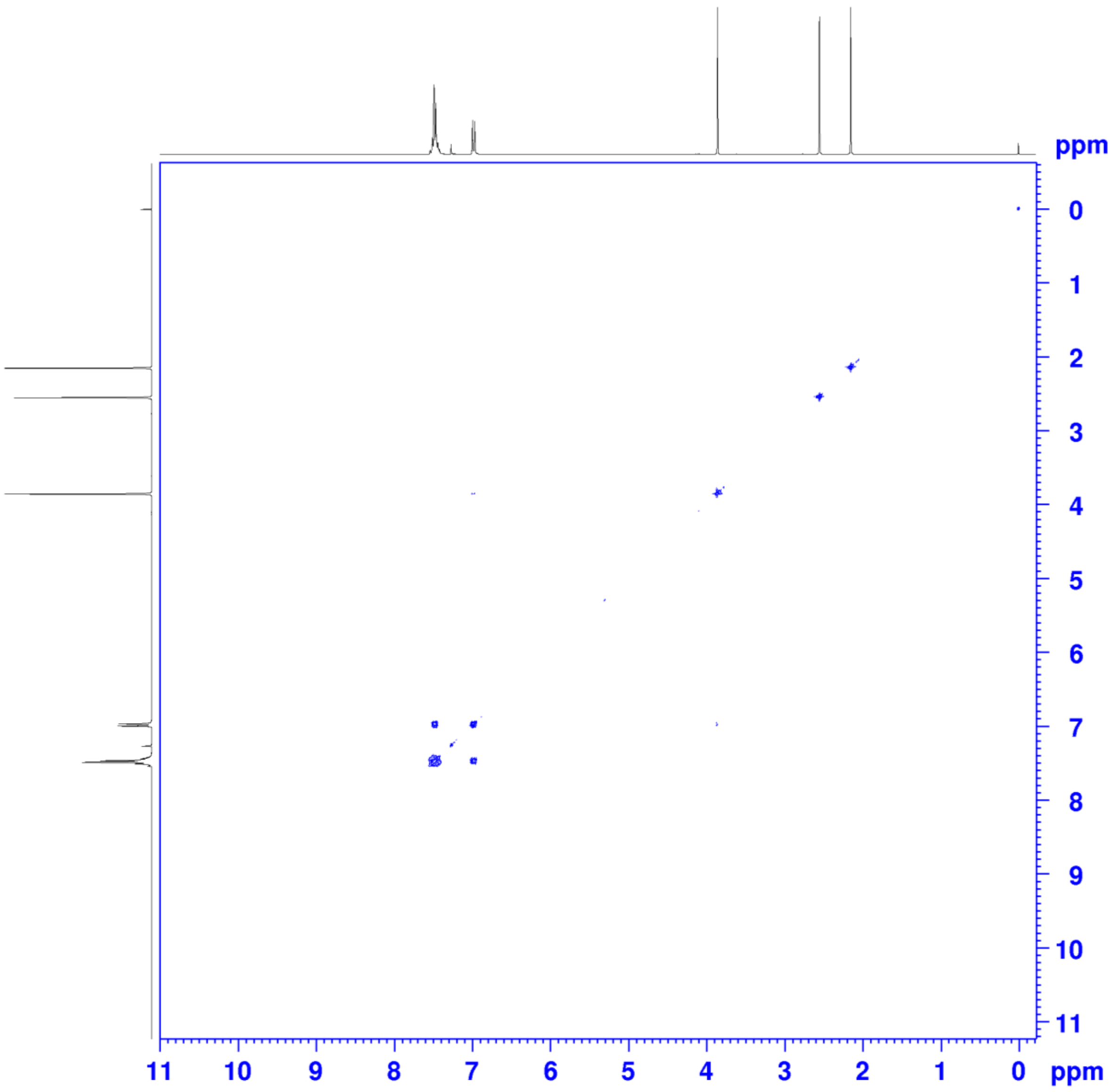

HRMS

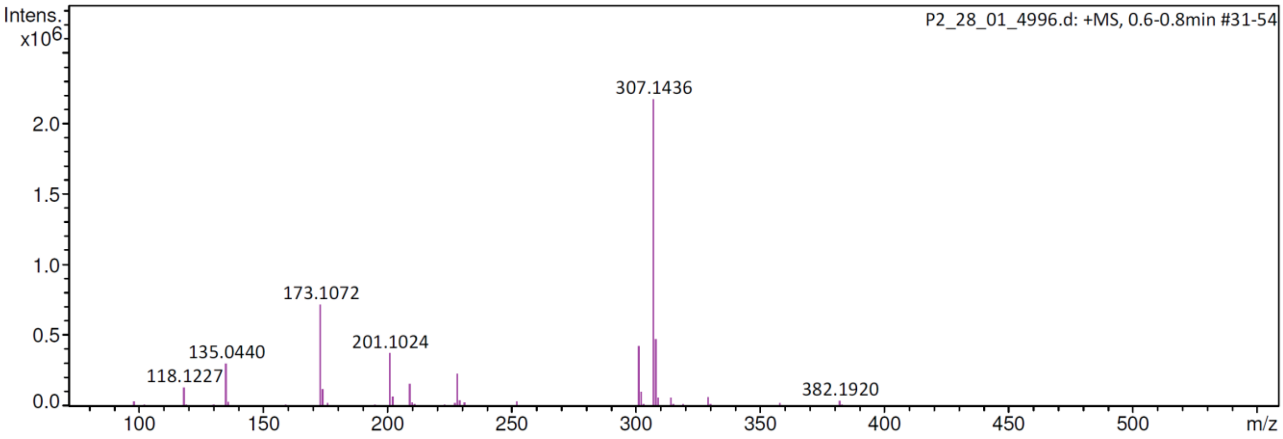

Intens.
x10^6
P2_28_01_4996.d: +MS, 0.6-0.8min #31-54
307.1436
173.1072
201.1024
135.0440
118.1227
382.1920
2.0
1.5
1.0
0.5
0.0
100
150
200
250
300
350
400
450
500
m/z

1-(5-Methyl-1-phenyl-3-(*p*-tolyl)-1*H*-pyrazol-4-yl)ethanone (6c)

^{1}H NMR

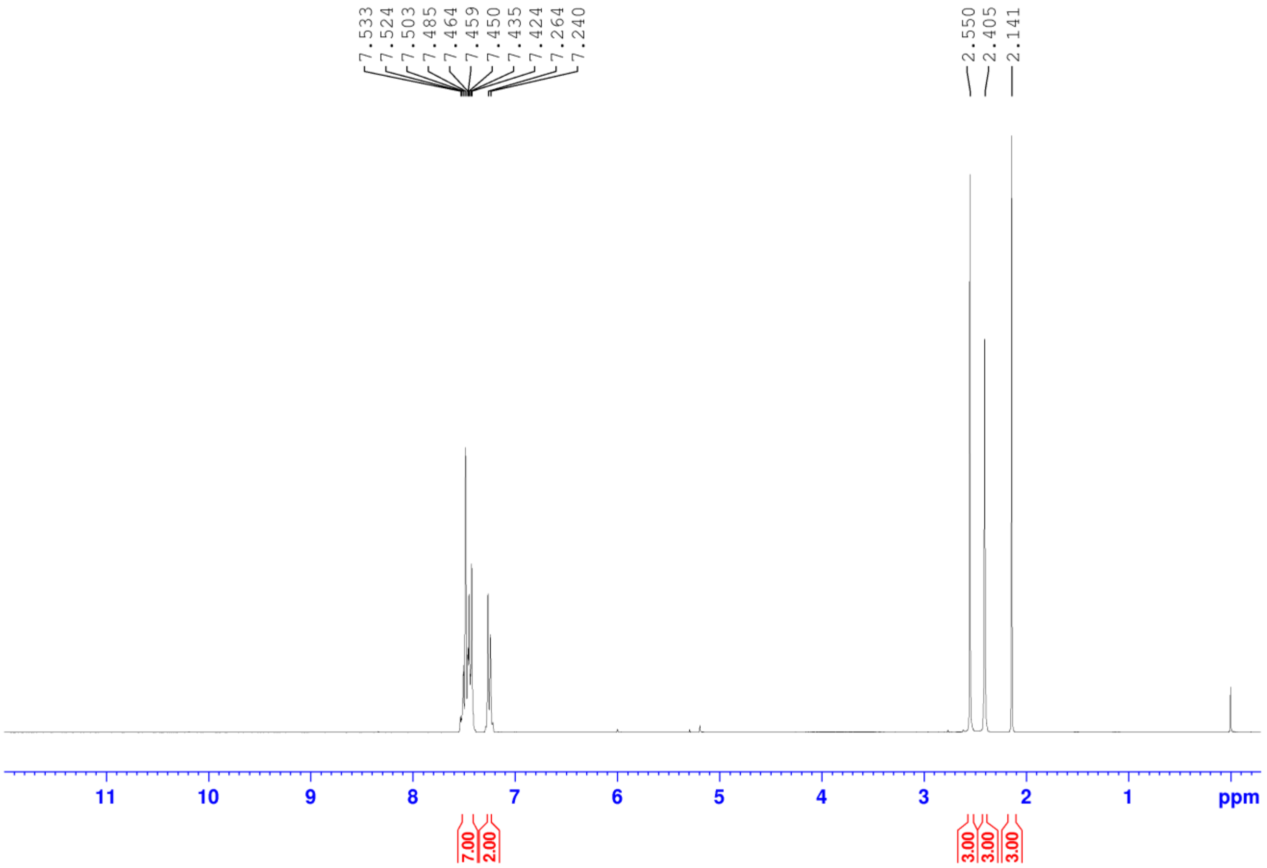

^{13}C NMR

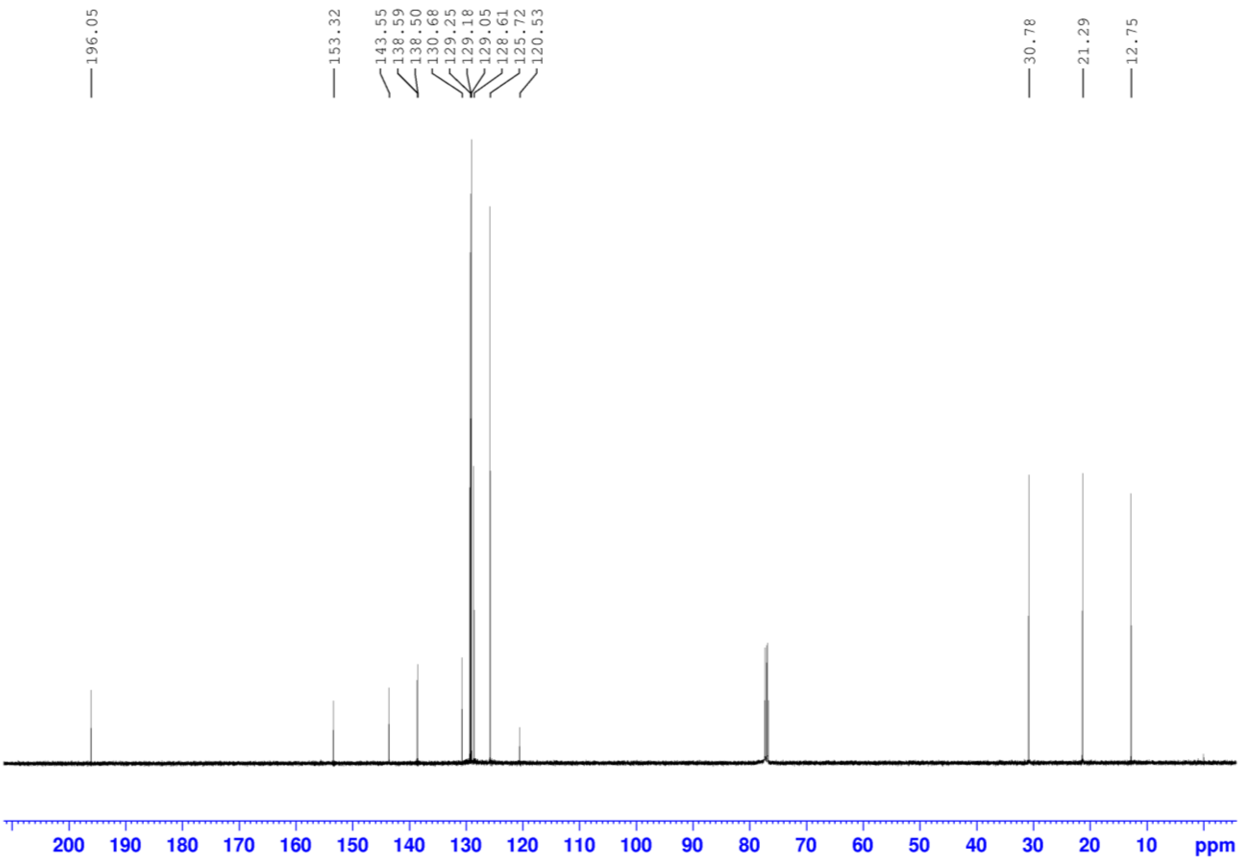

196.05
153.32
143.55
138.59
138.50
130.68
129.25
129.18
129.05
128.61
125.72
120.53
30.78
21.29
12.75
200
190
180
170
160
150
140
130
120
110
100
90
80
70
60
50
40
30
20
10
ppm

COSY NMR

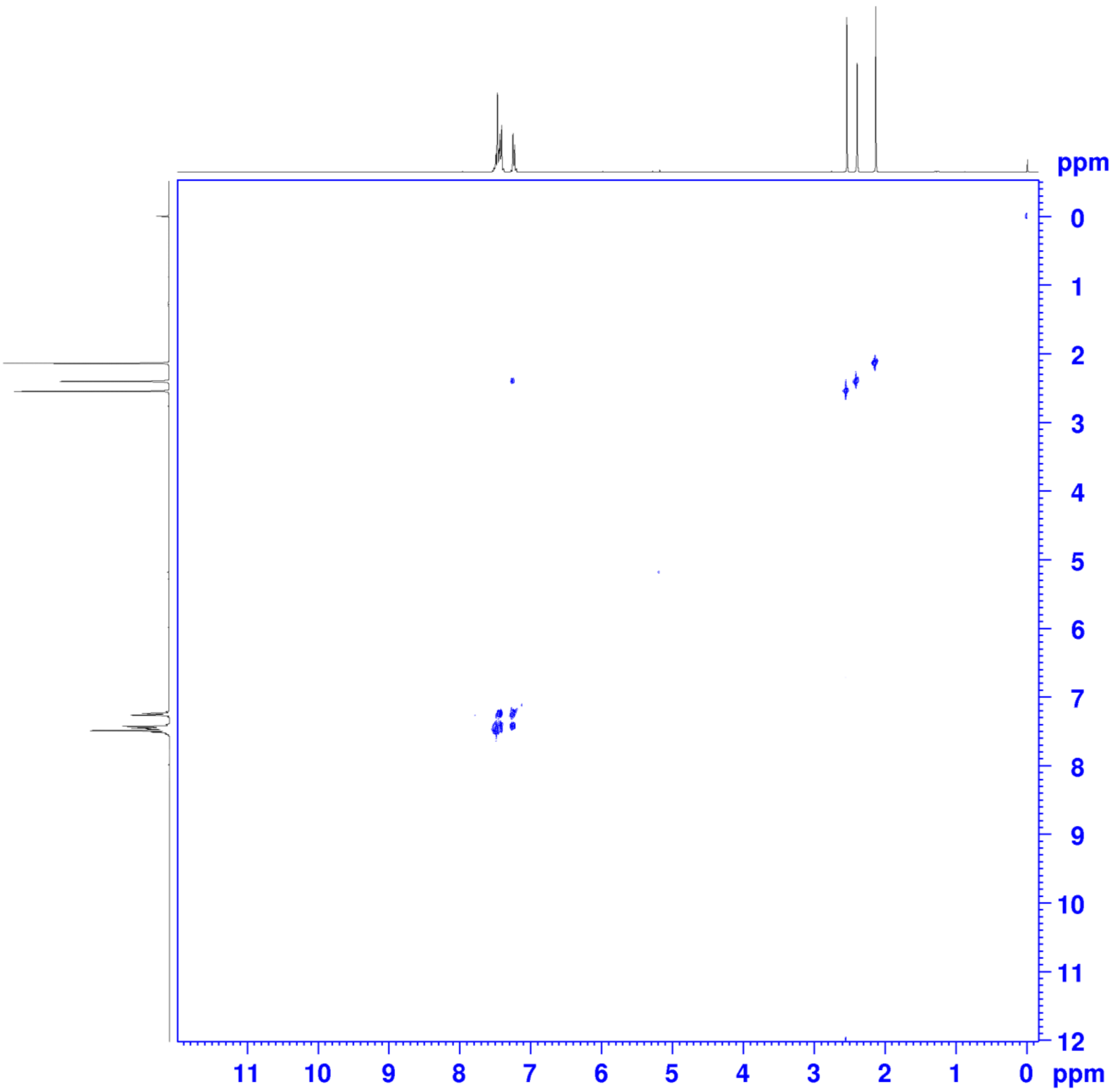

HRMS

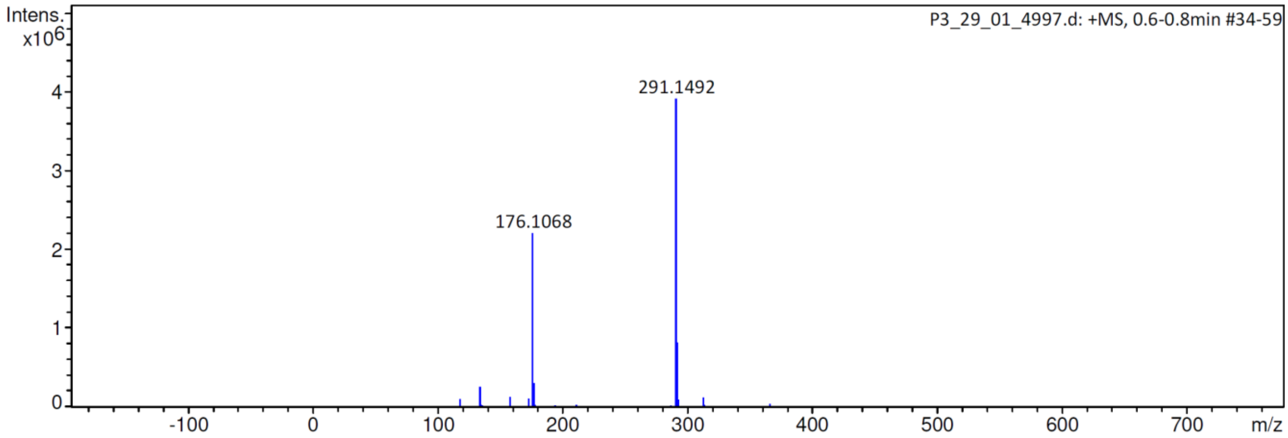

Intens.
x10^6
P3_29_01_4997.d: +MS, 0.6-0.8min #34-59
291.1492
176.1068
m/z

1-(5-Methyl-3-(4-nitrophenyl)-1-phenyl-1*H*-pyrazol-4-yl)ethanone (6d)

^{1}H NMR

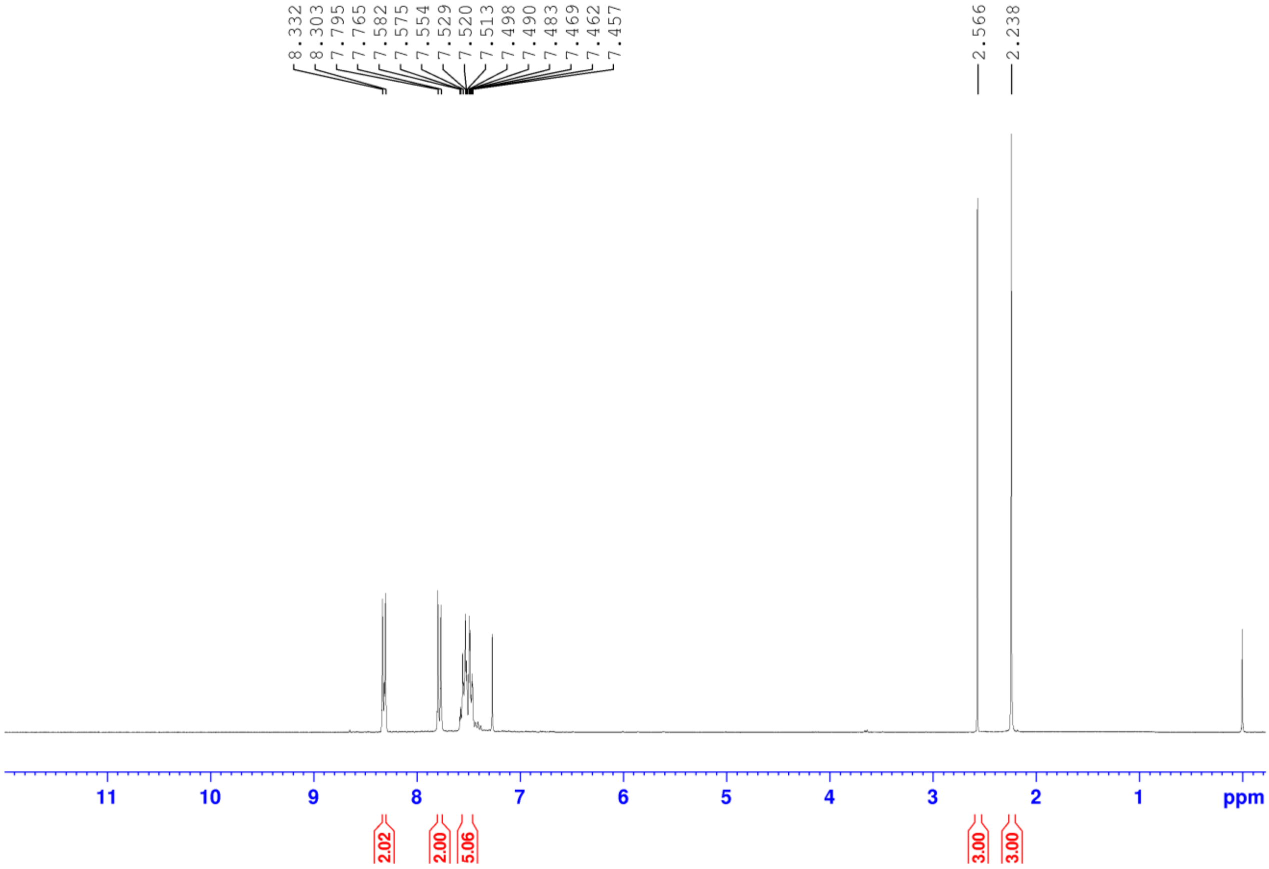

^{13}C NMR

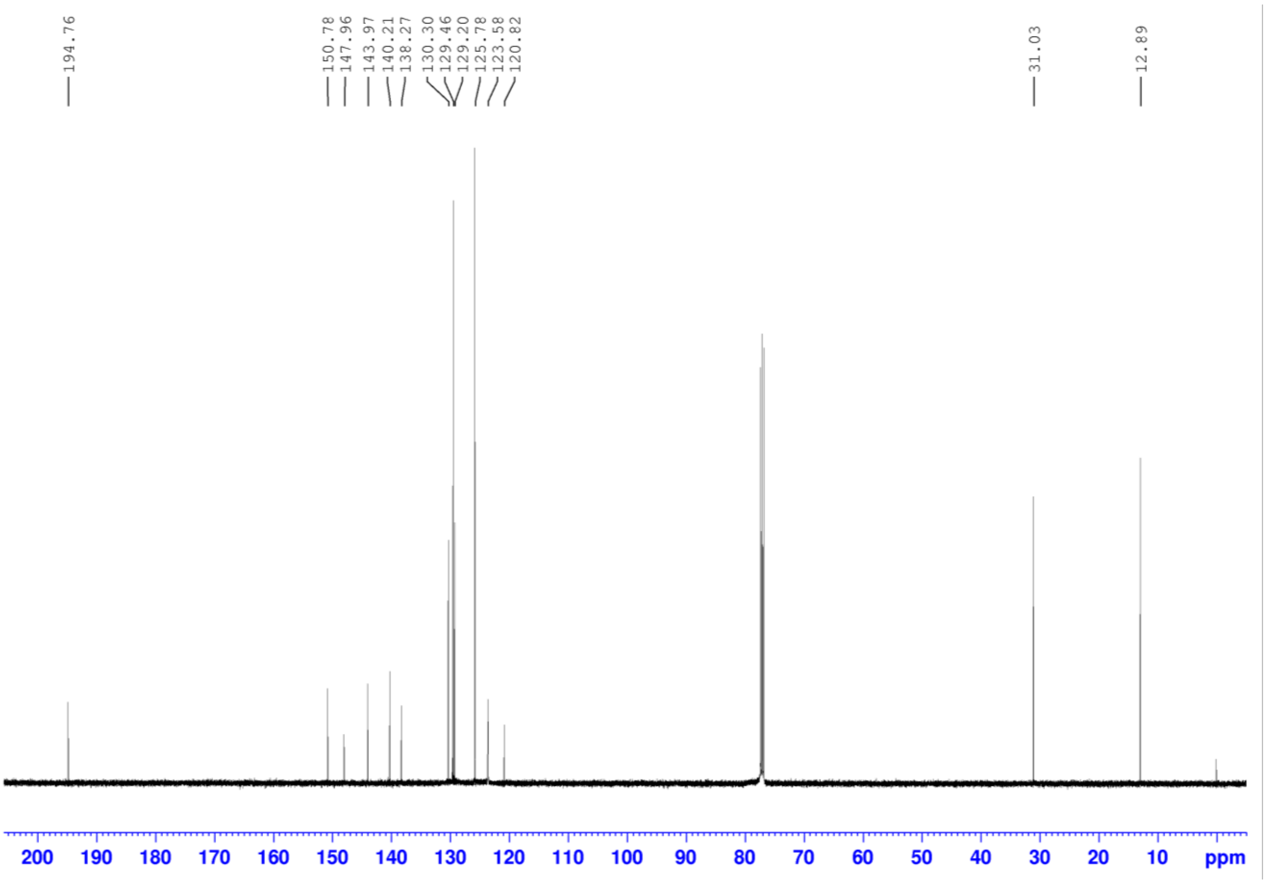
194.76
150.78
147.96
143.97
140.21
138.27
130.30
129.46
129.20
125.78
123.58
120.82
31.03
12.89
200 190 180 170 160 150 140 130 120 110 100 90 80 70 60 50 40 30 20 10 ppm

COSY NMR

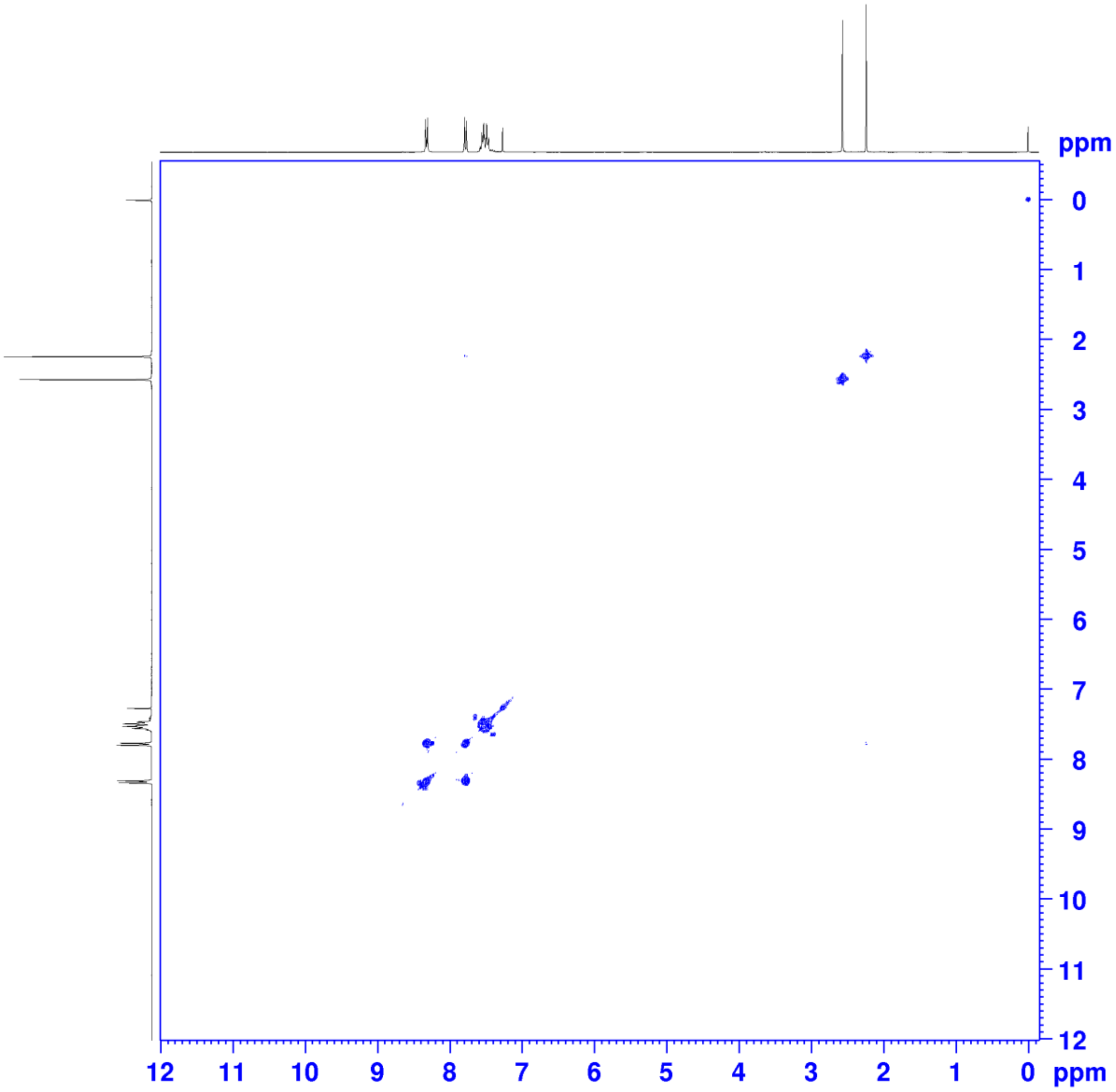

HRMS

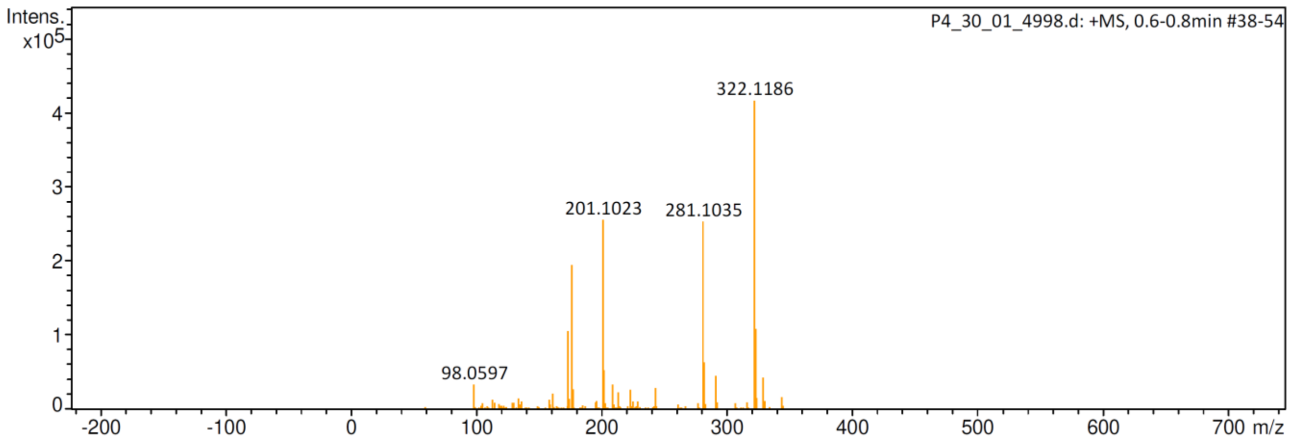
Intens.
x10^5
P4_30_01_4998.d: +MS, 0.6-0.8min #38-54
322.1186
201.1023
281.1035
98.0597
0
1
2
3
4
-200
-100
0
100
200
300
400
500
600
700 m/z

1-(3-(2-Chlorophenyl)-5-methyl-1-phenyl-1*H*-pyrazol-4-yl)ethanone (6e)

^{1}H NMR

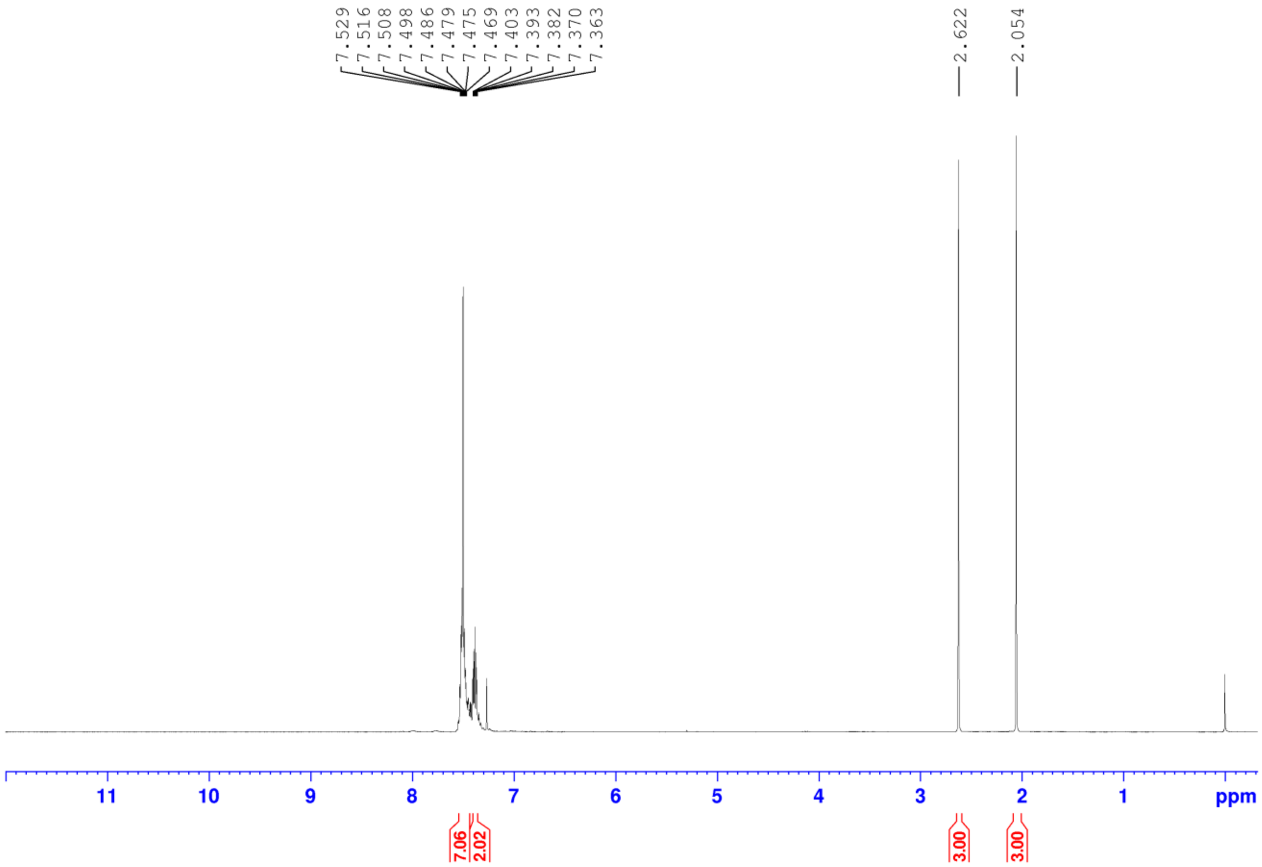

^{13}C NMR

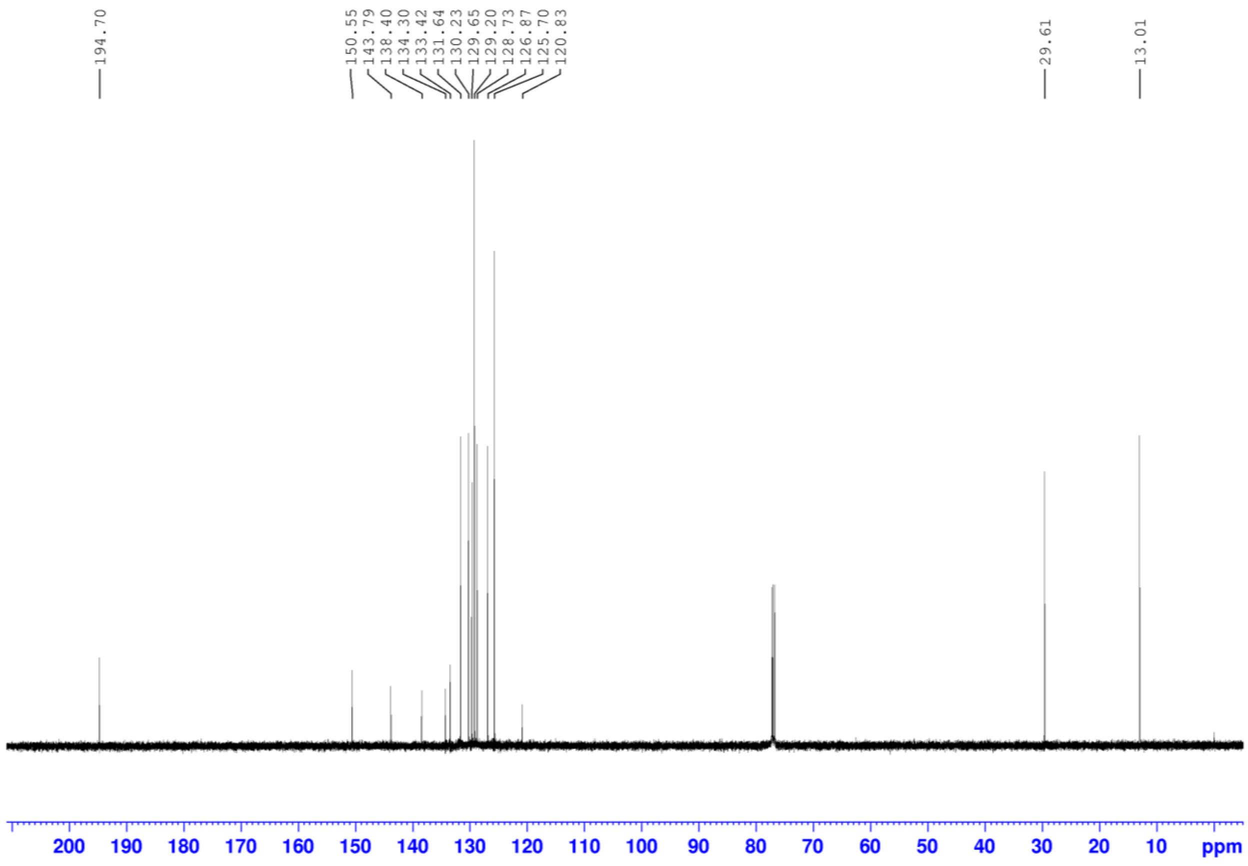

194.70
150.55
143.79
138.40
134.30
133.42
131.64
130.23
129.65
129.20
128.73
126.87
125.70
120.83
29.61
13.01
200
190
180
170
160
150
140
130
120
110
100
90
80
70
60
50
40
30
20
10
ppm

COSY NMR

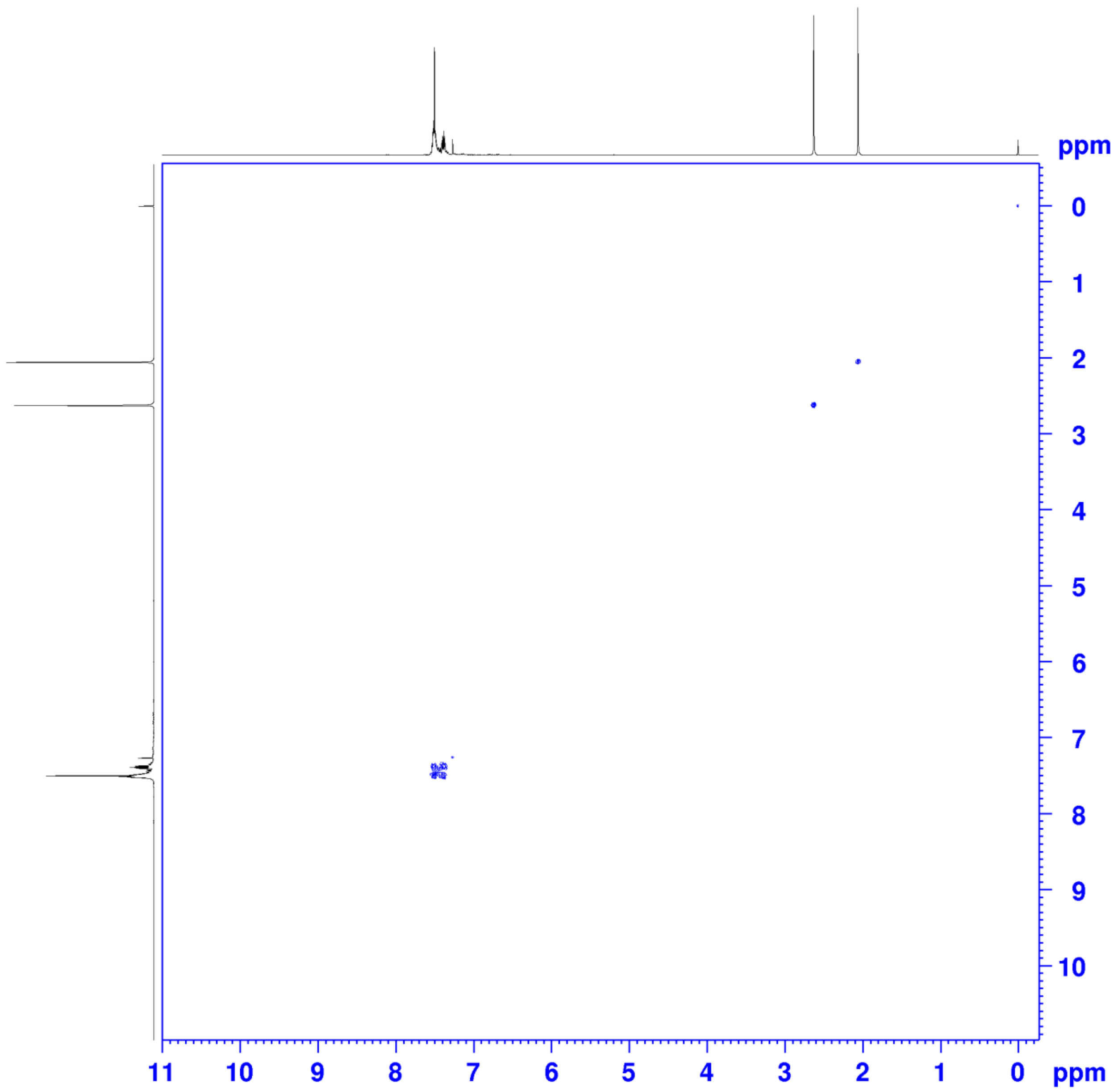

HRMS

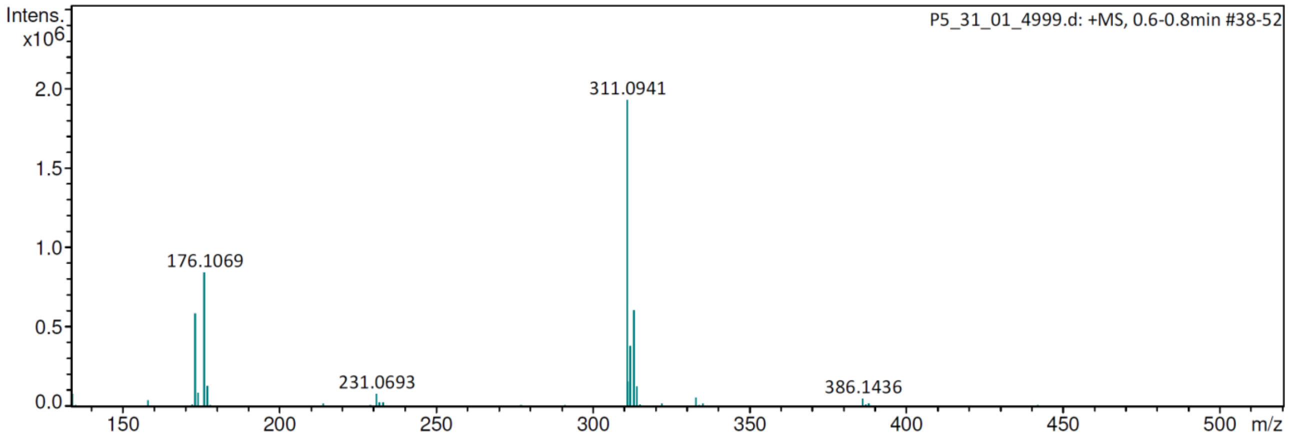

Intens.
x10^6
2.0
1.5
1.0
0.5
0.0
P5_31_01_4999.d: +MS, 0.6-0.8min #38-52
311.0941
176.1069
231.0693
386.1436
150
200
250
300
350
400
450
500
m/z

1-(3-(3-Chlorophenyl)-5-methyl-1-phenyl-1*H*-pyrazol-4-yl)ethanone (6f)

^{1}H NMR

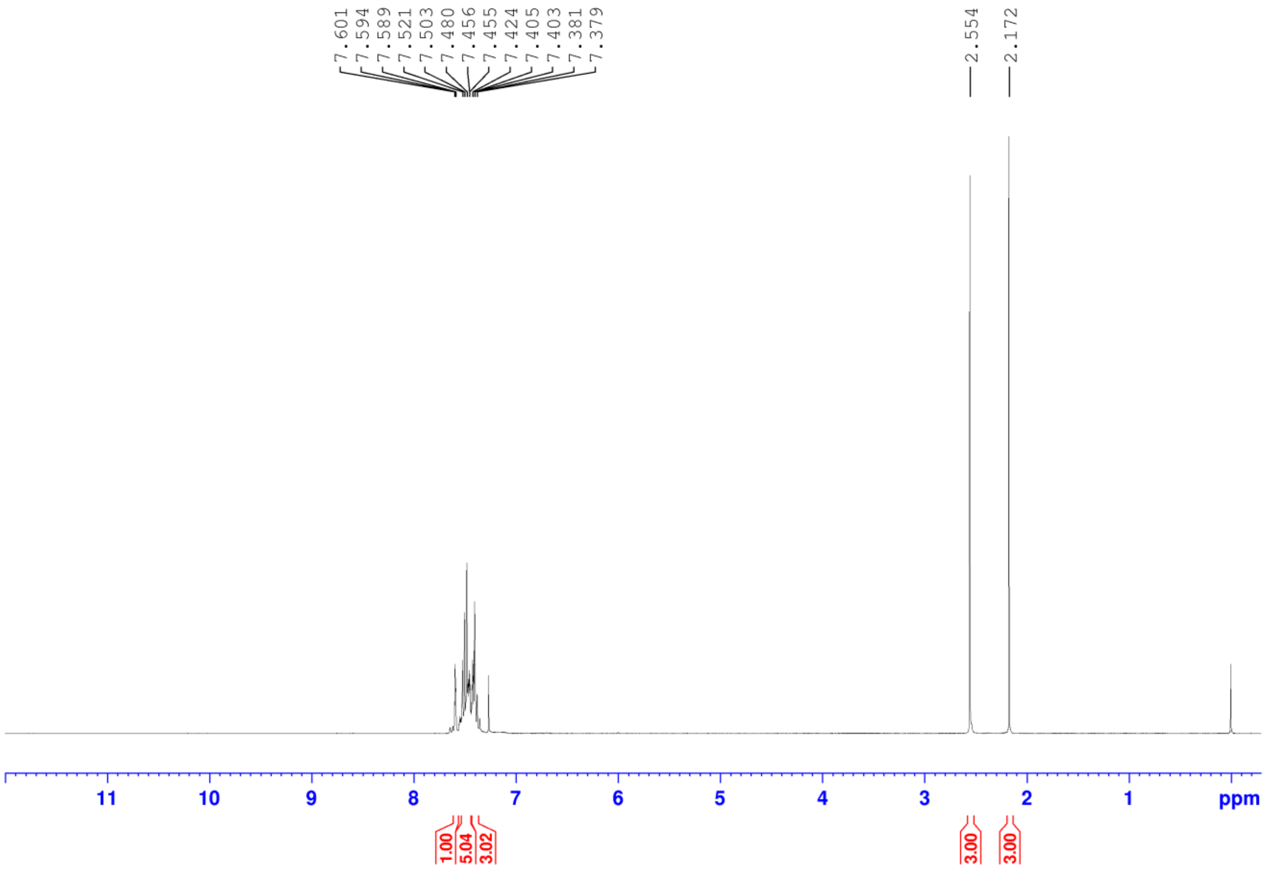

^{13}C NMR

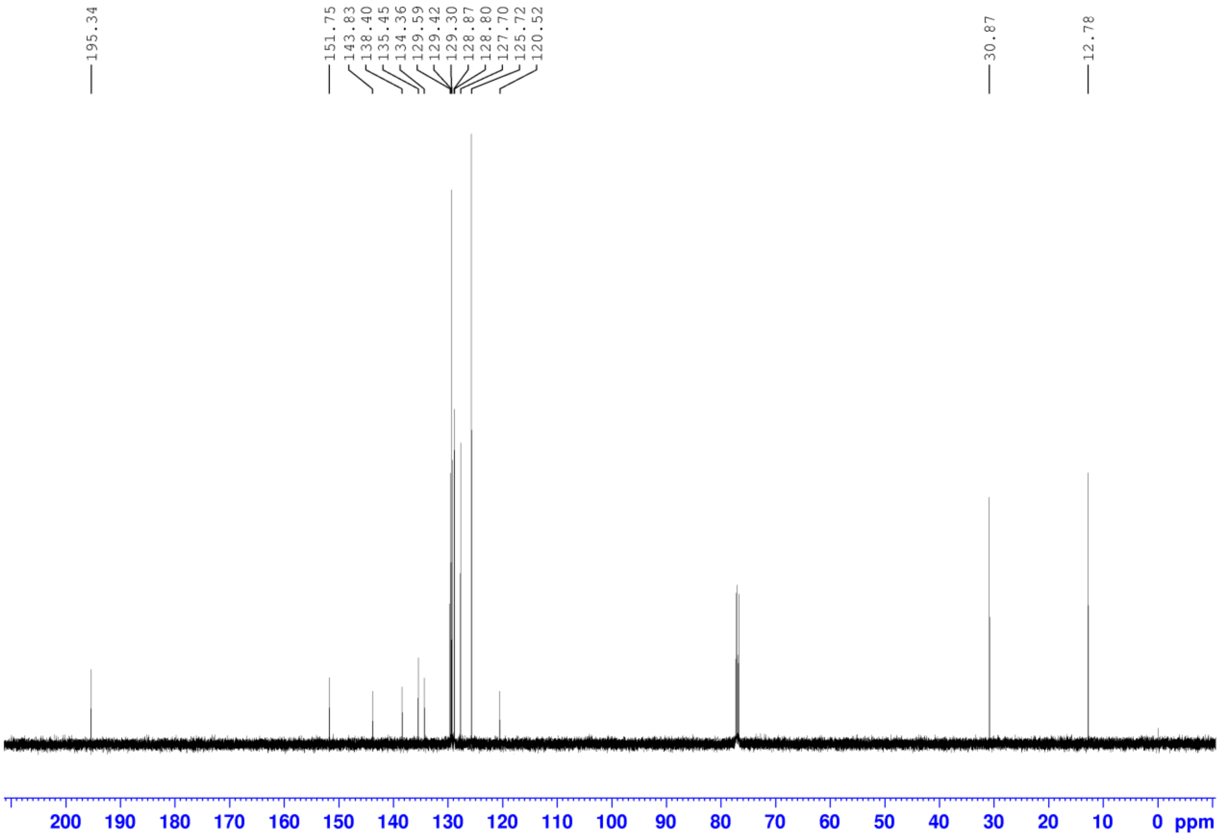
195.34
151.75
143.83
138.40
135.45
134.36
129.59
129.42
129.30
128.87
128.80
127.70
125.72
120.52
30.87
12.78
200 190 180 170 160 150 140 130 120 110 100 90 80 70 60 50 40 30 20 10 0 ppm

COSY NMR

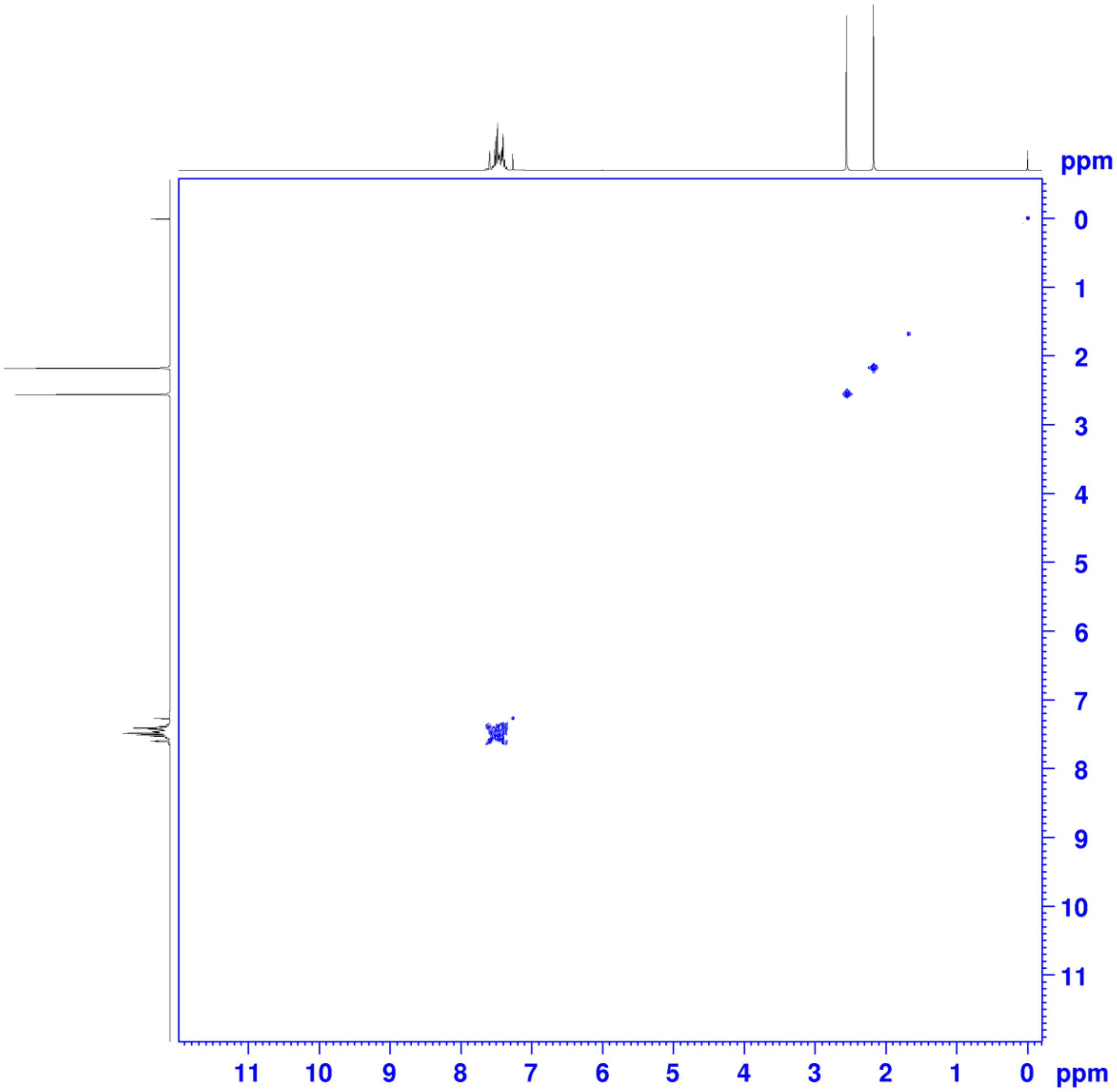

HRMS

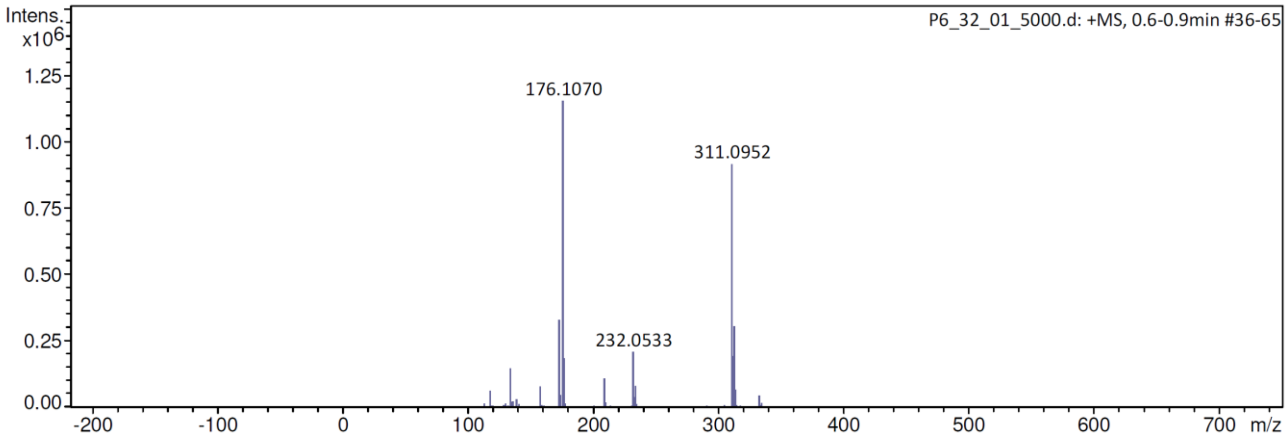
Intens.
x10^6
P6_32_01_5000.d: +MS, 0.6-0.9min #36-65
1.25
1.00
0.75
0.50
0.25
0.00
176.1070
311.0952
232.0533
-200
-100
0
100
200
300
400
500
600
700
m/z

1-(3-(4-Chlorophenyl)-5-methyl-1-phenyl-1*H*-pyrazol-4-yl)ethanone (6g)

^{1}H NMR

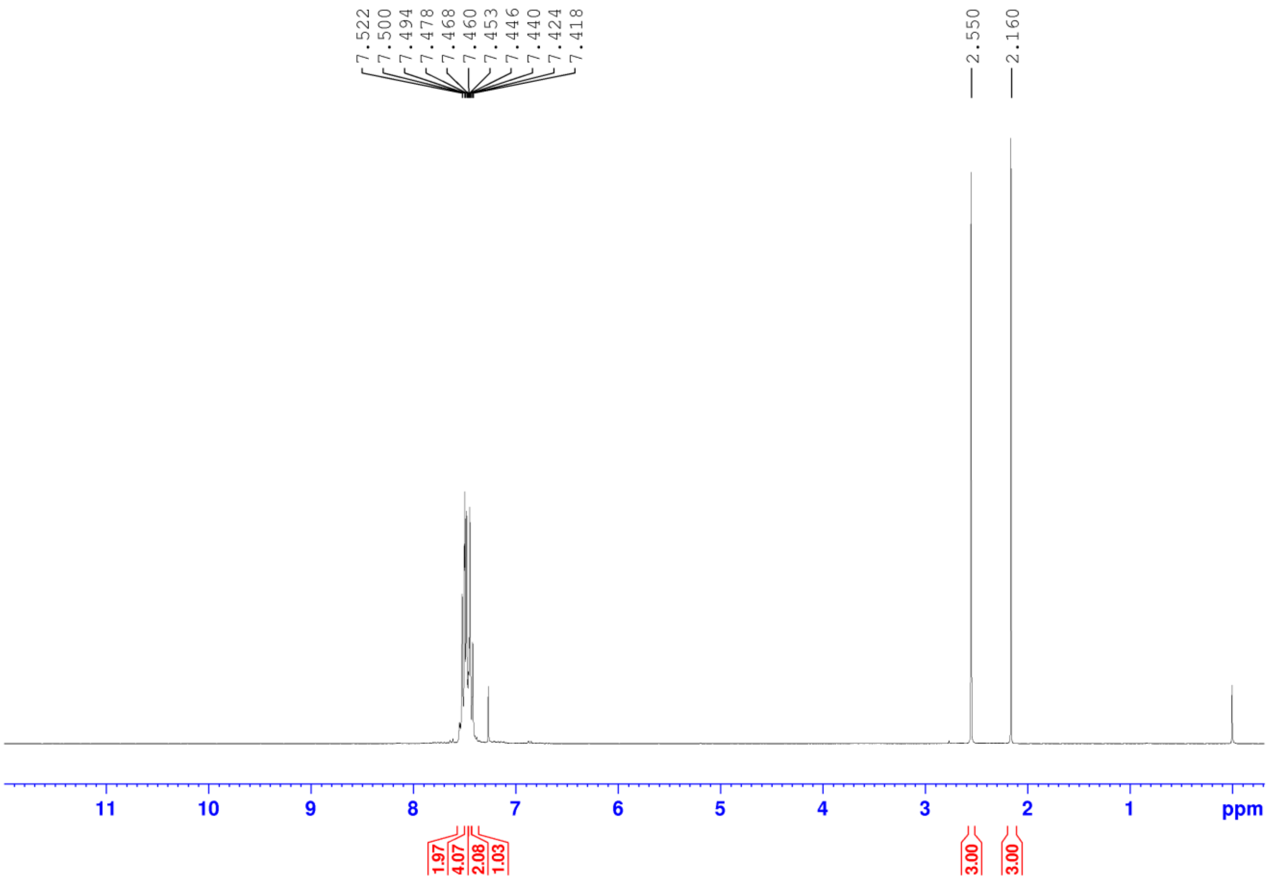

^{13}C NMR

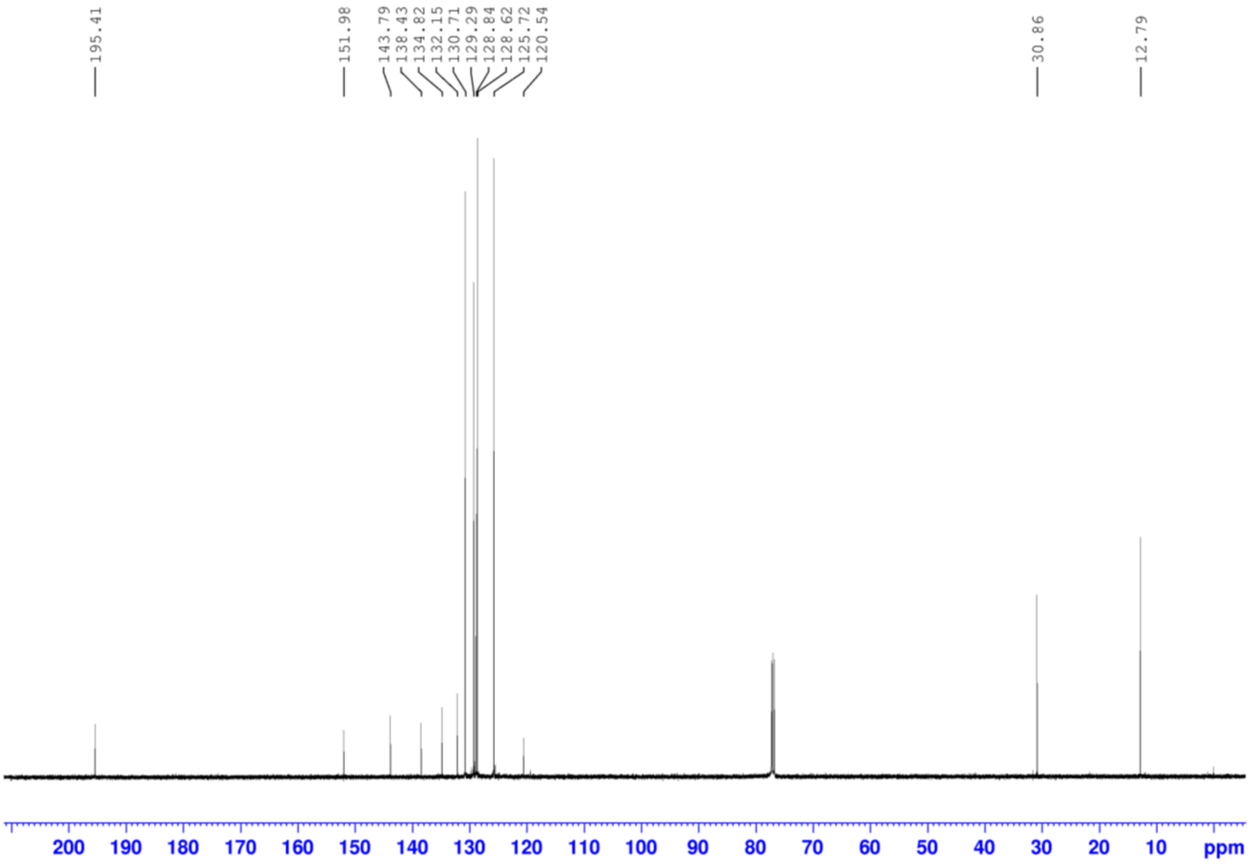
195.41
151.98
143.79
138.43
134.82
132.15
130.71
129.29
128.84
128.62
125.72
120.54
30.86
12.79
200 190 180 170 160 150 140 130 120 110 100 90 80 70 60 50 40 30 20 10 ppm

COSY NMR

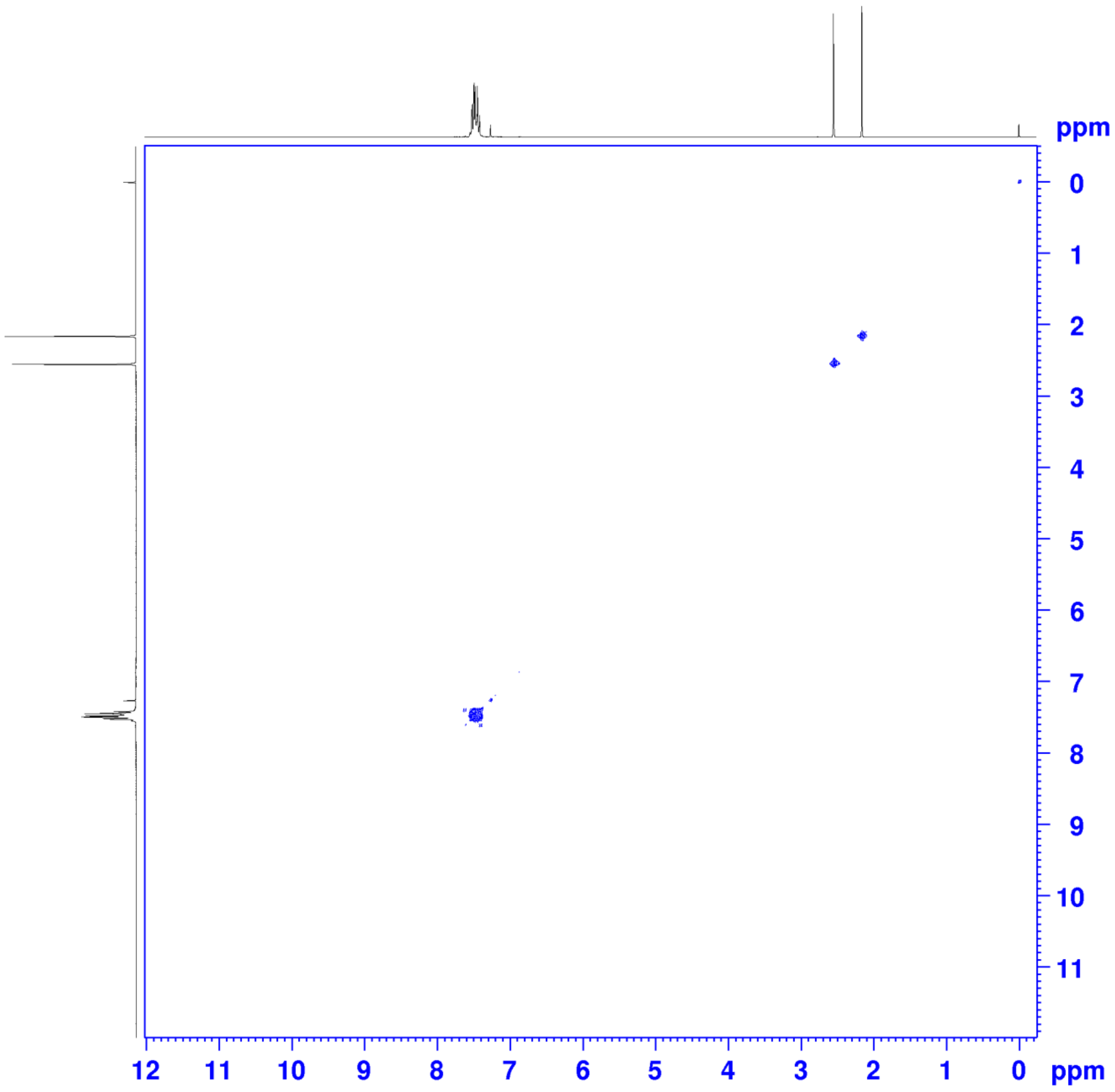

HRMS

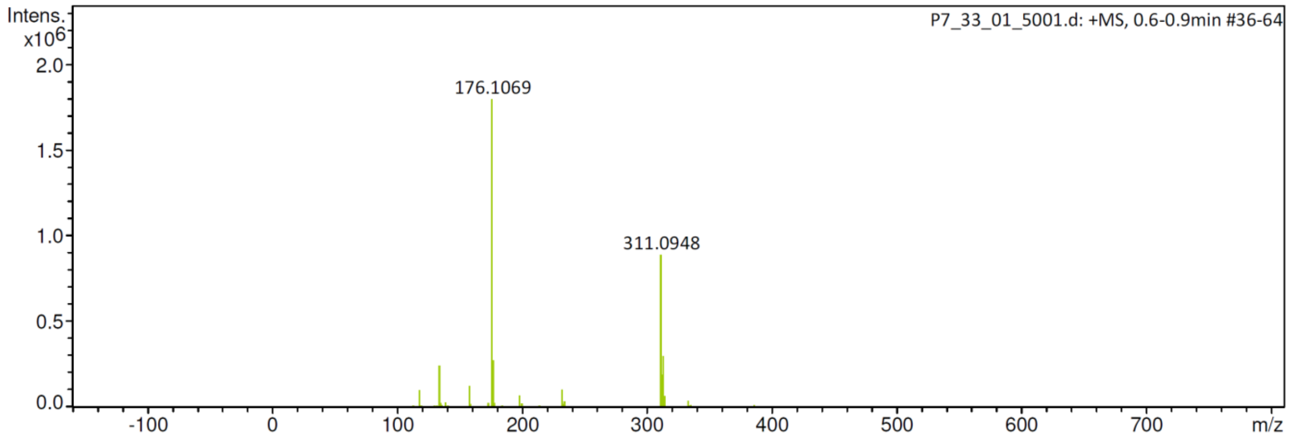

P7_33_01_5001.d: +MS, 0.6-0.9min #36-64
Intens.
x10^6
2.0
1.5
1.0
0.5
0.0
176.1069
311.0948
-100
0
100
200
300
400
500
600
700
m/z

(5-Methyl-1,3-diphenyl-1*H*-pyrazol-4-yl)(phenyl)methanone (6h)

^{1}H NMR

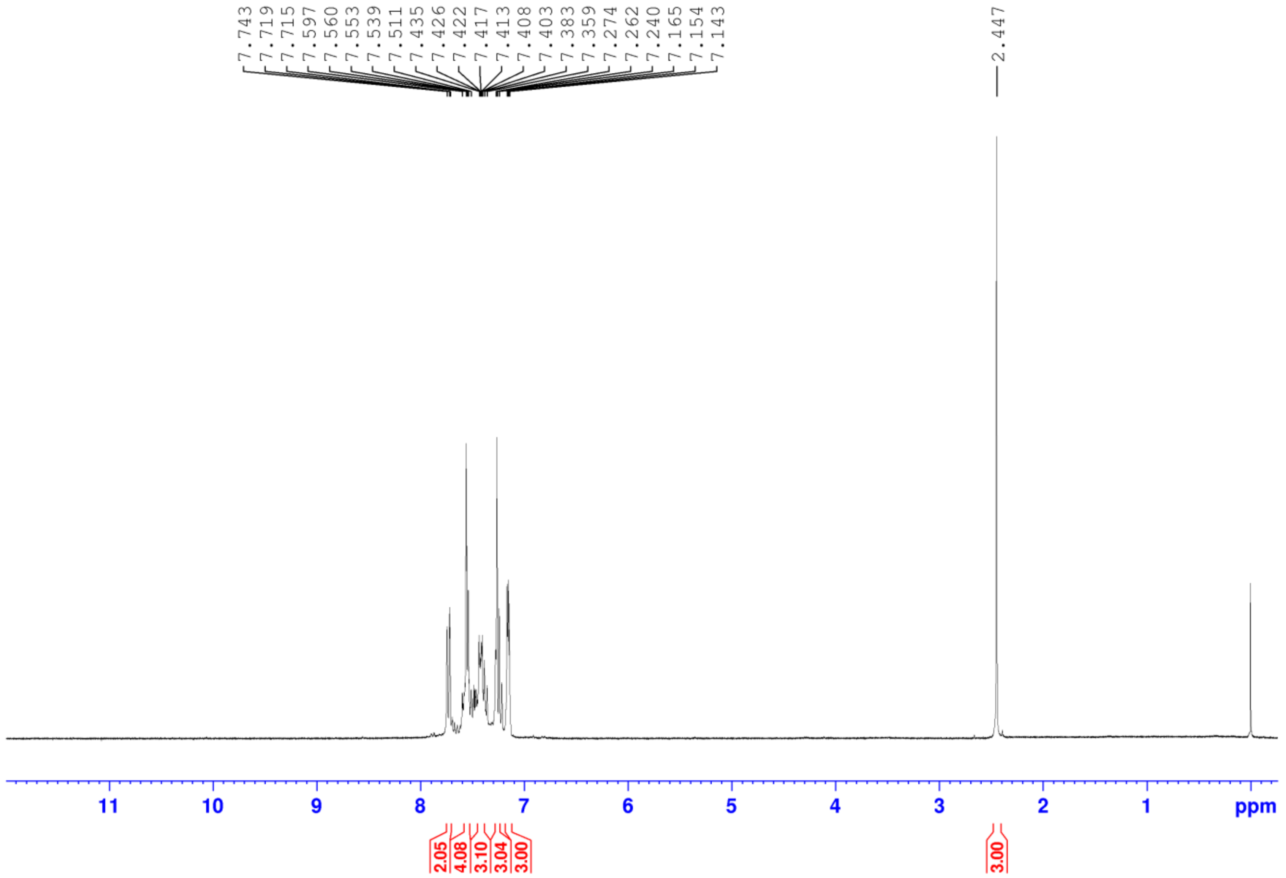

^{13}C NMR

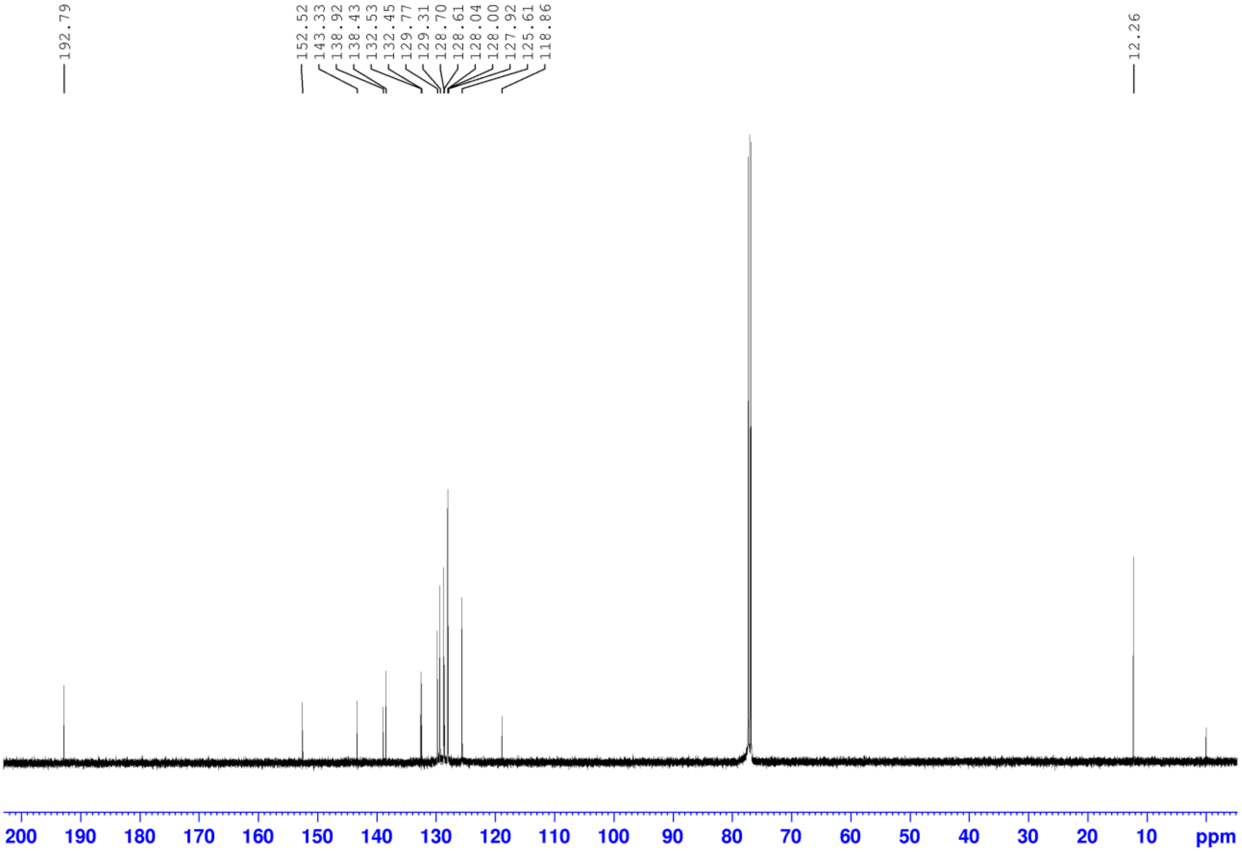

192.79
152.52
143.33
138.92
138.43
132.53
132.45
129.77
129.31
128.70
128.61
128.04
128.00
127.92
125.61
118.86
12.26
200
190
180
170
160
150
140
130
120
110
100
90
80
70
60
50
40
30
20
10
ppm

COSY NMR

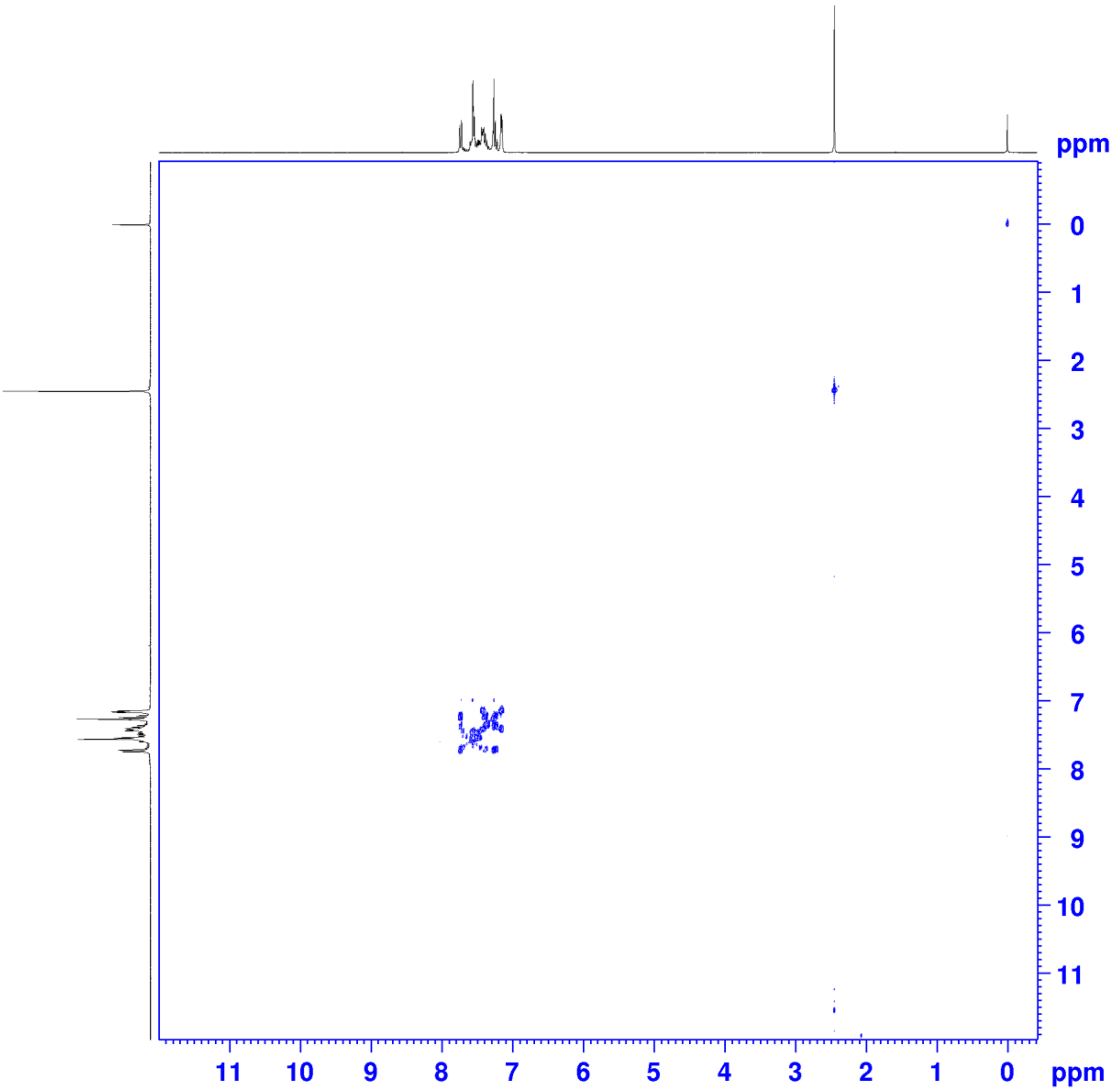

HRMS

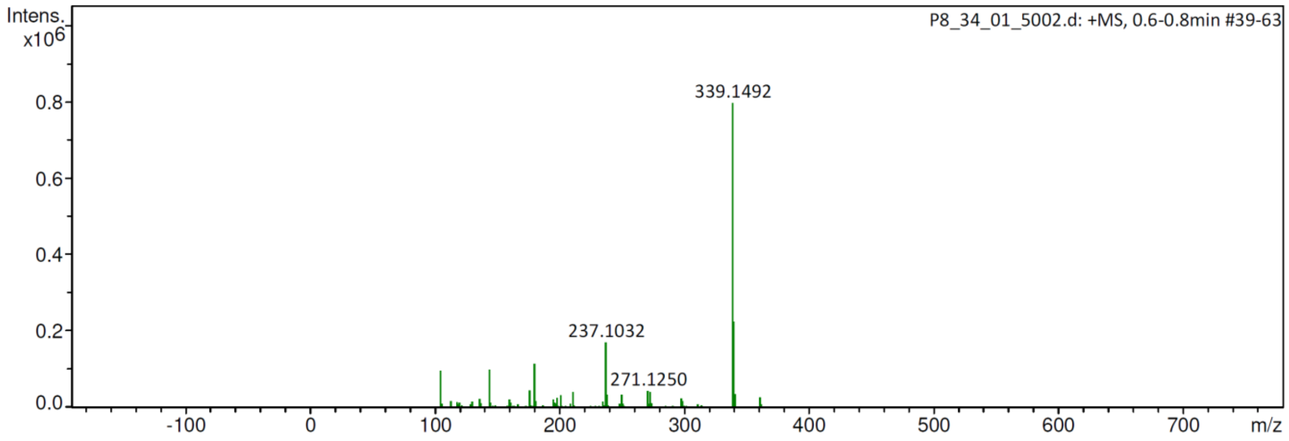
Intens.
x10^6
P8_34_01_5002.d: +MS, 0.6-0.8min #39-63
339.1492
237.1032
271.1250
0.8
0.6
0.4
0.2
0.0
-100
0
100
200
300
400
500
600
700
m/z

(3-(4-Methoxyphenyl)-5-methyl-1-phenyl-1*H*-pyrazol-4-yl)(phenyl)methanone (6i)

^{1}H NMR

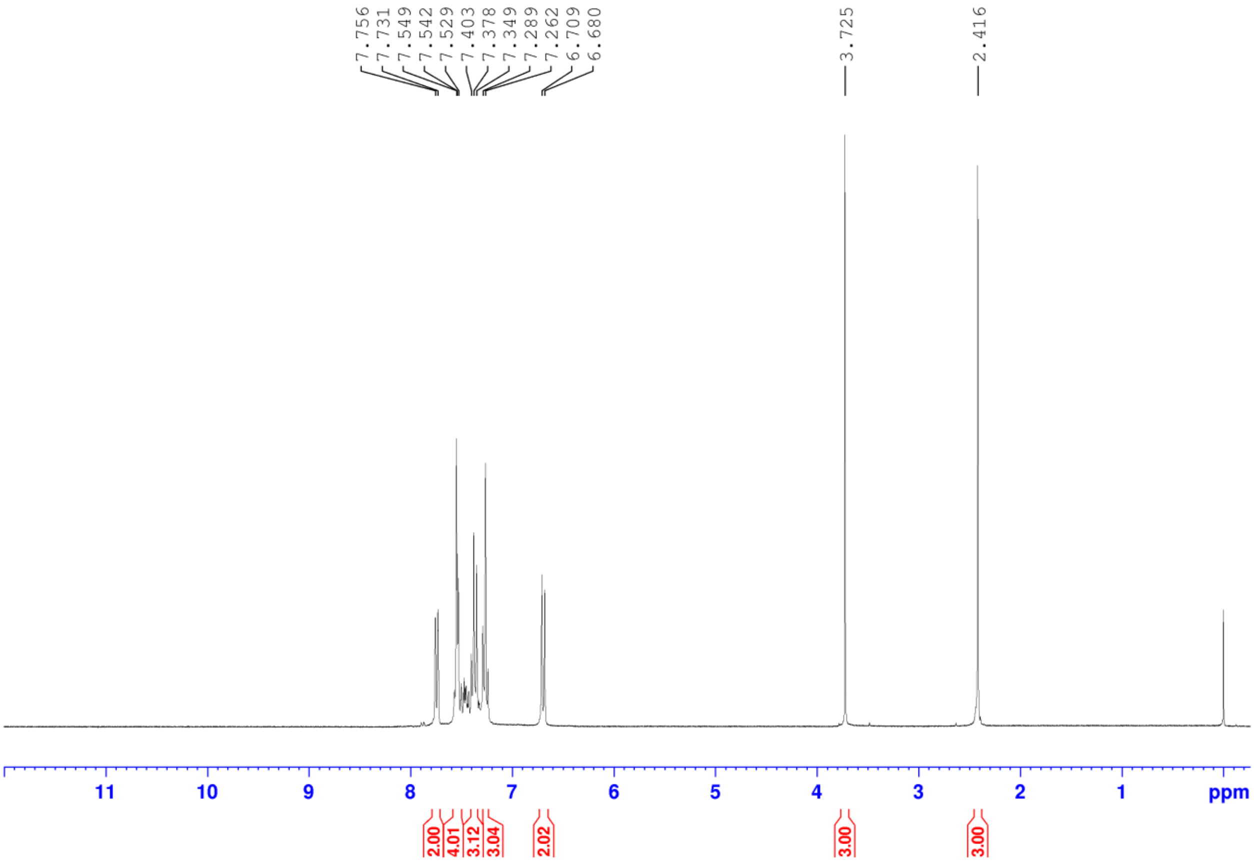

^{13}C NMR

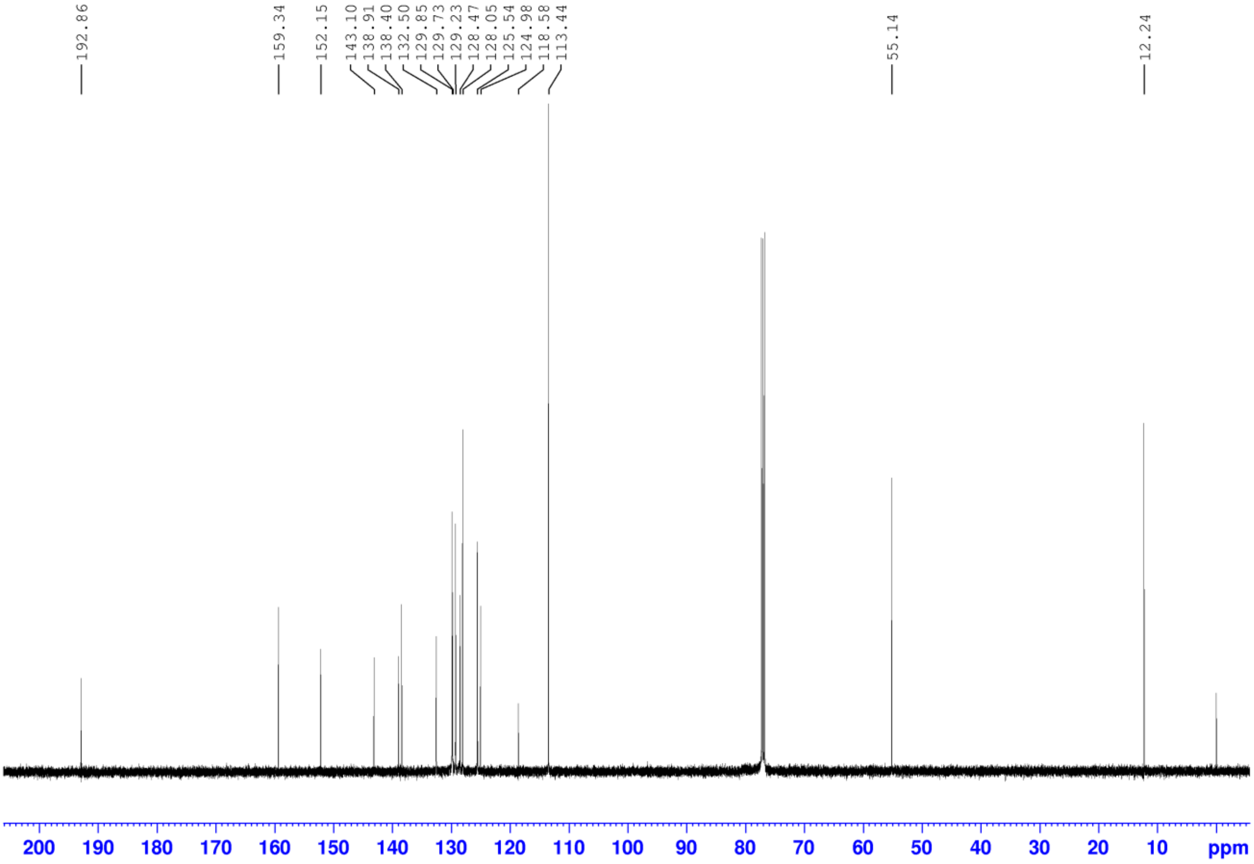
192.86
159.34
152.15
143.10
138.91
138.40
132.50
129.85
129.73
129.23
128.47
128.05
125.54
124.98
118.58
113.44
55.14
12.24
200 190 180 170 160 150 140 130 120 110 100 90 80 70 60 50 40 30 20 10 ppm

COSY NMR

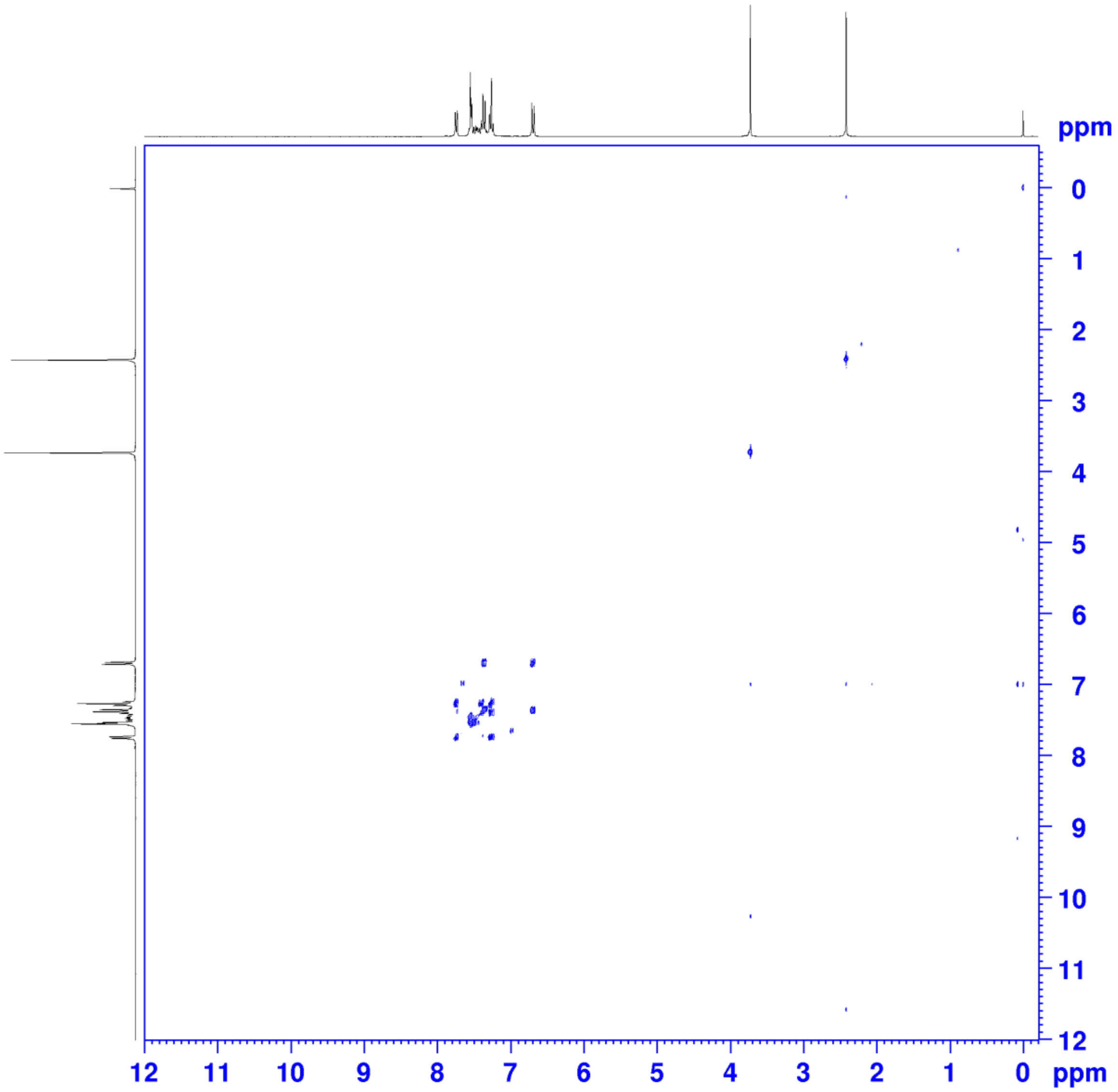

HRMS

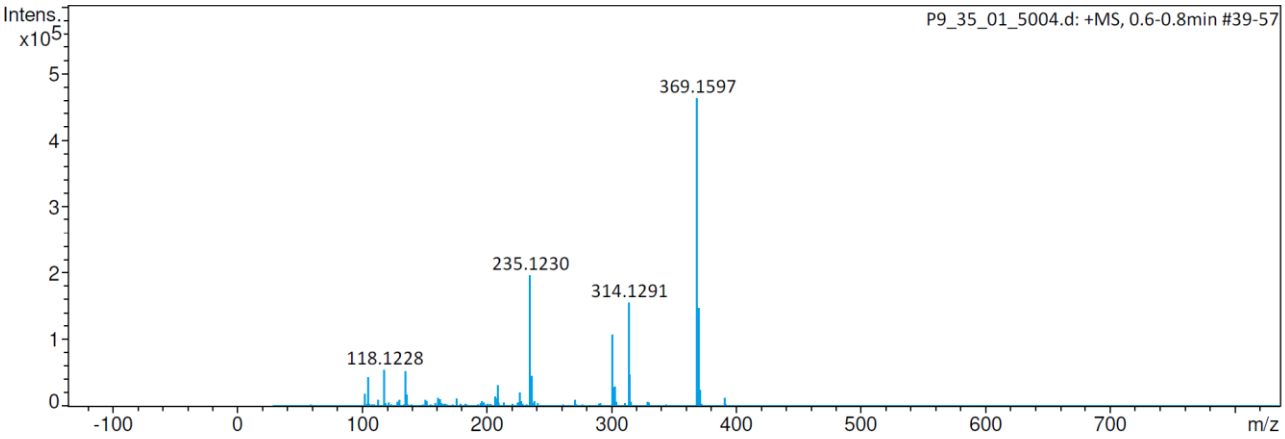

Intens.
x10^5
P9_35_01_5004.d: +MS, 0.6-0.8min #39-57
369.1597
235.1230
314.1291
118.1228
m/z

(5-Methyl-1-phenyl-3-(*p*-tolyl)-1*H*-pyrazol-4-yl)(phenyl)methanone (6j)

^{1}H NMR

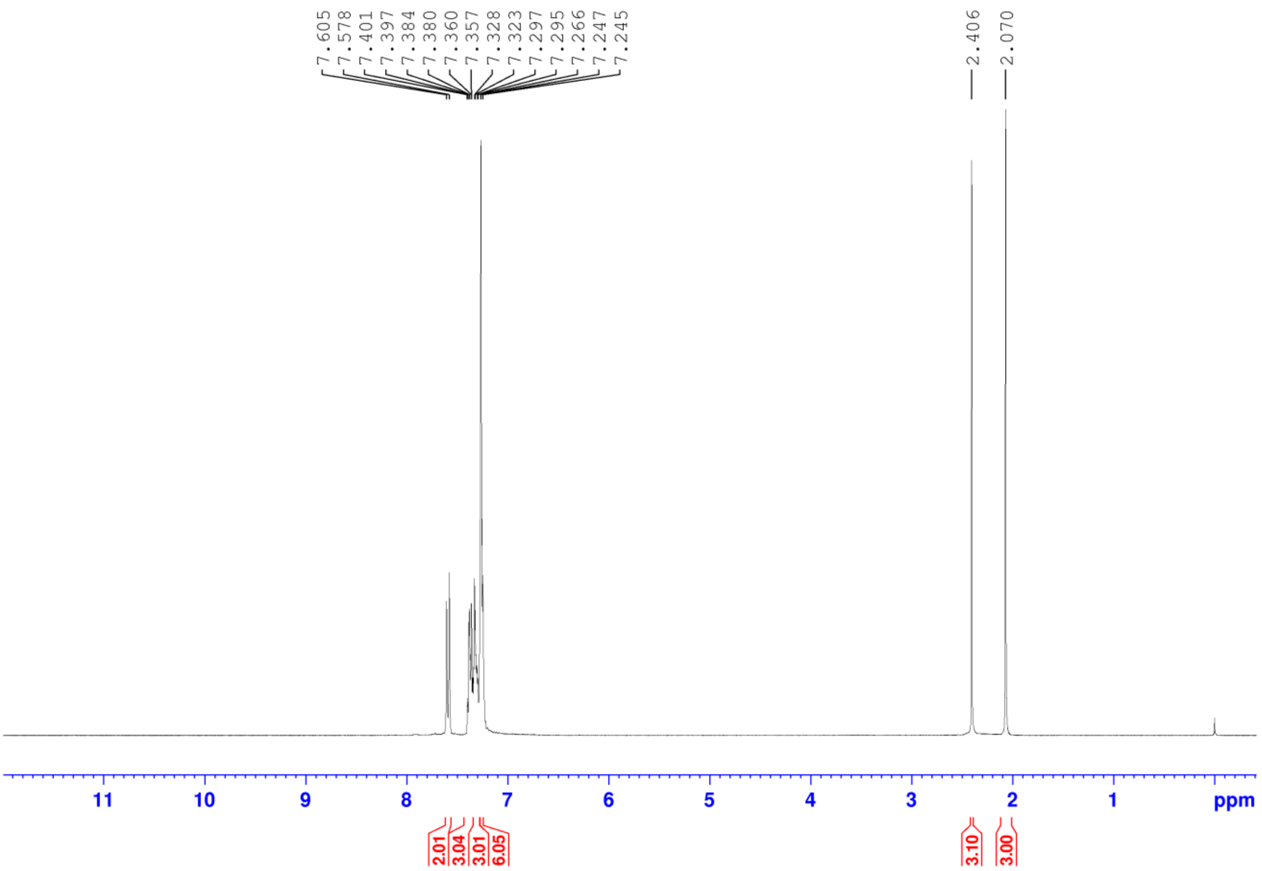

^{13}C NMR

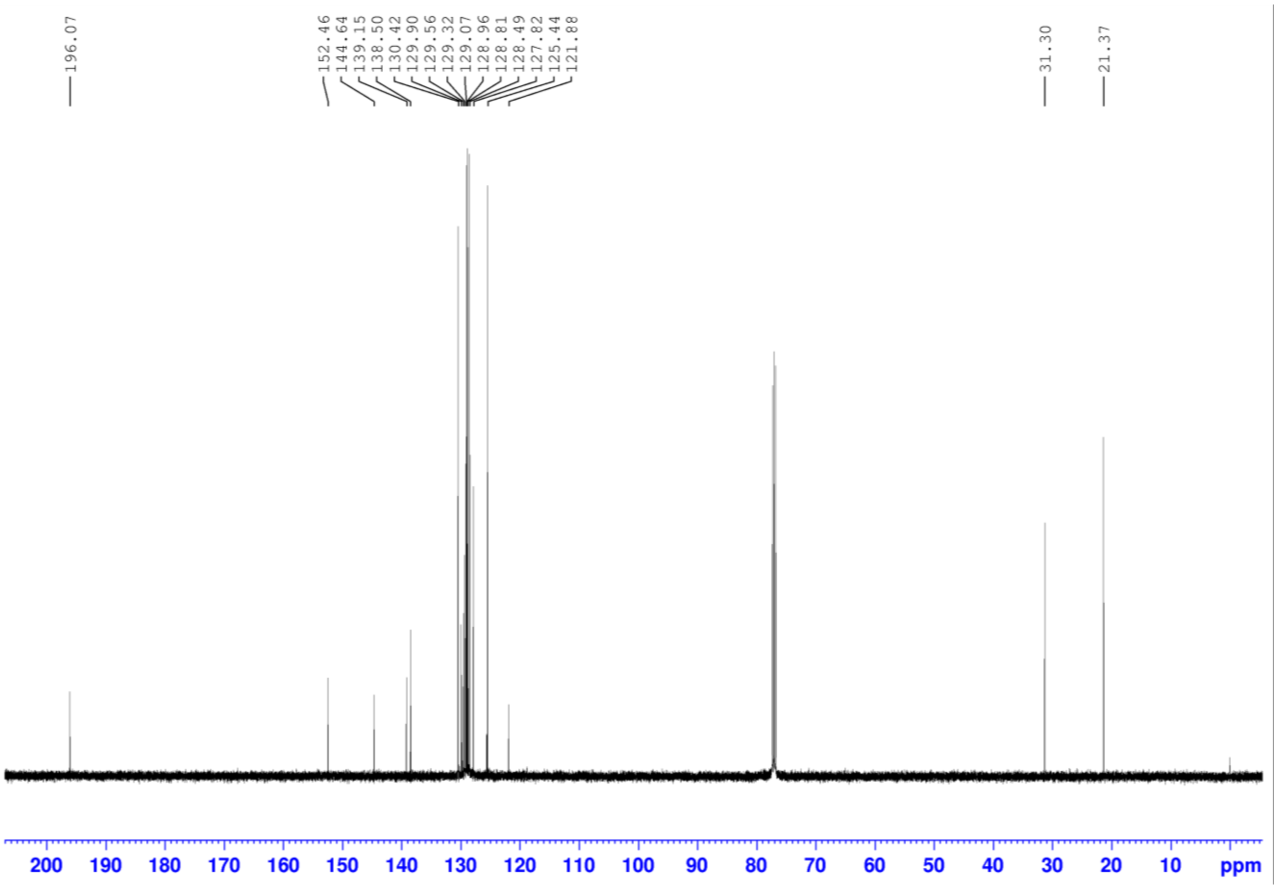
196.07
152.46
144.64
139.15
138.50
130.42
129.90
129.56
129.32
129.07
128.96
128.81
128.49
127.82
125.44
121.88
31.30
21.37
200 190 180 170 160 150 140 130 120 110 100 90 80 70 60 50 40 30 20 10 ppm

COSY NMR

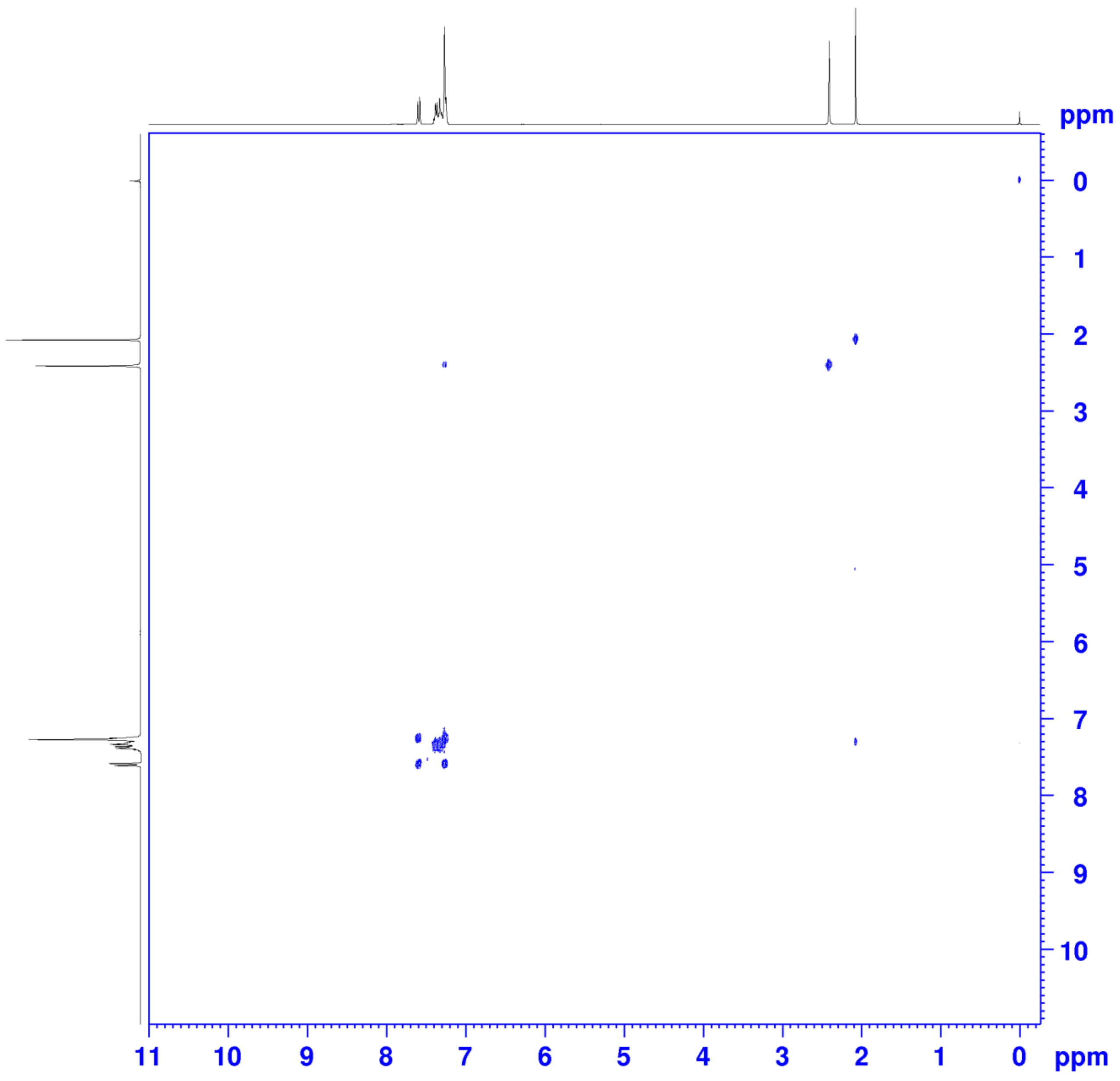

(5-Methyl-3-(4-nitrophenyl)-1-phenyl-1*H*-pyrazol-4-yl)(phenyl)methanone (6k)

^{1}H NMR

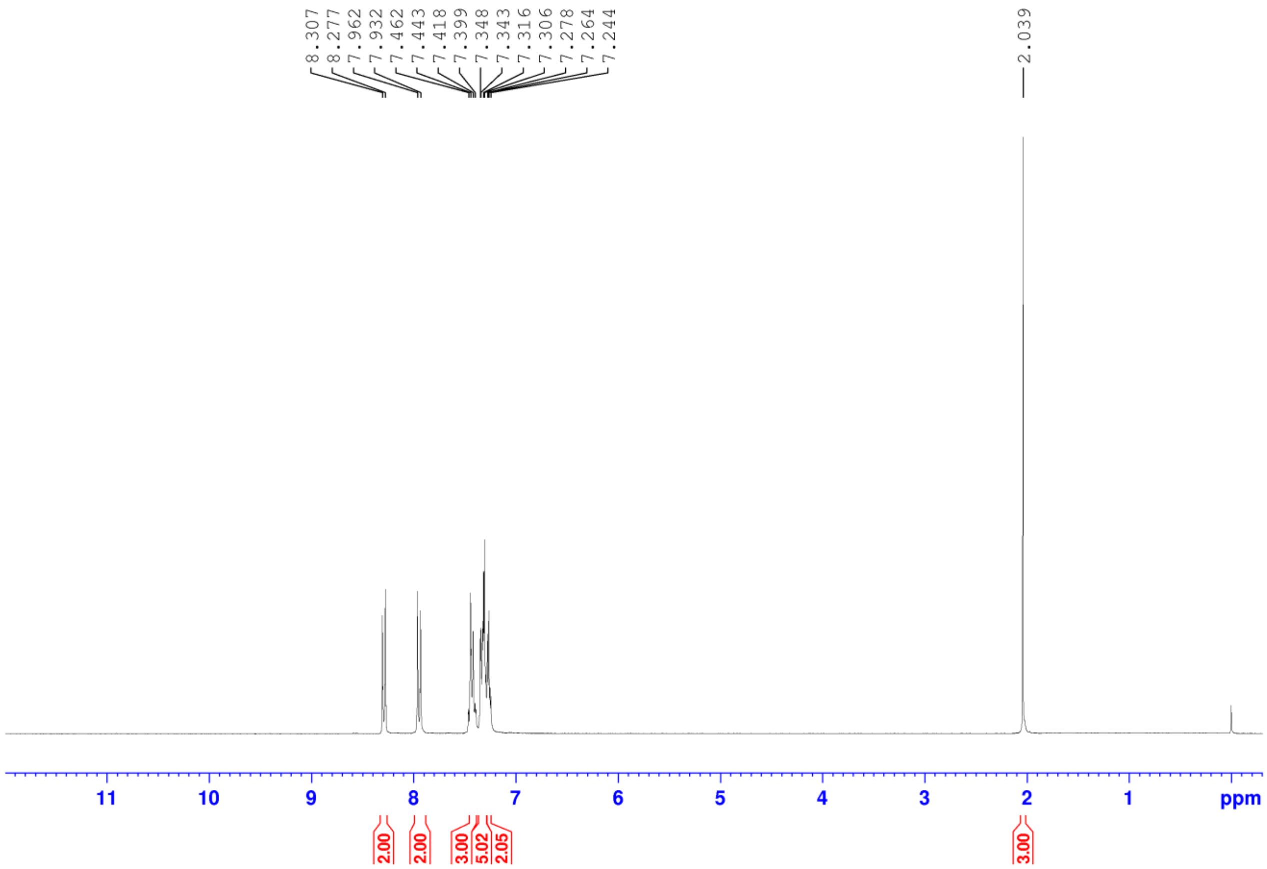

^{13}C NMR

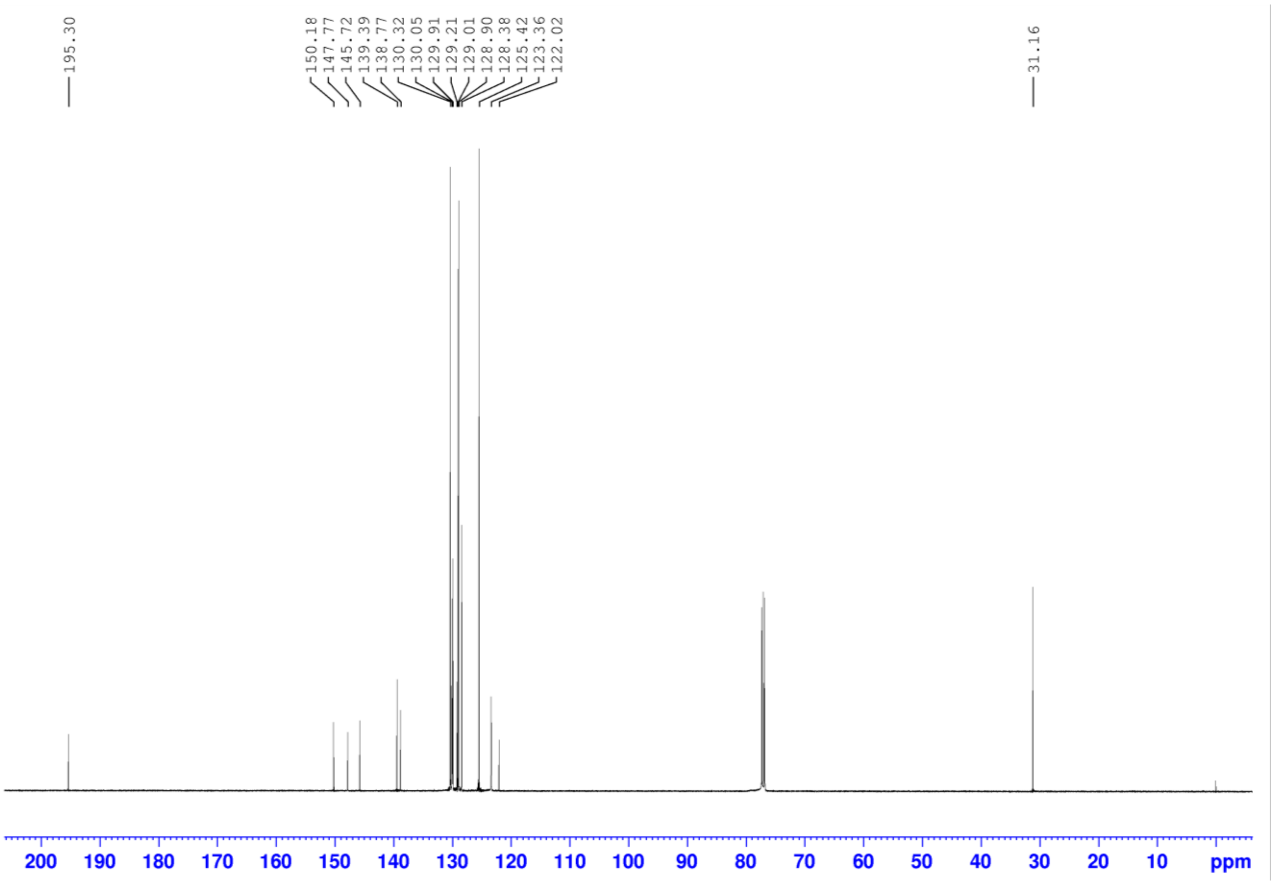

195.30
150.18
147.77
145.72
139.39
138.77
130.32
130.05
129.91
129.21
129.01
128.90
128.38
125.42
123.36
122.02
31.16
200 190 180 170 160 150 140 130 120 110 100 90 80 70 60 50 40 30 20 10 ppm

COSY NMR

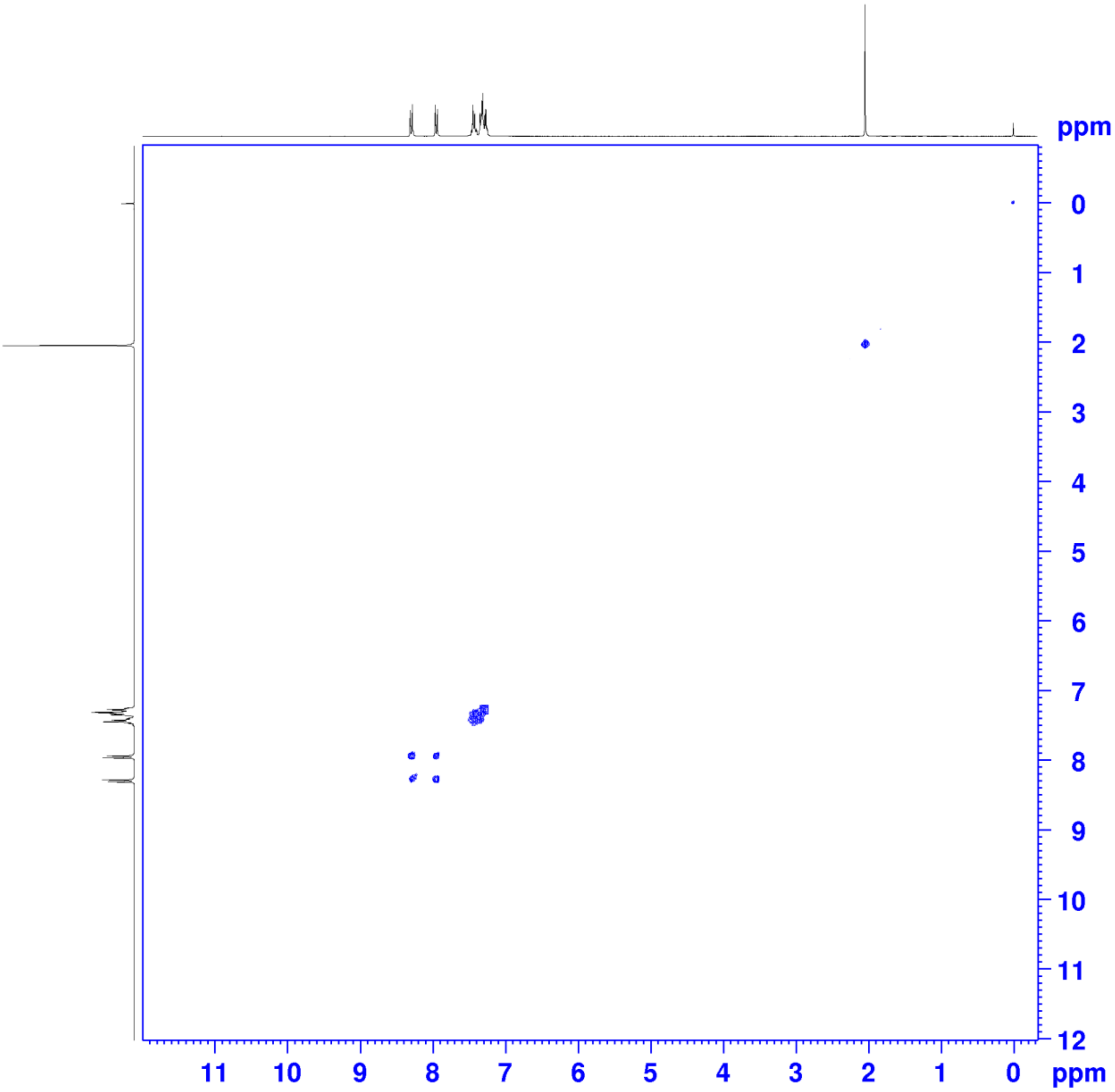

HRMS

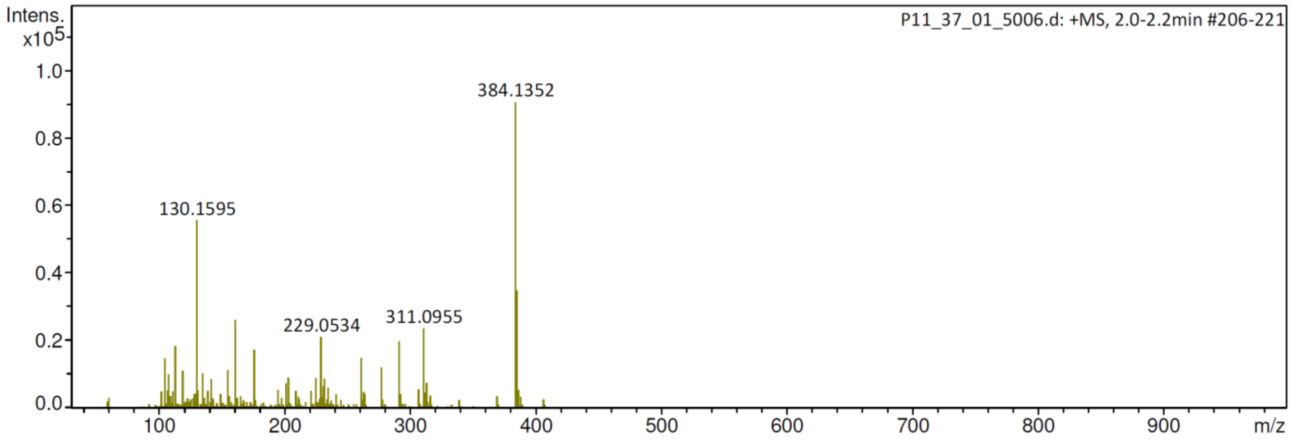
Intens.
x10^5
1.0
0.8
0.6
0.4
0.2
0.0
P11_37_01_5006.d: +MS, 2.0-2.2min #206-221
384.1352
130.1595
229.0534
311.0955
100
200
300
400
500
600
700
800
900
m/z

(3-(2-Chlorophenyl)-5-methyl-1-phenyl-1*H*-pyrazol-4-yl)(phenyl)methanone (6l)

^{1}H NMR

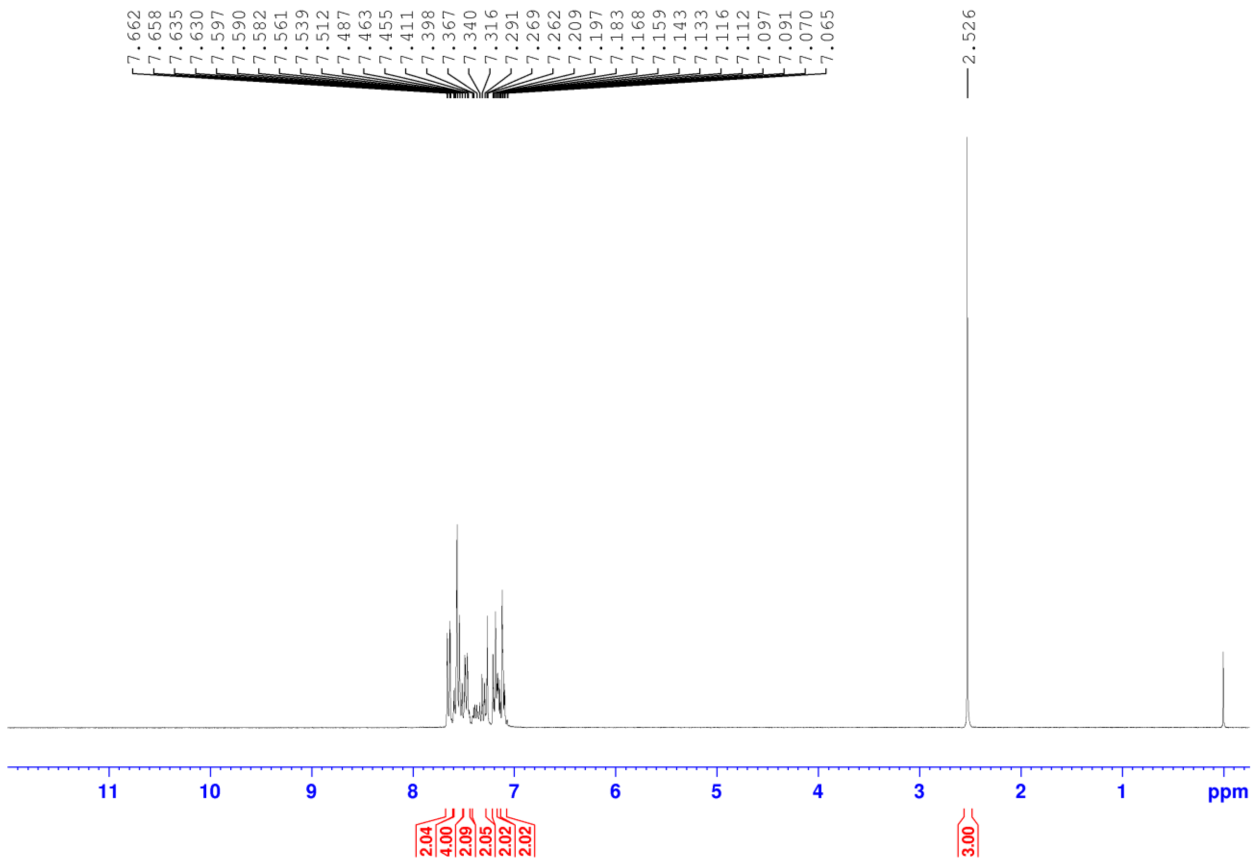

^{13}C NMR

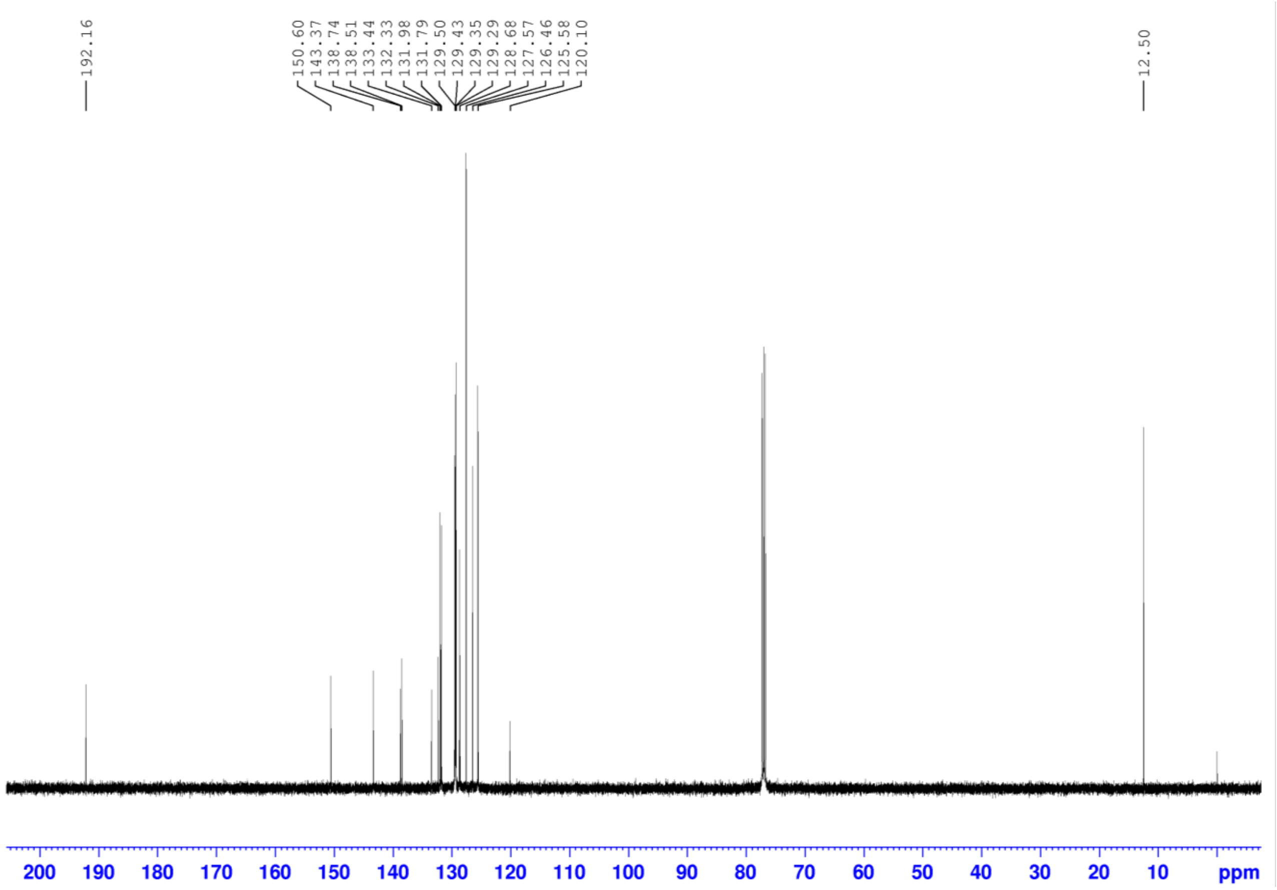
192.16
150.60
143.37
138.74
138.51
133.44
132.33
131.98
131.79
129.50
129.43
129.35
129.29
128.68
127.57
126.46
125.58
120.10
12.50
200
190
180
170
160
150
140
130
120
110
100
90
80
70
60
50
40
30
20
10
ppm

COSY NMR

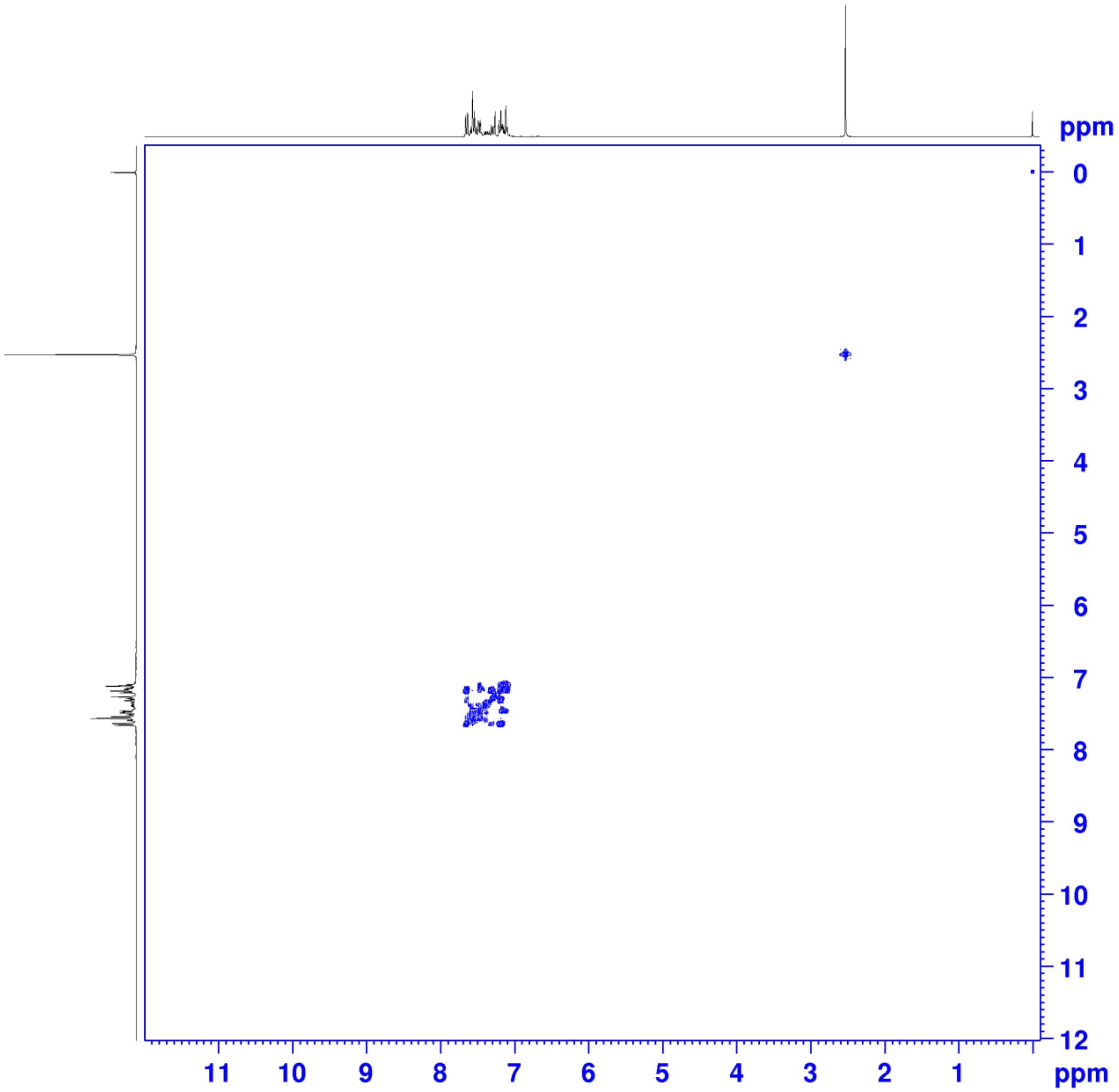

HRMS

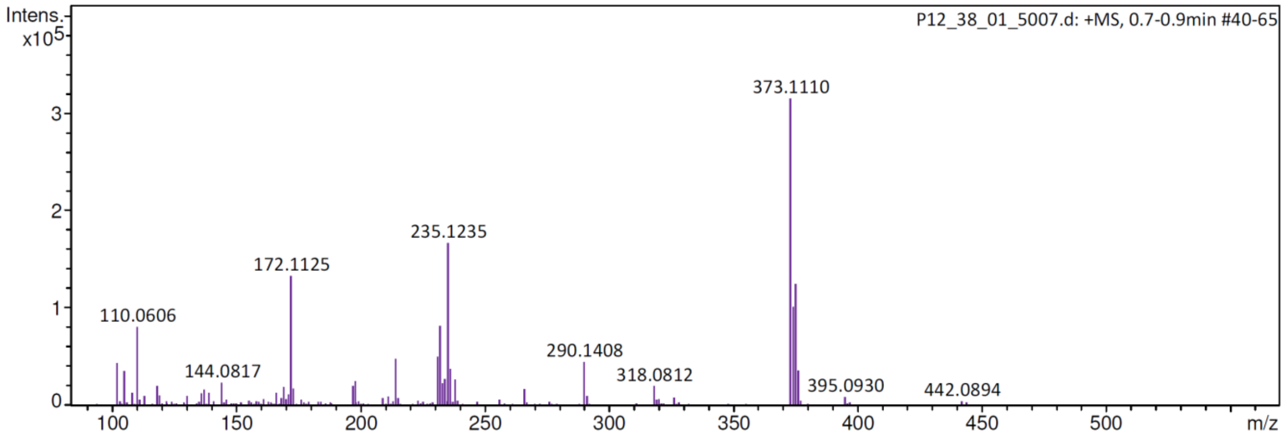

Intens.
x10^5
P12_38_01_5007.d: +MS, 0.7-0.9min #40-65
373.1110
235.1235
172.1125
110.0606
144.0817
290.1408
318.0812
395.0930
442.0894
3
2
1
0
100
150
200
250
300
350
400
450
500
m/z

(3-(3-Chlorophenyl)-5-methyl-1-phenyl-1*H*-pyrazol-4-yl)(phenyl)methanone (6m)

^{1}H NMR

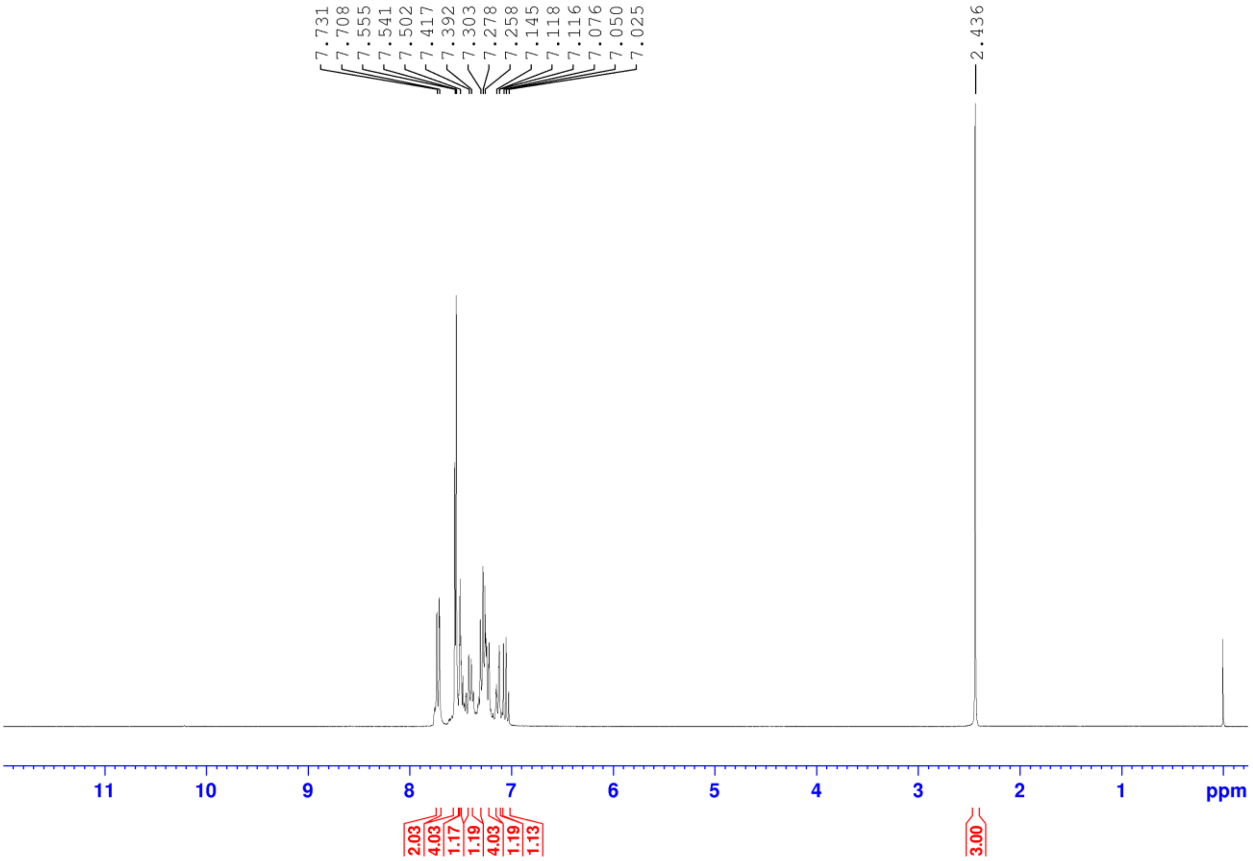

^{13}C NMR

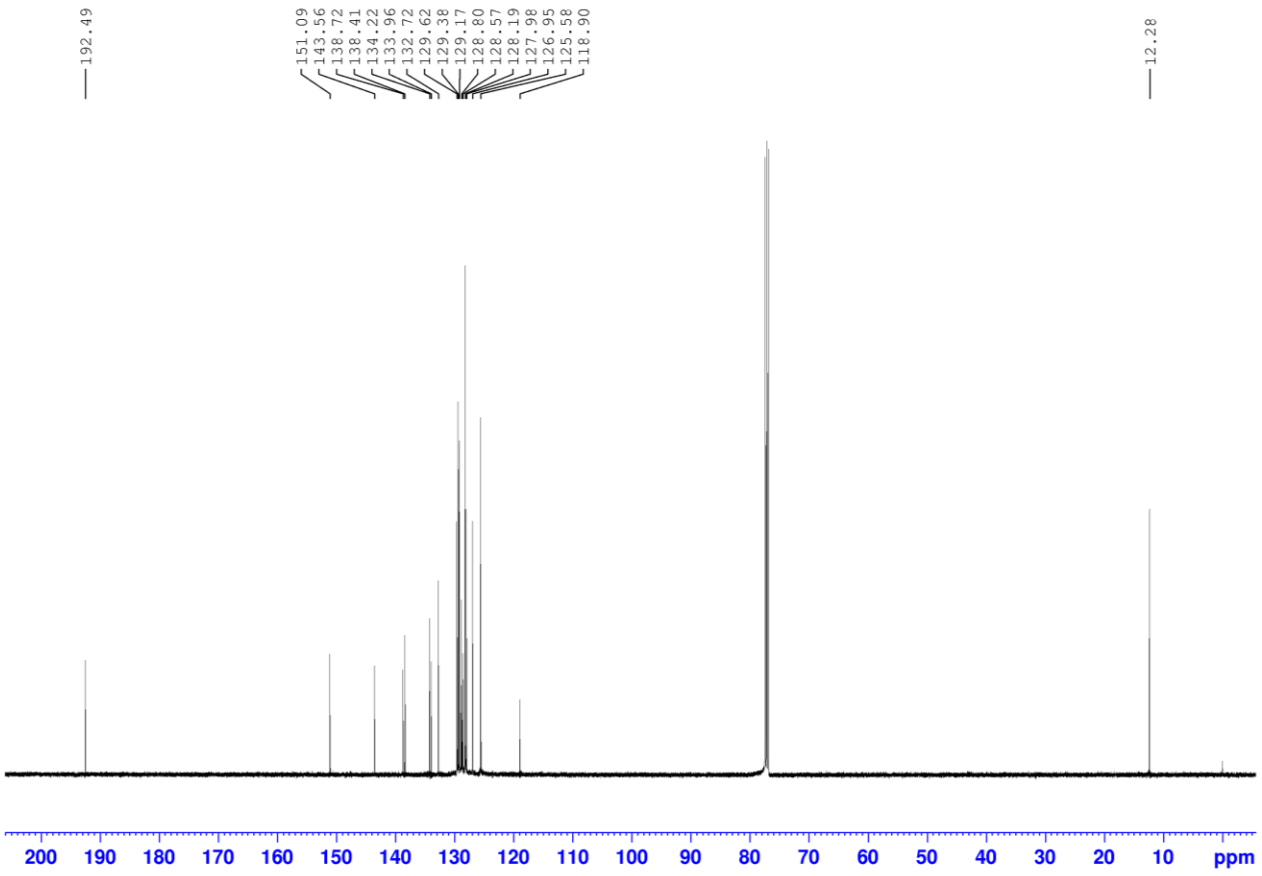
192.49
151.09
143.56
138.72
138.41
134.22
133.96
132.72
129.62
129.38
129.17
128.80
128.57
128.19
127.98
126.95
125.58
118.90
12.28
200 190 180 170 160 150 140 130 120 110 100 90 80 70 60 50 40 30 20 10 ppm

COSY NMR

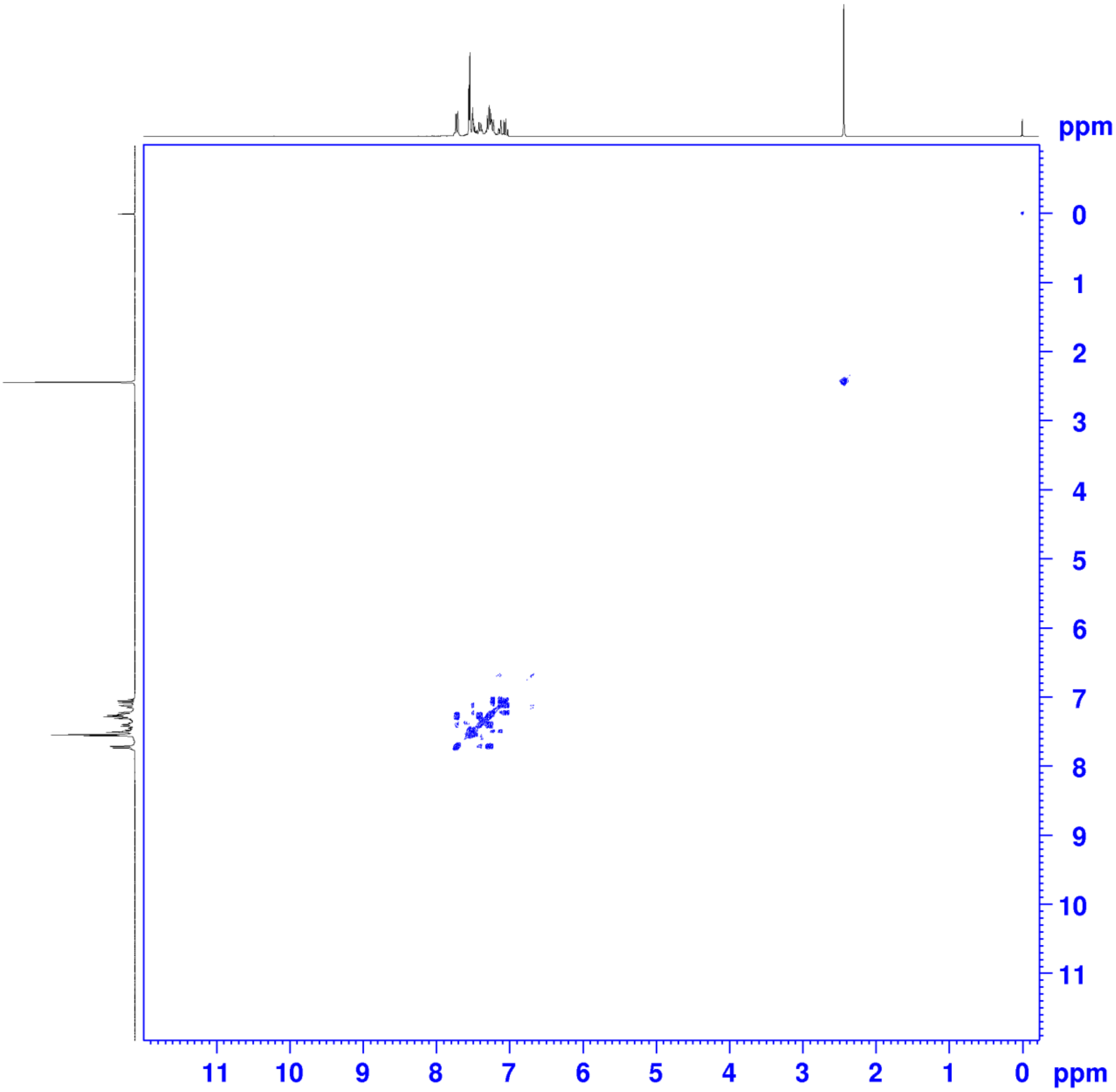

HRMS

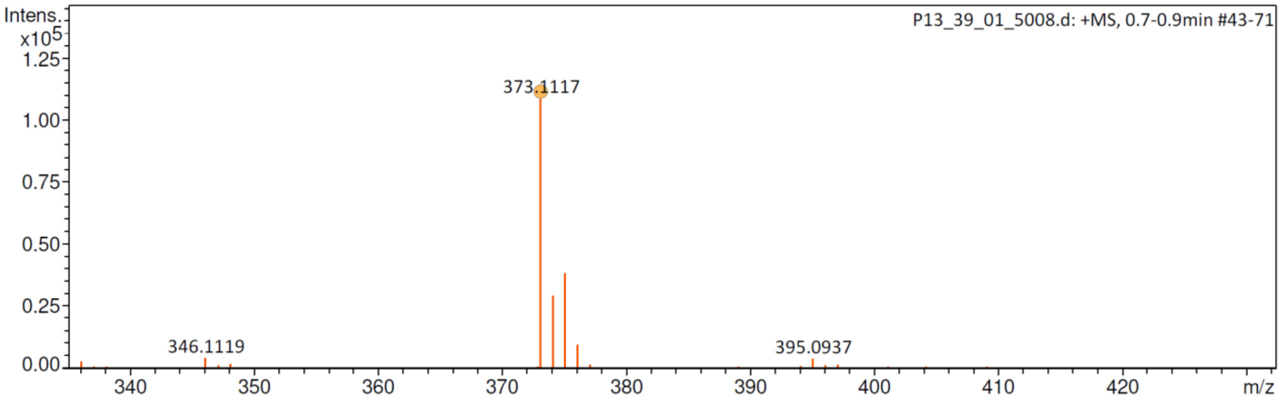
Intens.
x10^5
1.25
1.00
0.75
0.50
0.25
0.00
P13_39_01_5008.d: +MS, 0.7-0.9min #43-71
373.1117
346.1119
395.0937
340
350
360
370
380
390
400
410
420
m/z

(3-(4-Chlorophenyl)-5-methyl-1-phenyl-1*H*-pyrazol-4-yl)(phenyl)methanone (6n)

^{1}H NMR

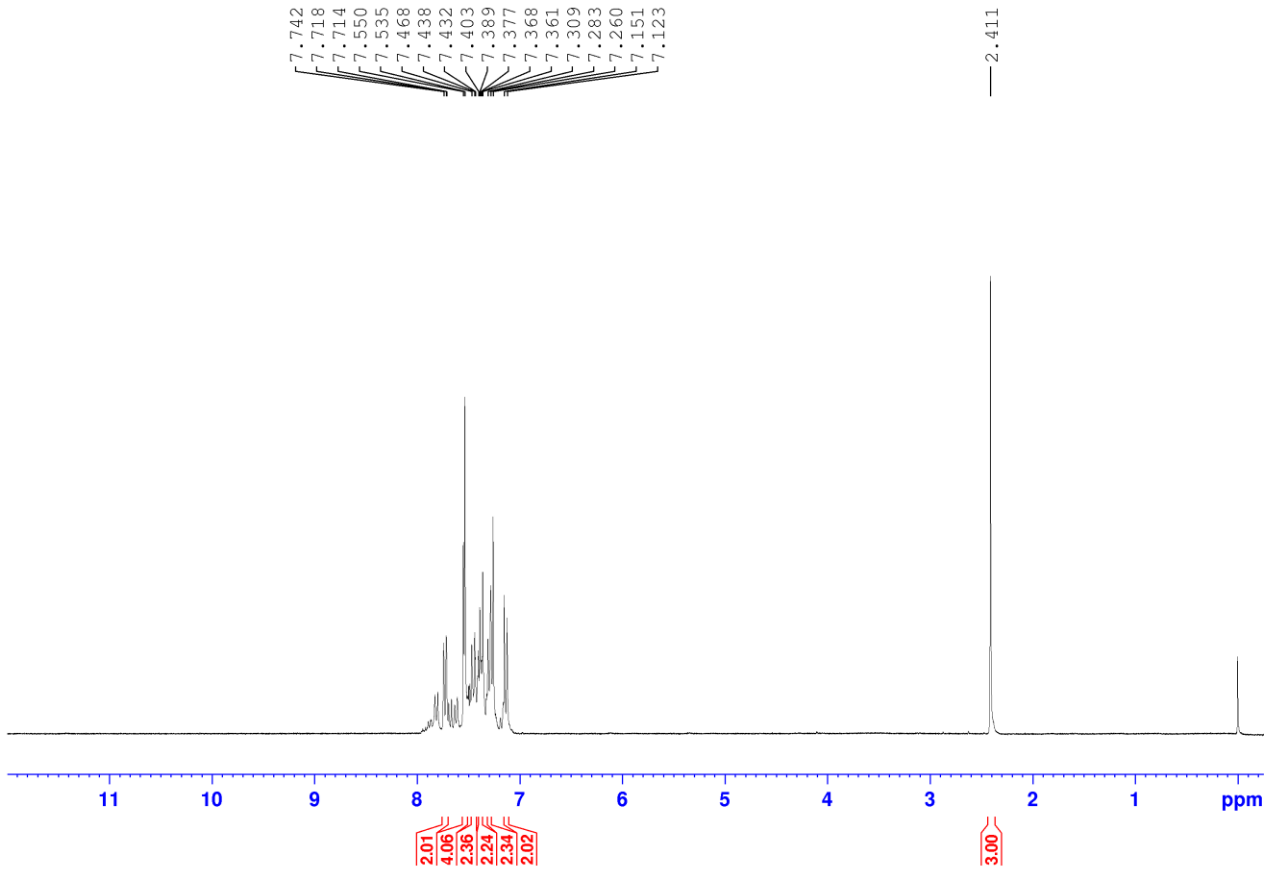

^{13}C NMR

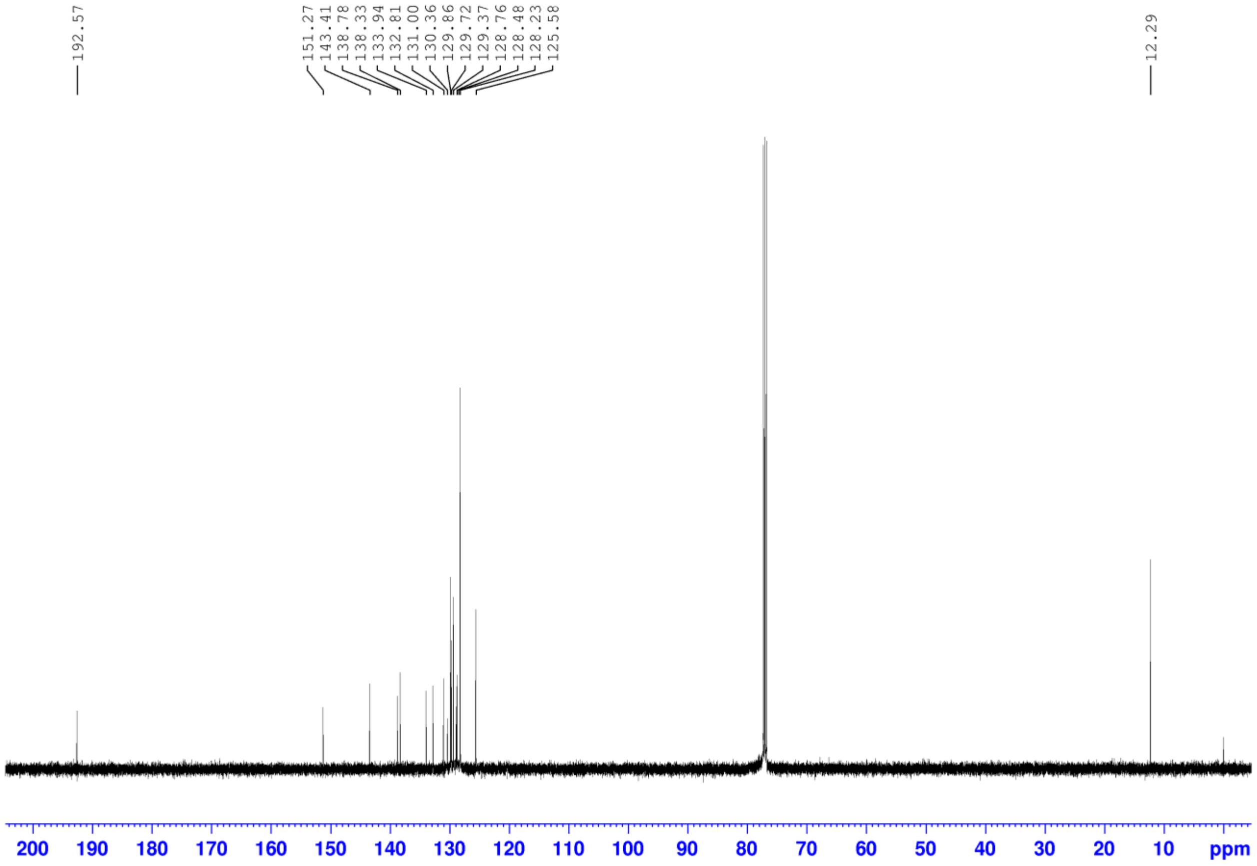

192.57
151.27
143.41
138.78
138.33
133.94
132.81
131.00
130.36
129.86
129.72
129.37
128.76
128.48
128.23
125.58
12.29
200
190
180
170
160
150
140
130
120
110
100
90
80
70
60
50
40
30
20
10
ppm

COSY NMR

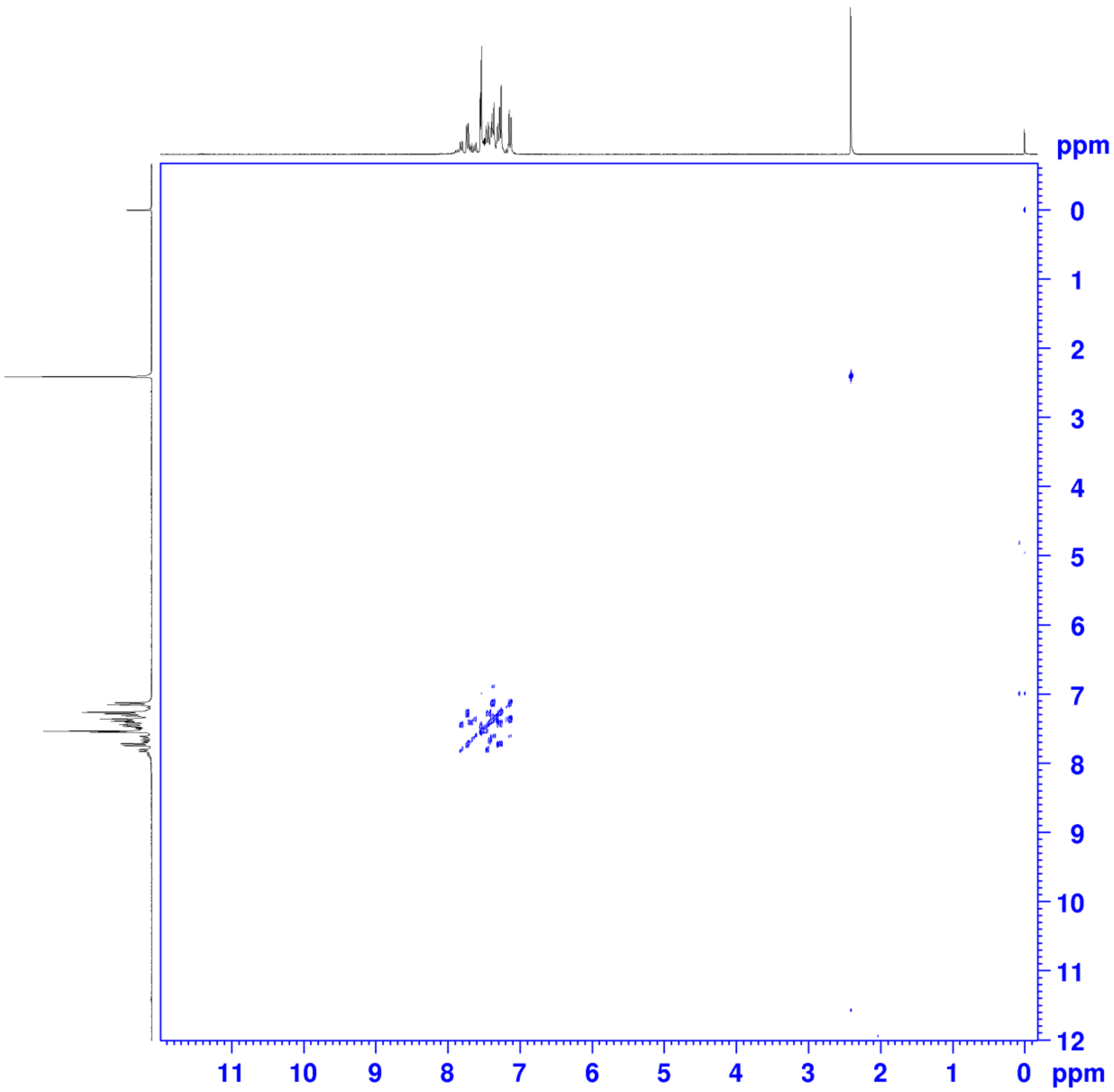

HRMS

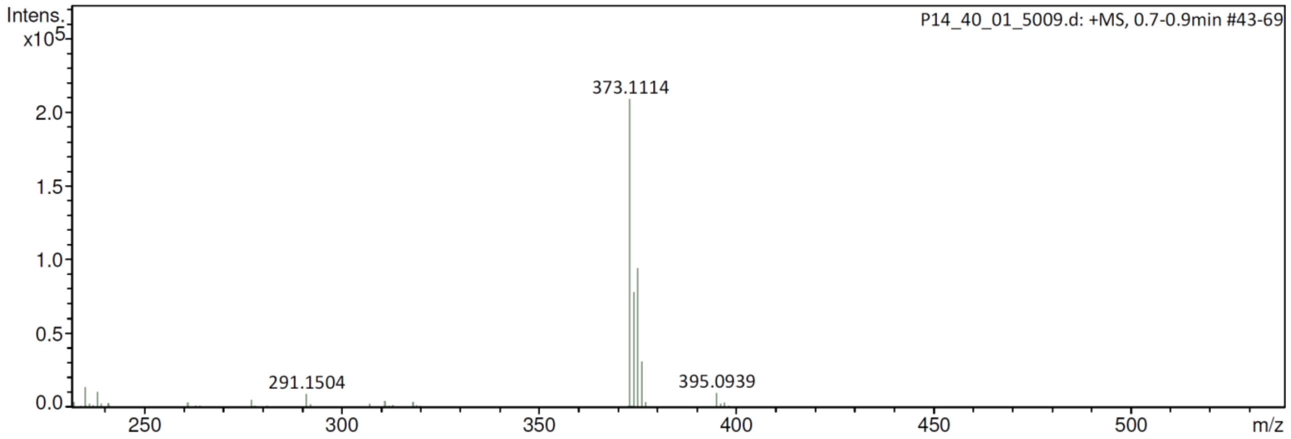

Intens.
x10^5
P14_40_01_5009.d: +MS, 0.7-0.9min #43-69
373.1114
291.1504
395.0939
2.0
1.5
1.0
0.5
0.0
250
300
350
400
450
500
m/z

Phenyl(1,3,5-triphenyl-1*H*-pyrazol-4-yl)methanone (6o)

^{1}H NMR

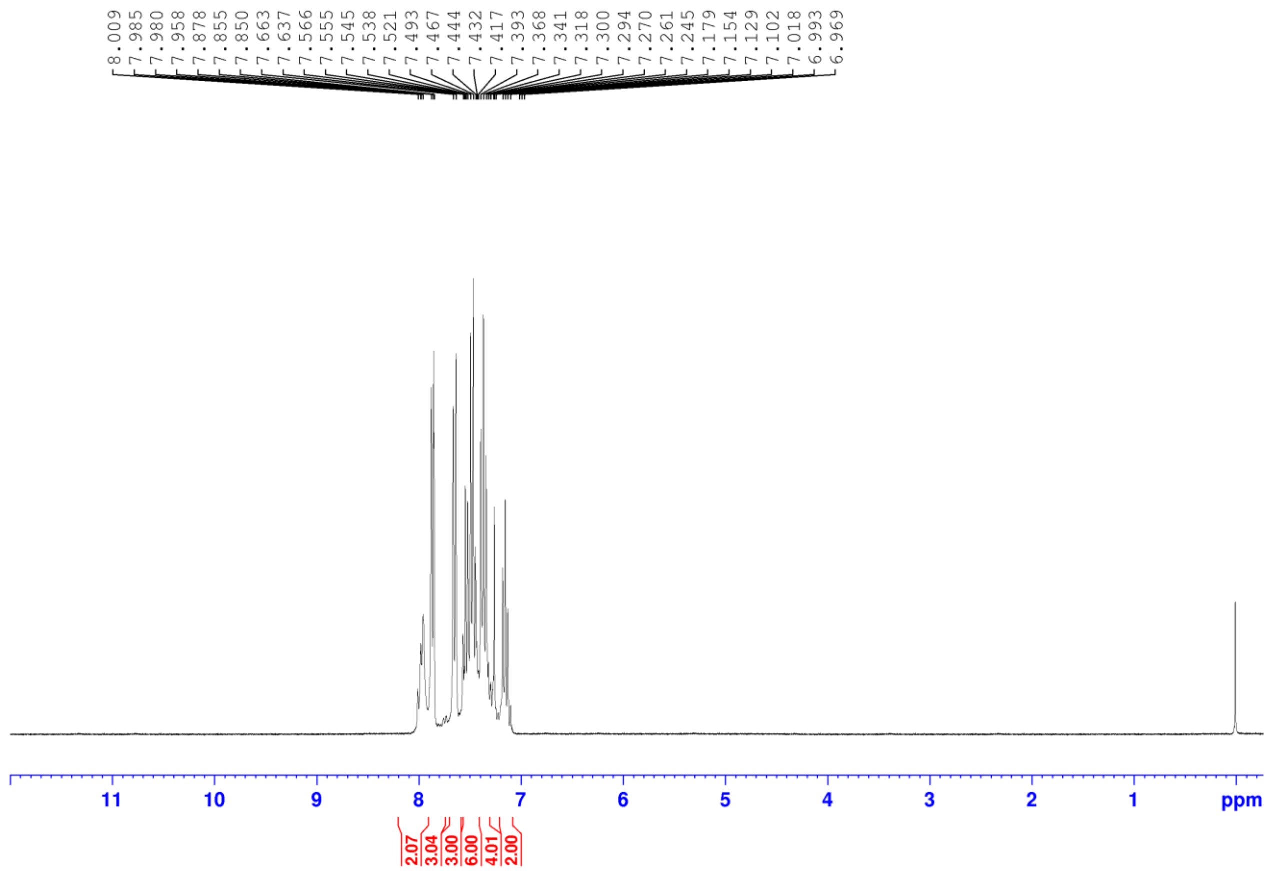

^{13}C NMR

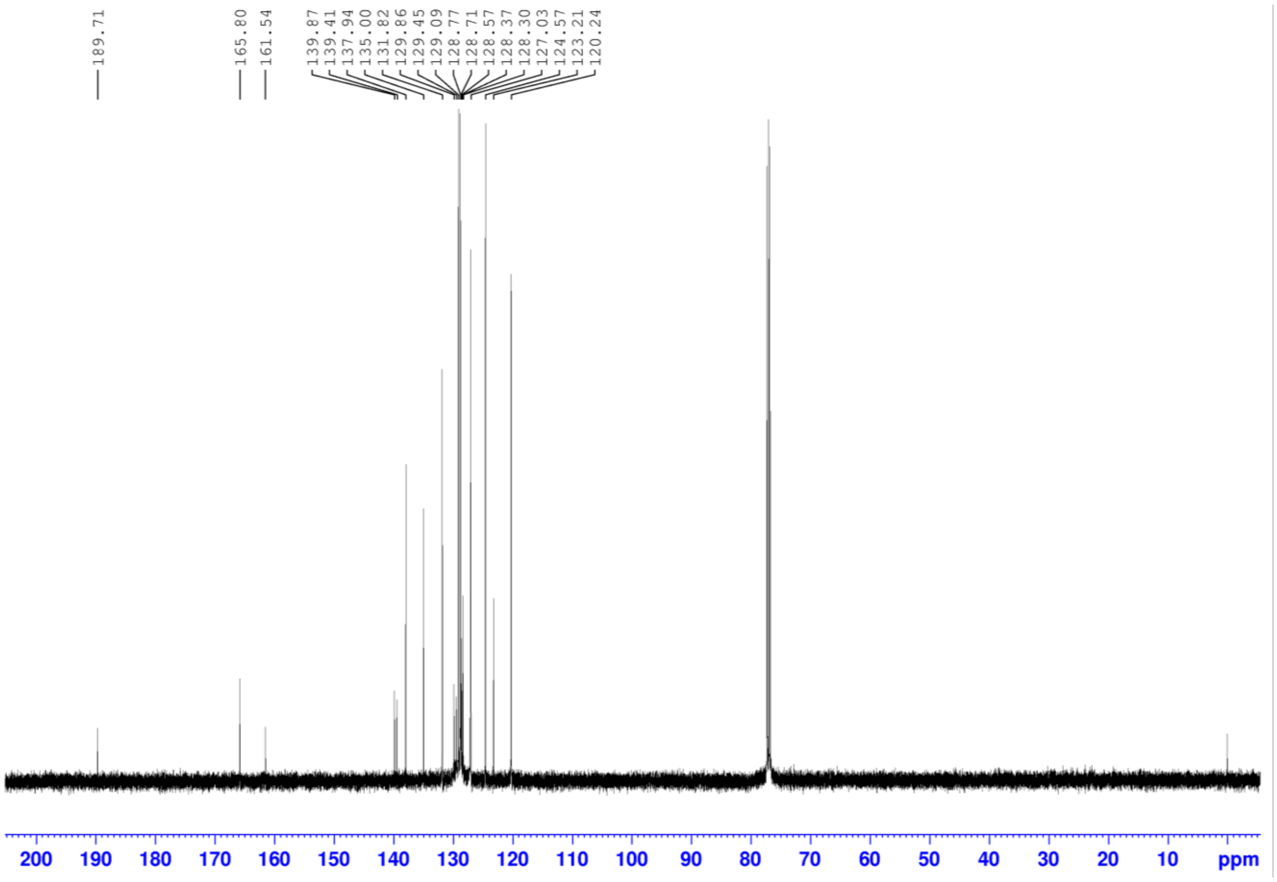

189.71
165.80
161.54
139.87
139.41
137.94
135.00
131.82
129.86
129.45
129.09
128.77
128.71
128.57
128.37
128.30
127.03
124.57
123.21
120.24
200 190 180 170 160 150 140 130 120 110 100 90 80 70 60 50 40 30 20 10 ppm

COSY NMR

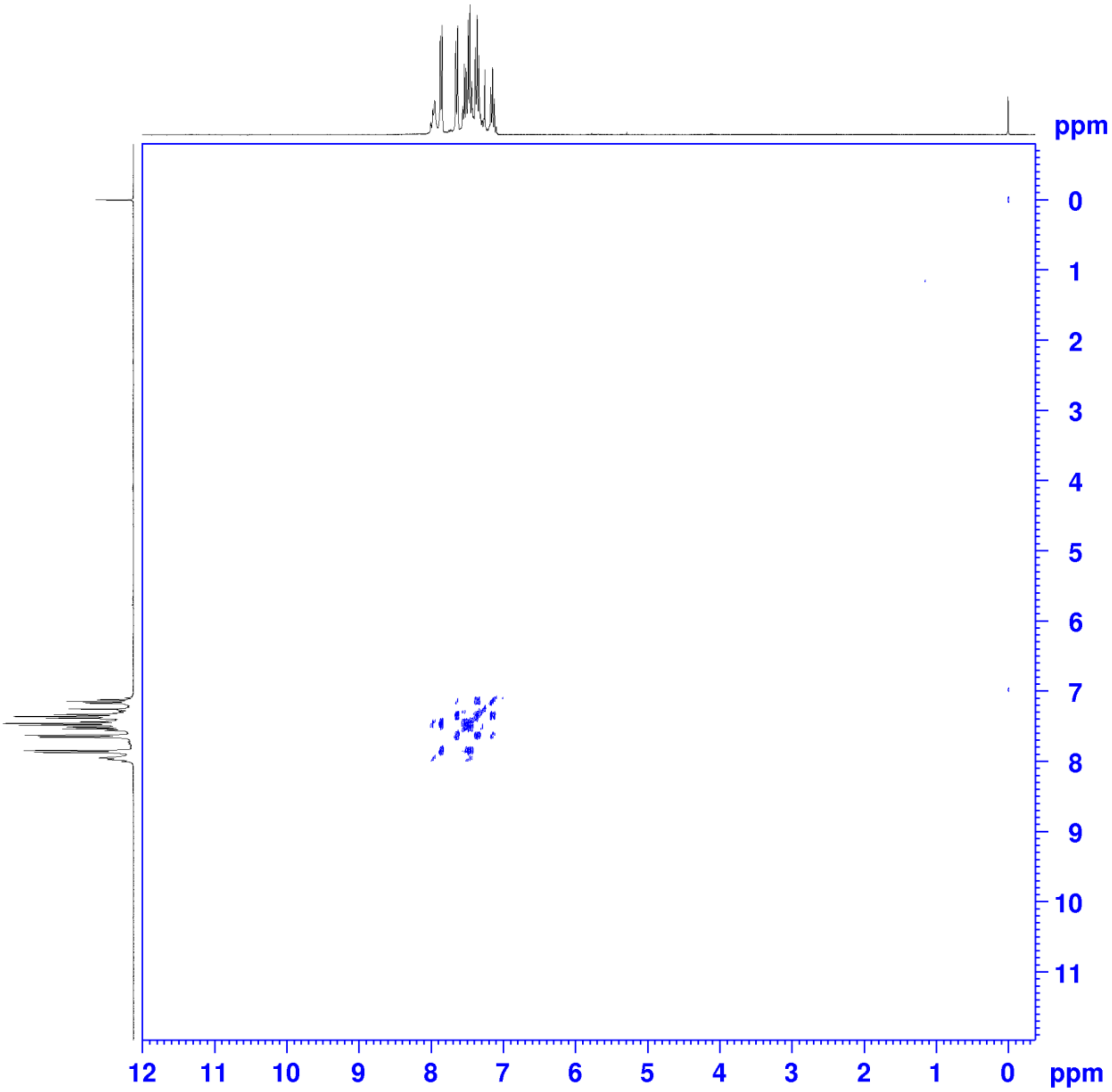

HRMS

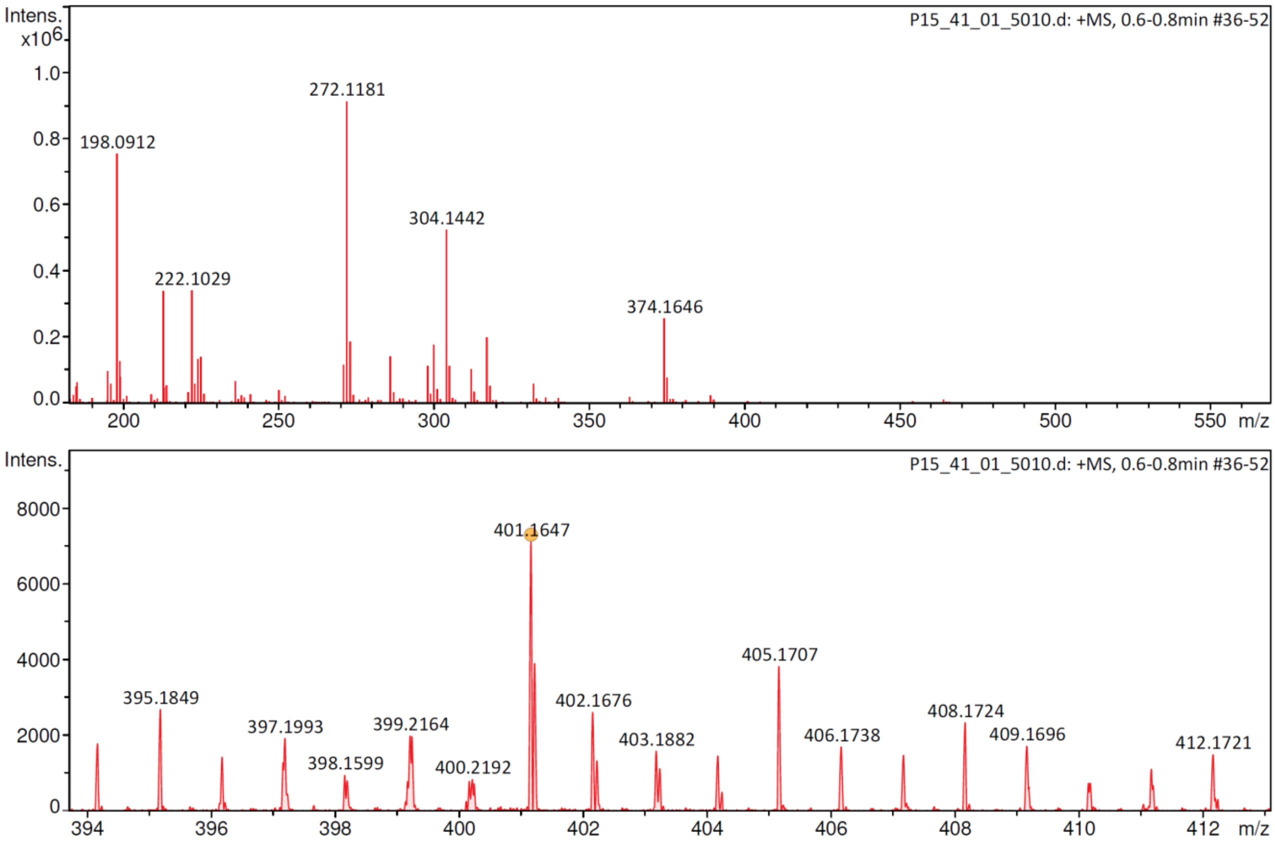
Intens.
x10^6
1.0
0.8
0.6
0.4
0.2
0.0
P15_41_01_5010.d: +MS, 0.6-0.8min #36-52
198.0912
222.1029
272.1181
304.1442
374.1646
200
250
300
350
400
450
500
550
m/z
Intens.
8000
6000
4000
2000
0
P15_41_01_5010.d: +MS, 0.6-0.8min #36-52
401.1647
395.1849
397.1993
398.1599
399.2164
400.2192
402.1676
403.1882
405.1707
406.1738
408.1724
409.1696
412.1721
394
396
398
400
402
404
406
408
410
412
m/z

^{1}H NMR spectra of crude reaction mixtures illustrated in Table 1.

Entry 1

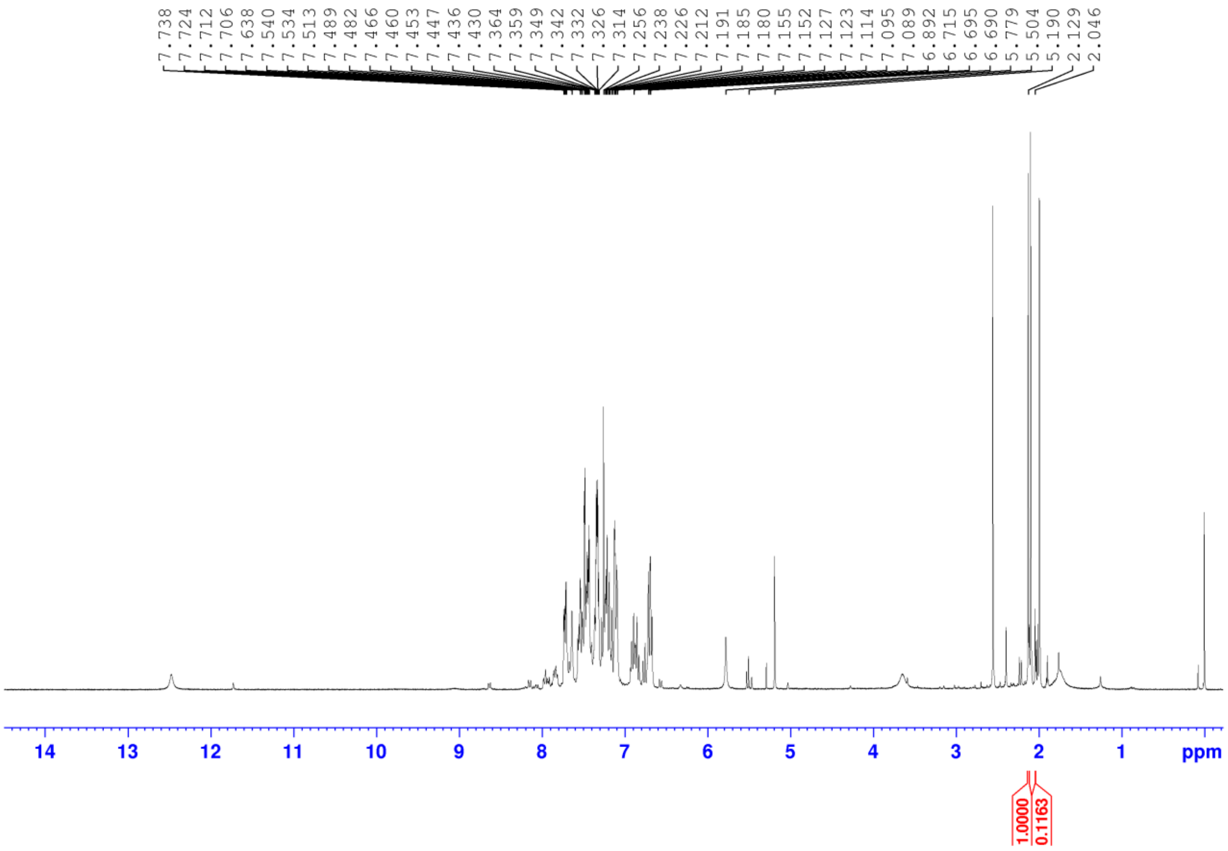

Entry 2

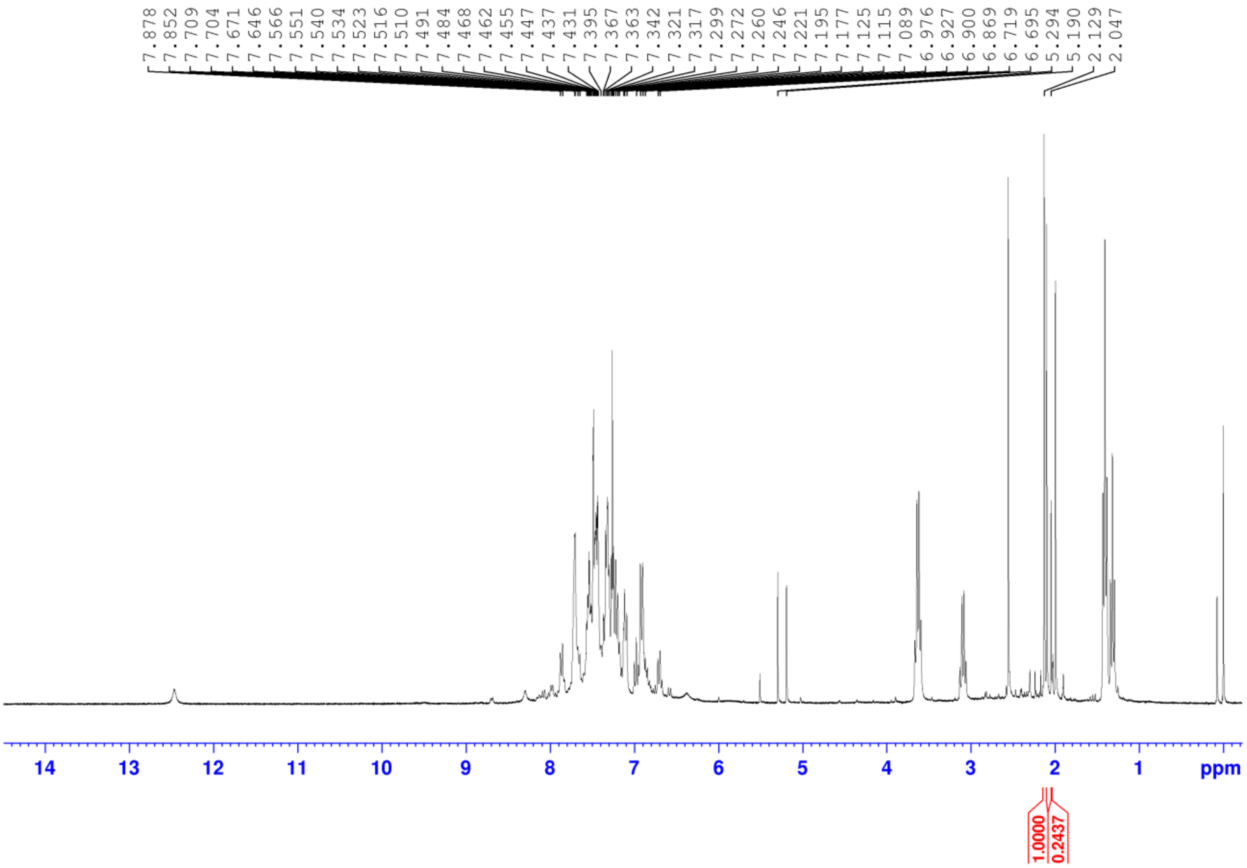

7.878
7.852
7.709
7.704
7.671
7.646
7.566
7.551
7.540
7.534
7.523
7.516
7.510
7.491
7.484
7.468
7.462
7.455
7.447
7.437
7.431
7.395
7.367
7.363
7.342
7.321
7.317
7.299
7.272
7.260
7.246
7.221
7.195
7.177
7.125
7.115
7.089
6.976
6.927
6.900
6.869
6.719
6.695
5.294
5.190
2.129
2.047
14
13
12
11
10
9
8
7
6
5
4
3
2
1
ppm
1.0000
0.2437

Entry 3

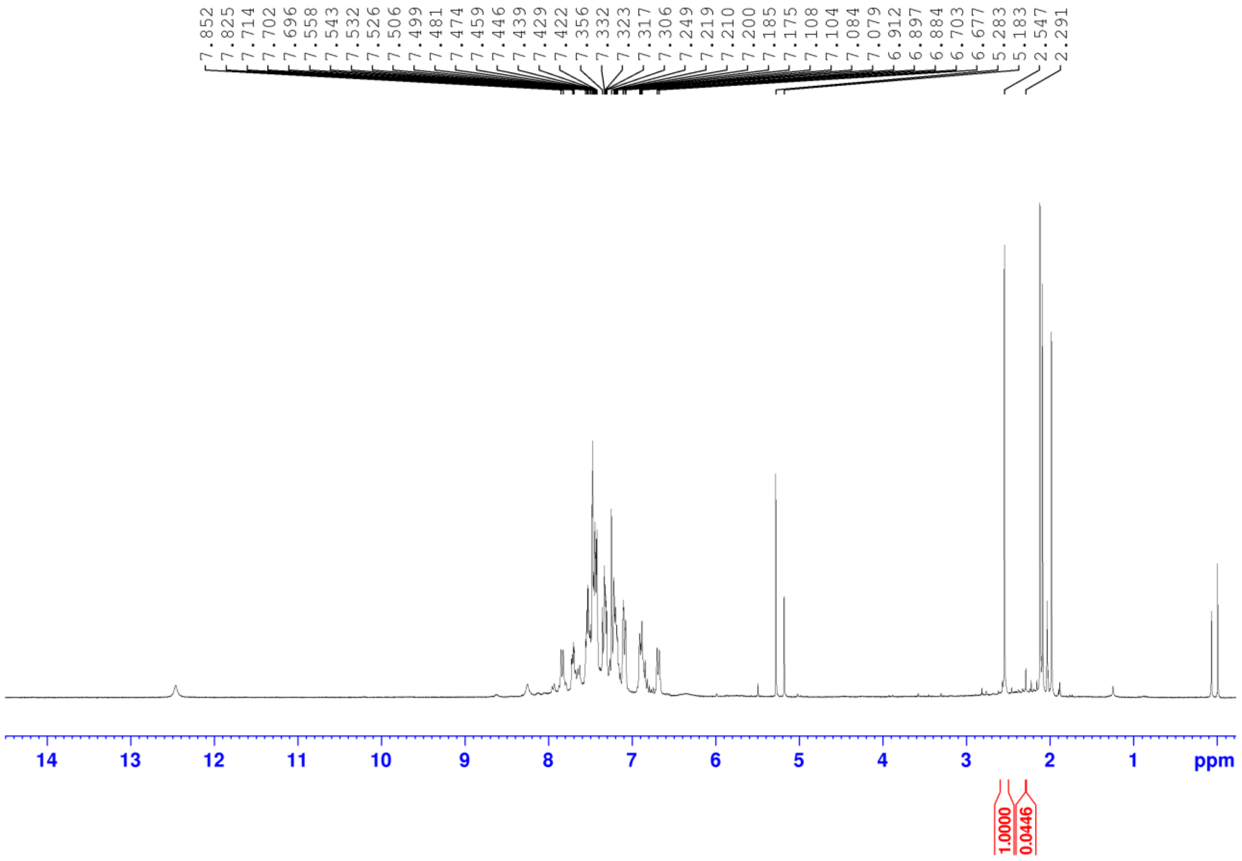
7.852
7.825
7.714
7.702
7.696
7.558
7.543
7.532
7.526
7.506
7.499
7.481
7.474
7.459
7.446
7.439
7.429
7.422
7.356
7.332
7.323
7.317
7.306
7.249
7.219
7.210
7.200
7.185
7.175
7.108
7.104
7.084
7.079
6.912
6.897
6.884
6.703
6.677
5.283
5.183
2.547
2.291
14
13
12
11
10
9
8
7
6
5
4
3
2
1
ppm
1.0000
0.0446

Entry 4

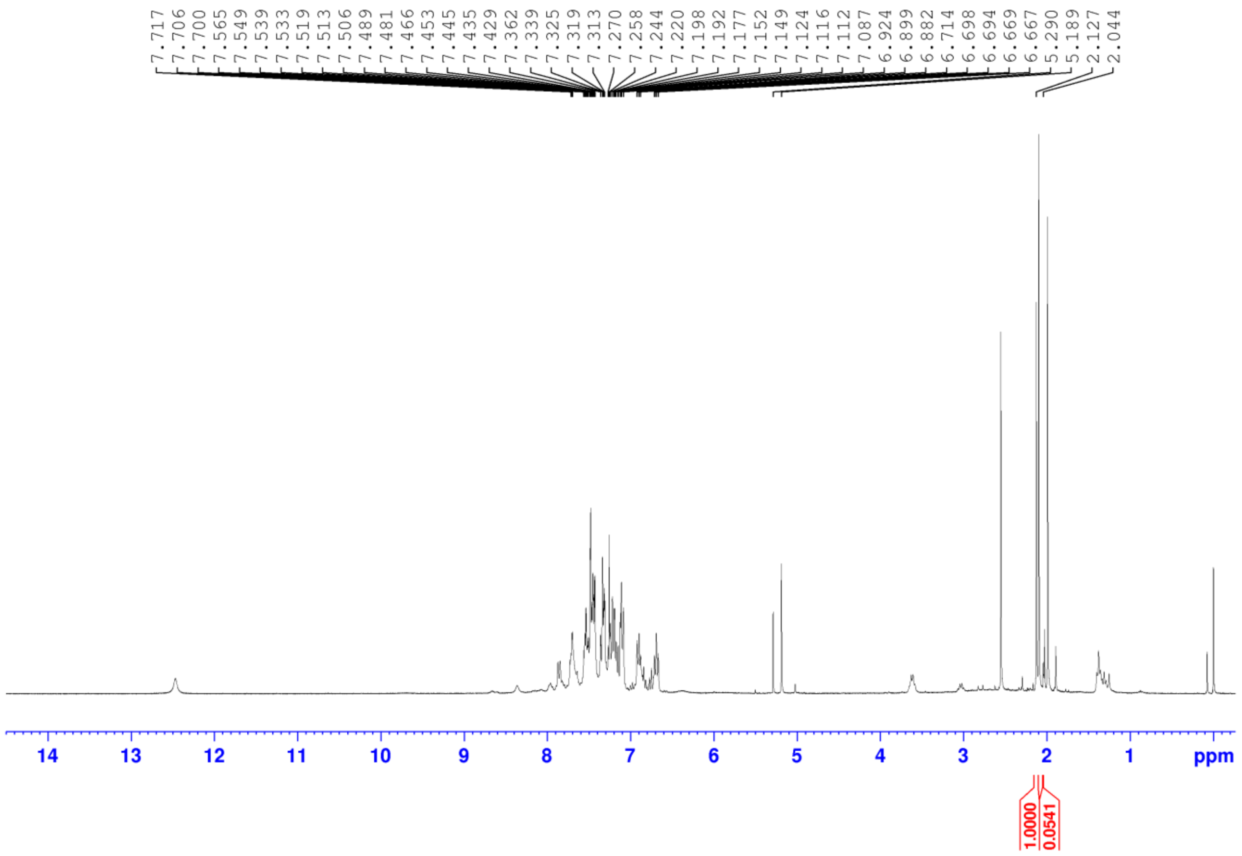
7.717
7.706
7.700
7.565
7.549
7.539
7.533
7.519
7.513
7.506
7.489
7.481
7.466
7.453
7.445
7.435
7.429
7.362
7.339
7.325
7.319
7.313
7.270
7.258
7.244
7.220
7.198
7.192
7.177
7.152
7.149
7.124
7.116
7.112
7.087
6.924
6.899
6.882
6.714
6.698
6.694
6.669
6.667
5.290
5.189
2.127
2.044
14
13
12
11
10
9
8
7
6
5
4
3
2
1
ppm
1.0000
0.0541

Entry 5

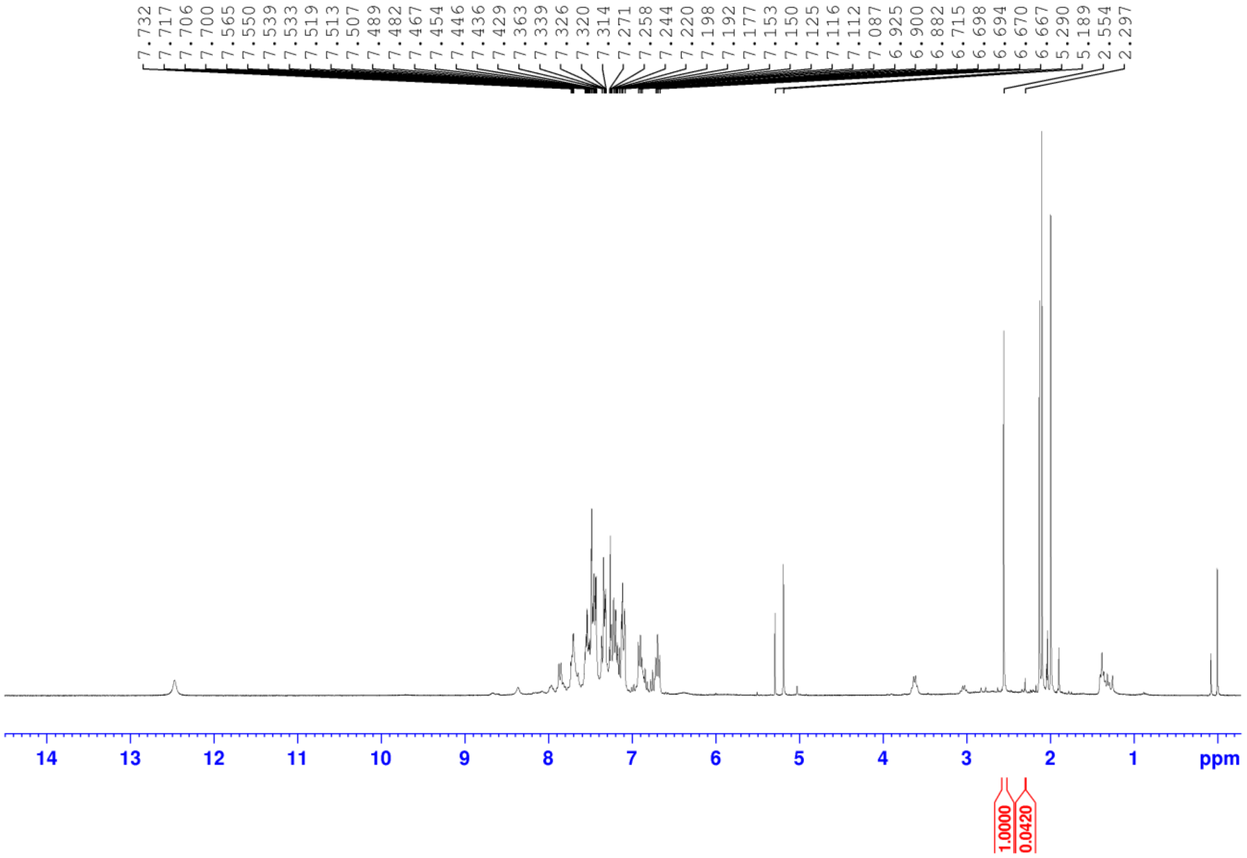
7.732
7.717
7.706
7.700
7.565
7.550
7.539
7.533
7.519
7.513
7.507
7.489
7.482
7.467
7.454
7.446
7.436
7.429
7.363
7.339
7.326
7.320
7.314
7.271
7.258
7.244
7.220
7.198
7.192
7.177
7.153
7.150
7.125
7.116
7.112
7.087
6.925
6.900
6.882
6.715
6.698
6.694
6.670
6.667
5.290
5.189
2.554
2.297
14
13
12
11
10
9
8
7
6
5
4
3
2
1
ppm
1.0000
0.0420

Entry 6

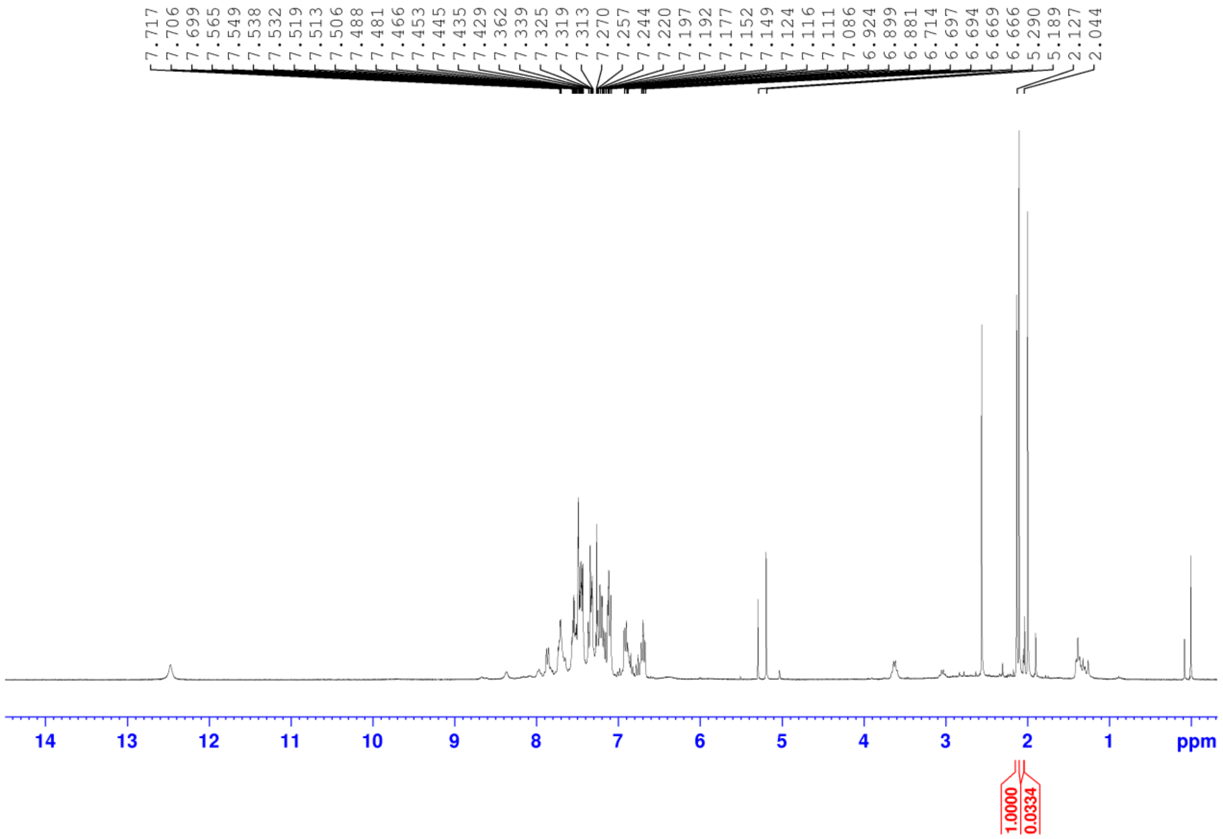

7.717
7.706
7.699
7.565
7.549
7.538
7.532
7.519
7.513
7.506
7.488
7.481
7.466
7.453
7.445
7.435
7.429
7.362
7.339
7.325
7.319
7.313
7.270
7.257
7.244
7.220
7.197
7.192
7.177
7.152
7.149
7.124
7.116
7.111
7.086
6.924
6.899
6.881
6.714
6.697
6.694
6.669
6.666
5.290
5.189
2.127
2.044
14 13 12 11 10 9 8 7 6 5 4 3 2 1 ppm
1.0000
0.0334

Entry 8

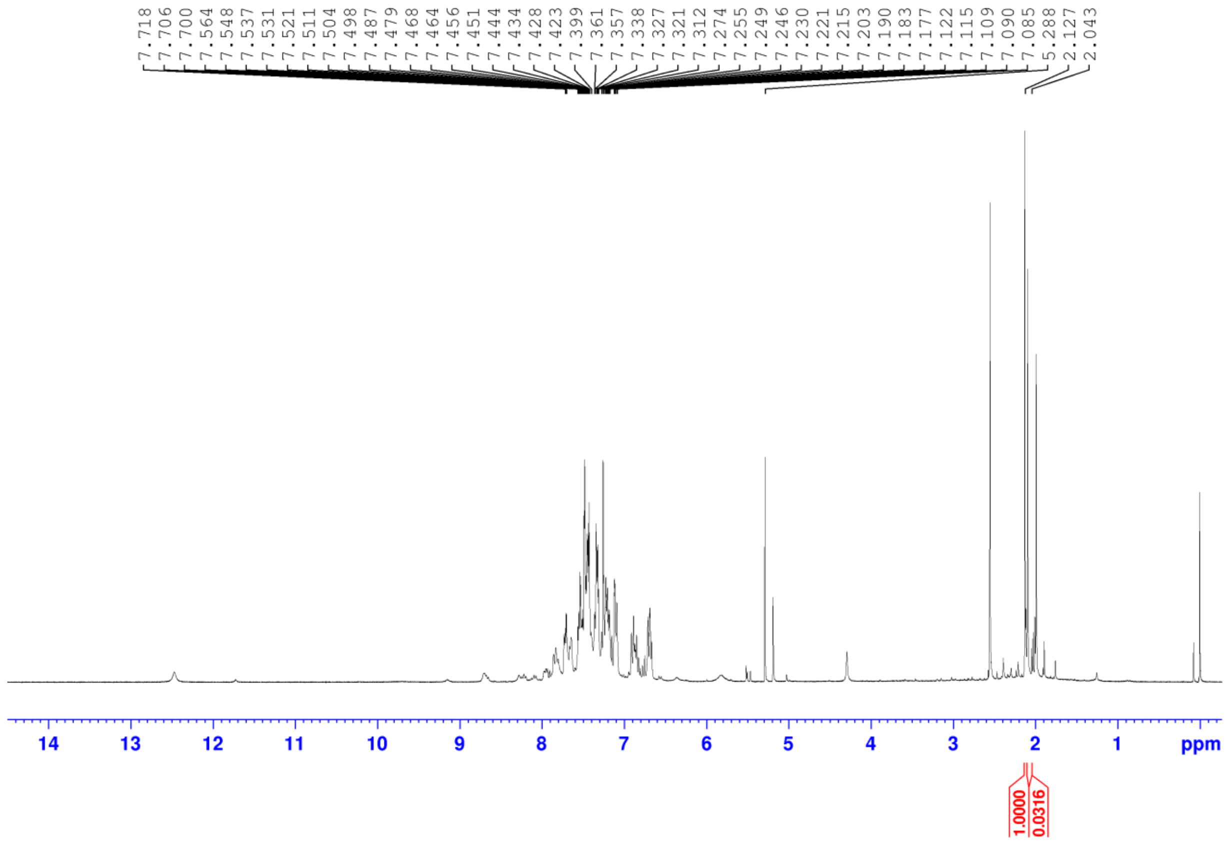

7.718
7.706
7.700
7.564
7.548
7.537
7.531
7.521
7.511
7.504
7.498
7.487
7.479
7.468
7.464
7.456
7.451
7.444
7.434
7.428
7.423
7.399
7.361
7.357
7.338
7.327
7.321
7.312
7.274
7.255
7.249
7.246
7.230
7.221
7.215
7.203
7.190
7.183
7.177
7.122
7.115
7.109
7.090
7.085
5.288
2.127
2.043
14 13 12 11 10 9 8 7 6 5 4 3 2 1 ppm
1.0000
0.0316

Entry 9

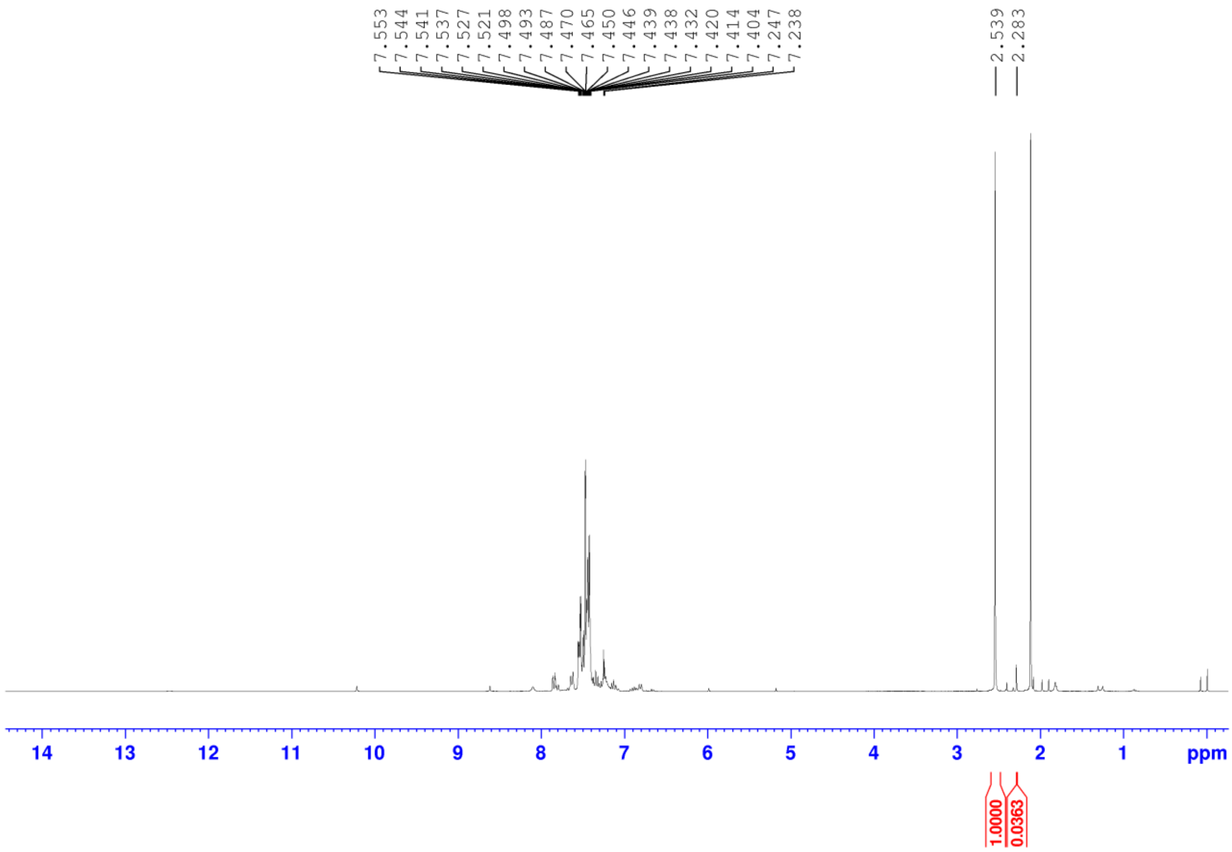
7.553
7.544
7.541
7.537
7.527
7.521
7.498
7.493
7.487
7.470
7.465
7.450
7.446
7.439
7.438
7.432
7.420
7.414
7.404
7.247
7.238
2.539
2.283
14 13 12 11 10 9 8 7 6 5 4 3 2 1 ppm
1.0000
0.0363

Entry 10

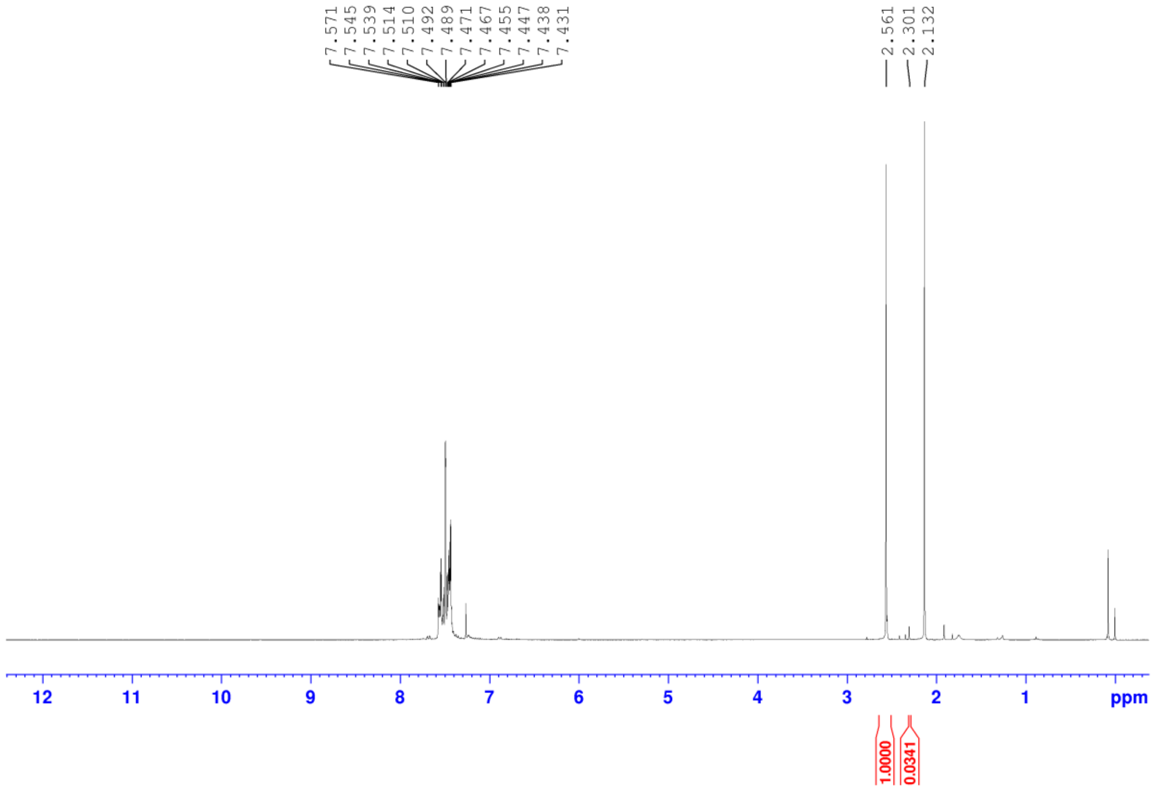
7.571
7.545
7.539
7.514
7.510
7.492
7.489
7.471
7.467
7.455
7.447
7.438
7.431
2.561
2.301
2.132
12
11
10
9
8
7
6
5
4
3
2
1
ppm
1.0000
0.0341

Entry 11

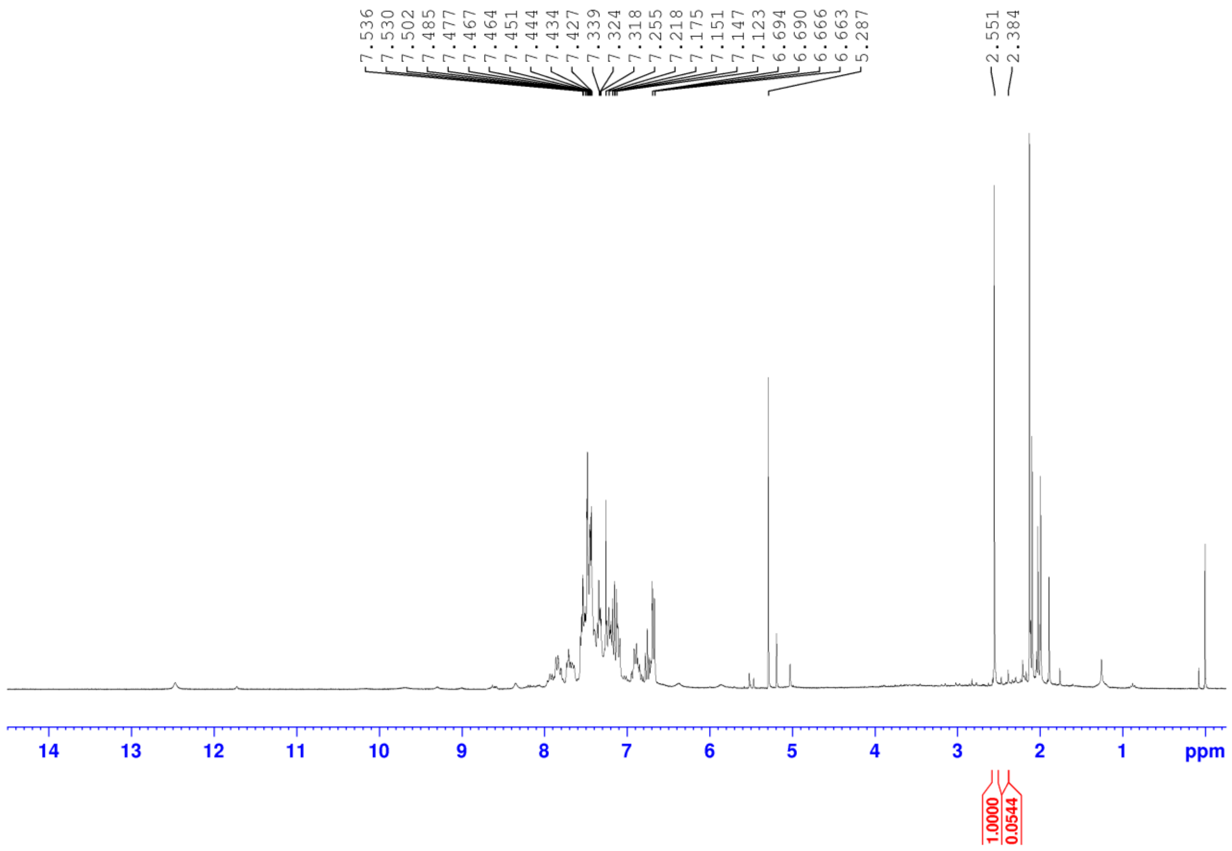

7.536
7.530
7.502
7.485
7.477
7.467
7.464
7.451
7.444
7.434
7.427
7.339
7.324
7.318
7.255
7.218
7.175
7.151
7.147
7.123
6.694
6.690
6.666
6.663
5.287
2.551
2.384
14
13
12
11
10
9
8
7
6
5
4
3
2
1
ppm
1.0000
0.0544

Entry 12

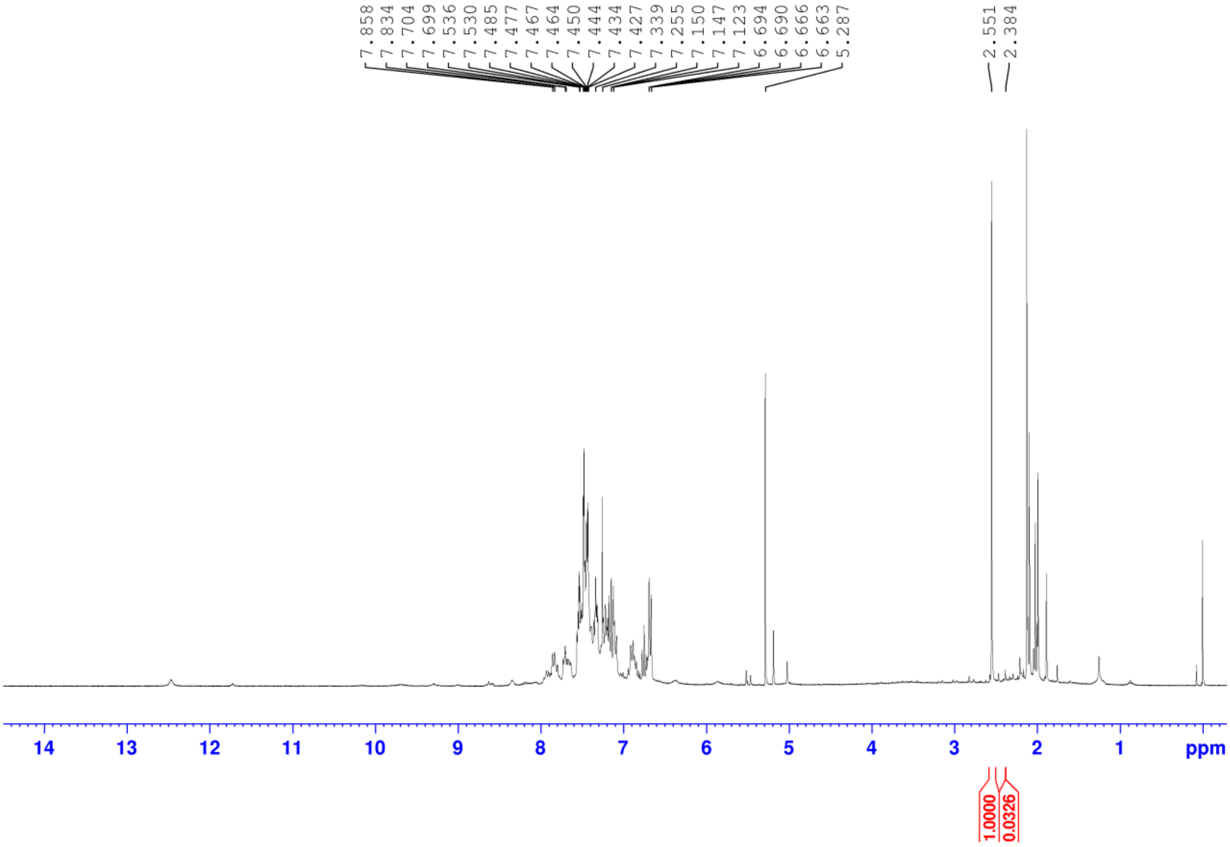
7.858
7.834
7.704
7.699
7.536
7.530
7.485
7.477
7.467
7.464
7.450
7.444
7.434
7.427
7.339
7.255
7.150
7.147
7.123
6.694
6.690
6.666
6.663
5.287
2.551
2.384
14
13
12
11
10
9
8
7
6
5
4
3
2
1
ppm
1.0000
0.0326

Entry 13

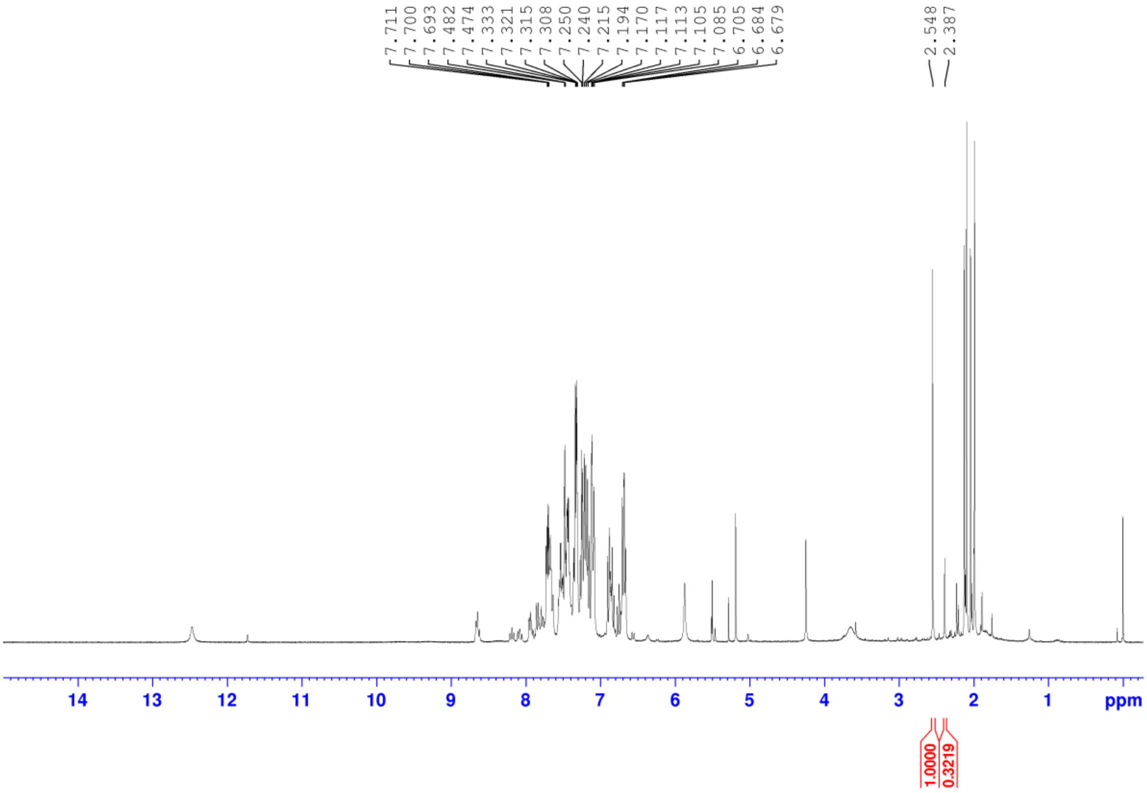
7.711
7.700
7.693
7.482
7.474
7.333
7.321
7.315
7.308
7.250
7.240
7.215
7.194
7.170
7.117
7.113
7.105
7.085
6.705
6.684
6.679
2.548
2.387
14
13
12
11
10
9
8
7
6
5
4
3
2
1
ppm
1.0000
0.3219

Entry 14

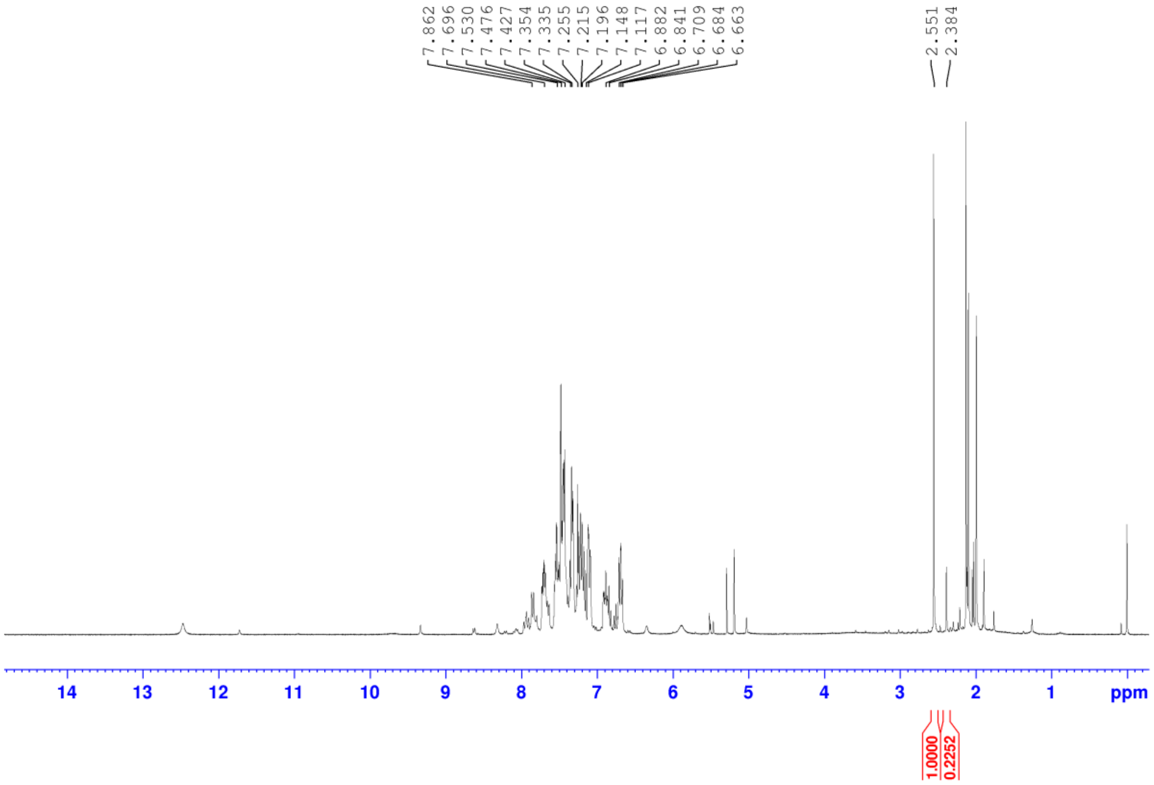
7.862
7.696
7.530
7.476
7.427
7.354
7.335
7.255
7.215
7.196
7.148
7.117
6.882
6.841
6.709
6.684
6.663
2.551
2.384
14
13
12
11
10
9
8
7
6
5
4
3
2
1
ppm
1.0000
0.2252

^{1}H NMR spectra of crude reaction mixtures illustrated in Table 2.

Entry 1

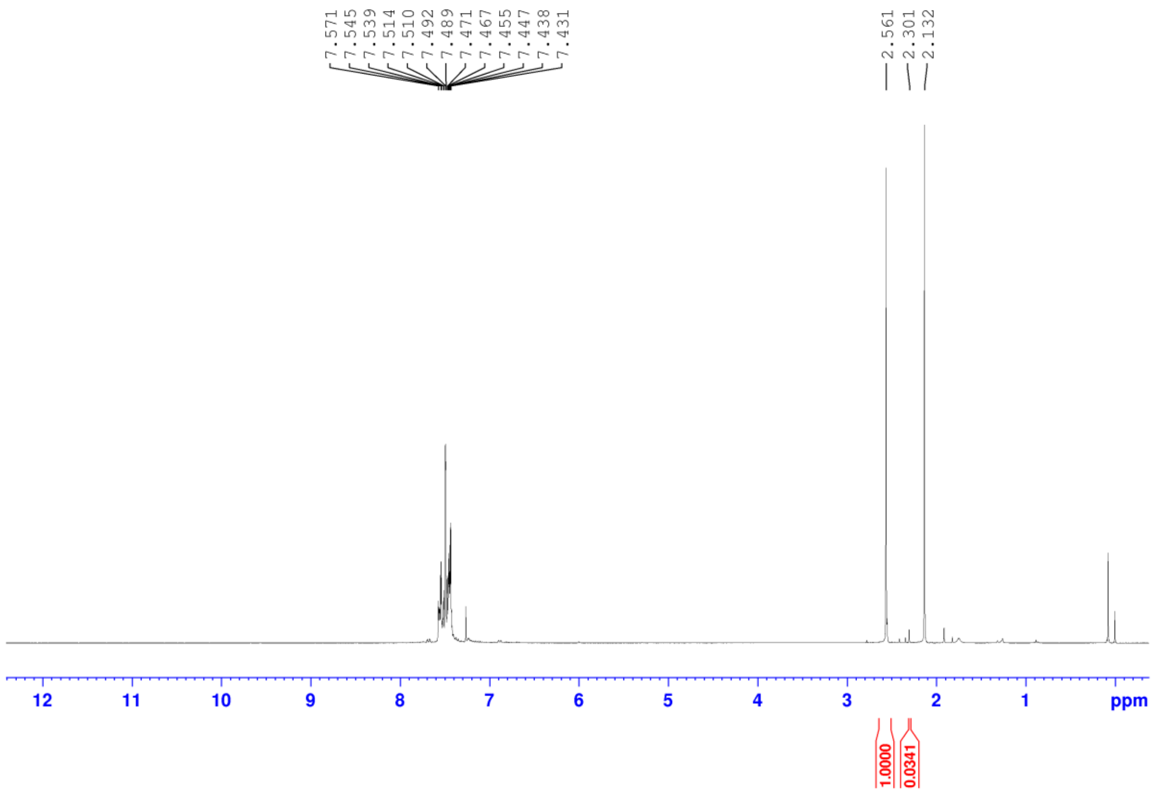

Entry 2

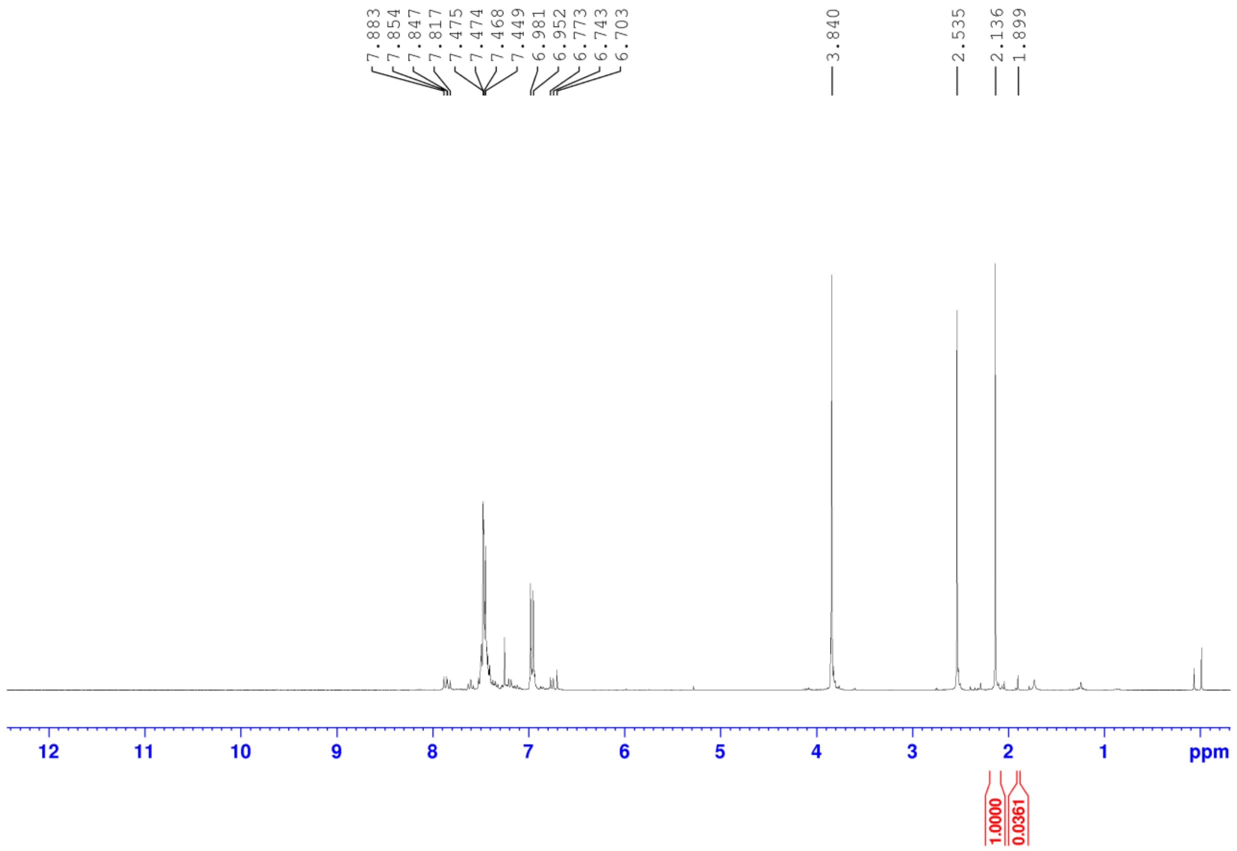
7.883
7.854
7.847
7.817
7.475
7.474
7.468
7.449
6.981
6.952
6.773
6.743
6.703
3.840
2.535
2.136
1.899
12
11
10
9
8
7
6
5
4
3
2
1
ppm
1.0000
0.0361

Entry 3

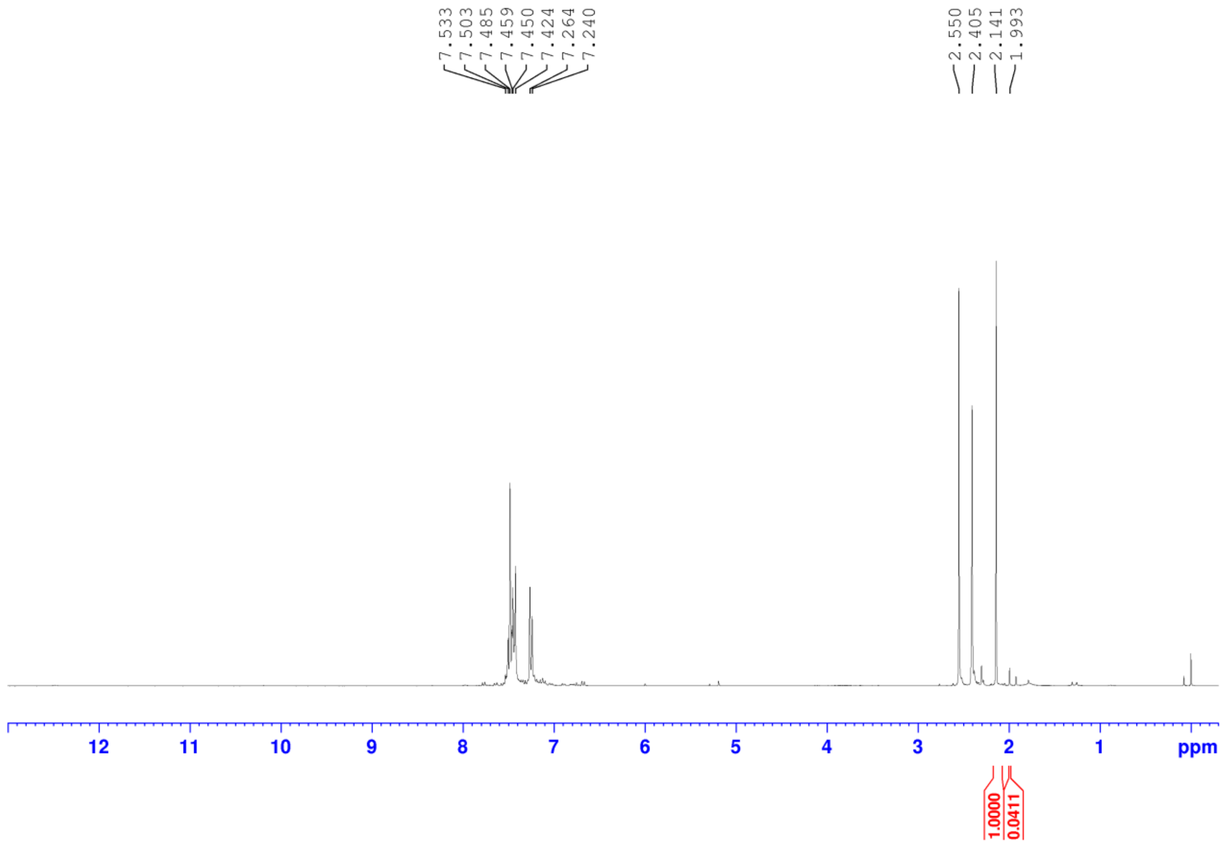
7.533
7.503
7.485
7.459
7.450
7.424
7.264
7.240
2.550
2.405
2.141
1.993
12
11
10
9
8
7
6
5
4
3
2
1
ppm
1.0000
0.0411

Entry 4

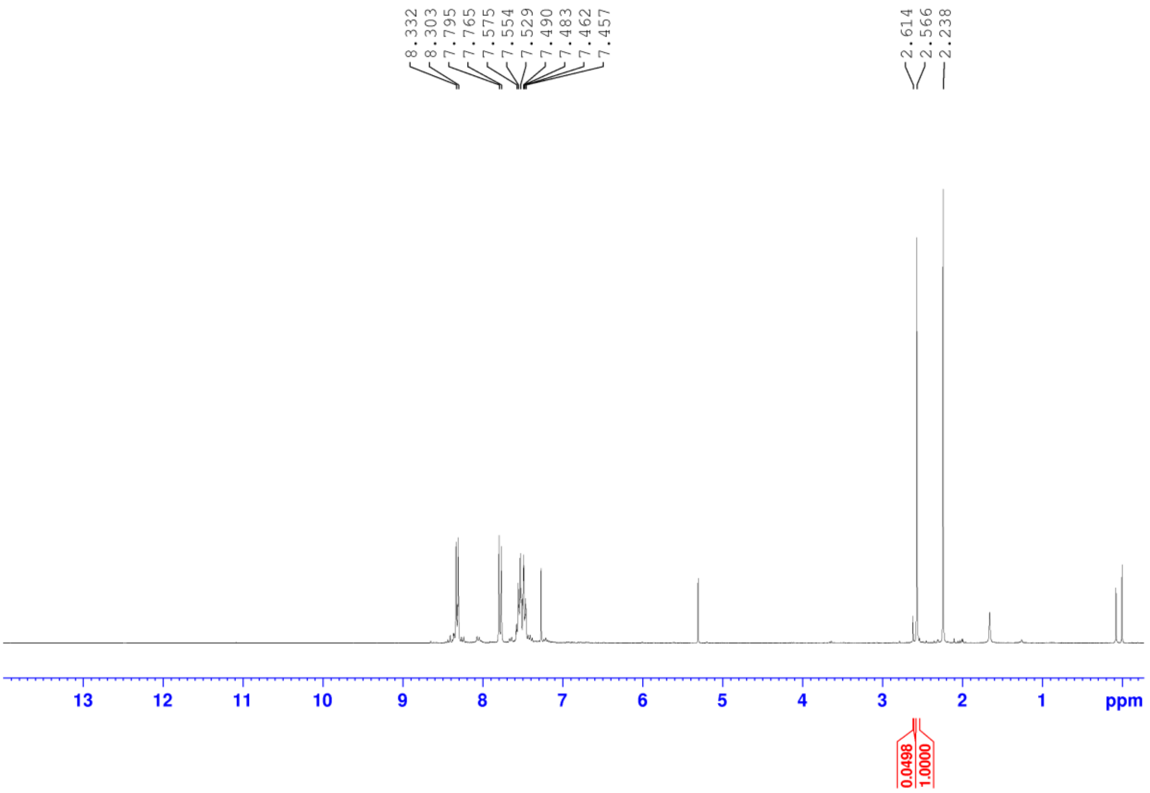

8.332
8.303
7.795
7.765
7.575
7.554
7.529
7.490
7.483
7.462
7.457
2.614
2.566
2.238
13
12
11
10
9
8
7
6
5
4
3
2
1
ppm
0.0498
1.0000

Entry 5

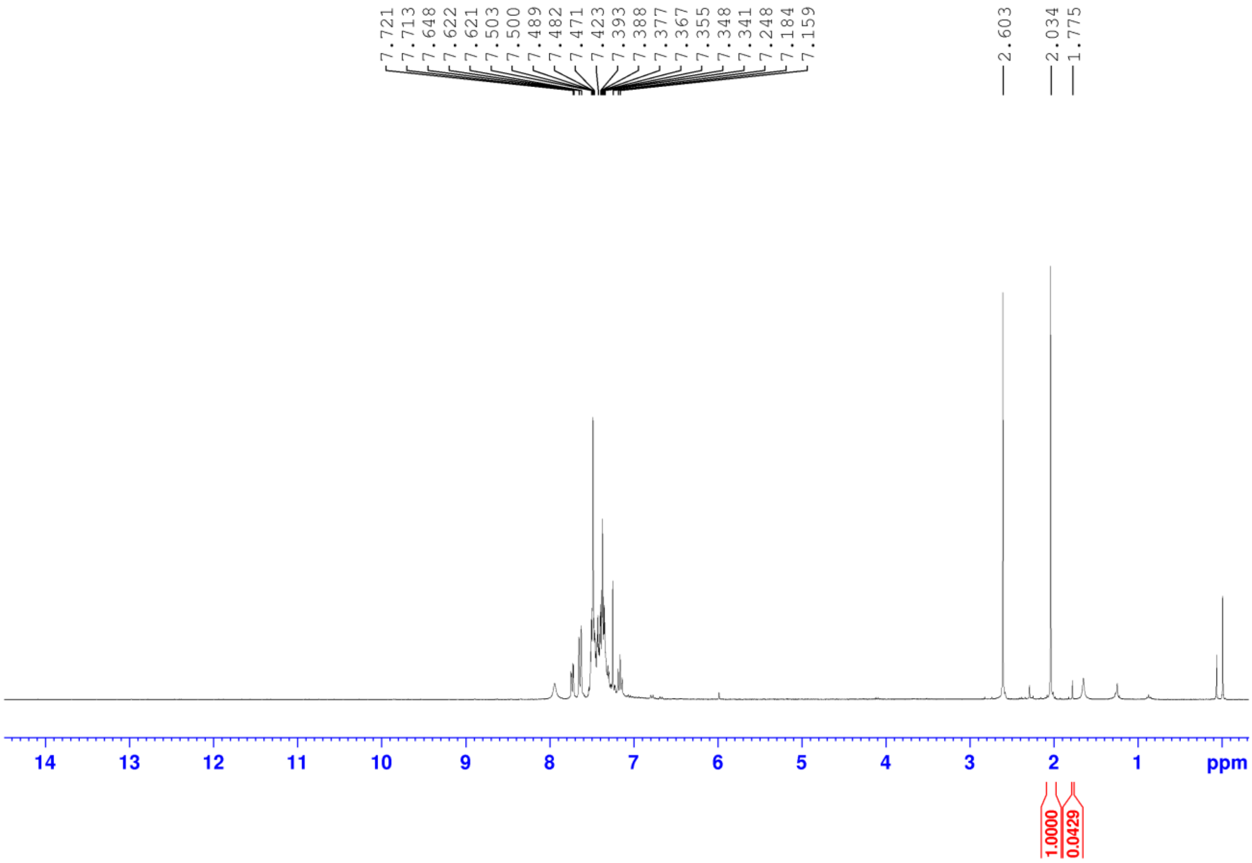

7.721
7.713
7.648
7.622
7.621
7.503
7.500
7.489
7.482
7.471
7.423
7.393
7.388
7.377
7.367
7.355
7.348
7.341
7.248
7.184
7.159
2.603
2.034
1.775
14
13
12
11
10
9
8
7
6
5
4
3
2
1
ppm
1.0000
0.0429

Entry 6

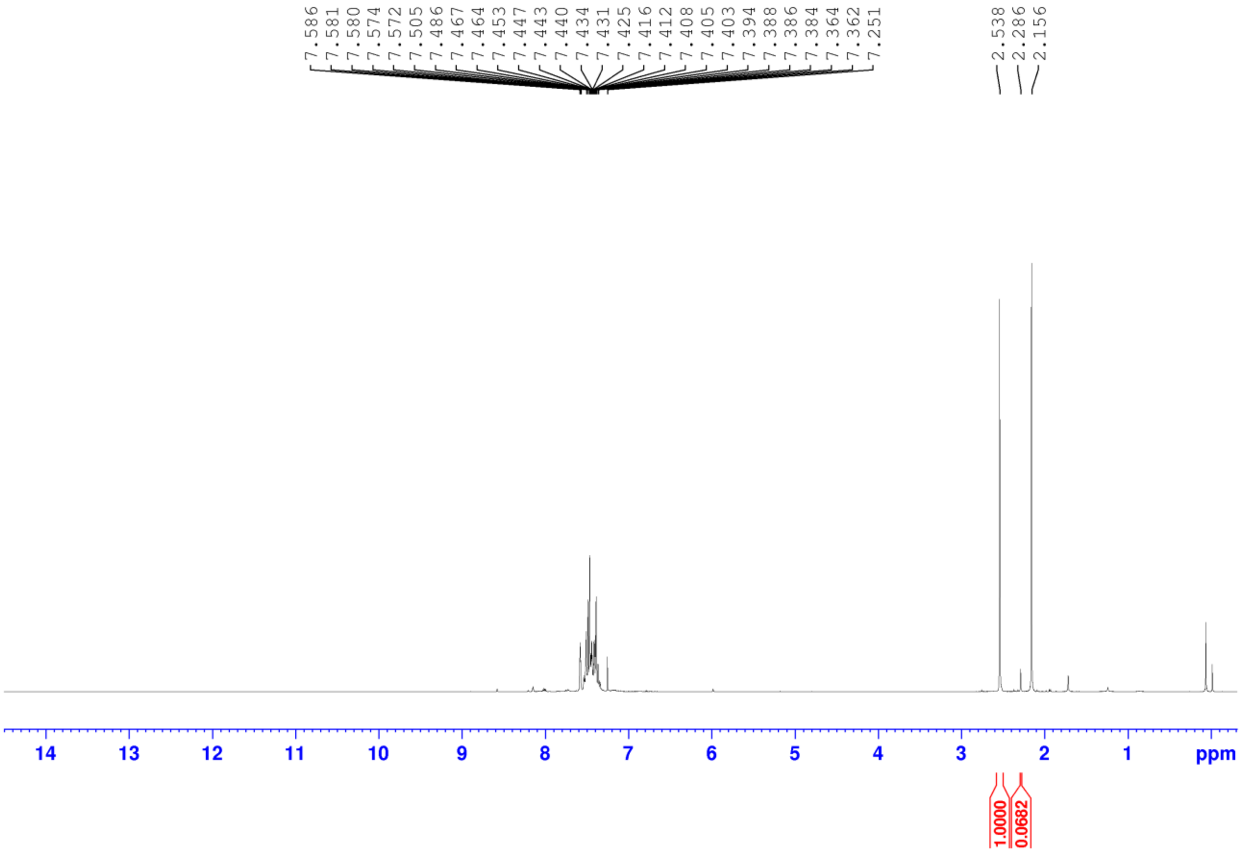
7.586
7.581
7.580
7.574
7.572
7.505
7.486
7.467
7.464
7.453
7.447
7.443
7.440
7.434
7.431
7.425
7.416
7.412
7.408
7.405
7.403
7.394
7.388
7.386
7.384
7.364
7.362
7.251
2.538
2.286
2.156
14
13
12
11
10
9
8
7
6
5
4
3
2
1
ppm
1.0000
0.0682

Entry 7

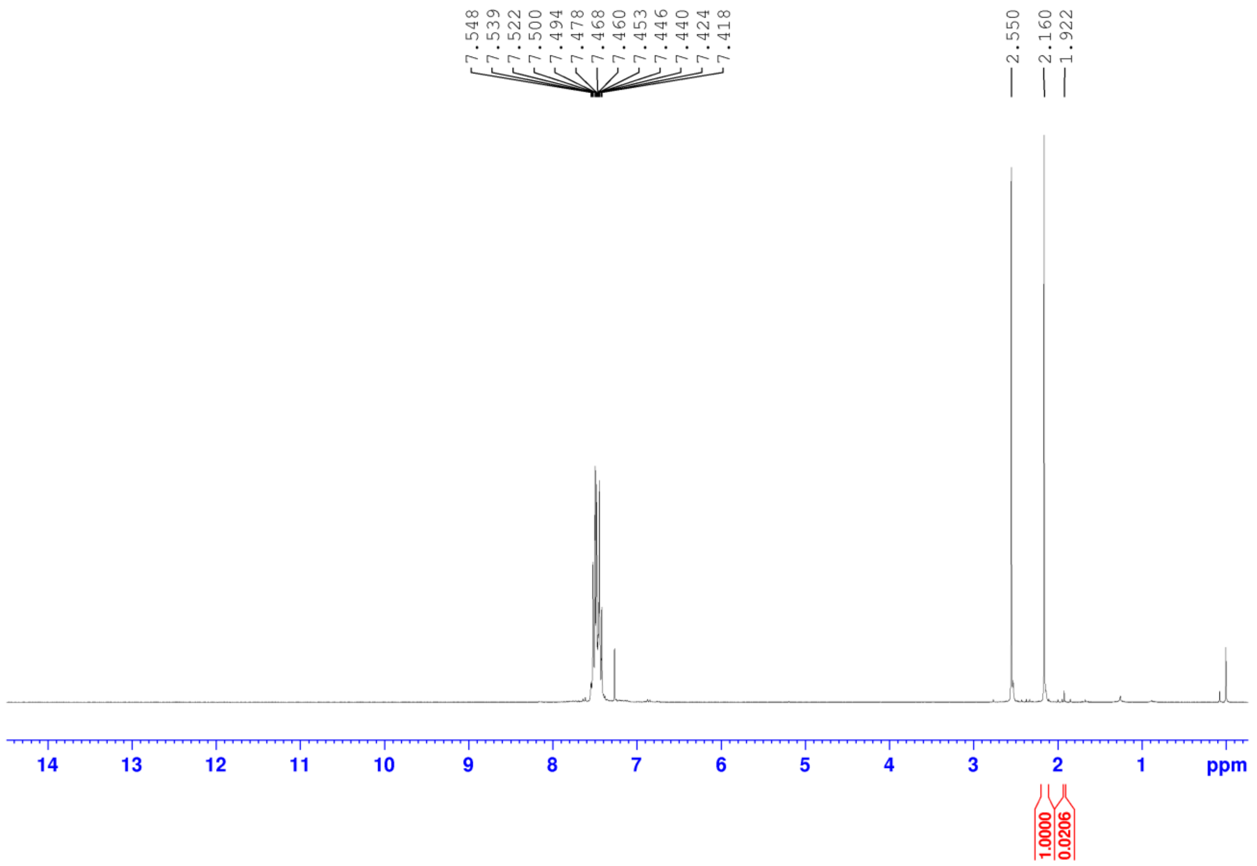

7.548
7.539
7.522
7.500
7.494
7.478
7.468
7.460
7.453
7.446
7.440
7.424
7.418
2.550
2.160
1.922
14
13
12
11
10
9
8
7
6
5
4
3
2
1
ppm
1.0000
0.0206

Entry 8

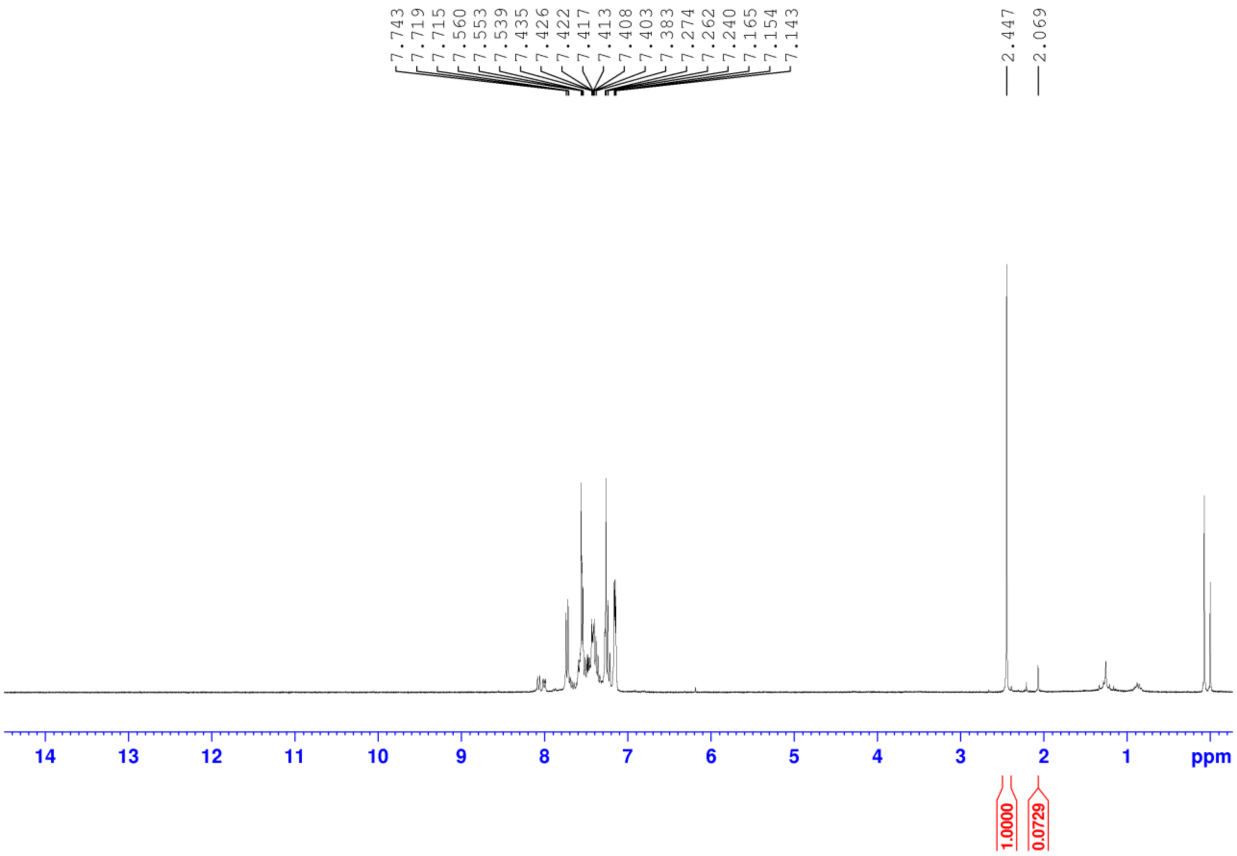

7.743
7.719
7.715
7.560
7.553
7.539
7.435
7.426
7.422
7.417
7.413
7.408
7.403
7.383
7.274
7.262
7.240
7.165
7.154
7.143
2.447
2.069
14
13
12
11
10
9
8
7
6
5
4
3
2
1
ppm
1.0000
0.0729

Entry 9

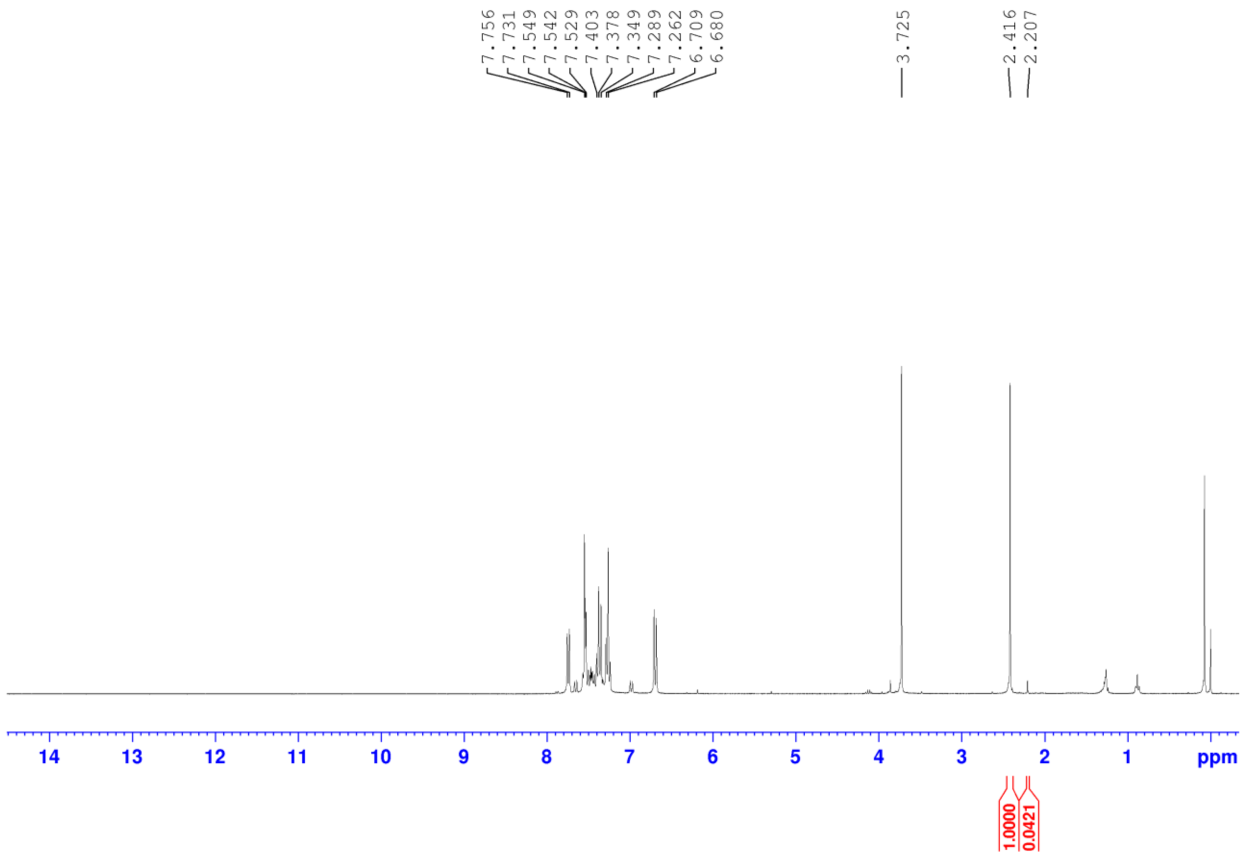
7.756
7.731
7.549
7.542
7.529
7.403
7.378
7.349
7.289
7.262
6.709
6.680
3.725
2.416
2.207
14 13 12 11 10 9 8 7 6 5 4 3 2 1 ppm
1.0000
0.0421

Entry 10

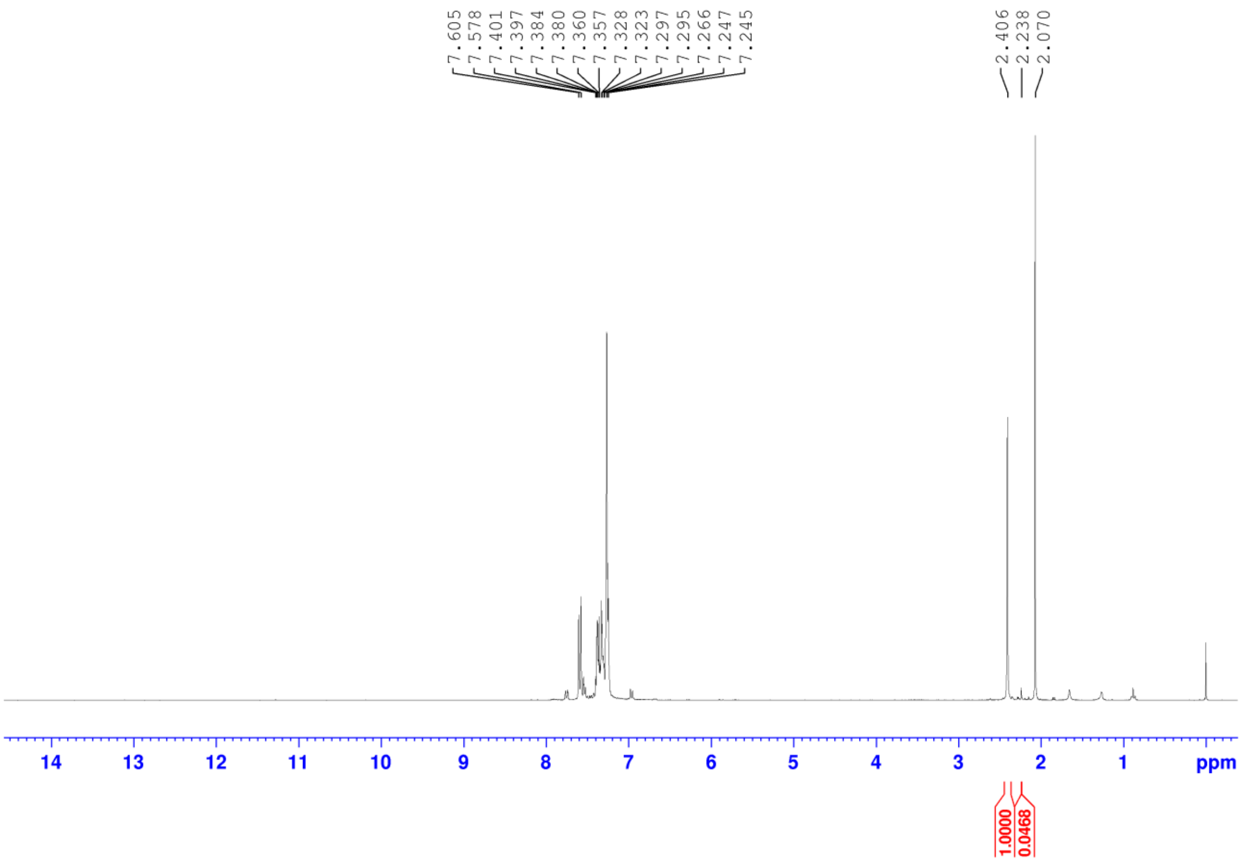

7.605
7.578
7.401
7.397
7.384
7.380
7.360
7.357
7.328
7.323
7.297
7.295
7.266
7.247
7.245
2.406
2.238
2.070
14
13
12
11
10
9
8
7
6
5
4
3
2
1
ppm
1.0000
0.0468

Entry 11

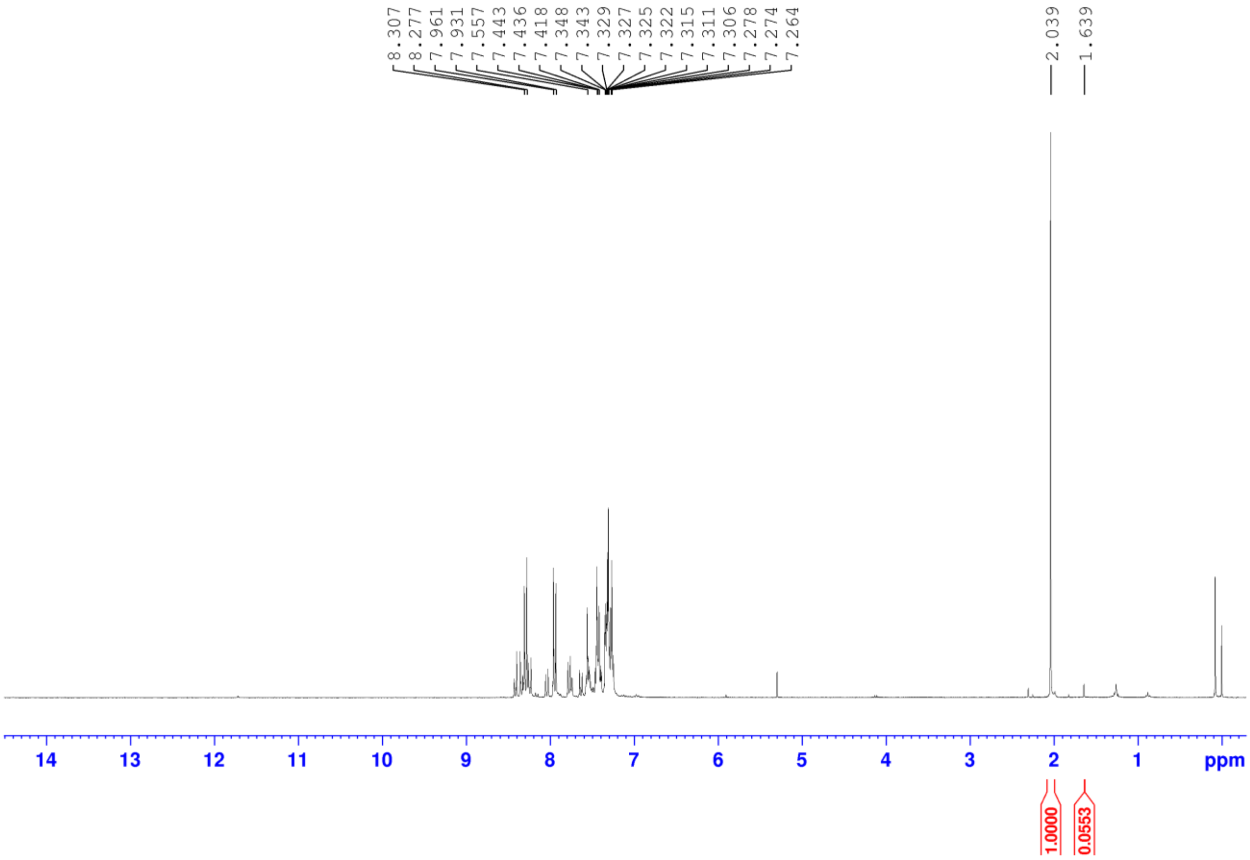
8.307
8.277
7.961
7.931
7.557
7.443
7.436
7.418
7.348
7.343
7.329
7.327
7.325
7.322
7.315
7.311
7.306
7.278
7.274
7.264
2.039
1.639
14
13
12
11
10
9
8
7
6
5
4
3
2
1
ppm
1.0000
0.0553

Entry 12

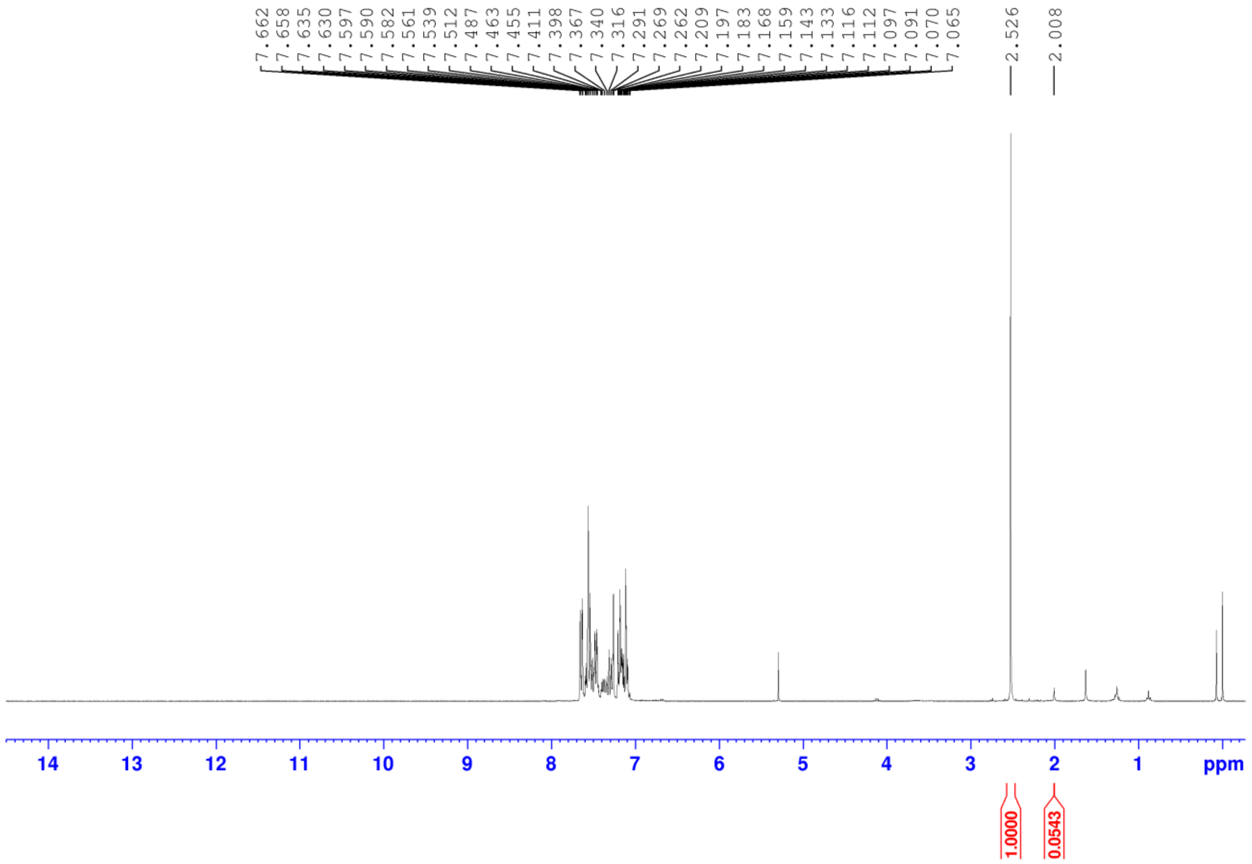

7.662
7.658
7.635
7.630
7.597
7.590
7.582
7.561
7.539
7.512
7.487
7.463
7.455
7.411
7.398
7.367
7.340
7.316
7.291
7.269
7.262
7.209
7.197
7.183
7.168
7.159
7.143
7.133
7.116
7.112
7.097
7.091
7.070
7.065
2.526
2.008
14
13
12
11
10
9
8
7
6
5
4
3
2
1
ppm
1.0000
0.0543

Entry 13

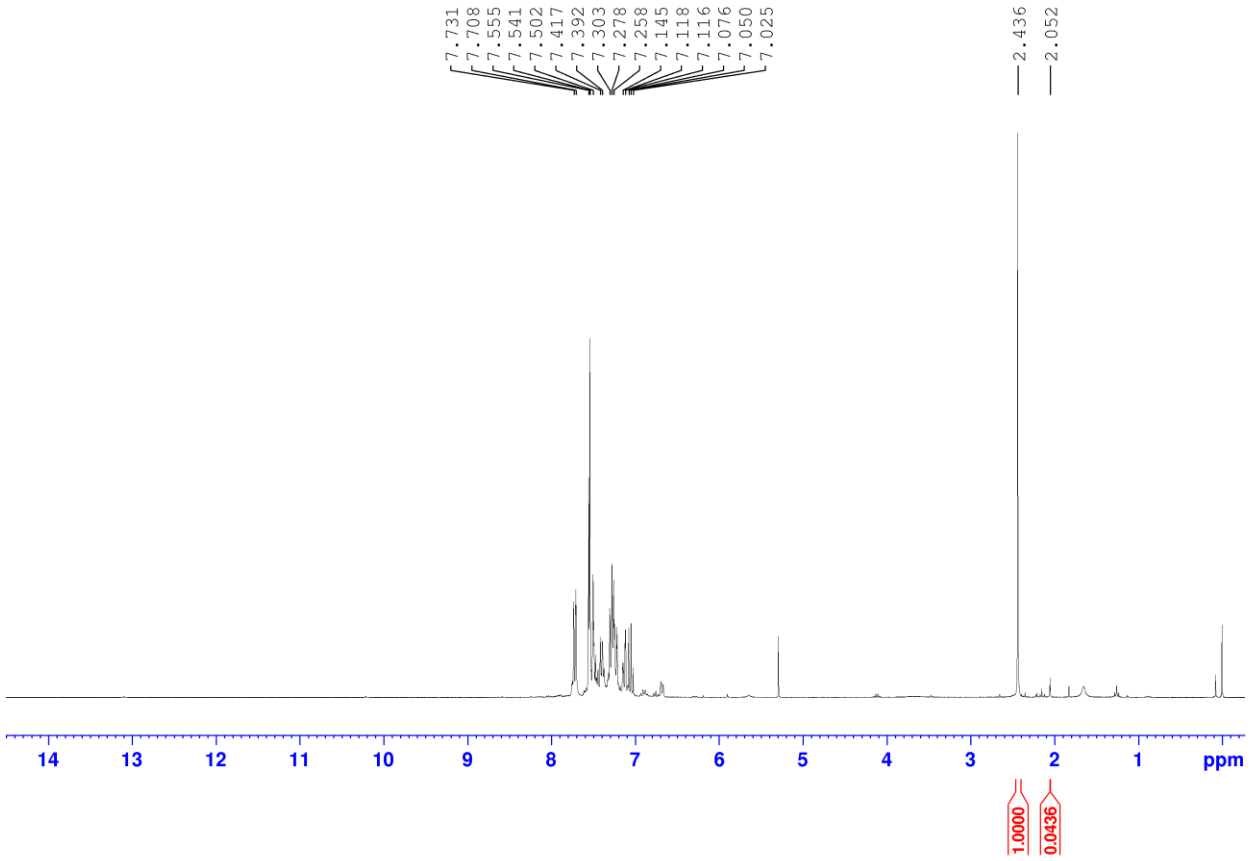
7.731
7.708
7.555
7.541
7.502
7.417
7.392
7.303
7.278
7.258
7.145
7.118
7.116
7.076
7.050
7.025
2.436
2.052
14
13
12
11
10
9
8
7
6
5
4
3
2
1
ppm
1.0000
0.0436

Entry 14

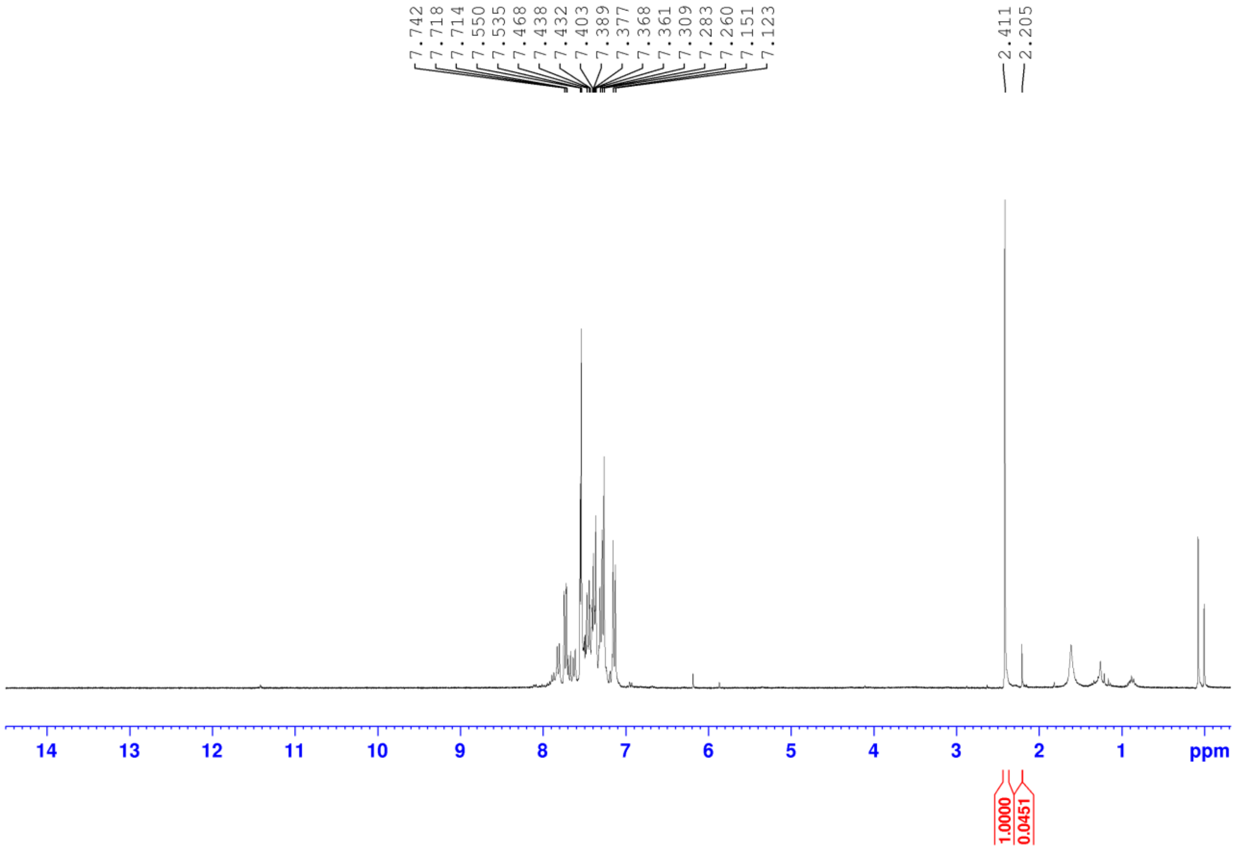
7.742
7.718
7.714
7.550
7.535
7.468
7.438
7.432
7.403
7.389
7.377
7.368
7.361
7.309
7.283
7.260
7.151
7.123
2.411
2.205
14
13
12
11
10
9
8
7
6
5
4
3
2
1
ppm
1.0000
0.0451

Entry 15

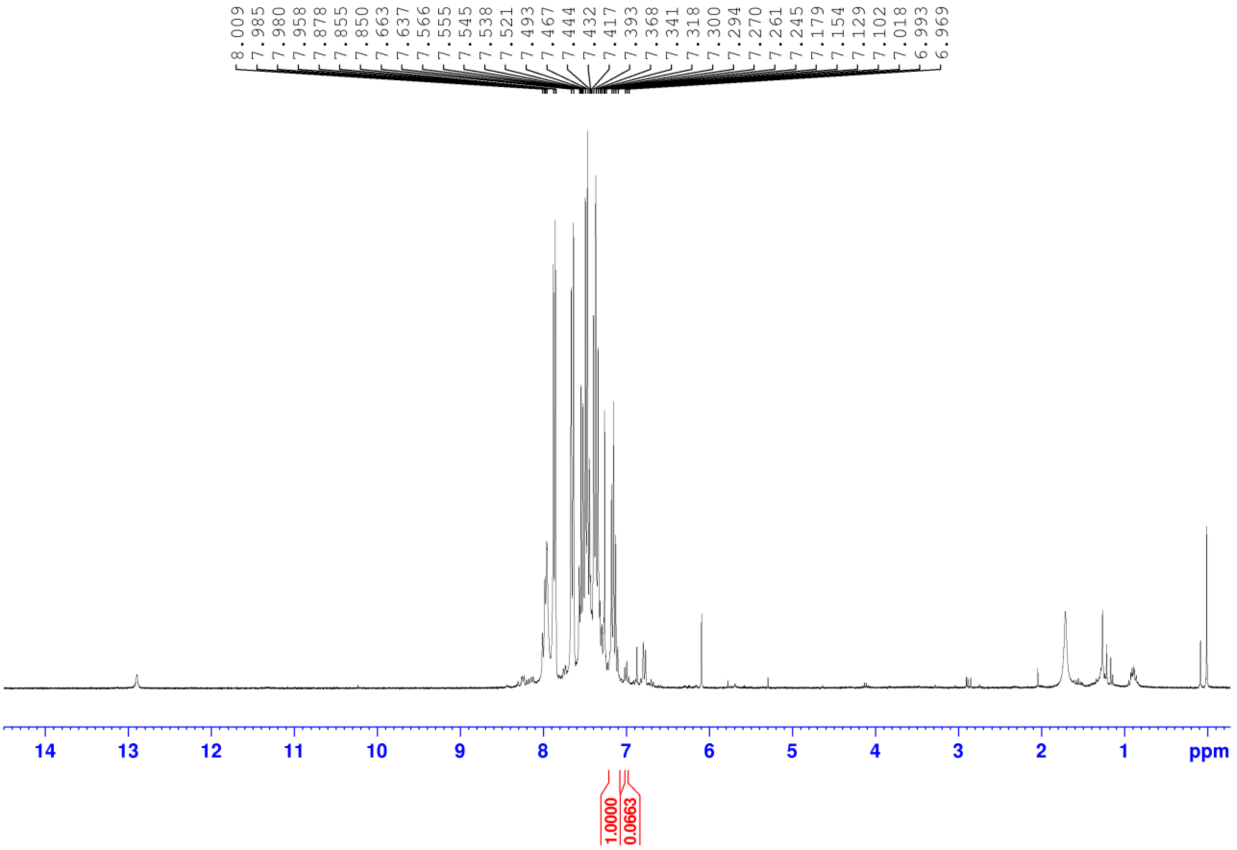
8.009
7.985
7.980
7.958
7.878
7.855
7.850
7.663
7.637
7.566
7.555
7.545
7.538
7.521
7.493
7.467
7.444
7.432
7.417
7.393
7.368
7.341
7.318
7.300
7.294
7.270
7.261
7.245
7.179
7.154
7.129
7.102
7.018
6.993
6.969
14 13 12 11 10 9 8 7 6 5 4 3 2 1 ppm
1.0000
0.0663

References

1 Gallagher, N.; Zangh, H.; Junghoefer, T.; Giangrisostomi, E.; Ovsyannikov, R.; Pink, M.; Rajca, S.; Casu, M.B.; Rajca. A. Termally and magnetically robust triplet ground state diradical. *J.Am.Chem.Soc.*, **2019**, *141*, 4764–4774.

2 Zhang, C.-Y.; Liu, X.-H.; Wang, B.-L.; Wang, S.-H.; Li, Z.-M. Synthesis and antifungal activities of new pyrazole derivatives via 1,3-dipolar cycloaddition reaction. *Chem. Biol. Drug. Des.*, **2010**, *75*, 489–493.

3 De Nino, A.; Maiuolo, L.; Merino, P.; Nardi, M.; Procopio, A.; Roca-López, D.; Russo, B.; Algieri, V. Efficient Organocatalyst Supported on a Simple Ionic Liquid as a Recoverable System for the Asymmetric Diels-Alder Reaction in the Presence of Water. *ChemCatChem*, **2015**, *7*, 830–835.
